# The spectrum of communication abilities in children with 12 rare neurodevelopmental disorders: a qualitative study with caregivers

**DOI:** 10.1111/jcpp.70063

**Published:** 2025-10-20

**Authors:** Christina K. Zigler, Molly McFatrich, Nicole Lucas, Kate Plyler, Leslie Zapata‐Leiva, Kelly Gordon, Harrison N. Jones, Li Lin, Jennifer Kern, Abigail Radar, Dandan Chen, Elika Bergelson, Kate Still, Brigette Hinger, Christal G. Delagrammatikas, Sarah Poliquin, Brittany P. Short, Liz Marfia‐Ash, Kimberly Stephens, Haley O. Oyler, J Michael Graglia, Kali Worth, Charlene Son Rigby, James R. Goss, Bo Bigelow, Geraldine Bliss, Karen Beatty, Leah Schust Myers, Melissa Thelen, Nuala Summerfield, Terry Jo Bichell, Bryce B. Reeve

**Affiliations:** ^1^ Department of Physical Medicine & Rehabilitation University of Pittsburgh School of Medicine Pittsburgh PA USA; ^2^ Department of Population Health Sciences Duke University School of Medicine Durham NC USA; ^3^ Center for Communication Science RTI International Research Triangle Park NC USA; ^4^ Duke Health Durham NC USA; ^5^ Department of Head and Neck Surgery & Communication Sciences Duke University School of Medicine Durham NC USA; ^6^ Department of Psychology Harvard University Cambridge MA USA; ^7^ Phelan‐McDermid Syndrome Foundation Laurel FL USA; ^8^ NR2F1 Foundation Pflugerville TX USA; ^9^ Malan Syndrome Foundation Old Bridge NJ USA; ^10^ COMBINEDBrain Brentwood TN USA; ^11^ GRIN2B Foundation Niles IL USA; ^12^ Muenzer MPS Research and Treatment Center at UNC Chapel Hill NC USA; ^13^ SETBP1 Society Austin TX USA; ^14^ SYNGAP Research Fund San Diego CA USA; ^15^ STXBP1 Foundation Holly Springs NC USA; ^16^ Foundation for Hao‐Fountain Syndrome Falmouth ME USA; ^17^ CureSHANK Los Angeles CA USA; ^18^ Project Alive Davenport FL USA; ^19^ FamilieSCN2A Foundation Gettysburg PA USA; ^20^ The Schinzel‐Giedion Syndrome Foundation West Sussex UK

**Keywords:** Communication, non‐verbal communication, behavioural measures, qualitative methods, caregiver

## Abstract

**Background:**

Our aim was to update an existing model of communication ability for children with rare neurodevelopmental disorders (NDDs) by centring caregiver and family perspectives. This project is part of a larger initiative to improve the measurement of communication ability for these children in the context of clinical trials.

**Methods:**

We conducted concept elicitation interviews with purposively selected clinical experts and caregivers of children with 12 NDDs, focusing on a broad definition of communication ability based on the Observer‐Reported Communication Ability (ORCA) measure, which is inclusive of different communication modalities and covers expressive, receptive and pragmatic communication concepts. Content‐based and thematic analysis was performed on the qualitative data.

**Results:**

Altogether, 115 interviews were conducted with caregivers across the 12 NDDs and with 9 clinicians. Commonly mentioned concepts across NDDs included requesting an object, refusing an object, responding to familiar directions and seeking attention. There was notable heterogeneity within and across NDD groups in terms of the specific communication behaviours described for each communication concept. One common example was requesting; children used verbal speech, gestures, sign language, eye gaze, body movements and augmentative and assistive communication to ask for what they wanted. Novel communication concepts identified that were not part of the existing model were (1) feelings, emotions, and bodily sensations, (2) commenting on likes and dislikes, and (3) communicating and understanding humour.

**Conclusions:**

Caregivers offered a detailed and nuanced picture of their child's day‐to‐day communication. There was a considerable overlap between the communication concepts discussed by caregivers in the interviews and the existing conceptual model of communication ability. Some newly identified concepts underscore the need for further adaptation of the model and subsequent validation of any clinical outcome assessment before communication ability can be confidently measured for these individuals in clinical trials.

## Introduction

For typically developing children, the presence of a language‐rich environment is sufficient to facilitate the development of critical communication skills (Brock & Rankin, 2008). However, for children with neurodevelopmental disorders (NDDs), a broad term referring to multiple conditions impacting brain development and function (Parenti, Rabaneda, Schoen, & Novarino, 2020), an optimal environment may not be adequate. Delays in communication milestones are often one of the first signs of an NDD that caregivers notice (Burdeus‐Olavarrieta, Nevado, van Weering‐Scholten, Parker, & Swillen, 2023; Luyster, Seery, Talbott, & Tager‐Flusberg, 2011). Depending on the specific diagnosis and underlying genetic cause, children with NDDs may experience atypical trajectories of development, which can include communication delays or overall limitations in skills (Bain et al., 2021; Berg, Palac, Wilkening, Zelko, & Schust Meyer, 2021; Droogmans, Swillen, & Van Buggenhout, 2020; Fountain et al., 2019; Freunscht et al., 2013; Holder, Hamdan, & Michaud, 1993; Morgan et al., 2021; O'Brien, Ng‐Cordell, Astle, Scerif, & Baker, 2019; Rech et al., 2020). For some children with NDDs, communication skills can develop and then be lost due to regression (Soorya et al., 2013) or to the natural progression of disease (Acuna‐Hidalgo et al., 2017; Hashmi, 2021). Even for children with verbal speech abilities, expressive language difficulties may present, increasing the need for support from their communication partners or the use of augmentative and alternative communication (AAC) systems (Mulder et al., 2020).

For families caring for a child with a NDD, functional abilities like communication can mediate the relationship between diagnosis and outcomes, including health of parents, economic well‐being of the family, and the child's participation in activities (Miller, Shen, & Mâsse, 2016). Along with our current knowledge of the impacts on caregivers of children with NDDs (Boettcher, Boettcher, Wiegand‐Grefe, & Zapf, 2021; Cohen et al., 2022; Gallop, Lloyd, Olt, & Marshall, 2021; Robertson et al., 2024), it is then unsurprising that caregivers consistently select ‘improvements in communication’ as a top priority for future research and treatment studies (Bliss & Palaty, 2023; Willgoss et al., 2020).

Despite its crucial importance, measuring communication ability is challenging for children with atypical communication development. Existing measures often focus primarily on the typical progression of verbal speech, and by excluding children with NDDs and those that use assistive technology from normative value calculations, floor effects then limit the usefulness of scores in evaluating improvement (Loveall, Channell, Mattie, & Barkhimer, 2022). Despite efforts to quantitatively explore alternatives to traditional norm‐referenced scores using existing measures (Farmer, Thurm, Troy, & Kaat, 2023), there remains an overall dearth of family involvement in measure development and evaluation, resulting in a gap between the interpretation of measurement scores and meaningful life experiences (U.S. Department of Health and Human Services et al., 2022). Commonly, rigorous qualitative work for measure development is conducted for one disease group at a time, making measurement development inefficient for the rare disease community as a whole (Keeley et al., 2024). Further, measures often lack validity support for adolescents and adults (Berg, Kaat, Zelko, & Wilkening, 2022; Chadwick, Buell, & Goldbart, 2019), as communication measures that do include preverbal skills are mostly designed for very young children. Even within NDD clinical trials, which include additional regulatory oversight over measurement selection, the outcome measures utilized have been evaluated as being *moderate* in quality (Budimirovic et al., 2017). Thus, we must improve the measurement of communication abilities for individuals with NDDs, as without high‐quality measures, we cannot fully understand the natural history of a NDD, track symptoms within clinical settings or evaluate treatment efficacy in terms of communication.

The first step in measurement development is to clearly define the concept of interest (U.S. Department of Health and Human Services et al., 2022). Qualitative work is essential at this stage to understand the phenomena and provide documented support for content validity. In clinical trials, there is continued importance placed on gathering input from the target population when selecting, modifying or developing measures (Patrick et al., 2011a). This focus on formative qualitative work is in line with the shift in focus towards acknowledging families as full partners in clinical care and research settings (Ronen, 2022). As stated above, there is a lack of qualitative evidence supporting the validity of existing measures of communication that include caregivers of children with NDDs and experienced clinicians. Thus, the objective of this study was to conduct qualitative interviews with caregivers of individuals with 12 NDDs (Table 1), as well as with clinicians who have experience working with these children to identify core communication concepts and the range of communication abilities. Caregivers are experts on their children, communicating with them in multiple settings within their daily routines, as well as supporting their communication development. Clinical experts can provide a useful understanding of the range of communication ability across children, which is helpful context, especially in rare diseases.

**Table 1:** Neurodevelopmental disorders participating in this project (please note, seizures and epilepsy are a feature of all the disorders in at least a subset of the population)

Neurodevelopmental disorder	Advocacy organization partner	Incidence/features					
		Predicted de novo disease incidence in 100,000 births or (total number of known individuals)	Gene affected	Neurodegenerative	Hearing or vision loss common	Clinical diagnosis/clear cut dysmorphology	Yes‐linked
Bosch–Boonstra–Schaaf optic atrophy syndrome (BBSOAS)	NR2F1 Foundation	[~200]^a^	NR2F1		Yes – vision common, hearing in subset		
GRIN2B‐related neurodevelopmental disorder	GRIN2B Foundation	6^b^	GRIN2B				
SYNGAP1‐related intellectual disability	SynGAP Research Fund (SRF)	6^b^	SYNGAP1				
Hao‐Fountain Syndrome (HAOFOUS)	Foundation for USP7‐Related Diseases	[~250]^c^	USP7				
HNRNPH2‐related disorders	Yellow Brick Road Project	[~150]^d^	HNRNPH2				Yes – F
Hunter syndrome (MPS‐II)	Project Alive	0.6^e^	IDS	Yes	Yes – hearing loss/profound severe	Yes	Yes – M
Malan syndrome	Malan Syndrome Foundation	2.6^b^	NFIX		Yes – vision common, hearing in subset	Yes	
Phelan McDermid syndrome (PMS)	CureSHANK and Phelan‐McDermid Syndrome Foundation	[~2,200 – 2,500]^f^	SHANK3				
Schinzel–Giedion syndrome (SGS)	Schinzel–Giedion Syndrome Foundation	[>50]^g^	SETBP1	Yes	Yes	Yes	
SCN2A‐related disorders (SRD)	FamilieSCN2A Foundation	8^b^	SCN2A				
SETBP1 haploinsufficiency disorder (SETBP1‐HD)	SETBP1 Society	0.5^b^	SETBP1		Yes –vision/mild		
STXBP1‐related disorders	STXBP1 Foundation	3.6^b^	STXBP1	Unclear	Mild to moderate cortical visual impairment (CVI)		

^a^ Monnier C. BBSOAS guides/Las Guías BBSOAS. NR2F1 Foundation. https://nr2f1.org/bbsoas‐guides‐las‐guias‐bbsoas/.

^b^ López‐Rivera et al. (2020).

^c^ Accessed 9/27/2024. https://www.usp7.org/.

^d^ Accessed 9/27/2024. https://yellowbrickroadproject.org/pages/hnrnph2‐genetics‐101.

^e^ Hashmi MS GVI (Hashmi, 2021).

^f^ Accessed 9/27/2024. https://my.clevelandclinic.org/health/diseases/23087‐phelan‐mcdermid‐syndrome.

^g^ Duis and van Bon (2024).

This work was funded by the U.S. Food and Drug Administration (FDA) Center for Drug Evaluation and Research (CDER) Pilot Grant Program to develop core clinical outcome assessments and their related endpoints. During analysis, we leveraged a pre‐existing conceptual model of communication ability. The Observer‐Reported Communication Ability (ORCA) measure was based on this model and developed using qualitative and quantitative input from caregivers of individuals with Angelman syndrome and further evaluated for individuals with Rett syndrome (Reeve et al., 2023; Zigler et al., 2023). This model is inclusive of the many different modalities (e.g. gestures, signs, vocalizations and use of a device) individuals with NDDs use to successfully communicate and was developed using a strengths‐based perspective (i.e. focusing on what children can do), rather than a deficit perspective. Previous qualitative work supports the content of the conceptual model and the associated measurement model, readability of the related items in the ORCA measure and relevance/importance of the communication concepts to caregivers (Reeve et al., 2023; Zigler et al., 2023). The ORCA measure was designed to include aspects of expressive, receptive and pragmatic communication, with all items contributing to one specific factor representing overall ‘communication ability’ (Zigler, Lin, et al., 2023). Scores calculated using the ORCA measure have been shown to correlate with existing caregiver‐reported measures, like the Communication and Symbolic Behaviors Scale (Reeve et al., 2023). Since the model of communication ability was developed specifically for Angelman syndrome, further research is needed to ensure the concepts included in the conceptual and measurement model are relevant to children with other NDDs.

## Methods

### Overall approach

We performed in‐depth qualitative interviews with a goal of enrolling 8–12 parents for each NDD group and 8–12 clinicians. We aimed to expand the existing conceptual framework of communication ability developed for Angelman syndrome (Zigler, Lucas, et al., 2023) and document similarities and differences within and across NDDs. Ultimately, the findings from the interviews intend to inform future modifications and additions to an existing caregiver‐reported measure of communication ability, the ORCA measure (Zigler, Lin, et al., 2023). Our qualitative methodology was aligned with practices described by Patrick et al. for eliciting relevant content for measure development (Patrick et al., 2011a, 2011b), the formative approaches detailed by Willis in his foundational texts (Willis, 2004, 2015) and the recently (U.S. Department of Health and Human Services et al., 2022) released Patient‐Focused Drug Development Guidance.

### Lived‐experience partners and collaborative engagement

For this study, COMBINEDBrain, a consortium of patient advocacy foundations, organized a formal Patient Advocates and Community Partners (PACP) group. The PACP consisted of at least one caregiver of a child that represented each of the NDDs included in this study. The PACP met every 2 weeks at regular meetings with Core Research Team representatives and provided detailed and essential feedback on study design, recruitment, enrolment and findings, as well as important background and context around the lived experiences of individuals with NDDs. These individuals were critical members of the study team and are authors on this manuscript. Study start‐up procedures were completed using regular touchpoints with representatives for each group (Table 2) to ensure the protocols were aligned with family needs. Additionally, this study brought together a multidisciplinary team of experts and other lived experience partners in paediatric clinical trials for NDDs. The External Technical Advisory Committee (ETAC) included expertise in patient advocacy, communication, community engagement, assistive devices, child development, psychometrics, neurology, paediatric trials, industry and regulatory affairs.

**Table 2:** Start‐up procedures for each neurodevelopmental disorder group

Step	Description
1.	Overall kick‐off meeting with COMBINEDBrain and Patient Advocates and Community Partners (PACP).
2.	One‐on‐one meeting with PACP representatives from each NDD in the cohort to discuss confirmation of diagnosis, resources and stratification considerations for recruitment, and recruitment process and procedures.
3.	Development of NDD‐specific recruitment materials and study documents by study team.
4.	Review of study documents by PACP representatives (e.g. recruitment flyers, demographic form, screening form, NDD‐specific protocol).
4.	Recorded presentations by PACP representatives to the qualitative study team. Typical content included an overall introduction to the NDD, caregiver experiences with caring for individuals with the NDD, what was known about communication ability, videos of individuals communicating and review of current clinical trials (e.g. in progress, in development, planned). This meeting offered opportunities for the qualitative team to engage with the patient advocates and ask questions prior to conducting caregiver interviews.
5.	Regulatory submission and approval from the Institutional Review Board.
6.	Review and feedback from the U.S. Food & Drug Administration team assigned to this project.

NDD, neurodevelopmental disorder.

### Populations of interest

Twelve NDDs were selected for inclusion in this project based on interest from the communities via engagement with the COMBINEDBrain consortium (Table 1). Representatives from each NDD community confirmed that communication ability was a priority outcome for clinical trials.

### Procedures

IRB approval was obtained by the Duke Institutional Review Board (PRO00108588), and consent was obtained from all participants in advance of data collection. The qualitative interviews occurred in a sequential fashion via four cohorts, each consisting of three NDDs, with clinician enrolment occurring simultaneously. Broad eligibility criteria for caregivers included: age ≥ 18 years, parent or caregiver of a child aged 1–18 years who was diagnosed with one of the included NDDs confirmed by a genetic or other laboratory testing report, and the ability to read, speak and understand English. Eligible caregivers also had to live with the child at least part‐time (defined as 3 days per week). For most NDDs, enrolment was stratified by age range of the child using three age groupings: 1–4 years old, 5–8 years old and 9–18 years old, to ensure representation across developmental stages. Detailed NDD‐specific procedures and considerations developed in collaboration with the PACP can be found in the Appendix S1. Purposive sampling was performed to ensure representation across other demographic characteristics, including race and ethnicity. Caregivers were recruited through postings via each patient advocacy group's community, including natural history studies, patient registries, Facebook pages, websites or email lists. Community representatives were also able to share screened patient lists for targeted recruitment.

For clinical experts, eligibility criteria included having an advanced graduate degree in speech‐language pathology (or related field) and/or training and expertise in AAC and at least 2 years of experience working with children diagnosed with at least one of the included NDDs. Educators were also recruited who had a bachelor's degree or higher with at least 5 years of experience in the special education setting and experience working with at least one child diagnosed with at least one of the included NDDs. All clinicians/educators were required to have access to a telephone or online meeting platform for in‐depth interviews. The names of potentially eligible clinical experts were shared with the study team by PACP and ETAC members. A member of the study team then reached out to gauge interest and availability.

### Concept of interest

The focus of the interviews was to identify and record examples of communication behaviours from the perspective of caregivers and clinicians. Communication ability was defined as functional (i.e. successful), interpretable and inclusive of both symbolic and non‐symbolic communication.

Symbolic communication was defined as ‘the ability to represent concepts…and use symbols for the purpose of communicating with others’ (Goodwyn, Acredolo, & Brown, 2000). Symbolic communication includes the use of verbal speech, word approximations, specific gestures (i.e. waving ‘hi’ as a greeting), American Sign Language (ASL), adapted signs and symbols/buttons/pictures on high tech or low tech AAC devices. An example of symbolic communication could be a child signing ‘more’ to indicate they would like more of their snack.

Non‐symbolic communication was defined as a direct response to a stimulus or a behaviour that has another goal, other than communication, for example, reaching to grab something. This type of behaviour can be communicative but requires a higher level of interpretation on behalf of the communication partner. It can also be intentional or non‐intentional. Examples of non‐symbolic communication include non‐specific sounds, crying, cooing, reaching, bringing something to a caregiver, non‐specific eye gaze, facial expressions and hand waving (Siegel‐Causey & Guess, 1989). For ambulatory children, an example of non‐symbolic communication could be grabbing the hand of their caregiver and bringing them over to a desired object.

While symbolic language, by definition, is always intentional, the distinction between symbolic versus non‐symbolic and intentional versus non‐intentional communicative behaviour is not always clear. It is important to note that caregivers are often instructed by speech‐language pathologists to assume intentionality in all potentially communicative behaviour (Sigafoos et al., 2000). This response can, in turn, support the development of communication skills and result in the child using the behaviour intentionally in the future (Johnson, Baumgart, Helmsetter, & Curry, 1996).

### Interview procedures

The content of the interview guide was based on prior qualitative work with caregivers of individuals with Angelman syndrome (Zigler, Lucas, et al., 2023) and Rett syndrome (Reeve et al., 2023) and modified for the aims of this project iteratively by the study team. The main portion of the interview focused on concept elicitation methods (Patrick et al., 2011a) featuring open‐ended questions about perceptions of communication ability. Caregivers were asked to describe how their child typically communicates (e.g. examples of expressive, receptive and pragmatic communication), the different modes of communication their child uses (e.g. gestures, vocalizations), what a meaningful change in communication would look like for their child and how their child's communication has changed over time. Clinicians were asked to describe the communication behaviours they observe in children with NDDs, factors affecting a child's communication and changes over time. The second portion of all interviews focused on cognitive debriefing methodology (Patrick et al., 2011b; Willis, 2004, 2015) using the ORCA measure (Reeve et al., 2023; Zigler, Lin, et al., 2023) (data reported elsewhere).

To maximize geographically dispersed participants, interviews were conducted virtually via meeting software. Interviews lasted on average from 60 to 90 min and were recorded with participant approval. Audio recordings were subsequently transcribed. Participants received remuneration of $40 after completion of an interview.

Interviewers took detailed field notes during the interviews and completed a semi‐structured debriefing form afterwards. Within the debriefing form, the interviewer summarized the key communication concepts and behaviours discussed by participants as well as their own thoughts on the content discussed. Throughout the interview process, analysts reviewed debriefing forms to identify patterns and thematic areas in the data.

### Analysis

We utilized both content‐based and thematic analysis methods to examine the qualitative interview data (Vaismoradi, Turunen, & Bondas, 2013). Content‐based coding involved assigning codes (i.e. labels) to quotes from participants with shared meaning. For example, different examples of children making requests for food or toys were coded with the ‘requesting’ code. Thematic analysis was an additional layer of review, allowing the study team to identify potential patterns within the data. For example, exploring patterns across NDD diagnosis.

Our analysis also followed the framework provided by Nowell, Norris, White, and Moules (2017) to build the trustworthiness of findings. The content‐ and thematic‐based codebook was developed iteratively, and analysts were trained on the codebook. Using NVIVO Software, three transcripts were coded by analysts to establish inter‐rater reliability for content coding. When the agreement in assigning codes was high (kappa >.9), the remaining transcripts were individually assigned to each analyst. A Standard Operating Procedure (SOP) document was created to support the consistency of coding among the analysts.

As new codes were identified or the definitions of codes needed to be updated during the coding process, the analysts held discussions at existing team meetings. When updates were confirmed, the lead analyst recorded the day of the change for audit trail purposes. After transcripts were coded, code reports were exported and assigned to individual analysts. Analysts reviewed code reports and summarized main findings in summary reports. Analysts paid particular attention to themes within and across NDDs, as well as across clinicians. The content of the interviews and counts of how many participants discussed each skill/concept were compared to the existing content of the ORCA measure (Zigler, Lin, et al., 2023). Novel themes were also highlighted.

## Results

### Caregivers

One‐hundred‐fifteen caregivers at an average age of 40.2 years participated in the qualitative interviews. A majority of the caregivers were female, married and working full‐ or part‐time (Table 3). For children, 40% of parents reported a comorbid diagnosis of epilepsy and 40% a diagnosis of autism spectrum disorder (ASD, Table 3). Approximately 42% of the children used AAC device(s) to communicate (high and/or low tech), which was introduced to the child when they were on average 4.5 years old (*SD* = 2.5 years). A more detailed breakdown of demographic information by the child's diagnosis can be found in Appendix S2.

**Table 3:** Demographic information for caregivers who participated in the qualitative interview study and their children (*N* = 115)

	*n* (%)
Gender, female	97 (84.3)
Ethnicity, Hispanic‐Latino	20 (17.4)
Race	
White	96 (83.5)
African American or Black	6 (5.2)
American Indian/Alaska Native	1 (0.9)
Asian	7 (6.9)
Native Hawaiian/Other Pacific Islander	1 (0.9)
More than one race	4 (3.5)^a^
Relationship status	
Married, or living with domestic partner	100 (87.0)
Single, never married	9 (7.8)
Divorced	6 (5.2)
Highest grade in school	
Less than high school diploma	1 (0.9)
High school degree or equivalent	13 (11.3)
Some college/University	25 (21.7)
College/University degree	32 (27.8)
Postgraduate degree	44 (38.3)
Occupational status	
Full‐time employed	69 (60.0)
Homemaker	17 (14.8)
Unemployed	4 (3.5)
Part‐time employed	25 (21.7)
Household income (annual)	
Less than $20,000	3 (2.6)
Between $20,001 and $40,000	9 (7.8)
Between $40,001 and $60,000	12 (10.4)
Between $60,001 and $80,000	16 (13.9)
Between $80,001 and $100,000	17 (14.8)
Between $100,001 and $250,000	42 (36.5)
Between $250,001 and $500,000	10 (8.7)
$500,000+	3 (2.6)
I prefer not to answer	3 (2.6)
Age, years (mean, *SD*)	40.2 (6.74)
Neurodevelopmental disorder of child	
Bosch–Boonstra–Schaaf optic atrophy syndrome (BBSOAS)	11 (9.6)
GRIN2B‐related neurodevelopmental disorder	8 (7.0)
Hao‐Fountain Syndrome (HAOFOUS)	9 (7.8)
HNRNPH2‐related disorders	7 (6.0)
Hunter syndrome (MPS II)	10 (8.7)
Malan syndrome	11 (9.6)
Phelan McDermid syndrome (PMS)	10 (8.7)
Schinzel–Giedion syndrome (SGS)	4 (3.5)
SCN2A‐related disorders (SRD)	12 (10.4)
SETBP1 haploinsufficiency disorder (SETBP1‐HD)	9 (7.8)
STXBP1‐related disorders	12 (10.4)
SYNGAP1‐related intellectual disability	12 (10.4)
Gender, female	64 (55.7)
Ethnicity, Hispanic‐Latino	25 (21.7)
Race	
White	89 (77.4)
African American or Black	7 (6.1)
American Indian/Alaska Native	1 (0.9)
Asian	7 (6.1)
Native Hawaiian/Other Pacific Islander	2 (1.7)
More than 2 races	9 (7.8)
Autism Spectrum Disorder Diagnosis	47 (40.9)
Epilepsy diagnosis	47 (40.9)
Types of therapy	
Physical therapy	73 (63.5)
Occupational therapy	88 (76.5)
Speech therapy	95 (82.6)
Other	63 (54.8)
AAC device	48 (41.7)
Age first introduced to device, years (mean, *SD*)	4.52 (2.52)
Device type	
High Tech	41 (35.7)
Low Tech	9 (7.8)
Places device used	
Home	32 (27.8)
School	41 (35.7)
Out in the community	8 (7.0)
Other—therapy	6 (5.2)
Age, years (mean, *SD*)	8.01 (4.21)

^a^ White and Middle Eastern, White and African‐American or Black, White and African‐American or Black, and White, American Indian/Alaska Native and Asian.

### Typical communication

Caregivers were able to provide detailed descriptions of their child's typical communication ability in the areas of expressive, receptive and pragmatic communication. For example, one caregiver (PI:1101, GRIN2B) described their child using a modified gesture/sign in an expressive way: ‘Well recently and this is a new thing, it's happened in the last month, I always sign the word ‘eat’ to him, and recently when he's getting hungry, he'll put his hand up to his mouth, like the eat motion, and then he'll kick his right leg to let us know that he's hungry’. Another caregiver (PI:1007, BBSOAS) gave an example of how they know their child understands their communication, saying, ‘She'll either follow through with what you ask of her or she'll respond in an appropriate manner. So, if you tell her that you love her, she'll give you a hug, or she'll smile, or something like that’. In terms of social communication, one caregiver (PI:701) spoke about their child giving hugs/kisses, saying, ‘She'll put her hand around your neck and then pull you close to kiss you or hug you or nuzzle you’.

At the content‐level, there was an overlap between the concepts reported by caregivers in the interviews and the existing conceptual model (Table 4). Commonly mentioned concepts included requesting an object, use of some words/verbal speech, refusing an object, responding to familiar directions, use of AAC devices and seeking attention. A detailed description of communication abilities by child‐NDD and concept can be found in Appendix S2.

**Table 4:** Frequencies of concepts discussed within the caregiver interviews by neurodevelopmental disorder (concepts currently on the ORCA measure)

	SYNGAP1 (*n =* 12)	STXBP1 (*n =* 12)	MPS II (*n =* 10)	PMS (*n =* 10)	Malan (*n =* 11)	SCN2A (*n =* 12)	SETBP1 (*n =* 9)	SGS (*n =* 4)	HNRNPH2 (*n =* 7)	BBSOAS (*n =* 11)	GRIN2B (*n =* 8)	HAFOUS (*n =* 9)
*Expressive communication*												
Seeking attention	5	2	6	4	5	7	4	0	3	2	0	4
Directing attention	1	0	2	5	8	3	4	0	7	1	1	4
Refusing an object	3	3	9	4	5	4	5	4^a^	5	7	4	6
Requesting an object	12	12	10	9	11	11	9	0	7	10	6	8
Requesting an object out of view	4	5	4	2	4	1	1	0	1	2	0	1
Requesting more of something	1	6	3	3	5	2	2	0	1	2	3	1
Asking questions	0	0	2	1	3	0	2	0	2	2	0	3
Communicating understanding	0	0	0	0	0	0	0	0	0	0	1	0
Communicating with people other than the caregiver	0	0	0	0	1	0	0	0	0	0	1	0
Telling stories	1	1	2	1	2	0	3	0	1	3	1	2
*Receptive communication*												
Taking turns in conversation	0	0	0	1	1	1	1	0	0	0	2	1
Making choices	1	6	4	4	5	4	1	0	0	4	4	3
Responding to name	0	1	1	0	0	3	1	0	0	0	1	0
Responding to familiar directions	3	3	2	5	10	2	6	0	5	4	4	7
Responding to new directions	0	0	0	0	0	0	3	0	0	0	1	1
Understanding mood	0	0	0	0	1	0	1	0	0	0	2	2
Understanding isolated words or phrases	4	1	4	0	0	3	1	1	1	1	1	1
Responding to Questions	0	1	5	0	5	2	4	0	2	1	3	3
*Pragmatic communication*												
Greeting	1	3	1	2	0	1	7	0	5	4	2	1
Using names	2	5	8	0	7	2	5	0	3	2	3	3
Playing games/Activities	1	1	1	2	1	2	1	0	1	2	1	2
Comforting others	0	0	0	0	1	0	1	0	0	0	2	2
*Communication modality*												
Uses AAC device	9	5	5	6	3	6	3	0	5	3	3	0
Uses some words	6	5	7	4	10	3	8	0	4	7	4	7

BBSOAS, Bosch–Boonstra–Schaaf optic atrophy syndrome; GRIN2B, GRIN2B‐related neurodevelopmental disorder; HAFOUS, Hao‐Fountain syndrome; HNRNPH2, HNRNPH2‐related disorders; Malan, Malan syndrome; MPS II, Hunter syndrome; PMS, Phelan McDermid syndrome; SCN2A, SCN2A‐related disorders; SETBP1, SETBP1 haploinsufficiency disorder; SGS, Schinzel–Giedion syndrome; STXBP1, STXBP1‐related disorders; SYNGAP1, SYNGAP1‐related intellectual disability.

^a^ All four caregivers of children with SGS spoke about how their children refused using physical gestures (e.g. looking away, turning head away), with the majority of examples related to *activities*. Please note, the refusal concept on the ORCA measure specifically refers to refusing *objects*.

**Figure 1:**
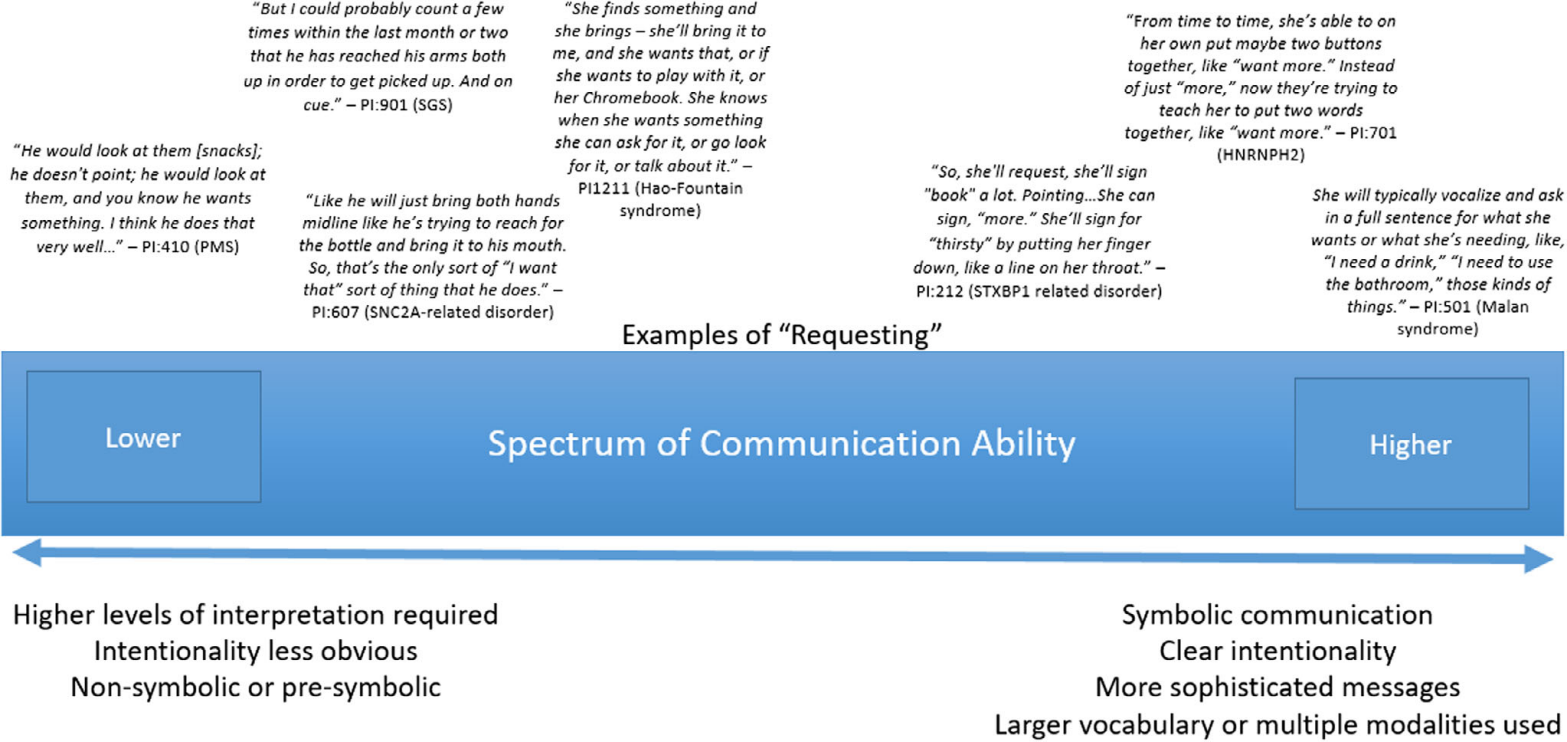
Heterogeneity in communication behaviours for one concept (requesting) within and across neurodevelopmental disorders (NDD)

There was notable heterogeneity within and across NDD groups in terms of the specific communication behaviours described within each communication concept, including the different ways children communicate such as verbal speech, gestures, ASL, eye gaze, body movements and use of AAC. Requesting was the most discussed communication concept, with almost every caregiver conversing about it during concept elicitation. As such, it can be used as an illustrative example of heterogeneity within a concept (Figure 1). For example, there were parental observations of the child's behaviour that required interpretation, ‘…if he's hungry, he kind of just gravitates towards our snack cupboard or our refrigerator. There is no communication in regards to him showing you a picture or him telling you what he needs or wants’ (PI:310, Hunter syndrome). Other caregivers described concrete use of symbolic language, ‘Then, she will use [her AAC device to say]… ‘I want to play,’…Then, there are choices with different toys and activities that she can choose from’. (PI:210, STXBP1‐related disorder).

### Novel communication concepts

**Figure 2:**
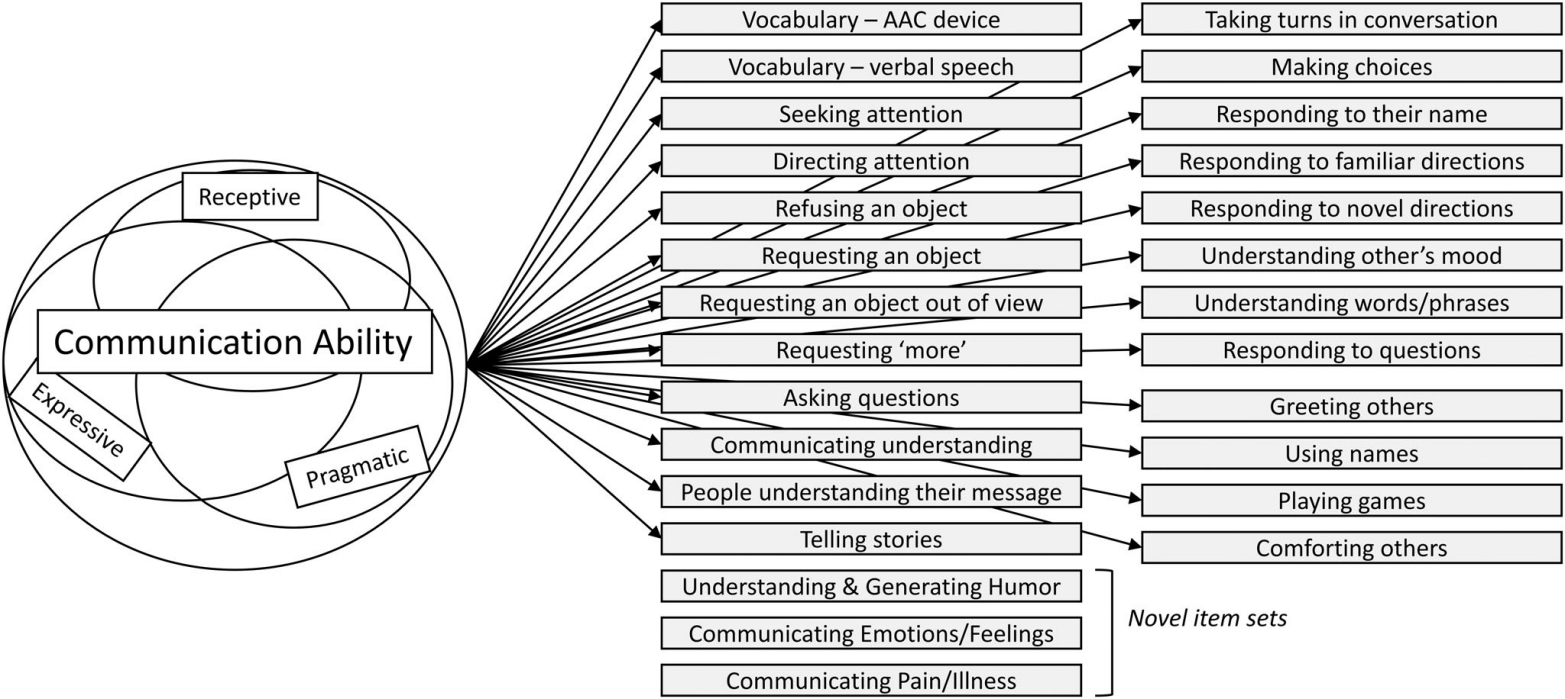
Existing and updated conceptual model of meaningful aspects of communication ability for children with rare neurodevelopmental disorders

Novel communication concepts identified within interviews but not currently within the conceptual model included children communicating about (1) feelings, emotions and bodily sensations, (2) commenting on likes and dislikes and (3) communicating and understanding humour (Table 5). The novel concepts are described in the following sections and added to an updated conceptual model (Figure 2). A full list of quotes can be found in Appendix S2.

**Table 5:** Novel communication concepts identified via caregiver interviews

Novel Concepts	SYNGAP1 (*n =* 12)	STXBP1 (*n =* 12)	MPS II (*n =* 10)	PMS (*n =* 10)	Malan (*n =* 11)	SCN2A (*n =* 12)	SETBP1 (*n =* 9)	SGS (*n =* 4)	HNRNPH2 (*n =* 7)	BBSOAS (*n =* 11)	GRIN2B (*n =* 8)	HAFOUS (*n =* 9)
Humour	0	2	3	2	2	2	1	0	0	2	1	2
Expressing feelings, emotions and bodily sensations using symbolic communication	4	2	1	0	3	1	1	4^a^	4	2	2	2
Commenting: expressing opinions or reactions	4	4	7	3	5	8	3	2	3	1	4	3
Self‐advocating	0	0	0	0	2	0	0	0	0	0	0	0
Responding to familiar voices/people	0	0	0	1	0	0	0	2	0	0	1	0
Responding to familiar routines	0	0	0	0	0	0	0	3	0	0	0	0
Social smile, makes eye contact, face ‘brightens’	0	2	0	2	0	4	0	3	0	2	1	0

^a^ All caregivers of children with SGS spoke about ways their children communicated comfort/discomfort and happiness/unhappiness, but unlike other interviews counted within this category, none of the four children with SGS used symbolic communication.

BBSOAS, Bosch–Boonstra–Schaaf optic atrophy syndrome; GRIN2B, GRIN2B‐related neurodevelopmental disorder; HAFOUS, Hao‐Fountain syndrome; HNRNPH2, HNRNPH2‐related disorders; Hunter, Hunter syndrome; Malan, Malan syndrome; PMS, Phelan McDermid syndrome; SCN2A, SCN2A‐related disorders; SETBP1, SETBP1 haploinsufficiency disorder; SGS, Schinzel–Giedion syndrome; STXBP1, STXBP1‐related disorders; SYNGAP1, SYNGAP1‐related intellectual disability.

#### Feelings, emotions, internal bodily sensations

A number of caregivers said their child could communicate, or has attempted to communicate, their emotions or how they are feeling (e.g. when sick, experiencing a stomach ache, etc.) using symbolic communication. One caregiver of a child with GRIN2B‐related neurodevelopmental disorder (PI:1106) said, ‘If she's tired, she'll say, “night‐night.” Letting us know what she needs, like if she needs to rest or if something is hurting, she'll say “hurt”’. Other caregivers described behaviours that were interpreted by the caregivers as their child communicating their feelings/emotions, and potentially, sickness/pain. However, this communication was not as clear as the examples provided above. For example, one caregiver (PI:210, STXBP1‐related disorder) said, ‘If she's in any kind of pain, she will kind of whimper or cry out her discomfort and let us know that’.

#### Commenting

Commenting is considered an aspect of expressive communication and commonly defined as verbal or written remarks expressing opinions or reactions (Oxford Languages, 2023). Fifty caregivers discussed how their children comment. The examples that they provided focused generally on children indicating their likes and dislikes. Although commenting was commonly mentioned across the interviews, examples were highly variable across families in terms of positive/negative comments, the content of the comments and the amount of interpretation required by the caregivers in understanding. Some of these comments intersected with expressing feelings, emotions and bodily sensations, underscoring the multifaceted nature of children's communication.

#### Humor: comprehension and use

A number of caregivers discussed humour as a concept that was relevant and important to their family, with a number of those having a teenage child (13–17 years old). Discussion around humour included the child's understanding of humour (e.g. laughing at things that should be funny, like a T.V. show), as well as the child's use of humour (e.g. the child saying something silly to prompt a laugh or initiating a joke). Caregivers also spoke about how meaningful these interactions were, or how they contributed to their child's social connections.

### Findings across NDDs

Regardless of diagnosis, caregivers whose children did not use symbolic communication regularly or at all described communication behaviours that still allowed them to understand and engage with their children. Caregivers mentioned their children making simple requests, responding to familiar voices or people, and responding to familiar routines. One caregiver (PI:903, SGS) said, ‘You can definitely tell when he hears someone that he knows well, like me, his mom, or his dad. I think the grandparents too, he really recognizes them, and his face almost brightens up some when he's awake. Sometimes, he'll smile’. For these children, caregivers were required to interpret non‐specific communication behaviours on a regular basis, and some reported that it took a lot of time or trial and error to understand what their child meant or what they needed.

Additionally, a number of caregivers reported that their child can get frustrated when (a) they are not being understood by a communication partner; (b) when they are not able to express themselves successfully; (c) or when they are being asked to do something they do not want to do. Caregivers said this frustration often leads to behaviour issues. One caregiver (PI:108, SYNGAP‐1‐related intellectual disability) said, ‘There are instances where…people do not comprehend what she wants. And then, that can get her frustrated, and that can trigger the behavior, and that can affect everything else within her day’. Additionally, caregivers discussed how their child communicates differently when they are sick, not feeling well or experiencing certain medical issues (e.g. seizures, gastrointestinal issues). One caregiver (PI:203, STXBP1‐related disorders) said, ‘We have had periods where he wasn't doing well…But it was because something was going on with him. It was either an illness or it was a reaction to a medication. During those times, he lost everything. He wasn't hardly interacting with us at all’.

### Changes in communication

In terms of changes over time, caregivers talked about the speed of skill acquisition. Some caregivers mentioned that skills are variable and inconsistent, and other skills may disappear when their child is trying to learn a new skill. For example, one caregiver (PI:309, Hunter syndrome) said, ‘He kind of has thing in general with all of his skills where he'll start to do something new. And then, for a little while he'll do it. And then, he'll kind of stop doing it. But he'll start to do something else new, and then later, maybe a few months later, he'll bring back the old skill too. So, it's kind of like, if he's learning something new, he can't focus on a bunch of new things at once. So, it's almost like his brain's like okay, we did that. Put that on pause. We're gonna focus on something new now. And then a little bit later, we got that. Now, we can bring it all back to use it consistently’. To some caregivers, changes in their child's communication felt fast and to others, it felt slow. One caregiver (PI:807, SETBP1‐HD) said, ‘And the changes are very slow…during one year, we see very few improvements, a little improvement’.

### Meaningful changes

During the interviews, caregivers were asked, ‘What would a meaningful change in communication look like for your child?’ In general, parents interpreted this question to be asking about improvements in communication ability or skills. Parents had a wide variety of skills and situations that they would consider meaningful for themselves and their children. For example, a caregiver of a child with Malan syndrome (PI:501) stated, ‘I think a meaningful change in her communication would be flipping communication to the other person. You know, ‘How was your day?’ So, her communication is currently all about herself…And, you know, I think communication too is a two‐way. So, maybe listening and responding’. Another caregiver (PI:801, SETBP1‐HD) mentioned skills that would help the child be better understood by others would be meaningful: ‘Definitely more, the clarity of pronunciation…there are some [sounds]…For room, she would say loom’.

One caregiver (PI:704, HNRNPH2‐related disorders) spoke about her experience supporting her daughter's communication, saying, ‘I think that would be the most meaningful part, is that when she did communicate, I could tell her once or twice. It would stick, she could use it in a sentence, and we could move on, instead of being hung up on something, and it taking days to understand…Probably because as her caregiver, it's tiring and taxing, and I have to be on my A game. I had to go back to college and take two years of American Sign Language just to be able to adequately teach her, and then understand. So, it takes a huge requirement, and basically life change on top of that to be able to teach your child a way to communicate that they can physically do and understand’.

In another example, a caregiver (PI:608, SCN2A‐related disorder) discussed how if the child had the ability to express that she was in pain, it would improve the child's overall quality of life, saying, ‘Anything progressing towards being able to do that [communicate pain] more successfully would be huge for our family – particularly with when she's not feeling good and something's wrong’.

If time permitted, caregivers were also asked, ‘If your child's communication skills were to decline or get worse, which communication skills would be most important for your child to retain?’ Responses to this question typically included specific modalities (e.g. eye gaze, device usage and gestures). Parents also mentioned general concepts like expressing needs and skills for independent living. When responding to this question, many caregivers also pointed out their child's level of skill. For example, one caregiver (PI:408, PMS) stated, ‘But right now, the communication is just so little that I don't…there's not much for her to lose’. A more detailed description of caregiver responses to the meaningful change question and the skill‐retention question can be found organized by NDD in Appendix S2.

### Clinicians

Nine clinical experts participated (4 MS degrees, 2 doctoral degrees and 3 MDs). Five indicated expertise in AAC, and seven had experience with clinical trials. The mean age was 47.4 years (range: 33–75 years), and five were women (a full description of the sample can be found in Appendix S2; Table S88). In the context of NDDs, most experts (*n =* 6) described ‘advanced communicators’ as those that use verbal speech. Two experts said high‐level communicators could also be those who are using an AAC device very sophisticatedly (e.g. combining multiple symbols together). Three experts noted some individuals with NDDs may have typical language for their age, although they may struggle with their motor speech function or social communication. For example, one expert (PI:004) said, ‘…so I see a lot of, you know, from the very minimally verbal/nonverbal/zero communication, very aloof children who don't have any gesture language, don't really have any eye contact, don't really have any babbling. You know, there's a very profound receptive expressive language delay all the way through to, you know, individuals who are 12, 13, 14 that I'm diagnosing for the first time with autism based on a wealth of more social pragmatic impairments, but they have very clear functional communication. I mean, they use, let's say, the expected amount of words’. Experts described lower level communicators as those with minimal or no verbal speech that may only use eye contact, facial expressions, sounds or physiological/unintentional reflexive behaviours to communicate. For example, one expert (PI:003) said, ‘When I work with…children with the most extreme communication problems, many of their communication is interpreted, so I also include interpretation – and that's based on physiological reactions – they're hot because they're sweating, they cry in a certain way when they're in pain versus they're hungry or they're sick, and…caregivers are able to differentiate those kinds of signals’.

Clinical experts also discussed how they assess communication ability, the health impacts on communication ability (and vice versa), changes over time in skills and caregivers as reporters on their child's communication outside of clinic. Additional details and supportive quotes can be found in Appendix S2.

## Discussion

This project is one of the first large‐scale qualitative studies exploring communication ability across a broad range of children with NDDs while centring the caregiver perspective. Virtually all caregivers discussed aspects of expressive communication during the interviews, with most caregivers also mentioning receptive and pragmatic communication. Overall, the concepts elicited by parents and the communication behaviours they described aligned well with the current concepts on the ORCA measure (Table 4). All concepts across the domains of expressive, receptive and pragmatic communication were discussed in at least one interview, with some concepts being mentioned more frequently. The topics that caregivers discussed across all NDDs also aligned with our current understanding of communication ability and development (Goodwyn et al., 2000; Johnson et al., 1996; Siegel‐Causey & Guess, 1989; Sigafoos et al., 2000). This observed consistency in the content indicates trans‐diagnostic relevance of the existing communication model.

Requesting, one of the most frequently discussed concepts, allows the child to ‘express their wants and needs to significant others in their proximity’. (Lancioni, Singh, O'Reilly, & Alberti, 2019). As expected based on previous work in Angelman syndrome (Zigler, Lucas, et al., 2023) and Rett syndrome (Reeve et al., 2023), the modality that children used to make requests and the sophistication of the communication behaviour varied across the sample (Figure 1). The description of different levels of communication within concepts supports the original conceptual model and measurement approach. Even children with severe communication challenges and those using AAC devices can be ranked along a continuum representing higher and lower communication by capturing different skill levels within relevant concepts, like requesting.

A few novel concepts were identified from the interviews that are not currently within the ORCA measure conceptual model, including communicating feelings, emotions, bodily sensations and likes/dislikes, as well as humour (Figure 2). There is merit in exploring these concepts as potential additions to the ORCA measure to improve content validity across different NDDs. For children who are not regularly exhibiting symbolic communication, the addition of items about social smiles and recognizing faces could be beneficial to expand the range of ability levels beyond that of the original development work for Angelman syndrome, who commonly show positive affect and social smiles (Margolis, Sell, Zbinden, & Brid, 2015). At the other end of the ability spectrum, the understanding and use of humour could also increase the content validity of the measure, especially for older children and teenagers who are able to use symbolic communication in this way. When drafting and testing out new items for the measure, care should be taken to ensure repetition is not being introduced in the content due to overlap with existing items. Cognitive testing could be conducted to ensure novel items are understandable, and item reduction could also be targeted in future quantitative work to ensure efficiency in the final item set. When discussing communication with caregivers, we heard about pragmatic concepts that are often considered part of separate social functioning domains on existing measures, like playing games and social relationships (Sparrow, Cicchetti, & Saulnier, 2016). This was aligned with the initial support for the measurement model (Reeve et al., 2023), which included concepts indicating social communication ability. It is also aligned with what we know from foundational work that communication is strongly related to both cognition and social development (Homer & Tamis‐LeMonda, 2013). As an additional verification of this component of the measurement model, quantitative work done on the current ORCA measure indicated that all items, including those representing expressive, receptive and pragmatic communication, loaded on a single factor representing ‘communication ability’ (Reeve et al., 2023). If novel items are added to the measure in the future, including additional items that indicate social communication, quantitative analysis should be completed to evaluate the measure's factor structure.

Demonstrated support for trans‐diagnostic content validity is an important first step in ensuring valid and reliable tools that capture communication ability for children with NDDs, many of which are rare. In addition to supporting the content included in the current ORCA measure, these results also indicate support for the content validity of other commonly used measures that ask caregivers to report on these aspects of their child's communication, like the Vineland Adaptive Behavior Scales (Sparrow et al., 2016). In terms of validity, the next step is to build upon this evidence to ensure the choice of any measure is ‘fit for purpose’. (U.S. Department of Health and Human Services et al., 2022) For example, researchers and clinicians who want to compare communication ability across NDDs/diagnoses, whether in combined clinical trials or other registries, should conduct necessary quantitative work to determine if the performance of the set of items and the underlying factor structure are invariant across NDDs to allow for the comparison of scores. For end‐users who want to support the use of a measure in one specific population, additional quantitative work is also important to provide evidence for the validity of the scores.

## Limitations

While over 30% of caregivers identified with a racial and/or ethnic group that traditionally is underrepresented in clinical research, the resulting sample of caregivers was overall highly resourced in terms of education and annual income (Table 3). We also limited our sample to English‐speaking caregivers. While our analysis is supportive of the salience of certain topics, as indicated by the number of interviews where the concept was discussed, the counts are neither fully indicative of the relevance of specific concepts to all families, nor should it be interpreted as inclusive of all skills used by individuals with NDDs. During 60–90‐min interviews, we could not discuss the entirety of a child's communication across their life. If a caregiver did not mention receptive communication, for example, this does not necessarily indicate that this type of communication is not important or that their child cannot perform this type of communication; it just meant that they discussed other topics during the interview.

Although our results were supportive of the conceptual model on which the current ORCA measure is based, more work should be done to support the validity of the measure in children and adolescents with the included NDDs before it is appropriate to use a modified version in clinical trials. As part of the larger CDER initiative, we plan to generate novel item sets and modify items, then cognitively debrief on the updated measure with caregivers. In the future, quantitative psychometric studies, including those that evaluate scoring approaches (like Farmer et al., 2023), should also be conducted, as there are risks to including untested measures in clinical trials (Keeley et al., 2024).

## Conclusions

We have conducted extensive qualitative work with caregivers of children with 12 different rare NDDs alongside clinical experts to explore observations of typical communication ability. Overall, the results from the concept elicitation interviews support the idea that communication behaviours are similar across rare disease groups. Additional concepts were also identified that could offer opportunities for novel item sets to broaden the relevance of measures aiming to quantify communication ability. While this qualitative work is formative in understanding the concept of communication ability, future work is needed to further develop patient‐centred measures that can be deemed ‘fit for purpose’ for critical NDD clinical trials.

## Ethical considerations

Informed consent has been appropriately obtained. Research protocols were reviewed and approved prior to initiation by the DUHS Institutional Review Board (original approval date 08/06/2021; Pro00108588‐INIT‐1.0).

**Key points**

**What's known?**

An expanded measurement model of communication ability for children with rare neurodevelopmental disorders (NDDs) is needed before this important concept can be measured within clinical trials.

**What's new?**

We conducted concept elicitation interviews with caregivers and purposively selected clinical experts, focusing on a broad definition of communication ability that was inclusive of expressive, receptive and pragmatic communication concepts.

**What's relevant?**

Commonly mentioned concepts across the 115 interviews included requesting an object, refusing an object, responding to familiar directions and seeking attention, but there was notable heterogeneity within and across NDD groups in terms of different ways children communicate. Future work is needed to further develop patient‐centred measures that can be deemed ‘fit for purpose’ for critical NDD clinical trials.

## Supporting information


**Appendix S1.** Methods.

**Appendix S2.** Additional results; including Tables S1–S91.

## Data Availability

The data that support the findings of this study are available from the corresponding author upon reasonable request.
