# Supplementary material for: The spectrum of communication abilities in children with 12 rare neurodevelopmental disorders: a qualitative study with caregivers
Source: J Child Psychol Psychiatry. 2025 Oct 20;67(5):740–54. doi: 10.1111/jcpp.70063 (PMC13102052; doi:10.1111/jcpp.70063)
Supplement: Supplementary file 1 — Appendix S1. Methods. [file JCPP-67-740-s001.docx]

The spectrum of communication abilities in children with twelve rare neurodevelopmental disorders: A qualitative study with caregivers

Supporting Information

**Appendix S1:** Methods

Methods Specific to Each NDD

Each neurodevelopmental disorder (NDD) included in this study had specific procedures that were developed in close contact with representatives from the Patient Advocates and Community Partners (PACP). The following sections provide an overview of each specific NDD and the procedures unique to each group that include current knowledge of communication ability, confirmation of genetic diagnosis, and specific recruitment methods.

Contents

[1. Bosch-Boonstra-Schaaf optic atrophy syndrome (BBSOAS) 2](#_Toc200457791)

[2. GRIN2B-related neurodevelopmental disorder 3](#_Toc200457792)

[3. Hao-Fountain Syndrome (HAFOUS) 4](#_Toc200457793)

[4. HNRNPH2-related disorders 5](#_Toc200457794)

[5. Hunter syndrome (Mucopolysaccharidosis Type II (MPS II)) 6](#_Toc200457795)

[6. Malan Syndrome (NFIX) 8](#_Toc200457796)

[7. Phelan McDermid Syndrome (PMS) 10](#_Toc200457797)

[8. Schinzel-Giedion Syndrome (SGS) 12](#_Toc200457798)

[9. SCN2A-related disorders 14](#_Toc200457799)

[10. SETBP1 Haploinsufficiency disorder (SETBP1-HD) 16](#_Toc200457800)

[11. STXBP1-related disorders 18](#_Toc200457801)

[12. SYNGAP1-related intellectual disability 20](#_Toc200457802)

[References 22](#_Toc200457803)

###

### Bosch-Boonstra-Schaaf optic atrophy syndrome (BBSOAS)

#### Overview

| Disorder & Incidence | BBSOAS | 1 in 100,000 to 250,000 |
| --- | --- | --- |
| Description of Disorder^1-3^ | BBSOAS is a rare neurological disorder caused by deletions or variants in the *NR2F1* gene. Common impacts include developmental delay, intellectual disability, and optic atrophy – the visual phenotype also includes cortical visual impairment and optic nerve hypoplasia. Other impacts include autism spectrum disorder, hypotonia, feeding difficulties, and seizures. | |
| Communication issues | Communication impairments are more severe in individuals with mutations caused by a missense variant or in frame deletion within the DNA-binding domain (DBD). Patients with other variants (e.g., nonsense or whole deletions) seem to develop language and higher level communication skills.^3^ | |
| Foundation (website) | NR2F1 Foundation (http://nr2f1.org) | |
| Members | 350 families | |
| Studies (new/ongoing) | Patient Registry via Matrix. | |
| PACP representative | Melissa Thelen | |

#### Communication Ability

Bosch-Boonstra-Schaaf Optic Atrophy Syndrome (BBSOAS) is caused by loss-of-function mutations in the *NR2F1* gene.^1^ Visual and cognitive deficits impact the majority of individuals,^2^ who also present with hypotonia, seizures, autism spectrum disorder (ASD), and hearing issues.^1^ In the largest published clinical cohort, one of the most common clinical features was speech delay (alongside vision impairment and hypotonia), which occurred in 91% of the sample.^3^ Forty-two percent of the sample were considered nonverbal, and patients with missense variants and in-frame deletions within the DBD were more likely to have severe impacts across multiple domains, including speech.^3^

#### Confirmation of Diagnosis

Genetic testing is the only way to confirm BBSOAS. Pathogenesis and variant/mutation type were verified by two genetic counselors at Duke University via review of the child’s genetic report submitted by the caregiver.

#### Recruitment Processes

The [NR2F1 Foundation](https://nr2f1.org/) is a charitable foundation established by parents of children with BBSOAS. Their mission is to fund research, connect families, and raise awareness about the condition. We purposely sampled English-speaking participants in close partnership with the foundation to ensure recruitment of individuals with confirmed diagnoses.

#### Representation across the Communication Ability Spectrum

As described in the study protocol, caregiver enrollment was stratified by child age (1-4, 5-8, and 9-<18 years old). We planned to recruit an even (or close to even) distribution of caregivers with female and male children. As with other cohorts, we reported if the child had seizures or an ASD diagnosis. We also asked caregivers about vision issues that may impact communication, as these are a core component of BBSOAS.^1^ Hearing issues also occur in a subset of individuals.^1^

### GRIN2B-related neurodevelopmental disorder

#### Overview

| Disorder & Incidence |  | 6 in 100,000 |
| --- | --- | --- |
| Description of Disorder^4^ | GRIN2B-Related Neurodevelopmental Disorder is caused by variations, deletions or duplications on the GRIN2B gene. This neurological condition is often characterized by low muscle tone, developmental delays, seizures and lack of speech. The spectrum of abilities is broad depending on the genetic variation. Some children cannot walk or talk, and most struggle to communicate basic daily needs. Approximately 30% of patients have some form of seizures. | |
| Communication issues | Children present with varying degrees of speech impairments, ranging from completely nonverbal to some children that can speak almost to age level.^5^ | |
| Foundation (website) | GRIN2B Foundation (www.grin2b.org) | |
| Members | 650+ families | |
| Studies (new/ongoing) | Natural history study with University of Colorado and a registry with Simon's Searchlight. | |
| PACP Representative | Liz Marfia-Ash | |

#### Communication Ability

GRIN2B-Related Neurodevelopmental Disorder is a rare condition caused by variants within the *GRIN2B* gene.^4^ To our knowledge, a full description of the spectrum of communication abilities for individuals with GRIN2B-related neurodevelopmental disorder has not been published in the literature. A clinical study describing five individuals was published in 2013 and described varying degrees of speech delay.^5^ Based on initial conversations with advocates from the community, we expected that we may see communication differences between those with deletion/duplication variants (less severely affected) vs. those with missense variants (more severely affected).

#### Confirmation of Diagnosis

Genetic testing is the only way to confirm GRIN2B-related neurodevelopmental disorder. Variants needed to be verified as pathogenic or likely pathogenic to be included in this study.

#### Recruitment Processes

The [GRIN2B Foundation](http://grin2b.com/) is a charitable foundation established by parents of children with GRIN2B-related neurodevelopmental disorder. Their mission is to fund research, connect families, and raise awareness about the condition. We recruited primarily via the Foundation’s registry, as caregivers must upload genetic reports to confirm diagnosis.

#### Representation across the Communication Ability Spectrum

As with other groups, caregiver enrollment was stratified by child age (1-4, 5-8, and 9-<18 years old). We planned to recruit an even (or close to even) distribution of caregivers with female and male children. As with other cohorts, we reported if the child had seizures or an ASD diagnosis. We also asked caregivers about vision and hearing issues that may impact communication.

### Hao-Fountain Syndrome (HAFOUS)

#### Overview

| Disorder & Incidence |  | Unknown |
| --- | --- | --- |
| Description of Disorder^6-8^ | Individuals who are born with a mutation in the USP7 protein-coding gene have a neurodevelopmental disorder called Hao-Fountain Syndrome (HAFOUS). Its symptoms include autism, epilepsy, speech delays, intellectual disability, and GI issues. | |
| Communication issues | There is a wide range of communication abilities in HAFOUS. Many individuals are nonverbal with mixed receptive-expressive language disorder, others with apraxia of speech, and others have typical communication. | |
| Foundation (website) | Foundation for USP7-Related Diseases (www.usp7.org) | |
| Members | 142 known cases worldwide, 40 total in US | |
| Studies (new/ongoing) | Preparing for phenotyping study. | |
| PACP Representative | Bo Bigelow | |

#### Communication Ability

Hao-Fountain syndrome (HAFOUS) is a neurodevelopmental disorder caused by mutations on the *USP7* gene.^6-8^ Recently named, HAFOUS full phenotypic spectrum is “still emerging”.^8^ To our knowledge, a full description of the spectrum of communication abilities for individuals with HAFOUS has not been published in the literature. In the largest cohort to-date, all individuals with data had “prominent speech delays” and four (18%) were considered nonverbal.^7^

#### Confirmation of Diagnosis

Genetic testing is the only way to confirm HAFOUS. Variants needed to be verified as pathogenic or likely pathogenic to be included in this study.

#### Recruitment Processes

The [Foundation for USP7-Related Diseases](https://www.usp7.org/) is a charitable foundation established by parents of children with Hao-Fountain syndrome. Their mission is to fund research, connect families, and raise awareness about the condition. We recruited primarily via the Foundation’s closed Facebook group. As we recruited from the community setting, we were not able to ensure molecular diagnosis confirmation in advance. However, we included on all advertisements that this was a requirement for participation. Variant/mutation type were verified by two genetic counselors at Duke University via review of the child’s genetic report submitted by the caregiver.

#### Representation across the Communication Ability Spectrum

As with other groups, caregiver enrollment was stratified by child age (1-4, 5-8, and 9-<18 years old). We planned to recruit an even (or close to even) distribution of caregivers with female and male children. As with other cohorts, we reported if the child had seizures or an ASD or ID diagnosis. We also asked caregivers about vision and hearing issues that may affect communication.

### HNRNPH2-related disorders

#### Overview

| Disorder & Incidence |  | 1.55 in 100,000 |
| --- | --- | --- |
| Description of Disorder | Similar to Rett Syndrome and Angelman syndrome. HNRNPH2-disorder is characterized by significant cognitive impairments and adaptive behavior. Patients manifest generalized developmental delay, autism, hypotonia, seizures, and motor and communication impairments.^9,10^ Although HNRNPH2-disorder can occur in males, it primarily affects females.^11,12^ | |
| Communication issues | Communication abilities vary. Most children are nonverbal or minimally verbal. Some children use AAC devices, eye gaze or American Sign Language. | |
| Foundation (website) | Yellow Brick Road Project (https://yellowbrickroadproject.org/) | |
| Members | ~140 families, with about 60 in the U.S. | |
| Studies (new/ongoing) | Natural history study ongoing. Foundational studies at St. Jude Children's Hospital. | |
| PACP Member | Trish Flanagan & Stacy Paddon | |

#### Communication Ability

HNRNPH2-related disorder is caused by variants in the X-linked HNRNPH2 gene. It is a relatively newly identified disorder, and the literature base is still being established.^9^ In 2021, authors published a manuscript that explored the clinical characterization of HNRNPH2-related disorders in 33 individuals.^10^ The authors reported 76% of those individuals as being nonverbal or “minimally verbal”, with speech acquisition between 1-5 years of age (for those that were verbal). In another study, authors found a positive correlation between motor impairments and non-verbal status in females with HNRNPH2-related disorder.^13^

#### Confirmation of Diagnosis

Genetic testing is the only way to confirm HNRNPH2-disorder. Variants needed to be verified as pathogenic or likely pathogenic to be included in this study.

#### *Recruitment Processes*

The [Yellow Brick](https://yellowbrickroadproject.org/) Road [Project](https://yellowbrickroadproject.org/) is a charitable foundation established by parents of children with HNRNPH2-related disorders. Their mission is to fund research, connecting families, and raising awareness about the condition, and they collect and review genetic reports before families can become a part of the community. We purposely sampled US-based patients in close partnership with the Yellow Brick Road Project to ensure we were only recruiting caregivers of individuals with a confirmed diagnosis.

Representation across the Communication Ability Spectrum

Caregiver enrollment was stratified by child age (1-4, 5-8, and 9-<18 years old). We expected to have all female individuals in this sample due to the x-linked nature of this disorder. As with other cohorts, we reported if the child had seizures or an ASD diagnosis. We also asked caregivers about vision issues that may affect communication, as these may occur in this population.^10^ Finally, we tracked the variant type but did not formally stratify recruitment using this variable, as currently there are no correlations between variant type and phenotype severity.

### Hunter syndrome (Mucopolysaccharidosis Type II (MPS II))

#### Overview

| Incidence |  | 1 in 162,000 |
| --- | --- | --- |
| Description of Disorder^14-21^ | MPS II is an X-linked genetic condition that primarily affects males. It results from a mutation in the iduronate 2-sulfatase gene, which causes changes in a specific enzyme used to break down cellular waste. When that waste builds up, it leads to progressive damage throughout the body. There are two main phenotypes: the severe phenotype is more common (~60-75%) and is associated with severe regression in multiple areas of functioning, hearing loss, seizures, and early mortality in the second decade of life. The attenuated phenotype is typically less severe with normal cognitive and intellectual development. | |
| Foundation (website) | Project Alive (projectalive.org) | |
| Members | 400+ families | |
| Studies (new/ongoing) | Natural history study ongoing; 3 clinical trials underway and 2 launching soon. | |
| PACP Member | Dr. Kim Stephens. President of Project Alive and mother to son with Hunter syndrome. She is a Diversity and Inclusion Manager with experience in the non-profit space as a fundraiser and Executive Director of Muenzer MPS Research & Treatment Center at UNC, Chapel Hill. | |

#### Communication Ability

In one cohort of 73 individuals, communication ability as measured by the Vineland-II was significantly lower for patients with the severe form of Hunter syndrome compared to those with the attenuated form, and it was strongly and negatively correlated with age.^19^ Caregivers have also reported significant impacts in communication ability in other forums. For example, in a recent Voice of the Patient report, 71% of caregivers reported delays in speech, and 57% reported loss of speech in their children.^22^

Moderate to profound hearing loss is expected to occur in Hunter syndrome^21^ and may affect communication ability. Routine screening is recommended.^15^

#### Confirmation of Diagnosis

Diagnosis typically occurs at a young age (generally between 2-4 years old)^14^ and is confirmed by genetic testing. Relevant information from the genetic report includes the specific gene and a determination of “pathogenic” or “likely pathogenic.” Diagnosis for Hunter syndrome is generally well established due to the unique presentation of these children.

#### Recruitment Processes

[Project Alive](https://projectalive.org/) is a nonprofit organization whose mission is “to find and fund a cure for Hunter syndrome through research and advocacy.” We recruited caregivers in close partnership with Project Alive, via their strongly established [Facebook community](https://www.facebook.com/projectalive/) and their email list, providing access to over 400 families. As we recruited from a community setting, we were not be able to ensure molecular diagnosis confirmation in advance. However, we included on all advertisements that this was a requirement for participation and collected this information during screening. Although we did not collect a copy of the genetic report, we were able to verify relevant information (including diagnosis and mutation type) by asking parents to access a copy of their genetic reports and report the relevant information to the study team verbally.

#### Representation across the Communication Ability Spectrum

Age has shown to be negatively related to communication skills in children with the severe form of Hunter syndrome,^19^ reflecting the regressions in skills associated with this disorder. Therefore, we expected caregivers of older individuals to report lower levels of communication ability and modified the age stratification from other groups included in this study to reflect tighter bands to ensure adequate representation across these critical time periods. We aimed to recruit 3 caregivers of individuals within the following age ranges: 1-5 years old, 6-8 years old, 9-11 years old, and 12-<18 years old. Based on reduced life expectancy, we expected that our sample would be smaller in the oldest age group. As Hunter syndrome typically only affects males, our recruitment did not include female children. We also added an NDD-specific screening question that asked caregivers if they have ever been told by a physician that their child has the severe form of Hunter syndrome. We excluded caregivers of individuals with the attenuated form, as they were likely to have cognitive and communication skills in the typically developing range.^19,20^

#### Additional Variables of Interest for Hunter syndrome

For Hunter syndrome, we expected to see differences in the development of early skills (e.g., some children initially develop verbal speech and some do not) and the age and presentation of regressions in skills. Thus, we paid particular attention to these topics in the qualitative interviews and created targeted probes to better understand this phenomena. We also collected information about hearing impairments and probed all caregivers on how hearing loss impacts their child’s communication. Finally, caregivers of individuals with Hunter syndrome were asked the types of treatment or therapy that their child was currently enrolled in, which typically included a description of the child’s involvement in clinical trials. This was important for this group as the individual’s exposure to clinical trials or enzyme replacement therapy (ERT) may impact their overall communication ability.

###

### Malan Syndrome (NFIX)

#### Overview

| INCIDENCE |  | 2.6 in 100,000^23^ |
| --- | --- | --- |
| Description of Disorder^24-29^ | Malan syndrome is linked to de novo mutations in the Nuclear Factor One X (NFIX) gene on Chromosome 19, which results in haploinsufficiency of the NFIX protein. The syndrome is characterized by macrocephaly, intellectual disability, vision and/or hearing impairments, skeletal anomalies, epilepsy and behavioral challenges. | |
| Communication issues | There is a spectrum. Some individuals are completely nonverbal; whereas, others speak in 5+ word combinations. Many individuals are diagnosed with apraxia. Receptive language is often times better than expressive language. | |
| Foundation (website) | Malan Syndrome Foundation (malansyndrome.org) | |
| Members | 300+ families | |
| Studies (new/ongoing) | Registry at Sanford CoRDS and RARE-X. Natural history study for electronic medical records started with Citizen in November 2022. | |
| PACP Member | Dr. Christal Delagrammatikas, Director of Research and co-founder of the Malan Syndrome Foundation; mother to son with Malan syndrome. | |

#### Communication Ability

Malan syndrome (previously called Sotos-2) is an ultra-rare disorder, with less than 100 patients in the literature to date.^24,30^ First described in 2010,^25^ individuals with Malan syndrome present with postnatal overgrowth and intellectual disability.^25-27^  In a detailed study of behavior and development, seven individuals with Malan syndrome (mean 14.6 yo, STD 6.7 years) were assessed using a mix of caregiver surveys, in-person assessments, and semi-structured interviews.^28^ Three of the seven individuals used ‘few words’, with four utilizing sentences to verbally communicate. Six out of the seven individuals had percentile ranks on the Communication domain of the Vineland-II assessment of <0.1 (the 7^th^ individual had percentile rank of 0.4), and developmental ages were estimated to be much younger than the chronological ages. Based on the in-person assessments, assessors noted that expressive language difficulties were “common” and individuals needed longer processing times and supportive associations/gestures, indicating promise for augmentative communication devices.^28^

A more recent study also supported these findings.^29^ Fifteen individuals were assessed using standardized and performance-based measures of cognition, communication, and daily functioning. On the Vineland-Adaptive Behavior Scales-II, communication scores for the sample were low (mean = 35.4). The authors found that seven of the 15 subjects used single word phrases, with five and three individuals utilizing 2-3 words or more complex verbal speech, respectively.^29^

#### Recruitment Processes

The [Malan Syndrome Foundation](https://www.malansyndrome.org/) aims to improve the lives of families and individuals affected by Malan syndrome through support, outreach, and research. As Malan syndrome is an ultra-rare disorder, we initially performed purposeful sampling via the MSF registry, which was hosted by [Sanford CoRDS](https://cordsconnect.sanfordresearch.org/BayaPES/sf/screeningForm?id=SFSFL) and currently includes ~145 participants.

#### Representation across the Communication Ability Spectrum

Caregiver enrollment was stratified by child age (1-4, 5-8, and 9-<18 years old) and caregivers answered a screening question about their child’s language use. Hearing and vision impairments are common for individuals with Malan syndrome,^28^ and thus, we expected more caregivers to be able to speak to how they perceive vision and hearing issues impacting their child’s communication ability. As with other disorders, we asked about other diagnoses, and expected that Autism Spectrum Disorder (ASD) and brain abnormalities (e.g., Chiari malformation)^27^ would be reported.

#### Confirmation of Diagnosis

Malan syndrome is often diagnosed or suspected clinically, as it is an overgrowth disorder. Based on clinical suspicion, molecular genetic testing is typically completed to confirm the *NFIX* haploinsufficiency (loss-of-function variants in NFIX or 19p13 microdeletions encompassing the gene). Variants had to be pathogenic or likely pathogenic to qualify for inclusion in this study. Variant types were classified by the study team in close collaboration with the geneticists and patient advocate representative using Priolo et al.,^27^ as a guide.

###

### Phelan McDermid Syndrome (PMS)

#### Overview

| Incidence |  | 1 in 15,000 |
| --- | --- | --- |
| Description of Disorder^31-33^ | Deletions and pathogenic variants of SHANK3 cause Phelan-McDermid syndrome, characterized by varying degrees of developmental delay, intellectual disability, delayed or absent speech, and autism spectrum disorder or symptoms of autism. | |
| Communication issues^34-40^ | Most individuals with PMS are minimally verbal or nonverbal. Some individuals develop verbal speech and can communicate needs and express thoughts; however, they sometimes have challenges with language (e.g., parents describe processing glitches, challenges with word retrieval). Some individuals are diagnosed with apraxia. Receptive communication is usually better than expressive communication. Some individuals learn to use a communication device. | |
| Foundation (website) | [CureSHANK Foundation](file:///C:\Users\nl154\Desktop\CureSHANK.org) & [Phelan-McDermid Syndrome Foundation](https://pmsf.org/) (PMSF) | |
| Members | 2,774 families | |
| Studies (new/ongoing) | 4 clinical trials in planning phase. | |
| PACP Member | Geraldine Bliss | |

#### Communication Ability

Phelan-McDermid Syndrome (PMS) is characterized by absent to severely delayed speech and intellectual disability.^32,33^ There have been recent calls for more research to better explore communication & language abilities of individuals with PMS.^40^ The estimated prevalence of individuals who can speak in sentences varies from 12-27%.^34,35^ In one study of 39 children with PMS in speech therapy, the majority of individuals were nonverbal but interactive with their environment, with less than 20% indicating the use of verbal speech, 11% using a blend of verbal & nonverbal language, and <8% were nonverbal and non-interactive.^36^ In a study of 15 individuals with PMS, most had low scores on the Communication Profile of the Mentally Handicapped.^34^ Although 3 of the 15 individuals were formally diagnosed with Autism Spectrum Disorder (ASD), 86% of the sample had scores that indicated ASD behaviors were present. Studies have also supported higher scores for receptive communication skills when compared to expressive.^37-39^ A large review paper reported a regression of communication development may be expected around ages 3-4 years in some children.^40^ There is more recent information that supports genotype-phenotype correlations in PMS between Class I and II deletions; children with Class I deletions and sequence variants were more likely to have single word & phrased speech.^41^ Although this link between genotype and phenotype is newly established, we collected information about a child’s deletion to assist in interpretation of our qualitative data.

#### Confirmation of Diagnosis

Genetic testing is required to diagnose a child with PMS. Variants had to be pathogenic or likely pathogenic to qualify for inclusion in this study. In addition to recruiting via a registry with a formal process to confirm diagnosis (see *Recruitment Processes*), pathogenesis and mutation type (e.g., class I or class II deletion) were verified by two genetic counselors at Duke University via review of the child’s genetic report.

#### Recruitment Processes

We recruited via the [Phelan-McDermid Syndrome Foundation](https://pmsf.org/)’s (PMSF) international registry using purposeful sampling methods and focused on English-speaking US-based families. A member of the foundation sent recruitment fliers to eligible families and invited them to reach out to the Duke team for screening and enrollment. PMSF estimates they have around 600 families within their registry who have uploaded a genetic report. The reports have been reviewed by a genetic expert (an MD) to confirm appropriate diagnosis. This approach ensured we limited recruitment to caregivers of individuals with a confirmed diagnosis of PMS.

#### Representation across the Communication Ability Spectrum

As described in the study protocol, caregiver enrollment was stratified by child age (1-4, 5-8, and 9-<18 years old) and caregivers answered a screening question about the child’s language use and ASD diagnosis. We also asked questions during the interview about regressions in skills the caregiver may have noticed.

###

### Schinzel-Giedion Syndrome (SGS)

#### Overview

| Disorder & Incidence |  | Unknown |
| --- | --- | --- |
| Description of Disorder | SGS is an ultra-rare neurodevelopmental disorder arising from de novo gain of function mutations in the SETBP1 gene located on chromosome 18. Prominent features include facial dysmorphism, severe global developmental delay, and seizures. “Classical SGS” patients have an average lifespan of 18-48 months. “Atypical SGS” patients have slightly modified phenotypes and may have a lifespan extending beyond the average.^42^ | |
| Communication issues | There is a spectrum. Majority of classical SGS children are non-verbal. Atypical SGS children can speak a few words and seem to understand some language. Most communicate through eye movements/eye gaze, sounds and gestures. | |
| Foundation (website) | The Schinzel-Giedion Syndrome Foundation (www.sgsfoundation.org) | |
| Members | ~150 families globally, with ~50 children currently alive around the world. | |
| Studies (new/ongoing) | Patient registry and caregiver-entered natural history study on AccrossHealthcare Matrix platform. No clinical trials. | |
| PACP Member | Nuala Summerfield & Dana Bradley | |

#### Communication Ability

Schinzel-Giedion syndrome (SGS) is an ultra-rare neurodevelopmental disorder that affects the SETPBP1 gene. In contrast to SETBP1-HD, which is associated with loss of function mutations, SGS is associated with “gain of function” mutations in which there is an overexpression of the gene. Patients with SGS can fall into two categories: classical or atypical. Patients with classical SGS are severely affected with a life expectancy of 18-48 months, while children with atypical SGS are less affected and may have a longer life span.^43,44^ There are not many studies focusing on the clinical presentation of SGS, and we could not find articles describing communication ability for these children. However, severe intellectual disability is associated with SGS,^45-47^ which impacts communication ability.

#### Confirmation of Diagnosis

Genetic testing is the only way to confirm SGS, and variants needed to be verified as pathogenic or likely pathogenic to be included in this study.^43^ SGS is commonly suspected in infancy based on facial features and early onset of symptoms (e.g. seizures, feeding difficulties).^45^ It also can be suspected based on renal abnormalities detected in utero.^46^

Recruitment Processes

The [Schinzel-Giedion Syndrome Foundation](https://sgsfoundation.org/) is a registered UK charity established in 2019 by parents of children with SGS. Their mission includes providing support for parents and research to improve quality of life of these children. We purposely sampled US-based patients in close partnership with the SGS Foundation, to ensure we were only recruiting caregivers of individuals with a confirmed diagnosis. Due to the rarity of SGS, we also opened enrollment to English-speaking participants in other countries (e.g. United Kingdom, Canada).

Representation across the Communication Ability Spectrum

Caregiver enrollment was stratified by child age (1-4, 5-8, and 9-<18 years old). Due to early mortality associated with SGS, we expected the sample to be younger on average than other groups. To ensure representation across the spectrum of ability levels, we enrolled children with both ‘classical’ and ‘atypical’ SGS.^43,44^

As with other cohorts, we also asked caregivers if the child was diagnosed with epilepsy, which can be difficult to control in SGS.^48^ We also asked caregivers about vision and hearing issues that may impact communication, as these may occur in this population. Finally, we asked caregivers about the medications their child was taking.

###

### SCN2A-related disorders

#### Overview

| **INCIDENCE** |  | **8 in 100,000^23^** |
| --- | --- | --- |
| **DESCRIPTION OF DISORDER^49-54^** | Many individuals with changes in the SCN2A gene have developmental delay/intellectual disability, intractable epilepsy, autism, movement disorders, hypotonia and gastrointestinal problems. Behavioral issues, ataxia and scoliosis are also common in certain cohorts. | |
| **COMMUNICATION ISSUES** | With the exception of one cohort with benign infantile seizures, communication is impaired for all patients with SCN2A-related disorders. Receptive communication is usually much better than expressive communication. Some individuals learn to use a communication device. Additional challenges to language include CVI and fine motor ability. | |
| **FOUNDATION (WEBSITE)** | FamilieSCN2A Foundation (https://www.scn2a.org/) | |
| **MEMBERS** | 1,000 + members | |
| **STUDIES (NEW/ONGOING)** | Natural History Study (Australia,) Ciitizen data collection, Simons Searchlight, SCN2A Clinical Trial Readiness Study | |
| PACP **MEMBER** | Leah Schust Myers | |

#### Communication Ability

SCN2A-related disorders occur in a diverse clinical spectrum,^49-52^ including benign familial neonatal-infantile seizures, Autism Spectrum Disorder (ASD), intellectual disability, and developmental and epileptic encephalopathies. There is support for age of onset of epilepsies being predictive of phenotype and subsequent response to treatment.^53^ To our knowledge, a full description of the spectrum of communication abilities for individuals with SCN2A related disorders has not been published in the literature, although subgroups have been described. For example, in a recent study of 60 individuals with confirmed SCN2A-associated developmental and epileptic encephalopathies, on the Vineland Behavioral Scales-II, the mean standardized score for communication was 56.9 (STD 15.9), which was more than 3 standard deviations below the mean (100).^50^ The authors of this paper purport that this is likely reflective of the large frequency of nonverbal individuals, and they highlighted the slow increase in raw scores over time (which caused standard scores to decline over time).

#### Recruitment Processes

[FamilieSCN2A Foundation](https://www.scn2a.org/scn2a.html) is parent-led non-profit that aims to find effective treatments and a cure for SCN2A related disorders. They are currently conducting a Clinical Trial Readiness Study (CTRS) which collects medical history and survey data. We purposely sampled US-based patients enrolled in this platform, in close partnership with The FamilieSCN2A Foundation, to ensure we only recruited caregivers of individuals with a confirmed diagnosis. Additionally, the CTRS reviews genetic reports to classify variants (see section on *Confirmation of Diagnosis*), and this information was shared with the Duke study team ahead of time to assist with purposeful sampling (see section below).

#### Confirmation of Diagnosis

Genetic testing is the only way to confirm SCN2A-related disorders. Variants must have been verified as pathogenic or likely pathogenic to be included in this study. A current model^52^ classifies variants as leading to ‘loss of function’ (LoF), ‘gain of function’ (GoF), or ‘mixed function’ (‘function’ referring to neuronal excitability), with these types influencing the clinical presentation of the child’s symptoms, including ASD and intellectual disability vs. early onset epilepsy, respectively. The CTRS utilized this model to classify variants, and these classifications were provided to the study team for recruitment purposes.

Representation across the Communication Ability Spectrum

Caregiver enrollment was stratified by child age (1-4, 5-8, and 9-<18 years old) and mutation type. It was expected that children with GoF mutation would have the most severe phenotype. The goal was to enroll at least one child with each mutation type in each age group. Although there is additional support for more gradient phenotype and genotype correlations,^54^ the broad categories related to functioning seemed best suited for this study, with the goal of ensuring representation across the range of communication. As with other cohorts, we also reported if the child was diagnosed with epilepsy, and asked caregivers to report the age of onset of epilepsy, as this can be linked to variants causing ‘gain of function’ or ‘loss of function’.^52^ We also asked caregivers about vision issues that may impact communication, as these may occur in this population.^52^

### SETBP1 Haploinsufficiency disorder (SETBP1-HD)

#### Overview

| Disorder & Incidence |  | 1 in 200,000^11 |
| --- | --- | --- |
| Description of Disorder | An extremely rare disorder caused by de novo pathogenic “loss-of-function” variants and deletions of SETBP1, characterized by varying degrees of developmental delay, intellectual disability, delayed/absent speech, ADHD, and autistic traits. Individuals may also have other comorbid conditions such as hypotonia, gastrointestinal problems, epilepsy, and sleep issues.^55^ | |
| Communication issues | Children have varying communication impairments including absent speech, delayed speech, and motor-planning speech and language disorders, while also being social and motivated to communicate.^56^ It is unknown why some individuals become verbal communicators while others do not. | |
| Foundation (website) | SETBP1 Society (www.setbp1.org) | |
| Members | 250 families connected to foundation, ~75 families in US with confirmed diagnosis. | |
| Studies (new/ongoing) | Registry with Simons Searchlight. No clinical trials. | |
| PACP Member | Haley Oyler | |

#### Communication Ability

Evidence suggests that the SETBP1 gene is associated with language development and speech.^57-59^ In a recent study, a battery of standardized tests were administered to 31 participants with pathogenic SETBP1 variants to assess communication ability and other domains, like motor skills.^56^ Data from parental report alongside clinical evaluations were also used. Eleven individuals were classified as minimally verbal (<50 spoken words), and children who were verbal also presented with delays; with 80% of the sample diagnosed with childhood apraxia of speech. The authors discussed how the results implicated both motor planning and language systems, and indicated that the delays in speech development were worse than delays in other adaptive behavior skills (as per the Vineland-3).^56^ In a related study, 34 patients were evaluated to delineate the clinical presentation of SETBP1-HD. ^59^ The authors found a range of intellectual disability (ID; none to severe ID) with the vast majority having speech delay (97%).

#### Confirmation of Diagnosis

Genetic testing is the only way to confirm SETBP1-HD. Variants involving the SETPB1 gene must have been verified as pathogenic or likely pathogenic to be included in this study. Since the primary phenotype does not involve seizures, structural barriers exist that limit the families receiving genetic testing, this in turn affects identification of patients and may influence the potential diversity of the enrolled sample.

#### Recruitment Processes

[SETBP1 Society](https://www.setbp1.org/welcome/) is a nonprofit organization created and run by parents of children with SETBP1-HD. As part of entry into the community, the organization collects genetic reports from families to confirm diagnosis. The study team purposely sampled US-based patients in close partnership with the SETBP1 Society, ensuring recruitment of caregivers of individuals with a confirmed diagnosis. Due to the rarity of SETBP1-HD, we also opened enrollment to English-speaking participants in other countries (e.g. United Kingdom, Canada).

#### Representation across the Communication Ability Spectrum

Caregiver enrollment for SETBP1-HD was stratified by child age (1-4, 5-8, 9-11 and 12-<18 years old). These age bands were based on the spectrum of ability levels and the need to ensure representation across developmental stages, particularly when first words typically occur.^56,59^ As with other cohorts, we asked caregivers about vision issues that may impact communication, as these may occur in this population but tend to be mild.^59^ As age at diagnosis can vary due to evolving access to genetic testing and establishment of SETBP1-HD diagnosis, we also asked caregivers to report the age that their child was diagnosed. In the study described previously, 8/16 participants had autism-like traits, but only three had a formal diagnosis, and described participants as “sociable with a strong desire to communicate.”^56^ Thus, we asked caregivers about ASD diagnosis and severity of their child’s intellectual disability (if known).

###

### STXBP1-related disorders

#### Overview

| INCIDENCE |  | 3.6 in 100,000^23^ |
| --- | --- | --- |
| Description of Disorder^23,60-65^ | Syntaxin-binding protein 1 (STXBP1) is caused by de novo mutations in the STXBP1 gene. This autosomal dominant disorder leads to impaired neurotransmission and compromised brain function. Patients exhibit a range of clinical features, with seizures as a dominant feature typically beginning in infancy. Global developmental delay, severe cognitive impairment, movement disorders, hypotonia, hypertonia, and autistic features are also common. | |
| Foundation (website) | STXBP1 Foundation (stxbp1disorders.org) | |
| Members | 1150+ families | |
| Studies (new/ongoing) | Registry at Simons Searchlight and RARE-X. Natural history study for electronic medical records launched April 2021. First clinical trial began in February 2021. Prospective natural history study launched July 2023. | |
| PACP Member | Charlene Son Rigby. President and co-founder of STXBP1 Foundation and mother to daughter with STXBP1-related disorder. She works in biotech and patient-advocacy. | |

#### Communication Ability

To our knowledge, a full qualitative description of the spectrum of communication abilities for individuals with STXBP1-related disorders has not been published in the literature. However, in a sample of 14 individuals with confirmed STXBP1 mutations, speech and language acquisition was delayed for all, with eight of those individuals utilizing at least some verbal communication.^65^ In the same sample, some individuals were reportedly able to form short phrases, and Vineland Communication standard scores ranged from 33 to 67, indicating moderate to severe impairment. Additionally, communication was more severe in individuals with STXBP1-related disorders when compared to a comparison group with intellectual disabilities, although their scores for global adaptive functioning were similar.^65^ In another sample of 45 individuals, seven were able to utilize some degree of verbal communication (defined by authors as “more than a few words”).^60^

#### Confirmation of Diagnosis

Genetic testing is required to diagnose a child with an STXBP1-related disorder. Relevant information from the genetic report includes the indication of a variant in the STXBP1 gene and that the variant is pathogenic or likely pathogenic. Although there is currently not a well-established relationship between specific mutations and severity levels, we collected this information to assist in interpretation of our qualitative data.

#### Recruitment Processes

[The STXBP1 Foundation](https://www.stxbp1disorders.org/what-is-stxbp1) is a parent-led organization that is dedicated to creating awareness and finding a cure for STXBP1-related disorders. They currently have initiated three natural history studies hosted by [Simons Searchlight](https://www.stxbp1disorders.org/simonssearchlight), [RARE-X](https://rare-x.org/), and [Ciitizen](https://www.ciitizen.com/research/) (now part of Invitae). Ciitizen collects medical records, Simons collects medical history and survey data, and RARE-X collects patient-reported survey data. The Simons Searchlight study is also open to international English-speaking families. To confirm diagnosis, we recruited caregivers with children enrolled in these platforms, in close partnership with the STXBP1 Foundation. Further, caregivers were able to easily access a copy of their child’s genetic report through Ciitizen, which facilitated the Duke team’s ability to verify relevant information (including mutation type).

#### Representation across the Communication Ability Spectrum

As described in the study protocol, caregiver enrollment was stratified by child age (1-4, 5-8, and 9-<18 years old) and caregivers answered a screening question about the child’s language use (e.g., no words, few words, short phrases or full sentences). Based on our overall understanding of STXBP1-related disorders, we expected that communication ability levels would be similar to those seen in Angelman syndrome.

### SYNGAP1-related intellectual disability

#### Overview

| Incidence |  | 6 in 100,000^23^ |
| --- | --- | --- |
| Description of Disorder^66-71^ | Children with this disorder have a pathogenic variant in the gene SYNGAP1, which disrupts the functioning of synapses in the brain. SYNGAP1 is considered a spectrum disorder since all patients are not affected exactly the same way or with the same severity. The most common symptoms include intellectual disability (moderate to severe), hypotonia (low muscle tone), epilepsy, global development delay, sensory processing disorder, feeding & swallowing issues, gross and fine motor skill delays, dyspraxia, and speech delay/apraxia. | |
| Foundation (website) | SynGAP Research Fund (https://www.cureSYNGAP1.org/) | |
| Members | 1,500+ families (seehttps://curesyngap1.org/how-many-people-have-syngap1-census/) | |
| Studies (new/ongoing) | Launched a retrospective natural history study on 9/1/20 and prospective NHS on 10/1/23 that has expanded to three sites, one clinical trial ongoing, multiple in planning phase. | |
| PACP Member | Mike Graglia. Managing Director and co-Founder of SynGap Research Fund and father of son with SYNGAP1. | |

#### Communication Ability

To our knowledge, a full description of the spectrum of communication abilities for individuals with SYNGAP1-related intellectual disability (SYNGAP1) has not been published in the literature. However, in one of the largest international cohorts of 57 individuals with likely pathogenic SYNGAP1 variants, severe communication and language deficits were reported across the sample.^70^ In the international study, twelve individuals (21%) were completely nonverbal (ages ranged from 2 to 33 years old). Developmental delay was seen in all children, with 55 of the 57 meeting criteria for intellectual disability (50 being moderate to severe).^70^ Based on previously described clinical cases and discussions with parent representatives, we expected a rather wide range of ability levels within this population (please see Holder et al, 2019 for a detailed list of case reports of individuals with SYNGAP1).

#### Confirmation of Diagnosis

Genetic testing is required to diagnose a child with SYNGAP1. Relevant information from the genetic testing report includes the indication of a variant in the SYNGAP1 gene and that the variant is pathogenic or likely pathogenic. Although there is currently not a well-established relationship between specific mutations and severity levels, we collected this information to assist in interpretation of our qualitative data.

#### Recruitment Processes

[SynGAP Research Fund](https://www.syngapresearchfund.org/) (SRF) is a global patient advocacy organization whose mission is to “support the research and development of treatments, therapies, and support systems for SynGAP1 patients worldwide.” SRF has recently initiated a digital natural history study hosted by [Ciitizen](https://www.ciitizen.com/) (now Invitae, see the recent [press release](https://www.prnewswire.com/news-releases/invitae-to-acquire-ciitizen-to-strengthen-its-patient-consented-health-data-platform-to-improve-personal-outcomes-and-global-research-301369974.html)). Ciitizen collects patient medical record information, including genetic reports, which are reviewed and verified by study personnel. To ensure that we only recruited caregivers with a child with a confirmed diagnosis of SYNGAP1, we recruited patients enrolled in this natural history study. Additionally, caregivers could easily access a copy of their child’s genetic report through Ciitizen, which facilitated the Duke team’s ability to verify the relevant information (including mutation/genotype).

#### Representation across the Communication Ability Spectrum

As described in the study protocol, caregiver enrollment was stratified by child age (1-4, 5-8, and 9-<18 years old) and caregivers answered a screening question about the child’s language use (e.g., no words, few words, short phrases or full sentences). Based on our overall understanding of SYNGAP1, we expected that communication behaviors would be similar to those seen in Angelman syndrome.

# References

1. Chen CA, Bosch DG, Cho MT, et al. The expanding clinical phenotype of Bosch-Boonstra-Schaaf optic atrophy syndrome: 20 new cases and possible genotype-phenotype correlations. *Genet Med*. Nov 2016;18(11):1143-1150. doi:10.1038/gim.2016.18

2. Bosch DG, Boonstra FN, Gonzaga-Jauregui C, et al. NR2F1 mutations cause optic atrophy with intellectual disability. *Am J Hum Genet*. Feb 6 2014;94(2):303-9. doi:10.1016/j.ajhg.2014.01.002

3. Rech ME, McCarthy JM, Chen CA, et al. Phenotypic expansion of Bosch-Boonstra-Schaaf optic atrophy syndrome and further evidence for genotype-phenotype correlations. *American journal of medical genetics Part A*. Jun 2020;182(6):1426-1437. doi:10.1002/ajmg.a.61580

4. Platzer K, Lemke JR. GRIN2B-Related Neurodevelopmental Disorder. In: Adam MP, Mirzaa GM, Pagon RA, Wallace SE, Bean LJH, Gripp KW, Amemiya A, eds. *GeneReviews(®)*. University of Washington, Seattle

Copyright © 1993-2023, University of Washington, Seattle. GeneReviews is a registered trademark of the University of Washington, Seattle. All rights reserved.; 1993.

5. Freunscht I, Popp B, Blank R, et al. Behavioral phenotype in five individuals with de novo mutations within the GRIN2B gene. *Behav Brain Funct*. May 29 2013;9:20. doi:10.1186/1744-9081-9-20

6. Hao YH, Fountain MD, Jr., Fon Tacer K, et al. USP7 Acts as a Molecular Rheostat to Promote WASH-Dependent Endosomal Protein Recycling and Is Mutated in a Human Neurodevelopmental Disorder. *Mol Cell*. Sep 17 2015;59(6):956-69. doi:10.1016/j.molcel.2015.07.033

7. Fountain MD, Oleson DS, Rech ME, et al. Pathogenic variants in USP7 cause a neurodevelopmental disorder with speech delays, altered behavior, and neurologic anomalies. *Genet Med*. Aug 2019;21(8):1797-1807. doi:10.1038/s41436-019-0433-1

8. Marbach F, Schaaf CP. Response to Briuglia et al. *Genetics in Medicine*. 2021/02/01 2021;23(2):423-424. doi:10.1038/s41436-020-00977-y

9. Bain JM, Cho MT, Telegrafi A, et al. Variants in HNRNPH2 on the X Chromosome Are Associated with a Neurodevelopmental Disorder in Females. *Am J Hum Genet*. Sep 1 2016;99(3):728-734. doi:10.1016/j.ajhg.2016.06.028

10. Bain JM, Thornburg O, Pan C, et al. Detailed Clinical and Psychological Phenotype of the X-linked HNRNPH2-Related Neurodevelopmental Disorder. *Neurol Genet*. Feb 2021;7(1):e551. doi:10.1212/nxg.0000000000000551

11. HNRNPH2 Genetics 101. Accessed July 8, 2022. <https://yellowbrickroadproject.org/pages/hnrnph2-genetics-101>

12. Somashekar PH, Narayanan DL, Jagadeesh S, et al. Bain type of X-linked syndromic mental retardation in a male with a pathogenic variant in HNRNPH2. *American journal of medical genetics Part A*. Jan 2020;182(1):183-188. doi:10.1002/ajmg.a.61388

13. Salazar R, Beenders S, LaMarca NM, et al. Cross-sectional, quantitative analysis of motor function in females with HNRNPH2-related disorder. *Research in developmental disabilities*. 2021/12/01/ 2021;119:104110. doi:<https://doi.org/10.1016/j.ridd.2021.104110>

14. Mucopolysaccharidosis Type II. Accessed 9-3-2021, <https://rarediseases.org/rare-diseases/mucopolysaccharidosis-type-ii-2/>

15. Muenzer J, Beck M, Eng CM, et al. Multidisciplinary management of Hunter syndrome. *Pediatrics*. Dec 2009;124(6):e1228-39. doi:10.1542/peds.2008-0999

16. Young ID, Harper PS, Newcombe RG, Archer IM. A clinical and genetic study of Hunter's syndrome. 2. Differences between the mild and severe forms. *J Med Genet*. Dec 1982;19(6):408-11. doi:10.1136/jmg.19.6.408

17. Coppa GV, Buzzega D, Zampini L, et al. Plasmatic and Urinary Glycosaminoglycans Characterization in Mucopolysaccharidosis II Patient Treated with Enzyme-Replacement Therapy with Idursulfase. *JIMD Rep*. 2012;4:79-90. doi:10.1007/8904_2011_75

18. Wraith JE, Scarpa M, Beck M, et al. Mucopolysaccharidosis type II (Hunter syndrome): a clinical review and recommendations for treatment in the era of enzyme replacement therapy. *Eur J Pediatr*. Mar 2008;167(3):267-77. doi:10.1007/s00431-007-0635-4

19. Hashmi MS GVI. Mucopolysaccharidosis Type II. 2021 Aug 1. Available from: <https://www.ncbi.nlm.nih.gov/books/NBK560829/>

20. Needham M, Packman W, Rappoport M, et al. MPS II: adaptive behavior of patients and impact on the family system. *J Genet Couns*. Jun 2014;23(3):330-8. doi:10.1007/s10897-013-9665-4

21. Cho YS, Kim JH, Kim TW, Chung SC, Chang SA, Jin DK. Otologic manifestations of Hunter syndrome and their relationship with speech development. *Audiol Neurootol*. 2008;13(3):206-12. doi:10.1159/000113511

22. Stephens KPA. *The Voice of the Patient: Summary report resulting from an externally conducted patient-focused survey*. 2019. <https://projectalive.org/wp-content/uploads/2021/07/VOP_Hunter_Syndrome.pdf>

23. López-Rivera JA, Pérez-Palma E, Symonds J, et al. A catalogue of new incidence estimates of monogenic neurodevelopmental disorders caused by de novo variants. *Brain*. Apr 1 2020;143(4):1099-1105. doi:10.1093/brain/awaa051

24. Malan overgrowth syndrome. . Accessed January, 25 2022. <https://www.orpha.net/consor/cgi-bin/OC_Exp.php?lng=EN&Expert=420179>

25. Malan V, Rajan D, Thomas S, et al. Distinct effects of allelic NFIX mutations on nonsense-mediated mRNA decay engender either a Sotos-like or a Marshall-Smith syndrome. *Am J Hum Genet*. Aug 13 2010;87(2):189-98. doi:10.1016/j.ajhg.2010.07.001

26. Klaassens M, Morrogh D, Rosser EM, et al. Malan syndrome: Sotos-like overgrowth with de novo NFIX sequence variants and deletions in six new patients and a review of the literature. *Eur J Hum Genet*. May 2015;23(5):610-5. doi:10.1038/ejhg.2014.162

27. Priolo M, Schanze D, Tatton-Brown K, et al. Further delineation of Malan syndrome. *Human mutation*. 2018;39(9):1226-1237. doi:<https://doi.org/10.1002/humu.23563>

28. Mulder PA, van Balkom IDC, Landlust AM, et al. Development, behaviour and sensory processing in Marshall-Smith syndrome and Malan syndrome: phenotype comparison in two related syndromes. *J Intellect Disabil Res*. Dec 2020;64(12):956-969. doi:10.1111/jir.12787

29. Alfieri P, Macchiaiolo M, Collotta M, et al. Characterization of Cognitive, Language and Adaptive Profiles of Children and Adolescents with Malan Syndrome. *Journal of Clinical Medicine*. 2022;11(14):4078.

30. Macchiaiolo M, Panfili FM, Vecchio D, et al. A deep phenotyping experience: up to date in management and diagnosis of Malan syndrome in a single center surveillance report. *Orphanet J Rare Dis*. Jun 18 2022;17(1):235. doi:10.1186/s13023-022-02384-9

31. Phelan K, McDermid HE. The 22q13.3 Deletion Syndrome (Phelan-McDermid Syndrome). *Mol Syndromol*. Apr 2012;2(3-5):186-201. doi:10.1159/000334260

32. Phelan K, Rogers RC, Boccuto L. Phelan-McDermid Syndrome. In: Adam MP, Mirzaa GM, Pagon RA, Wallace SE, Bean LJH, Gripp KW, Amemiya A, eds. *GeneReviews(®)*. University of Washington, Seattle

Copyright © 1993-2023, University of Washington, Seattle. GeneReviews is a registered trademark of the University of Washington, Seattle. All rights reserved.; 1993.

33. Phelan-McDermid Syndrome Accessed 12-10, 2021 <https://rarediseases.org/rare-diseases/phelan-mcdermid-syndrome/>

34. Droogmans G, Swillen A, Van Buggenhout G. Deep Phenotyping of Development, Communication and Behaviour in Phelan-McDermid Syndrome. *Mol Syndromol*. Jan 2020;10(6):294-305. doi:10.1159/000503840

35. Samogy-Costa CI, Varella-Branco E, Monfardini F, et al. A Brazilian cohort of individuals with Phelan-McDermid syndrome: genotype-phenotype correlation and identification of an atypical case. *J Neurodev Disord*. Jul 18 2019;11(1):13. doi:10.1186/s11689-019-9273-1

36. Meersman T, Mathieson K. Examining factors affecting parental satisfaction with speech therapy in children with Phelan-McDermid Syndrome. *Int J Dev Disabil*. Mar 27 2019;66(4):304-316. doi:10.1080/20473869.2019.1582906

37. Phelan MC, Rogers RC, Saul RA, et al. 22q13 deletion syndrome. *Am J Med Genet*. Jun 15 2001;101(2):91-9. doi:10.1002/1096-8628(20010615)101:2<91::aid-ajmg1340>3.0.co;2-c

38. Soorya L, Kolevzon A, Zweifach J, et al. Prospective investigation of autism and genotype-phenotype correlations in 22q13 deletion syndrome and SHANK3 deficiency. *Mol Autism*. Jun 11 2013;4(1):18. doi:10.1186/2040-2392-4-18

39. Zwanenburg RJ, Ruiter SA, van den Heuvel ER, Flapper BC, Van Ravenswaaij-Arts CM. Developmental phenotype in Phelan-McDermid (22q13.3 deletion) syndrome: a systematic and prospective study in 34 children. *J Neurodev Disord*. 2016;8:16. doi:10.1186/s11689-016-9150-0

40. Vogels A, Droogmans G, Vergaelen E, Van Buggenhout G, Swillen A. Recent developments in Phelan-McDermid syndrome research: an update on cognitive development, communication and psychiatric disorders. *Curr Opin Psychiatry*. Mar 1 2021;34(2):118-122. doi:10.1097/yco.0000000000000672

41. Levy T, Foss-Feig JH, Betancur C, et al. Strong evidence for genotype-phenotype correlations in Phelan-McDermid syndrome: results from the developmental synaptopathies consortium. *Hum Mol Genet*. Feb 21 2022;31(4):625-637. doi:10.1093/hmg/ddab280

42. Schinzel-Giedion Syndrome. Accessed July 8, 2022. <https://rarediseases.org/rare-diseases/schinzel-giedion-syndrome/#:~:text=Schinzel%20Giedion%20syndrome%20(SGS)%20is,and%20damaged%20kidneys%20(hydronephrosis>)

43. Acuna-Hidalgo R, Deriziotis P, Steehouwer M, et al. Overlapping SETBP1 gain-of-function mutations in Schinzel-Giedion syndrome and hematologic malignancies. *PLoS Genet*. Mar 2017;13(3):e1006683. doi:10.1371/journal.pgen.1006683

44. About SGS. Accessed July 8, 2022. <https://sgsfoundation.org/about-sgs/>

45. Al-Mudaffer M, Oley C, Price S, Hayes I, Stewart A, Hall CM, Reardon W. Clinical and radiological findings in Schinzel-Giedion syndrome. *Eur J Pediatr*. Dec 2008;167(12):1399-407. doi:10.1007/s00431-008-0683-4

46. Labrune P, Lyonnet S, Zupan V, Imbert MC, Goutieres F, Hubert P, Le Merrer M. Three new cases of the Schinzel-Giedion syndrome and review of the literature. *Am J Med Genet*. Mar 1 1994;50(1):90-3. doi:10.1002/ajmg.1320500120

47. Hoischen A, van Bon BW, Gilissen C, et al. De novo mutations of SETBP1 cause Schinzel-Giedion syndrome. *Nat Genet*. Jun 2010;42(6):483-5. doi:10.1038/ng.581

48. Minn D, Christmann D, De Saint-Martin A, et al. Further clinical and sensorial delineation of Schinzel-Giedion syndrome: report of two cases. *Am J Med Genet*. May 1 2002;109(3):211-7. doi:10.1002/ajmg.10348

49. Sanders SJ, Murtha MT, Gupta AR, et al. De novo mutations revealed by whole-exome sequencing are strongly associated with autism. *Nature*. 2012;485:237-241. doi:10.1038/nature10945

50. Berg AT, Palac H, Wilkening G, Zelko F, Schust Meyer L. SCN2A-Developmental and Epileptic Encephalopathies: Challenges to trial-readiness for non-seizure outcomes. *Epilepsia*. Jan 2021;62(1):258-268. doi:10.1111/epi.16750

51. Herlenius E, Heron SE, Grinton BE, Keay D, Scheffer IE, Mulley JC, Berkovic SF. SCN2A mutations and benign familial neonatal-infantile seizures: the phenotypic spectrum. *Epilepsia*. Jun 2007;48(6):1138-42. doi:10.1111/j.1528-1167.2007.01049.x

52. Sanders SJ, Campbell AJ, Cottrell JR, et al. Progress in Understanding and Treating SCN2A-Mediated Disorders. *Trends Neurosci*. Jul 2018;41(7):442-456. doi:10.1016/j.tins.2018.03.011

53. Wolff M, Johannesen KM, Hedrich UBS, et al. Genetic and phenotypic heterogeneity suggest therapeutic implications in SCN2A-related disorders. *Brain*. May 1 2017;140(5):1316-1336. doi:10.1093/brain/awx054

54. Crawford K, Xian J, Helbig KL, et al. Computational analysis of 10,860 phenotypic annotations in individuals with SCN2A-related disorders. *Genetics in medicine : official journal of the American College of Medical Genetics*. 2021/07// 2021;23(7):1263-1272. doi:10.1038/s41436-021-01120-1

55. SETBP1 Haploinfufficiency Disorder. Accessed July 8, 2022. <https://rarediseases.org/rare-diseases/setbp1-disorder/>

56. Morgan A, Braden R, Wong MMK, et al. Speech and language deficits are central to SETBP1 haploinsufficiency disorder. *Eur J Hum Genet*. Aug 2021;29(8):1216-1225. doi:10.1038/s41431-021-00894-x

57. Hildebrand MS, Jackson VE, Scerri TS, et al. Severe childhood speech disorder. *Gene discovery highlights transcriptional dysregulation*. 2020;94(20):e2148-e2167. doi:10.1212/wnl.0000000000009441

58. Marseglia G, Scordo MR, Pescucci C, et al. 372 kb microdeletion in 18q12.3 causing SETBP1 haploinsufficiency associated with mild mental retardation and expressive speech impairment. *Eur J Med Genet*. Mar 2012;55(3):216-21. doi:10.1016/j.ejmg.2012.01.005

59. Jansen NA, Braden RO, Srivastava S, et al. Clinical delineation of SETBP1 haploinsufficiency disorder. *Eur J Hum Genet*. Aug 2021;29(8):1198-1205. doi:10.1038/s41431-021-00888-9

60. Stamberger H, Nikanorova M, Willemsen MH, et al. STXBP1 encephalopathy: A neurodevelopmental disorder including epilepsy. *Neurology*. Mar 8 2016;86(10):954-62. doi:10.1212/wnl.0000000000002457

61. Abramov D, Guiberson NGL, Burré J. STXBP1 encephalopathies: Clinical spectrum, disease mechanisms, and therapeutic strategies. *J Neurochem*. Apr 2021;157(2):165-178. doi:10.1111/jnc.15120

62. Guiberson NGL, Pineda A, Abramov D, et al. Mechanism-based rescue of Munc18-1 dysfunction in varied encephalopathies by chemical chaperones. *Nature Communications*. 2018/09/28 2018;9(1):3986. doi:10.1038/s41467-018-06507-4

63. Deák F, Xu Y, Chang WP, et al. Munc18-1 binding to the neuronal SNARE complex controls synaptic vesicle priming. *J Cell Biol*. Mar 9 2009;184(5):751-64. doi:10.1083/jcb.200812026

64. Saitsu H, Kato M, Mizuguchi T, et al. De novo mutations in the gene encoding STXBP1 (MUNC18-1) cause early infantile epileptic encephalopathy. *Nat Genet*. Jun 2008;40(6):782-8. doi:10.1038/ng.150

65. O’Brien S, Ng-Cordell E, Astle DE, Scerif G, Baker K, The DDDS. STXBP1-associated neurodevelopmental disorder: a comparative study of behavioural characteristics. *J Neurodev Disord*. 2019/08/06 2019;11(1):17. doi:10.1186/s11689-019-9278-9

66. Berryer MH, Hamdan FF, Klitten LL, et al. Mutations in SYNGAP1 cause intellectual disability, autism, and a specific form of epilepsy by inducing haploinsufficiency. *Human mutation*. Feb 2013;34(2):385-94. doi:10.1002/humu.22248

67. Clement JP, Aceti M, Creson TK, et al. Pathogenic SYNGAP1 mutations impair cognitive development by disrupting maturation of dendritic spine synapses. *Cell*. Nov 9 2012;151(4):709-723. doi:10.1016/j.cell.2012.08.045

68. Hamdan FF, Gauthier J, Spiegelman D, et al. Mutations in SYNGAP1 in autosomal nonsyndromic mental retardation. *N Engl J Med*. Feb 5 2009;360(6):599-605. doi:10.1056/NEJMoa0805392

69. Kim JH, Liao D, Lau LF, Huganir RL. SynGAP: a synaptic RasGAP that associates with the PSD-95/SAP90 protein family. *Neuron*. Apr 1998;20(4):683-91. doi:10.1016/s0896-6273(00)81008-9

70. Holder JL, Jr., Hamdan FF, Michaud JL. SYNGAP1-Related Intellectual Disability. In: Adam MP, Mirzaa GM, Pagon RA, Wallace SE, Bean LJH, Gripp KW, Amemiya A, eds. *GeneReviews(®)*. University of Washington, Seattle

Copyright © 1993-2023, University of Washington, Seattle. GeneReviews is a registered trademark of the University of Washington, Seattle. All rights reserved.; 1993.

71. Vlaskamp DRM, Shaw BJ, Burgess R, et al. SYNGAP1 encephalopathy: A distinctive generalized developmental and epileptic encephalopathy. *Neurology*. Jan 8 2019;92(2):e96-e107. doi:10.1212/wnl.0000000000006729
