# Supplementary material for: The spectrum of communication abilities in children with 12 rare neurodevelopmental disorders: a qualitative study with caregivers
Source: J Child Psychol Psychiatry. 2025 Oct 20;67(5):740–54. doi: 10.1111/jcpp.70063 (PMC13102052; doi:10.1111/jcpp.70063)
Supplement: Supplementary file 2 — Appendix S2. Additional results; including Tables S1–S91. [file JCPP-67-740-s002.docx]

The spectrum of communication abilities in children with twelve rare neurodevelopmental disorders: A qualitative study with caregivers

Supporting Information

**Appendix S2:** Additional results

Overview of document structure

To supplement the overall results in the main paper, results for research questions 1-4 are presented for each NDD as well as for clinicians.

Relevant demographics for each NDD sample are presented within those NDD-specific sections.

Research questions 5 & 6 are summarized across NDDs.

** Please note, although counts are reported to indicate salience of each theme, these data are limited in that they do not indicate *all* communication behaviors relevant to each child, only the ones that caregivers discussed during the interview.

Table S1. Research questions driving the qualitative analysis of the caregiver interviews.

|  | Presented by NDD |
| --- | --- |
| 1 | Within each NDD, how do caregivers describe their child’s typical communication ability? |
| 2 | How do caregivers describe changes over time they have observed in their child’s communication ability? |
| 3 | As per caregivers, how do hearing and vision impairments impact their child’s communication ability? |
| 4 | What do caregivers see as a meaningful change in communication ability? |
|  | **Presented across NDD** |
| 5 | How much does the current content of Observer-Reported Communication Ability (ORCA) measure capture communication concepts and behaviors described by caregivers and clinicians across the NDD and how could it be expanded to improve content validity for the NDDs included in this project? |
| 6 | How do the above research questions vary across NDDs? |

Contents

[Results for Research Questions 1-4 3](#_Toc200459916)

[1. Bosch-Boonstra-Schaaf optic atrophy syndrome (BBSOAS) 3](#_Toc200459917)

[2. GRIN2B-related neurodevelopmental disorder 14](#_Toc200459918)

[3. Hao-Fountain Syndrome (HAFOUS) 24](#_Toc200459919)

[4. HNRNPH2-related disorders 34](#_Toc200459920)

[5. Hunter syndrome 47](#_Toc200459921)

[6. Malan syndrome (NFIX) 59](#_Toc200459922)

[7. Phelan McDermid Syndrome (PMS) 73](#_Toc200459923)

[8. Schinzel-Giedion syndrome (SGS) 84](#_Toc200459924)

[9. SCN2A-related disorders 92](#_Toc200459925)

[10. SETBP1-haploinsufficiency disorder (SETBP1-HD) 102](#_Toc200459926)

[11. STXBP1-related disorders 112](#_Toc200459927)

[12. SYNGAP1-related intellectual disability 124](#_Toc200459928)

[13. Clinical Experts 135](#_Toc200459929)

[NDD Specific Quotes on Novel Communication Topics Identified During Interviews 145](#_Toc200459930)

[Results for Research Question 6: Findings across NDDs 154](#_Toc200459931)

[Frustration Due to Communication Issues & Impacts on the Family 154](#_Toc200459932)

[Medical Issues & Communication Ability 158](#_Toc200459933)

[Mood & Communication 159](#_Toc200459934)

## Results for Research Questions 1-4

### Bosch-Boonstra-Schaaf optic atrophy syndrome (BBSOAS)

#### Demographics

Eleven caregivers of individuals with BBSOAS participated. Most caregivers were female and on average 37.9 years of age (Table S2). Two caregivers self-identified as Hispanic-Latino. Eight caregivers identified as white, one as African American or Black, and two as mixed race. Most caregivers reported that they were currently married or living with a domestic partner. Overall, the participants reported high levels of education, employment, and annual income (Table S2).

Table S2. Demographic information for 11 caregivers of individuals with BBSOAS.

| *Caregivers* | *n (%)* |
| --- | --- |
| Female | 10(90.9) |
| Age, years (Mean/SD) | 37.9/4.7 |
| Ethnicity |  |
| Not Hispanic or Latino | 9(81.8) |
| Hispanic-Latino | 2(18.2) |
| Race |  |
| White | 8(72.7) |
| African-American or Black | 1(9.1) |
| American Indian/Alaska Native | 0 |
| Asian | 0 |
| Middle Eastern | 0 |
| Native Hawaiian/Other Pacific Islander | 0 |
| More than one race* | 2(18.2) |
| Relationship status |  |
| Single, never married | 0 |
| Married, or living with domestic partner | 10(90.9) |
| Separated | 0 |
| Divorced | 1(9.1) |
| Widowed | 0 |
| Highest grade in school |  |
| Less than high school diploma | 0 |
| High school degree or equivalent | 1(9.1) |
| Some college/University | 2(18.2) |
| College/University degree | 4(36.4) |
| Postgraduate degree | 4(36.4) |
| Occupational status |  |
| Homemaker | 0 |
| Unemployed | 0 |
| Retired | 0 |
| On disability | 0 |
| On leave of absence | 0 |
| Full-time employed | 5(45.5) |
| Part-time employed | 6(54.5) |
| Full-time student only | 0 |
| Income of U.S. residents |  |
| Less than $20,000 | 1(9.1) |
| Between $20,001 and $40,000 | 0 |
| Between $40,001 and $60,000 | 1(9.1) |
| Between $60,001 and $80,000 | 2(18.2) |
| Between $80,001 and $100,000 | 1(9.1) |
| Between $100,001 and $250,000 | 4(36.4) |
| Between $250,001 and $500,000 | 1(9.1) |
| $500,000+ | 1(9.1) |
| I prefer not to answer | 0 |
| I do not know | 0 |
| Relation to child |  |
| Mother/Step-mother | 10(90.9) |
| Father/Step-father | 1(9.1) |

Note: *White and African-American or Black (n=1), White, American Indian/Alaska Native, and Asian (n=1)

Caregivers also provided demographic information for their child with BBOSAS. Nine children were female, with similar rates to caregivers in regards to ethnicity and race (Table S3). All mutations were considered missense, CNV/deletion, or initiation codon variants.

Four caregivers reported their children had an ASD diagnosis, and four reported that their child had epilepsy. Some children were receiving therapeutic services with most (10/11) receiving occupational therapy and five receiving vision therapy (Table S3). Three caregivers reported that their child used a high-tech AAC device. The settings in which the device was used varied (Table S3).

When asked about their child’s verbal language, three caregivers indicated their child used ‘full sentences’, one indicated their child used ‘short phrases’, three indicated their child used a ‘few words’, and four indicated their child used ‘no words’.

Table S3. Demographic information for 11 children with BBSOAS.

| *Children* | *n (%)* |
| --- | --- |
| Age, years (Mean/SD) | 7.1/2.9 |
| Child gender Female | 9(81.8) |
| Ethnicity |  |
| Not Hispanic or Latino | 8(72.7) |
| Hispanic-Latino | 3(27.3) |
| Race |  |
| White | 9(81.8) |
| African-American or Black | 1(9.1) |
| American Indian/Alaska Native | 0 |
| Asian | 0 |
| Middle Eastern | 0 |
| Native Hawaiian/Other Pacific Islander | 0 |
| More than one race* | 1(9.1) |
| Child’s genotype |  |
| Missense | 5(45.5) |
| CNV/deletion | 4(36.3) |
| Initiation codon variant | 2(18.2) |
| Autism Spectrum Disorder (ASD) | 4(36.4) |
| Epilepsy | 4(36.4) |
| Types of therapy |  |
| Physical Therapy | 6(54.6) |
| Occupational Therapy | 10(90.9) |
| Speech Therapy | 8(72.7) |
| Other therapy  Aquatic Therapy  Feeding Therapy  Hearing Therapy  Music Therapy  Resource Therapy  Vision Therapy | 1(9.1)  1(9.1)  1(9.1)  1(9.1)  1(9.1)  6(54.6) |
| Age first introduced to device (Mean/SD) | 5.0/2.6 |
| AAC device |  |
| No | 8(72.7) |
| Yes | 3(27.3) |
| Device |  |
| High tech | 3(27.3) |
| Low tech | 1(9.1) |
| Places to use the device |  |
| Home | 1(9.1) |
| School | 2(18.2) |
| Out in the community | 0 |
| Other - Therapy | 1(9.1) |

Note: *White and African-American or Black

#### Typical Communication Ability

The results for this section are organized by behaviors that correspond to expressive, receptive, and pragmatic (i.e. social) communication (Table S4), aligning with the conceptual framework that was developed for individuals with Angelman syndrome.^1^ Please note, although counts are reported to indicate salience of each theme, these data are limited in that they do not indicate *all* communication behaviors relevant to each child, only the ones that caregivers discussed during the interview.

Table S4. Number of caregivers that mentioned one or more examples of expressive, receptive, and/or pragmatic communication.

| Communication Behaviors | *n* (total *n*=11) |
| --- | --- |
| Expressive | 10 |
| Receptive | 9 |
| Pragmatic (Social) | 6 |

##### Expressive

Most caregivers of children with BBSOAS discussed expressive communication concepts. Requesting was a communication concept mentioned by most caregivers (Table S5).

Table S5. Expressive communication concepts on the ORCA measure mentioned by caregivers of individuals with BBSOAS.

| Expressive Communication Function | *n* (total *n*=11) |
| --- | --- |
| Seeking Attention | 2 |
| Directing Attention | 1 |
| Refusing an Object | 7 |
| Requesting an Object | 10 |
| Requesting an Object out of View | 2 |
| Requesting More | 2 |
| Asking Questions | 2 |
| Telling Stories | 3 |

Two caregivers of children with BBSOAS discussed how their children **seek attention**. Both caregivers shared that their children used sounds to seek attention. For example, one caregiver (PI:1004) said, “*So, if she wants to go outside, she will continue to be loud and just really “Ahh” until she gets your attention to where you’re gonna respond to her*.” The other caregiver (PI:1006) said their child might use gestures and sounds together to seek attention. For example, they said, “*Or, she’ll kinda point to herself if she wants something and kinda do that “Ah! Ah!”*

One caregiver (PI:1004) also said their child could seek attention in a number of ways other than just using sounds. Some other examples included:

- “*Socially, she’ll kind of – she enjoys being around people. So, she’ll typically go up to people and she may kinda touch you by the hand or take your hand [to get your attention].”*
- “*Typically, she will make eye contact [to seek attention] if – say for instance, if her brother’s around – she has a two-year-old brother – and he’s playing with his trucks or something, she may look at him because she likes – she enjoys watching him play.*”
- “*Or if it’s with me and I’m offering her a bite to eat, if she really wants it, sometimes she’ll kinda hit me if I’m not going fast enough almost as to say “Hello? I’m waiting for the next bite, and you’re not paying attention.” So – but those moments are rare*.”

One caregiver (PI:1011) said their child uses verbal words, as well as physical body movements, to **direct the caregiver’s attention** to something. For example, they said, “*Like we went to the library… and he’s like, “Look at this book.” He’s obsessed with books and he’s like, “Look at this book I got. Look*.”

Seven caregivers discussed how their child communicated **refusal**. Three caregivers described how their children used words or word approximations to communicate refusal. For example, one caregiver (PI:1011) described how their child says, *“I don’t want to. No,”* when they are refusing something. Another caregiver described a typical exchange where their child refuses an item. For example, the caregiver (PI:1001) said, *“And then like, if she says, ‘I want a snack,’ I can say, ‘Do you want crackers?’ and she’ll say, ‘No, I want cookies,” or she’ll say, ‘No, cookies’”.*

One caregiver (PI:1010) described how their child used gestures/signs to communicate refusal and explained that “*All Done”* is one of the signs that the child knows to indicate refusal. Two caregivers explained how their child will make sounds to refuse something. For example, one caregiver (PI:1008) said, “*When she’s communicating things that she doesn’t like, which she might kinda make a frustrated…grunt or moan.”*

When describing refusal, five caregivers described communication behaviors that were not symbolic, but were interpreted by the caregivers as indicating refusal. For example, one caregiver (PI:1006) described how their child will “*turn her head”* or *“won’t open her mouth”* when trying to brush her teeth. This caregiver also said their child indicates refusal by “*screaming and crying*” (e.g., crying/fussing).

Ten out of the 11 caregivers discussed how their children communicated **requests** during concept elicitation. Almost all caregivers indicated that their child utilized symbolic communication to request (e.g. gestures, signs, words, word approximations, sounds), although the specific modality differed between children. Some of the caregivers also reported other non-symbolic communication behaviors that they interpreted as requesting. One caregiver (PI:1008) only described these types of behaviors. The most commonly requested objects were food and drink, but individuals in this sample also requested TV and places/activities (i.e. outside). Three caregivers (PI:1001, PI:1002 and PI:1011) gave examples of their child self-referencing while requesting.

Five caregivers said their child used words/word approximations to request. For example, one caregiver (PI:1001) said, “*She can communicate quite a bit, but she really can’t piece together four- and five- – word sentences. I mean, sometimes they’ll sporadically come out of her, and they’re appropriate, but it’s a lot of – maybe one- to three- word – kinda back and forth conversationally asking for things. And then like, if she says, “I want a snack,” I can say, “Do you want crackers?” and she’ll say, “No, I want cookies,” or she’ll say, “No, cookies.” Something like that*.” Another caregiver (PI:1006) said, “*So, her brother – she loves her brother. She loves to go in his room. So, she may say, “Room!” and I’ll know exactly what she’s talking about. Or, he has a PlayStation. So, she may say, “’Troller!” for “controller”. And so, “Milk!” if she wants her milk*.”

Three caregivers described gestures/signs (ASL sign, finger point, modified gesture/sign) their child uses to request. For example, one caregiver (PI:1006) said, “*She’ll do this [hand gesture] to her mouth and sort of guide me to the kitchen if she’s hungry*.” Another caregiver (PI:1010) said, “*He’s nonverbal and he’s very limited in his signs, so it’s mostly he knows more, all done, water, and please. That’s usually what he communicates about. Otherwise, it’s more just like pointing. If he wants a snack, he’ll point*.” One caregiver (PI:1009) described how their child uses sounds to request. For example, they said, *“Like, she has a screeching sounds when she’s angry and wants the door open or the [TV] show to change.”*

When describing requesting many parents described communication behaviors that were not symbolic, but were interpreted by the caregivers as requesting. For example, parents described how their child might use physical gestures to request. Example quotes included:

- “*It’s the same thing with going outdoors. She just touches the doorknob. She goes by the door – or if she wants to go for a ride in her wheelchair, she goes stands by the wheelchair. And she kinda gestures to pick her up to sit in the wheelchair*.” – PI:1004
- “*Part of it is just she’ll go sit in front of the TV. She also will often grab the remote. We usually put it in a drawer to prevent that, but if it’s out, she’ll find it, and grab it, and sometimes bring it to us, but more often, just start pushing buttons..*.” – PI:1008
- “*If she’s hungry, she physically brings us the bottle or, like I said, she lays down ready to get it. So, it’s more so just it’s physical*…” – PI:1009
- “…*or he’ll go to the refrigerator and like bring me the watermelon, or bring me something that he can’t open*.” – PI:1011

Two caregivers discussed how their child **requested more of something** using gestures/signs. For example, one caregiver (PI:1004) said, “*So, when I feed her, to get her to ask for more, I will kinda take the bowl away from her and then say “If you want more, you need to touch the bowl, ask for it.” And then, eventually, I will just kinda leave it there. And then I started noticing she would just tap on the bowl or tap on her thermos when she wants more to eat*.” The other caregiver (PI:1010) said their child uses the ASL sign for “more”.

Two caregivers (PI:1001 & PI:1011) discussed how their child **requested objects that they could not see** using symbolic communication (e.g., words). For example, one caregiver (PI:1011) said, *“A lot of times, he’ll say – he loves Raisin Bran and he’s like, “Bread.”. When he’s doing something – like if he is on his iPad or something, he’ll say, “Bread. Bread. Bread.” I’m like, “[child’s name], full sentence.” He’ll be like, “May I have bread, please?” he knows how to do it. He just – if he’s busy doing – like playing with an iPad and stuff, he’ll use these one-word demands…”*

Two caregivers described how their children **asked questions**. One caregiver (PI:1006) shared that they interpreted some of the child’s words as questions. For example, they said, “*Does she ever ask where her brother is?” “No, she’ll say ‘room,” and I’ll say “[Name] not here,” “[Name]’s at school,” or “[Name]’s asleep still,” but it’s not really in a question form. Maybe it’s a question to her, but it’s kinda...I mean she just will say like one word, so it’s kinda hard to tell if that’s a question or not.”*

Three caregivers spoke about their children **telling stories**. Example quotes included:

- *“She’ll come home from school, and she’ll say something sporadically like “sweater,” and it’s like, “Okay, what is she saying that about?” Well, then, I find out the next day that they’re supposed to wear Christmas sweaters to school. Well, she can’t piece all that together, you know what I mean? Or, one day, she was adamant about wearing a hockey jersey, and we just could not figure out why. Well, it was hockey week during PE, but she couldn’t tell us all that. She just knew that she wanted to participate in some way with that hockey jersey. So, it’s like we get bits and pieces, but we don’t get the full picture at all” -*PI:1001
- “*If I ask her, she will-because when I pick her up from school every day, I say “Did you have a good day? What did you do? And she will typically name her friends that maybe she saw that day. And so I’ll ask, “Did you play outside?” and she’ll say yes. Sometimes, of course, she’ll say no, and it’s in the right context again when they really did go outside. But usually, it’s the people she saw, not the things that she did.”* -PI:1006

##### Receptive

Nine of the eleven caregivers mentioned aspects of receptive communication. For this group, concepts included following familiar one-step directions (*n=*4), making choices (*n=*4), following familiar two-step directions (*n=*3), understanding isolated words or phrases (*n=*1), responding to simple questions (*n=*1), and listening/asking appropriate follow-up questions (*n=*1).

For following directions, one caregiver (PI:1011) said, *“If you say, “[Child’s name], stop doing that,” she’ll stop. Or, if you say, “Can you grab the milk out of the fridge?” she’ll grab the milk. So, I mean, her receptive language is pretty spectacular.*” Another caregiver (PI:1007) gave an example of how they know their child understands their communication, saying, “*She’ll either follow through with what you ask of her or she’ll respond in an appropriate manner. So, if you tell her that you love her, she’ll give you a hug, or she’ll smile, or something like that*.”

In terms of responding to questions, one caregiver (PI:1002) spoke about the child’s ability to respond to simple questions (yes/no questions) with mixed results in terms of more complex questions, “*So, if I ask her, “Did you read a book at school today” – she will tell me, “Yeah.” And I’ll say, “What was the story about?” And I think she just tells me something random because there have been a couple times where the teacher was like, “No, we absolutely did not read that. We read something about a mouse.*”

Another caregiver (PI:1011) discussed a skill that that has not been frequently identified across the qualitative interviews. They said, *“I talk to him about something. He like really listens and he asks appropriate questions. And he really is always listening, even when you think he’s not listening, because then, he’ll ask, later on, “What did you say about this? “You know, what did you say about this, so I think his receptive s probably better than expressive.”* This example also showcases an advanced expressive skill, in combination with receptive language skills.

##### Pragmatic

Six caregivers discussed aspects of pragmatic (social) communication. Specific concepts included greeting people (words *n=*2, waving *n=*2), social smiling (*n=*2), using names for people or themselves (*n=*2), initiating games (*n=*1), and pretend play (*n=*1).

In terms of using names, one caregiver (PI:1001) said, *“She sees her brother talking, having conversations with us, and she kinda looks at him and notices that – you know. And then, she’ll start kinda babbling, or she’ll say her father or my name over, and over, and over again, and we think that’s her way of having a conversation, in a way -of having that back and forth.”* Another caregiver (PI:1011) said, *“Now, these new people pull up and there’s a lot of people, because they’re newlyweds and I guess family’s helping them move in and stuff, and he’ll say “Hey [name]. Look at me. Look at me. Look how I ride my scooter so fast.”*

One caregiver (PI:1010) discussed how their child initiates a game, saying, “*I mean, and again, he’ll play – he plays peek-a-boo. He’ll engage in that. He’ll start it. He’ll cover his eyes and play peek-a-boo with you.*” Another caregiver (PI:1011) described the pretend play their child engages in, “*He’ll tell his dad that they have a band and it’s called [name] and he’s gonna play this, and it’s only the bros, and the girls can’t be part of it, and now, we have a new neighbor that moved across the street and he asks him, “Do you want to be part of our bro band?*”

#### Changes in Communication

The research team interviewed 11 caregivers whose children had BBSOAS and all 11 discussed how their child’s communication changed overtime.

Seven out of 11 caregivers spent the majority of the time describing how their child developed verbal speech. Six out of the seven caregivers said their child began using words around age 3. For example, one caregiver (PI:1002) said, “*And that was something that I was told is common for kids who are diving into the verbal world that have her condition, was that around preschool is when they start blossoming. And that was definitely – she started preschool this year. And that’s definitely where we’re at is the blossom stage... One day – and she’s always been like that with everything. So, she will not be able to do something forever and then all of a sudden one day she can*.” Another caregiver (PI:1003) said, “*And for speech everything was initially pretty slow with any of the skills, and then something just seemed to click in her and she would take off with it*.” Five caregivers said the changes occurred when the child began attending pre-school/kindergarten and/or speech therapy. Prior to gaining verbal speech, a couple of caregivers (*n=*2) said their child was reliant on American Sign Language to communicate; however, once their child began using verbal speech, the child stopped using their ASL signs.

Caregivers described their child relying on a few words initially and then increasing vocabulary and stringing words together into sentences overtime. For example, one caregiver (PI:1007) shared, “*For a long time, she was only saying maybe three words. And then around age four, we got maybe to about five words. And then now at age five, she’s saying about nine words that are legible… The past year though, I would say that she went from speaking very limited amount of words to she is now putting three and four words together almost to form a sentence*.” Another caregiver (PI:1006) said, “*I mean, she had little to no words. She now has over 100 words. I mean, she’s putting some two and three words together. She has a few things that she’ll put two words together, and just recently, with probably in the past couple of weeks, she has got one phrase that’s three words, which is ”I want some.” And so, that’s her most recent. But really, within probably the past year, her language has really improved*.”

Several caregivers said their child is currently working on improving apraxia, pronunciation, annunciation and “intelligibility” in speech therapy. For example, one caregiver (PI:1005) said, “*Now, it’s just she’s limited by a little bit of the apraxia of speech, which impacts her intelligibility. And, we think she might have a little bit of a dysarthria piece going on, as well. Maybe some just general weakness that’s impacting her intelligibility. So, her speech therapy now focuses on not distorting vowels in a word, and working on blends, and keeping her lips taut enough to say a sound*.”

Four caregivers said their child had no verbal words. While discussing changes over time, these caregivers described improvements in their child’s gestures and signs. For example, one caregiver (PI:1008) said, “*So, I would say the noises have always been there – especially crying and laughing – since a baby. But as far as kinda the more intentional – like bringing the iPad…actually wanting something from someone – that’s new. That’s probably within the last two years*.” Another caregiver (PI:1009) said, “*Before, to turn the channel, she would just sit there and scream. But now, she will actively, physically search for the remote and bring it to us. Or…she’ll bring her bottle to us so she can eat. And before, it wasn’t that. It was more of us prompting all of that*.” Like the caregivers of children with verbal speech, these caregivers also said that learning communication skills takes time, as well as repetition and modeling/prompting. For example, one caregiver (PI:1004) said, “*…it takes her a very long time to learn a skill…Like just the – just trying to teach her to ask for more…this had been going on for years. I try to get her to self-feed, but she just wasn’t really picking it up. So, when I feed her, to get her to ask for more, I will kinda take the bowl away from her and then say “If you want more, you need to touch the bowl, ask for it.”…And then I started noticing she would just tap on the bowl or tap on her thermos when she wants more to eat*.”

Two caregivers described their child’s inability to tax two systems at the same time during skill acquisition. For example, one caregiver (PI:1006) said, “*So, when she learned how to walk which was around a year ago language was…not – I mean, we were trying to learn new words and trying to get her to do new things, and it just was not happening…if there’s one thing she’s focusing on, the other areas sort of fall behind or become stagnant*.” The other caregiver (PI:1009) said, “*So, it’s a mixture of things – of she became much better at physically moving, and her walking is improving and all these things, and then, we see tiny little regressions in the background of things…She figured out how to walk, she can run, she can do all these things unassisted, and then, feeding just took a 180, and we’re back to full bottle-fed or using her G-Tube because it took too much out of her to learn the one big thing, so the other thing went to the sideline*.” This caregiver also spoke about a regression in their child’s communication, saying, “*So, it’s kind of regressed and improved. She used to do a lot more babbling and things like that – and repeating certain sounds. So, from like three on, that kind of went away. She went from like 15 sounds to like four*.”

Three caregivers (PI:1002, PI:1007, PI:1010) of young children (ages 4 -5) said words/sounds can be fleeting and may disappear and reappear. For example, one of the caregivers (PI:1002) said, “*Sometimes she’ll put a pause on the things she knows. And so, she’ll lose a word and gain a word. And then once she’s mastered the new word, then we’ll see the old ones brought back*.” Another caregiver (PI:1007) said, “*It’s just maybe three words that she had once learned how to say, she no longer says*.” PI:1010 said, “*…we don’t hear sounds consistently. I’ve heard him say mamama, but then, I won’t hear it for months*.”

#### Hearing & Vision Impacts

Four out of 11 caregivers reported that their child had a hearing impairment; however, only one of these caregivers (PI:1003) reported a formal diagnosis (conductive hearing loss in the right ear). The caregiver said the hearing loss impacts their child’s receptive communication, but not their expressive. For example, they said, “*Not for her to communicate, but for the receptive line of it for sure, but on the other side of it I don't think it [hearing impairment] has an impact on her speech or anything*.” Two caregivers (PI:1006 & PI:1007) mentioned their child may have a hard time hearing certain pitches, but neither child had been formally diagnosed with a hearing impairment, and neither caregiver felt the hearing issue impacted their child’s ability to communicate. One caregiver (PI:1011) felt their child was sensitive to sounds which could lead to the child feeling overwhelmed, especially in new environments. For example, the caregiver said, “*The microwave went off and you could see that he was like – like it just like broke his train of thought and I had to bring him back, but I knew it was the sound. Little sounds throw him off. Especially, if we’re at school or we’re at a different place, or someone’s walking by, he’s very distracted by things…Even when the AC kicks in, he’s like, “What’s that?” he knows it’s the AC, but in – if he’s class or in a new place, like he’s – sounds that we tend to tune out, he just – he’s very in tuned to every little noise. That’s very distracting for him with communication*.”

Ten out of 11 caregivers reported that their child had a vision impairment (Table S6). Many caregivers reported multiple vision impairments, with five mentioning optic atrophy, a common component of BBSOAS. Other common impairments mentioned by caregivers included cortical visual impairment (CVI; *n=*4 confirmed, 2 suspected), and farsightedness (*n=*3).

Table S6. Caregiver reported vision impairments for their children with BBSOAS.

|  | Freq. | Participant IDs |
| --- | --- | --- |
| Not specified by caregiver | 1/11 | 1006 |
| Wears glasses | 4/11 | 1002, 1003, 1005, 1007 |
| Peripheral blindness | 1/11 | 1007 |
| Legally blind | 1/11 | 1001 |
| Nystagmus | 1/11 | 1002 |
| Optic nerve hypoplasia | 2/11 | 1002, 1009 |
| Esotropia | 2/11 | 1002, 1005 |
| Cortical Vision Impairment | 4/11 | 1004, 1007, 1009, 1011  [1002, 1008^1^] |
| Nearsightedness | 1/11 | 1002 |
| Optic atrophy | 5/11 | 1002, 1003, 1004, 1007, 1009 |
| Farsightedness | 3/11 | 1003, 1007, 1009 |
| Astigmatism | 1/11 | 1003 |
| Strabismus | 1/11 | 1009 |

^1^For participants 1002 and 1008, parents reported suspected CVI,

but their child had not received a formal diagnosis.

Caregivers tended to discuss impacts on their child’s gross motor abilities or depth perception/spatial awareness rather than impacts on communication. For example, one caregiver (PI:1004) said, “*So, she does walk. But she doesn’t walk long distances. So, she may walk a few feet, and then she’s all of the sudden – she just sits down for whatever reason or she falls... And then, as far as depth perception, she has to really try to test something out first. If she’s going on the patio and she has to step down, it’s kinda a hesitation to it, a very long hesitation to it. And you have to kinda guide her because she doesn’t trust that she’s gonna land where she needs to land*.” Another caregiver (PI:1008) said, “*So, it does seem like when she’s walking, her field of vision, especially near her feet, is… She has trouble seeing things on the ground. Sometimes, it seems like even when she’s very interested in something, she kind of looks away. She may be looking out of the corner of her eye at some of these things*…”

Two caregivers said their child’s CVI can cause them to become overwhelmed, especially in new environments. For example, one caregiver (PI:1009) said, “*So, one of the main things is because of her visual impairment, she needs a routine. So, she knows her specific routes to places – she knows how to get from one room to another room. But if we’re in a different place, she gets herself lost. She’ll walk in a circle and then just kinda sit down because she doesn’t know what to do from there – she doesn’t know where to go from there. So, it’s mainly newer environments and newer things that she hasn’t comprehended enough to know what’s going on. So, she’s very limited to the here and now versus new things. So, she doesn’t know how to express herself when it comes to new things of frustration or anything like that – or needing something. She kind of just will just sit down and just tune the world out*.” The other caregiver (PI:1011) said, “*But I feel like his vision takes over everything. He has a hard time speaking through that. Especially, if there’s traffic. Even if he’s on the sidewalk and there’s cars coming his way, he buries his head. He can’t – I ask him like, “Are you okay? Are you okay? Do you want to sit down? Are you okay?” Like he can’t speak, so I feel like that always gets priority, like to process what he’s seeing is always the most difficult for him*.” This caregiver also felt their child’s CVI impacted their social communication, saying, “*He’s very social, but in novel places – a lot of things – it looks like typical autism, especially in novel places. He can’t make out faces, so he’s always looking at your shoes to try to memorize who you are. He looks down when he meets you and that looks like freight, no eye contact. … I think, especially for social communication, I think it impacts him significantly, aside from he doesn’t get social cues. People will say – like put hand up for high-five. He doesn’t see it. You know, like they have to say – or a handshake. He doesn’t see it. I think it definitely impacts, you know, relationships and stuff*.”

#### Meaningful Change

All caregivers were asked, “*What would a meaningful change in communication look like for your child*?” And time permitting, most caregivers were asked “*If your child’s communication skills were to decline or get worse, which communication skills would be most important for your child to retain*?”

Parents generally interpreted “meaningful change” to mean “improvements” in communication ability or skills. During this question, some parents made the distinction of realistic or not realistic goals they had for their child. Five caregivers mentioned that they would like their children to communicate their preferences and feelings, with three caregivers responding that they would like improved AAC device usage (all themes in Table S7).

Participant 1007 stated *“Just for her to be able to communicate what she wants. I don’t care how she communicates. So many of her frustrations and her behaviors come from her inability to be able to tell us how she’s feeling: if she’s hurt, or if she’s upset about something, or frustrated. And so, increasing her ability to use all the technology and skills to communicate whether it’s verbally, or through a tablet, or sign language, would just be a huge improvement. Because you can tell how frustrated she gets when she can’t communicate with you.”*

Participant 1008 stated, *“I think the most realistic kind of next step would be probably her learning to use the augmentative communication device on her iPad. Obviously, any words would be huge. But it doesn’t seem like that’s coming any time soon…I think [AAC device usage] would be gradual. Sometimes, it seems like she really gets the “more” and “all done” piece [on the device], but not all the time. So, even progressing to where it’s clear she knows what she’s trying to tell you and she’s doing that consistently, that would be a big improvement.”*

Table S7. Responses to “*What would a meaningful change in communication look like for your child*?” from caregivers of children with BBSOAS.

| Meaningful Improvement | 1001 | 1002 | 1003 | 1004 | 1005 | 1006 | 1007 | 1008 | 1009 | 1010 | 1011 | Total |
| --- | --- | --- | --- | --- | --- | --- | --- | --- | --- | --- | --- | --- |
| Improved communication to reduce frustration (when other people do not understand) | 1 |  |  |  |  |  | 1 |  |  |  |  | 2 |
| Communicate about parts of the day | 1 |  |  |  |  |  |  |  |  |  |  | 1 |
| Participate in a back-and-forth conversation |  | 1 |  |  |  |  |  |  |  |  | 1 | 2 |
| Improved AAC device usage |  |  |  | 1 |  |  |  | 1 | 1 |  |  | 3 |
| Faster cognitive and visual processing |  |  | 1 |  |  |  |  |  |  |  |  | 1 |
| Improve verbal pronunciation |  |  |  |  | 1 |  |  |  |  |  |  | 1 |
| More words using verbal speech |  |  |  |  |  | 1 |  |  |  |  |  | 1 |
| Communicate preferences/feelings using any modality (e.g., words, AAC) |  |  |  |  |  | 1 | 1 | 1 |  | 1 | 1 | 5 |
| Start using verbal words |  |  |  |  |  |  |  | 1 |  |  |  | 1 |

In terms of skills that would be important to retain if skills were being lost, the four caregivers who were asked this question mentioned gestures/body movements (*n=*2), verbalizing feelings (*n=*1), pointing (*n=*1), and AAC device usage (*n=*1). One participant (PI:1009) described the hypothetical desire to keep body movements if communication were to decline, “*The physical communication signs, because those are really what we work on right now. So, those would be a lot more important just because we know the basics of that, and pretty much anybody in our family that sees her – we can all figure it out by what she’s physically bringing us or going. So, that’s a pretty basic form of communication, but it’s something that she’s definitely improved on and something that could be used for the long term*.”

### GRIN2B-related neurodevelopmental disorder

#### Demographics

Eight caregivers of individuals with GRIN2B-related neurodevelopmental disorder (GRIN2B) participated. Most caregivers were female and on average 37.6 years of age (Table S8). No caregivers self-identified as Hispanic-Latino. Five caregivers identified as white, two as Asian, and one as African-American or Black. All caregivers reported that they were currently married or living with a domestic partner. Overall, the participants reported high levels of education, employment, and annual income (Table S8).

Table S8. Demographic information for eight caregivers of children with GRIN2B-related neurodevelopmental disorder.

| *Caregivers* | *n (%)* |
| --- | --- |
| Female | 6(75.0) |
| Age, years (Mean/SD) | 37.6/5.4 |
| Ethnicity |  |
| Not Hispanic or Latino | 8(100.0) |
| Hispanic-Latino | 0 |
| Race |  |
| White | 5(62.5) |
| African-American or Black | 1(12.5) |
| American Indian/Alaska Native | 0 |
| Asian | 2(25.0) |
| Middle Eastern | 0 |
| Native Hawaiian/Other Pacific Islander | 0 |
| More than one race | 0 |
| Relationship status |  |
| Single, never married | 0 |
| Married, or living with domestic partner | 8(100.0) |
| Separated | 0 |
| Divorced | 0 |
| Widowed | 0 |
| Highest grade in school |  |
| Less than high school diploma | 0 |
| High school degree or equivalent | 2(25.0) |
| Some college/University | 3(37.5) |
| College/University degree | 2(25.0) |
| Postgraduate degree | 1(12.5) |
| Occupational status |  |
| Homemaker | 2(25.0) |
| Unemployed | 1(12.5) |
| Retired | 0 |
| On disability | 0 |
| On leave of absence | 0 |
| Full-time employed | 5(62.5) |
| Part-time employed | 0 |
| Full-time student only | 0 |
| Income of U.S. residents |  |
| Less than $20,000 | 0 |
| Between $20,001 and $40,000 | 1(12.5) |
| Between $40,001 and $60,000 | 2(25.0) |
| Between $60,001 and $80,000 | 2(25.0) |
| Between $80,001 and $100,000 | 0 |
| Between $100,001 and $250,000 | 2(25.0) |
| Between $250,001 and $500,000 | 1(12.5) |
| $500,000+ | 0 |
| I prefer not to answer | 0 |
| I do not know | 0 |
| Relation to child |  |
| Mother/Step-mother | 6(75.0) |
| Father/Step-father | 2(25.0) |

Caregivers also provided demographic information for their child with GRIN2B. Five children were female, with similar rates to caregivers in regards to ethnicity and race (Table S9). In terms of genotype, six were considered missense and two considered deletion.

One caregiver reported that their child had an ASD diagnosis, and three reported that their child had epilepsy. Most children were receiving therapeutic services including physical, occupational, and speech therapy (Table S9). Three caregivers reported that their child used a high-tech AAC device. The settings in which the device was used varied (Table S9).

When asked about their child’s verbal language, two caregivers indicated their child used ‘full sentences’, two caregivers indicated that their child used ‘short phrases’, none indicated their child used a ‘few words’, and four indicated their child used ‘no words’.

Table S9. Demographic information for eight children with GRIN2B-related neurodevelopmental disorder.

| *Children* | *n (%)* |
| --- | --- |
| Age, years (Mean/SD) | 7.8/4.8 |
| Child gender Female | 5(62.5) |
| Ethnicity |  |
| Not Hispanic or Latino | 8(100.0) |
| Hispanic-Latino | 0 |
| Race |  |
| White | 5(62.5) |
| African-American or Black | 0 |
| American Indian/Alaska Native | 0 |
| Asian | 1(12.5) |
| Middle Eastern | 0 |
| Native Hawaiian/Other Pacific Islander | 0 |
| More than one race* | 2(25.0) |
| Child’s genotype |  |
| Deletion | 2(25.0) |
| Missense | 6(75.0) |
| Autism Spectrum Disorder (ASD) | 1(12.5) |
| Epilepsy | 3(37.5) |
| Low Muscle Tone | 8(100.0) |
| Types of therapy |  |
| Physical Therapy | 6(75.0) |
| Occupational Therapy | 6(75.0) |
| Speech Therapy | 5(62.5) |
| Other therapy  ABA Therapy  Developmental Therapy  Hippo Therapy  Massage Therapy  Music Therapy | 1(12.5)  1(12.5)  1(12.5)  1(12.5)  2(25.0) |
| Age first introduced to device (Mean/SD) | 6.7/2.1 |
| AAC device |  |
| No | 5(62.5) |
| Yes | 3(37.5) |
| Device |  |
| High tech | 3(37.5) |
| Low tech | 0 |
| Places to use the device |  |
| Home | 1(12.5) |
| School | 3(37.5) |
| Out in the community | 0 |
| Other | 0 |

Note: *White and Asian (n=1), Asian and African-American or Black (n=1)

#### Typical Communication Ability

The results for this section are organized by behaviors that correspond to expressive, receptive, and pragmatic (i.e. social) communication (Table S10), aligning with the conceptual framework that was developed for individuals with Angelman syndrome.^1^ Please note, although counts are reported to indicate salience of each theme, these data are limited in that they do not indicate *all* communication behaviors relevant to each child, only the ones that caregivers discussed during the interview.

Table S10. Number of caregivers that mentioned one or more examples of expressive, receptive, and/or pragmatic communication.

| Communication Behaviors | *n* (total *n* = 8) |
| --- | --- |
| Expressive | 7 |
| Receptive | 7 |
| Pragmatic (Social) | 6 |

##### Expressive

Most caregivers of children with GRIN2B discussed expressive communication concepts. Requesting was a communication concept mentioned by the majority of caregivers (Table S11).

Table S11. Expressive communication concepts on the ORCA measure mentioned by caregivers of individuals with GRIN2B-related neurodevelopment disorder.

| Expressive Communication Function | *n* (total *n* = 8) |
| --- | --- |
| Directing Attention | 1 |
| Refusing an Object | 4 |
| Requesting an Object | 6 |
| Requesting More | 3 |
| Telling Stories | 1 |

One caregiver (PI:1108) said their child used physical body movements to **direct their attention to something**. For example, they said, “…*she can tap on you, and try to lead you towards what she wants*.”

Three caregivers discussed how children communicated **refusal**. All three caregivers described words or word approximations that their child uses to indicate refusal. For example, one caregiver (PI:1106) described how her child will say and sign ‘all done’ at the same time: *“she’ll say “all done,” but then she’ll sign it too.”*  Another caregiver (PI:1108) explained that one of the words her child typically uses is “*Stop.”* Two caregivers also indicated that their children use non-symbolic forms of communication to indicate refusal. For example, one caregiver (PI:1109) said, “*it’s either a very stiff arm like he doesn’t want you to – like especially if you’re trying to feed him more and he’s full, he’s like no, no, no or he just throws it at you. Drops it, throws it, you know, I’m done with this kind of thing.”*

Six caregivers discussed how their children communicated **requests**. All caregivers provided examples of how their child requested using symbolic communication. The most commonly requested objects were food and drink, but individuals with GRIN2B in our sample also frequently requested TV, places, and activities (e.g., outside). Three caregivers (PI:1104, PI:1106 & PI:1108) gave examples of their child self-referencing while requesting.

Four caregivers said their child uses words/word approximations to request. For example, one caregiver (PI:1104) said, “*He always asks for French toast. I want French toast. Well, we don’t have French toast. Do you want something else? And okay, I’ll have oatmeal. Okay. And thank you, Mommy*.” Another caregiver (PI:1106) said, “*If she wants water to drink, she’ll say, “water”. A lot of your basic stuff. Like if she wants to take a bath, she’ll say, “bath”, “bathtub”.”*

One caregiver (PI:1101) described their child using a modified gesture/sign to request. For example, they said, “*Well recently and this is a new thing, it's happened in the last month, I always sign the word eat to him, and recently when he's getting hungry, he'll put his hand up to his mouth, like the eat motion, and then he'll kick his right leg to let us know that he's hungry*.” Another caregiver (PI:1105) provided an example of how their child pairs multiple modalities (gestures/signs and sounds) together to request. For example, they said, “*Yeah, for example when she’s hungry she does make an M sound… So, if we pick her up, we bring her into the kitchen, and we would ask her if she’s hungry, sometimes she would, sometimes she wouldn’t but she opens up her mouth and she looks straight at the refrigerator and [sic] makes the um sound, so like she’s eating like, “Um*.”

Three caregivers discussed how their child requests more of something using words or gestures/signs (hand over hand gesture or ASL). For example, one caregiver (PI:1106) said, “*In the beginning, we used to use sign language for “more” and “all done”. I guess she will occasionally still use “more”, but she’ll [verbally] say “more”, and she’ll say “all done”, but then she’ll sign it too*.” Another caregiver (PI:1109) said, “*He will do some hand-over-hand. And sometimes we’ll grab his hands and he’ll do this. We know he wants more. We’re trying to kinda get that – we’ve been working on more since he was little, little, little. But – so, sometimes he’ll grab my hands and do it with my hands like “I wanna do it again*.”

##### Receptive

Seven of the caregivers in this group mentioned receptive communication concepts. Skills included following familiar one-step directions (*n=*4), making choices (*n=*4), responding to simple questions (*n=*3), turning towards their name (*n=*1), turning towards the sound of someone talking (*n=*1), understanding isolated words/phrases (*n=*1), and following novel one-step directions (*n=*1).

Four participants discussed how their child was able to **follow directions**. One caregiver (PI:1103) gave the example, “*I can’t give her multi-step directions. But if I would say stop or give me the crackers or put your seatbelt on, or whatever, put your hat on. She can understand those directions.*”

Two caregivers (PI:1103 & PI:1106) described how their child **makes choices** using words or word approximations. For example, one caregiver (PI:1106) said, *“If we give her options, like, “Do you want bathtub or shower?” she’ll say which one she wants to do.”* The other caregiver (PI:1103) said, *“So, like I mean she might just say I don’t know, like if it’s a toy or a food she might [verbally] say I choose Elsa or I choose Moana.”* Another caregiver (PI:1109) reported that their child uses an AAC device to make choices. For example, they said, “*Or if it’s things that are on his iPad, then he gets – I mean, I know I just kinda showed you the button. But for this, when the music therapist comes, he has options of “What do you want me to play?” And he’ll pick.*” The same caregiver shared that their child could also use non-symbolic communication (grabbing) to make choices, saying, “*…sometimes if I hold up two yogurts, he’ll choose which yogurt he wants, or if I say “Do you want potatoes, or do you want macaroni today,” he’ll pick one kind of thing.* […] *He will actually grab it. He’ll choose it.*”

Early receptive skills included **turning towards voices/sounds and turning towards their name**. One caregiver (PI:1102) said, “*If you’re talking to her, yeah, she might turn her head. She doesn’t always turn her head. She does like more of the high pitch sounds compared to regular sounds. Her little sister does good with her. Her mom can be better than I do with her as far as getting her attention. But she’ll turn some. But she doesn’t always turn.”* Another caregiver (PI:1105) said, “*I mean whenever I call her, she would follow me with her vision.”*

One caregiver (PI:1106) mentioned their child could **understand isolated words/phrases** and gave the following example, “*Yeah, she does, if she knows that word. So, if we talk about the zoo, then she’ll all of a sudden be like, “Zoo? Go zoo. Go zoo. Let’s go.”*

One caregiver (PI:1103) spoke to their child’s capacity for correction, “*So, I mean she’ll call [her teacher], Ms. [rhyming word]. Or she’ll call Ms. [second teacher name], Ms. [rhyming word]. But it’s so cute but then people facilitate. I feel like if you really were to correct her, she would hear the difference and be able to fix it.”*

##### Pragmatic

Six caregivers spoke about pragmatic (social) communication they observe in their child. Specific skills included using names (*n=*3), greeting using words (*n=*2), recognizing mood (*n=*2), turn taking (*n=*2), changing language according to the situation (*n=*1), pretend play (*n=*1), and social smiling (*n=*1).

In terms of **using names**, one caregiver (PI:1103) reported that their child can reference their teachers. Another caregiver (PI:1106) said, “*I say my nephew’s name, she’ll say, “See [name]. See [name].” “So, she likes to socialize, and so I guess those are the few ways that she will try to communicate her desire to play together. But yeah, it’s usually like, “Come on, everybody. Let’s go.” She’ll ask [name] “Come on, [name]. [Name], sit down.”*

Another caregiver (PI:1103) spoke about their child **recognizing mood**. They said, “*I mean she’s happy for her friends when they do something good. You know, she’s excited, you know she gives people hugs, or goes to them when their sad.*”

Other receptive communication examples included:

- “*So, most people, if they’re making a mistake in their language, will make the same mistake every time. But he doesn’t. And he kind of adapts it to the individual in a way that each individual can understand him. So, if saying it one way doesn’t work, he’ll try and say it a different way, until he’s understood. And he’ll add in gestures to help with that. He doesn’t usually use the AAC with his peers. We’re trying to have him use language first*.” - PI:1104
- “*So, the new word that she knows is “[child’s name] turn.” So, her and her brother, we have this roller coaster toy, and so we’re like, “Okay, it’s [sibling’s name] turn.” And she’s learning to wait. But then as soon as the car comes back, she’s like, “[child’s name] turn. [Child’s name] turn.” So, she’s starting to understand turn-taking, but she, I think, wouldn’t necessarily be great at it without our help*.” – PI:1106

One caregiver (PI:1106) shared how their child engages in **pretend play**, “*Yeah, some pretend play because she loves playing with her people figurines, so she’ll sometimes have them sleep or have them eat, have them fall. Other than figurine pretend play, I think it doesn’t really go beyond the dolls, eating and sleeping and walking around or something like that.*”

#### Changes in Communication

The research team interviewed eight caregivers whose children had GRIN2B-related neurodevelopmental disorder and all eight discussed how their child’s communication changed overtime.

Three out of 8 caregivers spent the majority of the time describing how their child developed verbal speech. All three caregivers said their child began using words around age 1. Prior to gaining verbal speech, all three said their child was reliant on American Sign Language (ASL) to communicate; however, once their child began using verbal speech, the child stopped using their ASL signs. One of the caregivers (PI:1103) said their child would string an ASL sign and word together to communicate a message prior to combining words together. For example, they said, “*So, or like she might do the sign for I want. Mumble something that was like that I knew meant myself or my husband or something. Or up. Like it might be “uh, uh” and point to herself so it was like that adding things together before she could truly say words together if that made sense*.”

One caregiver (PI:1106) said they saw increases in vocabulary when their child began different medication regimens. For example, they said, “*…when we started giving her the B12 injections around 1 1/2 months for three to four months, we saw something new every single day, whether it be a new word, new gesture, just something new that we didn’t know that she could do. So, that was probably the beginning of improvement that we saw in her…and then she would learn and then become kinda stagnant. The big moments I remember is when we started – so, she was only bottle-feeding milk, and then she got sick. She ended up getting a G tube at 4 years old, and then we saw another big jump at 4 when she started taking in real food because prior to that, it was just formula. So, then, there, we saw an increase in words and just cognitive development, and then we started the L-Serine last, so she’s been on it for almost a year now. And so, that’s another jump last year that we saw an increase in vocabulary and cognitive development. I would say those are the three distinctive moments..*.”

One caregiver (PI:1104) attributed improvements in pronunciation and enunciation to therapy and said they saw the biggest improvements between ages 5-9. For example, they said, “*He’s gotten better with being understood. It’s still a struggle. He had a speech assessment done when he was four or five, and they said that his articulation was at less than one-tenth of one percent at the time. And now, he’s understood about 80% of the time, which is pretty good*.” Please note, this child was the most advanced in the sample, and the caregiver believed their child may be the most advanced of the known cases of children with GRIN2B.

Caregivers of children without verbal speech described improvements in their child’s ability to use their AAC device (*n=*2), sound production (*n=*2), facial expressions (*n=*1), and processing ability (*n=*1). Examples can be found in Table S12. The two caregivers who saw improvements in sound production said this occurred around age 3.

Table S12. Examples of improvements in communication ability as per caregivers of children with GRIN2B-related neurodevelopmental disorder who do not report their child using verbal speech.

| Participant ID | Major Theme(s) Identified by Analyst | Quote(s) |
| --- | --- | --- |
| 1101 | Sound production | *He's gotten louder. Like, if he's upset, he gets real loud. For the longest time, we didn't know if he ever even felt pain because he just wouldn't make any type of noise.* |
| 1102 | Facial expression | *I feel like she’s more showing that gazing look or smiling a little bit.* |
| 1105 | Sound production | *Her communication was at such a low level where she would only cry because she didn't know how to control her voice to let us know what she needs. That's why we didn't know what was going on at the moment. Was she in pain? Was she uncomfortable? Was she hungry? Was she thirsty? And I feel like it has been like that ever since she was tiny. We would always think when babies cry that they’re hungry, they’re thirsty, they’re sleepy, they’re diaper’s wet. But sometimes maybe there was just something else going on. We just didn't know how to tell apart the different types of sounds and cues. So, it’s definitely a learning experience. She learned a lot, like I said how to control her voice. And we've also learned how to read those different types of sounds that she makes.* |
| 1108 | Use of AAC device  Processing ability | *And in the instances that she does use the AAC devices – I haven’t seen it firsthand, ‘cause we don’t really have those at our house but they’re saying that she’s getting better at using those, rather than just playing with them.*  *So, I would say she processes things a lot faster. Still not fast or a moderate speed. But she’s a lot faster at figuring out this situation is silly, this situation is bad, this situation is good. And so now, when she responds, you can see a lot more of her emotions, whereas before, we kind of just knew she was in a lot of distress, or if she was really overjoyed. And we had no real in-between. Now, she will even tell you, “I’m so sad.” “I’m angry.” And is able to literally give you a little more emotion.* |
| 1109 | Use of AAC device | *And then we went to GoTalk, where now it’s “I push this button and I get the drum,” or “I push” – whatever. So, I think he’s just kind of progressed in knowing the kinda cause and effect of what buttons do for him.* |

Three caregivers said changes in their child’s communication occurred when the child began attending pre-school/kindergarten and/or speech therapy. Four caregivers said improvements in communication skills are gradual and *slow*. For example, one caregiver (PI:1103) said, “*Nothing came quickly, and nothing continues to come quickly… it’s like a slow progression and it continues to be. But I do think we continue to see progress*.” Another caregiver (PI:1105) said, “*So, I would say it probably took us a good year and a half, two years to really be able to figure out what her needs are and how we can assist her whenever she’s squealing and what types of squeals are associated with what she needs. Yeah, so it’s been a slow, but it's progressing*.”

Similarly, caregivers (*n=*4) mentioned learning a new skill requires repetition and modeling. For example, one caregiver (PI:1109) said, “*Repetition is his learning style. He has to have constant repetition. And, if you stop doing it, he forgets*.” One caregiver (PI:1103) said they feel their child is improving with communication because of motivation. For example, they said, “*I think part of the reason why she’s continued to improve with her communication is that she’s motivated by it, she’s happy to work on it, so we also are facilitating that. I think her fine motor sucks. Like really sucks. And it’s just it’s a chore for us to work on it. She hates it. She hates writing, she hates coloring…Whereas she loves speech therapy, she loves music therapy where she can talk and communicate. She loves her friends; she wants to play with them. She wants to engage with us. She wants to tell us stuff. So, I feel like it’s easier for her to progress in those areas..*.”

One caregiver described their child’s inability to tax two systems at the same time during skill acquisition when the child was young. For example, the caregiver (PI:1103) said, “*If we were really working on consonant sounds, we could get a lot of consonant sound repetition or pairing with a more sign or something like that. And then we’d go focus on sitting or rolling or something else. And then it would be like, well [expletive], she’s not doing the m [sic] with the more sign now. And then we’d have to go back to it. So that’s been her way of learning and progressing with legitimately everything that we’ve done. Not just speech. It’s like a little bit in this stretch and a little bit backwards, a little bit forwards, a little bit backwards. And it’s not that we necessarily start off more behind but it’s like she develops in one area and then you flip the emphasis into another area, and you sort of stagnate or lose it.”*

#### Hearing & Vision Impacts

No caregivers of children with GRIN2B reported that their child had a hearing impairment. One caregiver of a child with GRIN2B reported that their child had a vision impairment. The caregiver (PI:1102) reported multiple vision impairments, including CVI and strabismus. The child was very young (age 2) and the caregiver could not speak to what impacts the impairments might have on the child’s communication.

#### Meaningful Change

All caregivers were asked, “*What would a meaningful change in communication look like for your child*?” And time permitting, most caregivers were asked “*If your child’s communication skills were to decline or get worse, which communication skills would be most important for your child to retain*?”

Parents generally interpreted “meaningful change” to mean “improvements” in communication ability or skills. During this question, some parents made the distinction of realistic or not realistic goals they had for their child. Four caregivers mentioned that they would like their children to communicate their preferences, with two caregivers responding that they would like their child to respond to complex questions (all themes in Table S13).

One caregiver (PI:1105) gave the following example, “*I mean like I said at the very beginning I would love for her to start to using a device that will tell us a little bit more specifics of her needs. An example would be whenever she’s hungry what does she want to eat? What’s her preference at the moment? If she wants a drink, what does she want to drink?”*

Another caregiver (PI:1108) stated that, “*I do just want to see her continue to increase her ability to express herself. So, right now, she can’t answer any questions that aren’t yes or no, and typical yes or no questions are not regularly reliable. If she was able to let people know more of what she’s thinking…If she was able to just kind of go, “Hey, do you like this?” “Yes.” “Do you like this?” “No.” And we could rely on and really run [with] it as an actual thing.*”

Table S13. Responses to “*What would a meaningful change in communication look like for your child*?” from caregivers of children with GRIN2B.

| Meaningful Improvement | 1101 | 1102 | 1103 | 1104 | 1105 | 1106 | 1108 | 1109 | Total |
| --- | --- | --- | --- | --- | --- | --- | --- | --- | --- |
| Be able to engage in conversation |  |  |  |  |  | 1 |  |  | 1 |
| More words using verbal speech |  |  |  |  |  | 1 |  |  | 1 |
| Improved communication to reduce frustration (when other people do not understand) |  |  |  | 1 |  |  |  |  | 1 |
| Communicate preferences using any modality (e.g., words, device) | 1 |  |  |  | 1 |  | 1 | 1 | 4 |
| Respond to complex questions |  |  | 1 |  |  |  | 1 |  | 2 |
| More consistent sounds (e.g., babbling) |  | 1 |  |  |  |  |  |  | 1 |
| Start using verbal words | 1 |  |  |  |  |  |  |  | 1 |

In terms of skills that would be important to retain if skills were being lost, the two caregivers who were asked this question mentioned sounds (*n=*1) and gestures (*n=*1). One caregiver (PI:1108) responded that if communication were to decline, “*So yeah, I think if she was able to keep her signing at a minimum, that would be the most necessary thing for her, because the “More” and “No more” signs are so much easier for people to understand across the board*.”

### Hao-Fountain Syndrome (HAFOUS)

#### Demographics

Nine caregivers of children with HAFOUS participated. Most caregivers were female and on average 43.1 years of age (Table S14). One caregiver self-identified as Hispanic-Latino, and all caregivers identified as white. All caregivers reported that they were currently married or living with a domestic partner. Overall, the participants reported high levels of education, employment, and annual income (Table S14).

Table S14. Demographic information for nine caregivers of individuals with Hao-Fountain syndrome.

| *Caregivers* | *n (%)* |
| --- | --- |
| Female | 9(100.0) |
| Age, years (Mean/SD) | 43.1/5.3 |
| Ethnicity |  |
| Not Hispanic or Latino | 8(88.9) |
| Hispanic-Latino | 1(11.1) |
| Race |  |
| White | 9(100.0) |
| African-American or Black | 0 |
| American Indian/Alaska Native | 0 |
| Asian | 0 |
| Middle Eastern | 0 |
| Native Hawaiian/Other Pacific Islander | 0 |
| More than one race | 0 |
| Relationship status |  |
| Single, never married | 0 |
| Married, or living with domestic partner | 9(100.0) |
| Separated | 0 |
| Divorced | 0 |
| Widowed | 0 |
| Highest grade in school |  |
| Less than high school diploma | 0 |
| High school degree or equivalent | 0 |
| Some college/University | 0 |
| College/University degree | 4(44.4) |
| Postgraduate degree | 5(55.6) |
| Occupational status |  |
| Homemaker | 2(22.2) |
| Unemployed | 0 |
| Retired | 0 |
| On disability | 0 |
| On leave of absence | 0 |
| Full-time employed | 4(44.4) |
| Part-time employed | 3(33.3) |
| Full-time student only | 0 |
| Income of U.S. residents |  |
| Less than $20,000 | 0 |
| Between $20,001 and $40,000 | 1(11.1) |
| Between $40,001 and $60,000 | 0 |
| Between $60,001 and $80,000 | 1(11.1) |
| Between $80,001 and $100,000 | 2(22.2) |
| Between $100,001 and $250,000 | 3(33.3) |
| Between $250,001 and $500,000 | 0 |
| $500,000+ | 1(11.1) |
| I prefer not to answer | 1(11.1) |
| I do not know | 0 |
| Relation to child |  |
| Mother/Step-mother | 9(100.0) |
| Father/Step-father | 0 |

Caregivers also provided demographic information for their child with HAFOUS. Five children were female, with similar rates to caregivers in regards to ethnicity and race (Table S15). Mutations included: missense, deletion, and indel/frameshift.

Four caregivers reported that their child had an ASD diagnosis and three reported that their child had epilepsy. Some children were receiving therapeutic services with almost all receiving speech therapy (Table S15). No caregivers reported that their child used an AAC device.

When asked about their child’s verbal language, six caregivers indicated their child used ‘full sentences’, no caregivers indicated that their child used ‘short phrases’, one indicated their child used a ‘few words’, and two indicated their child used ‘no words’.

Table S15. Demographic information for nine children with Hao-Fountain syndrome (HAFOUS).

| *Children* | *n (%)* |
| --- | --- |
| Age, years (Mean/SD) | 7.8/5.2 |
| Child gender Female | 5(55.6) |
| Ethnicity |  |
| Not Hispanic or Latino | 8(88.9) |
| Hispanic-Latino | 1(11.1) |
| Race |  |
| White | 9(100.0) |
| African-American or Black | 0 |
| American Indian/Alaska Native | 0 |
| Asian | 0 |
| Middle Eastern | 0 |
| Native Hawaiian/Other Pacific Islander | 0 |
| More than one race | 0 |
| Child’s genotype |  |
| Deletion | 2(22.2) |
| Missense | 4(44.4) |
| Unknown | 1(11.1) |
| Indel/frameshift causes nonsense | 2(22.2) |
| Autism Spectrum Disorder (ASD) | 4(44.4) |
| Epilepsy | 3(33.3) |
| Types of therapy |  |
| Physical Therapy | 3(33.3) |
| Occupational Therapy | 2(22.2) |
| Speech Therapy | 8(88.9) |
| Other therapy  ABA Therapy  Aquatic Therapy  Breathing Therapy  Equestrian Therapy | 1(11.1)  1(11.1)  1(11.1)  1(11.1) |
| AAC device |  |
| No | 9(100.0) |
| Yes | 0 |

#### Typical Communication Ability

The results for this section are organized by behaviors that correspond to expressive, receptive, and pragmatic (i.e. social) communication (Table S16), aligning with the conceptual framework that was developed for individuals with Angelman syndrome.^1^ Please note, although counts are reported to indicate salience of each theme, these data are limited in that they do not indicate *all* communication behaviors relevant to each child, only the ones that caregivers discussed during the interview.

Table S16. Number of caregivers that mentioned one or more examples of expressive, receptive, and/or pragmatic communication.

| Communication Behaviors | *n* (total *n* = 9) |
| --- | --- |
| Expressive | 9 |
| Receptive | 8 |
| Pragmatic (Social) | 7 |

##### Expressive

All caregivers of children with HAFOUS discussed expressive communication concepts. Requesting was a communication concept mentioned by most caregivers (Table S17).

Table S17. Expressive communication concepts on the ORCA measure mentioned by caregivers of children with HAFOUS.

| Expressive Communication Function | *n* (total *n* = 9) |
| --- | --- |
| Seeking Attention | 4 |
| Directing Attention | 4 |
| Refusing an Object | 6 |
| Requesting an Object | 8 |
| Requesting an Object out of View | 1 |
| Requesting More | 1 |
| Asking Questions | 3 |
| Telling Stories | 2 |

Four caregivers discussed how their child **seeks attention**. Two of the caregivers who discussed seeking attention shared that their children use words or word approximations to seek attention. For example, one caregiver (PI:1206) said, *“He can express [with words] that he’s angry, he can express that he’s hungry; he can express he wants to go somewhere, or how much he enjoyed something, or whether somebody’s annoying him, or whether he’s hurt, or whether he’d like your attention with something – whether he’d like to play a game, whether he liked – individual components of the game.”* The other caregiver (PI:1203) said their child could use words to seek attention, but also might use sounds. For example, they said, *“Right now, he yells a lot and gets frustrated, so he often screams to get my attention to ask for something, but he can say like, “I want to watch TV.”* Two other caregivers also described how their child uses sounds to seek attention. For example, one caregiver (PI:1209) said, *“Just like a couple of different noises he uses either to say yes, or no or to get our attention.”* Another caregiver *(*PI:1212) said their child could use sounds, as well as gestures or signs to seek attention. For example, they said, “*Well, she’ll yell and scream. Or she’s very – she’ll grab my hand or touch my face. I mean, because she’s very social. So, she really likes to have your full attention. Probably all of the time, she would prefer. But, yeah, it’s mainly she’s quite loud*.”

Four caregivers said their child used physical body movements to **direct their attention to something**. For example, one caregiver (PI:1203) said, “*He usually will say, “Momma, momma, momma,” and like really get in my face. Usually, with what he wants to show me*.” Another caregiver (PI:1211) said, “*She finds something and she brings – she’ll bring it to me.*” One caregiver (PI:1206) said their child also used a finger point to direct attention to something. For example, they said, “*And then, yeah, he’ll point to something, or he’ll just bring it to me, or he’ll try and kind of usher me in the right direction*.”

Six caregivers discussed how their children indicated **refusal**. Three caregivers described words/word approximations that their child uses to indicate refusal. For example, one caregiver (PI:1203) said that their child says *“no or no thank you.”* One caregiver (PI:1212) explained how their child uses modified gestures/signs to communicate refusal. For example, they said, “*She signs finished or all done.”* One caregiver (PI:1209) explained how their child uses sounds to communicate refusal. For example, they said, *“Just like a couple of different noises he uses either to say yes or no…you’ve got to have a trained ear to know the difference between the yes noise and the no noise.”*

Four caregivers explained how their children use physical body movements to communicate refusal. These behaviors required interpretation by the caregiver. For example, one caregiver (PI:1203) said their child will sometimes *“just ignore”* or “*walk away”* when they do not want to do something. Another caregiver (PI:1212) said, “*Now, she’ll actually push it away with her hand. If it’s food or something she’ll move her face if she’s done. When she’s full, sometimes she’ll just open her mouth and let the food drop out.”* This caregiver also described how their child will cry/fuss to communicate refusal, saying, *“Refusing something, so she hates transitions. So, she will cry, or kick a bit, or push us away with her hand, or scream. She used to try and bite us quite a bit, but we kind of leveled off with that behavior outburst in the last – probably since July*.”

Eight caregivers discussed how their children communicated **requests**. All caregivers indicated that their child utilized symbolic communication to request (e.g. gestures, signs, words, word approximations), although the specific modality differed between children. The most commonly requested objects were food and drink, but individuals with USP7 in our sample also requested TV and places/activities (i.e. outside). Four caregivers gave examples of their child self-referencing while requesting.

Two-thirds of caregivers said their child used words/word approximations to request. For example, one caregiver (PI:1203) said, “…*he can say like, “I want to watch TV.” He asks for water, to go wake up his brother... we’ve had a huge breakthrough in his speech, so he has words for just about everything you can think of*.” Another caregiver (PI:1207) said, “*So, she uses the word “me” a lot to refer to herself versus “I”. So, we’ve been working on that. Her grammar – like the order syntax of her words – are not accurate. So, she would say – “Me go – school,” or –she’s recently started adding “want” – like, “Me want –” something. Or, if she’ll wanna go out to dinner, she’ll say, “Out dinner?” She’ll skip a lot of the in-between words.”*

Two caregivers described gestures/signs (ASL sign, finger point) their child uses to request. One of these examples included **requesting more of something.** For example, one caregiver (PI:1212) said, “*So, she doesn’t request a ton of things. But she will sign, “Please.” Or she’ll sign, “More.” And then, “Food,” of course. But she’ll sign the same thing for a drink. She has started pointing to things which is fairly new. If she’s sitting in her chair and she’ll see her water cup on the counter, she’ll point to her water cup sometimes, not consistently*.”

One caregiver (PI:1211) discussed how their child **requested objects that they cannot see** using both symbolic and non-symbolic communication. For example, they said, “*She finds something and she brings – she’ll bring it to me, and she wants that, or if she wants to play with it, or her Chromebook. She knows when she wants something she can ask for it, or go look for it, or talk about it*.”

Three caregivers discussed how their children **ask questions**. All of the caregivers who described how their children ask questions described them using words/word approximations. For example, one caregiver (PI:1203) said: *“He asks permission to do a lot of things, like even simple things like can I look out the window. Like he needs me to tell him that it’s okay. He asks for water, to go wake up his brother.”* Another caregiver (PI:1204) said, *“[the child asks] Like what is going to happen next or what we’re going to do or what time we’re going to leave. She always wants to know what’s going to be happening. It’s kind of a planning thing for her. She just wants to be in the know. I think it just helps with her anxiety as well.”* Along those same lines, another caregiver (PI:1206) also described how their child likes to ask questions about future events: *“He would ask what’s for tea. So, he would ask about upcoming-like the weekend, “Are we doing anything?” So, he can project sort of future- sort of three or four days or even up to a month.”*

Two caregivers discussed how their children **told stories**. One caregiver (PI:1203) said, “*Well, he’s really into Thomas the Tank Engine, and we have train tracks and like most of the engines, so he watches the old Thomas TX series pretty frequently so he likes to tell rather fantastical stories. They don’t always make sense. A lot of it is reenacting things, so he’ll say, you know, “oh, so and so is stuck in the tunnel,” and it’s from like an episode, or he’ll say, “I want to build the trains or the tracks, or I want to-I need to have all my engines together.” Little bit of like a hyper fixation, but he’s also getting into the pretend aspect of it.”*

##### Receptive

Eight caregivers discussed aspects of receptive communication during the interview. Specific skills that they endorsed included following familiar one-step directions (*n=*7), responding to simple questions (*n=*3), making choices (*n=*3), understanding isolated words or phrases (*n=*1), engaging in back-and-forth conversations (*n=*1), and following novel directions (*n=*1).

Seven participants discussed how their child was able to **follow** **familiar or routine one-step directions**. One caregiver (PI:1209) stated, “*He would understand the instruction. If I say, “Go and sit on the step, so we can put your shoes on,” he’ll go and sit down on the step and wait for me to put his shoes on him, or if I say, “We need to go upstairs,” then, as long as he agrees to it, he’ll walk off to the stairs. If he’s playing on his tablet and he needs help with something and he’s asking me for help, I’m the other side of the room, if I say, “Well, bring it here, then.” Then, he’ll pick it up and bring it to me.”*

Another caregiver (PI:1204) gave an example of how they support their child with **novel directions**, “*I guess if I asked her, pick up her dishes from the table or put her laundry away, she would do those; she would do that. I’m trying to think of another example. Where she might need more direction? For example, we were doing a little craft project and she had a little friend here that's younger than her. She needed a lot more direction than her friend did who was actually younger than her, and you could see her frustration. I needed to actually really show her and imitate what she needed to do for her to understand it. So, a lot of times that is something I need to do for her is actually show her, and then she understands it better before she can do it*.”

Two caregivers (PI:1203 & PI:1206) described how their child **madechoices** using words or word approximations. For example, one caregiver (PI:1206) said, *“If it’s an option of going somewhere or doing something, he’ll study it for a moment and then decide what he wants to do. His processing is a little bit slow, so you do have to give him time. But he can answer, and he can put some thought into it.”*
The other caregiver (PI:1203) shared that their child struggles with making choices and would sometimes pick the last thing he heard, saying, “*He still – he used to – a lot of the time, if I would say like do you want X or Y, he would always just say Y, because it was the most recent thing he heard. And he does still do that sometimes, but he really struggles with making choices. A lot of times, he needs time. If I ask him, “Do you want to have your diaper changed on the couch or on the floor?” He’ll – you know, I have to wait up to three – like the therapist told me wait three times as long as you would expect for an answer. Like he really has to think about it, and now he’s started having additional questions, or he’ll come up with a choice that wasn’t presented to him.”*

One caregiver (PI:1202) reported that their child used gestures or signs to make choices, saying, “*He’ll listen, and either if – if he knows the sign – I try to ask him questions like “Do you want an apple or a banana?”, and he knows both signs, so he’ll tell me which one he wants or whatever, but if it’s something – if I ask him a question like “Are you feeling bad?” or something that is a complex kind of answer, he doesn’t – he’s almost 4, so he might not understand the concept of feeling bad. But, if he knows the sign, he will answer with the sign.”*

In this group, two caregivers mentioned how processing delays affect their children. One caregiver (PI:1206) gave the following example, “*He gets confused so easily, and flustered, and frustrated, and angry, and – he’ll just lose the thread completely. Even if it’s just one item, and you say, “Take this to so and so,” – that’s one item. So, I’ve put two variables in – but he’s got one of them in his hand. You can’t guarantee that that person will receive it. He’ll get halfway there and forget he was supposed – So, then, he’ll come back and say, “Who was I given this to?” “So and so.” “Okay. And where are they?” “They’re in their bedroom.” “Okay. Okay.””*

##### Pragmatic

Seven caregivers discussed pragmatic (social) behaviors that they have observed in their children. Specific skills included using names (*n=*3), recognizing mood and comforting someone (*n=*2), polite words (i.e. thank you, please; *n=*2), greetings (*n=*1), and pretend play (*n*=2).

In terms of **using names**, one caregiver (PI:1203) said, *“He usually will say, “Momma, momma, momma,” and like really get in my face.”* Another (PI:1207) said, *“She used to not be able to say her brother’s name, “[name],” but she can say it now, but it’s more like – I wish I had her recorded. It’s not enunciated. So, it sounds more like –[name].”*

Two caregivers talked about their children **recognizing mood in others and comforting them**. One caregiver (PI:1203) said, “*Sometimes I don’t know how much true empathy there is, but he does notice people are sad and he will say like, “I want to help you feel better,” and a lot of times he doesn’t like it when people are sad, and so he’ll be like, “Okay. Here. I brought you this popper. Now, you feel better.” Like, period. Like end of discussion. You have to feel better*.”

Another caregiver (PI:1204) described how her child likes to talk on the phone, “*She talks a lot on the phone to family members. She is like a little social butterfly like that. Very socially, especially to our family. And they live in [State name] and [State name], so she likes to keep in contact with everyone that way. She texts on her phone, and she does have a couple of friends that she does that too as well here where we live.”*

Two other caregivers described **social games** that their children play. One caregiver (PI:1203) said, *“They’re like nonsensical games, so like they have a game they play with the trucks. And so, he often struggles to give his brother his turn and will just say, “It’s my turn,” – he’ll bounce back and forth from being able to take turns to not being able to take turns, and sometimes, like if it’s time for him to take a turn and he can’t and, you know, we – I step in.”* The second caregiver (PI:1211) said, *“They love mimicking going to the doctor. It’s hilarious. They are into doing now, in the sofa area, with some stuff, putting together a house, and they move everything. And they say, “Oh, this is a refrigerator” and “This is the bed.” And I’m like, “Why don’t you do this?” She says, “No, no, we need the bed, so we cannot do that.”*

#### Changes in Communication

The research team interviewed nine caregivers whose children had HAFOUS and all nine discussed how their child’s communication changed overtime.

Six out of nine caregivers spent the majority of the time describing how their child developed verbal speech. Five out of the six caregivers said their child began using words between ages 2-3. Caregivers said their child had no words, and then one day, their child just began using words. For example, one caregiver (PI:1203) said, “*So, when he was between 18 months and 24 months, he only had, I think, about eight words that we could recognize and that he said… That summer, he went from having barely being able to string two words together. Like if he put two words together when he was three, it was like remarkable. And then, by the end of the summer, he was like actually communicating. It was just this rapid, rapid growth..*.” Another caregiver (PI:1208) said, “*And she had about 30 or 40 words heading into pre pre-K...So, that’s when you’re three, two and a half, I guess. And then within a year, so she went from about 30 words to I don't know, six, seven, eight [hundred].”*

Prior to gaining verbal speech, several caregivers (*n=*4) said their child was reliant on ASL to communicate; however, once their child began using verbal speech, the child stopped using their ASL signs. For example, one caregiver (PI:1204) said, “*Early on before she had her words, she did use sign language a little bit. But she has words now, so she’s good*.”

Caregivers described their child relying on a few words initially and then increasing vocabulary and stringing words together into sentences overtime. For example, one caregiver (PI:1206) shared, “*He was non-verbal until six. And then, he was – sort of one or two words until about I don’t know I’m guessing here about eight where he could string maybe up to four or five together. And then, it just took off. So, by the time he was nine or 10, it was pretty much as it should be*…” Another caregiver (PI:1207) said, “*So, about two years ago. So, age six. So, up until that point, we were just using – I mean, really, [child’s name] didn’t have very many – well, she didn’t really have any words until like age three. So, she’d say…sounds that sounded like “Mom” and “Dad,” but she wasn’t really talking a lot. She just had like a handful of words until age five. And then, she’d start using words here and there, but not sentences at all until probably first grade*.”

One caregiver (PI:1211) said increases in vocabulary came in “bursts”. For example, they said, “*She goes like bursts. She’s stuck and then, suddenly, you can see a growth in her vocabulary... And then, she gets stuck again. So, recently, it’s like a stair instead of a slope. It’s like she progresses and gets stuck for a while there. And then, she progresses and gets stuck there*.” Two caregivers said they saw the biggest jump in their child’s vocabulary between ages 3-4, one caregiver said between ages 5-6, and one caregiver said the biggest jump occurred between ages 8-10. Several caregivers said their child is currently working on improving pronunciation and annunciation (*n=*3), use of proper pronouns (*n=*2), and certain vowel sounds (*n=*1) in speech therapy.

Three caregivers said their child had no verbal words. While discussing changes over time, these caregivers described improvements in their child’s use of ASL. For example, one caregiver (PI:1202) said, “*Well, sadly, they’re really not changing a whole lot, except learning new signs. He’s still nonverbal, so as far as verbal communication, there’s been no change. In sign language, he’s just learned more signs*.” Another caregiver (PI:1209; 3 year old) said, “*He’s learned more signs…and he has started to use them spontaneously. He’ll come and sign something to us to tell us what he wants without us having to – originally, when he first learned signs, he would only sign a word after we had said it or we had signed it, but now, he will use his signs spontaneously*.” This caregiver also said their child’s use of sounds have regressed over time. For example, they said, “*His noises that he makes have sort of reduced a bit, become less interesting. He used to quite a good [grunt] for no… And now, his no is quite hard to – it’s just like a [makes sound], like that, and can be very similar to his yes. You’ve got to have a trained ear to know the difference between the yes noise and the no noise. They used to be more different*.” Along with an improvement in their child’s ASL, one caregiver (PI:1212) also said her child’s processing speed has improved, saying, “*But then, her reaction time for communicating has gotten better. I mean, now, we’ll say, “Oh, there’s Mrs. [name],” or, “Say goodbye to Mrs. [name].” And right away, she’ll raise her hand and wave. She never used to do anything like that*.”

Three caregivers said improvements in communication skills are gradual and slow. For example, one caregiver (PI:1204) said, “*Her progress has been just slow and steady, like with word increase in vocabulary.”* Another caregiver (PI:1212) said, *“It’s like everything has been super, super delayed…she’s gonna be 15. And she’s more like a one a half-year-old. And in some cases, a one-year-old. And even though children like that progress. It’s a lot faster. But I’m, okay, maybe when she’s 18, maybe she’ll be at two and a half, three-year-old level…that’s what we hope for. I mean, obviously, quicker would be awesome, but that’s just not gonna happen with her*.”

Similarly, caregivers (*n=*3) mentioned learning a new skill requires repetition and modeling. For example, one caregiver (PI:1202) said, “*He will lose a sign if we don’t use it, but as far as… He does pick them up very quickly*.” Another caregiver (PI:1209) said, “*If we stop using a word or a sign, and then we’ve not used it for a while, and then he’ll stop using it, and then he doesn’t – he forgets about it. I think the signs, I think he remembers, so if you start trying to remind him about a sign and do it again, then he’ll gradually pick it up again, but it’s the same with skills that he has. If you don’t sort of keep doing it, then he…gets out of practice, and then needs to get back into it to be able to do it again*.”

One caregiver described their child’s inability to tax two systems at the same time during skill acquisition when the child was young. For example, the caregiver (PI:1204) said, “*Now, I think she can work on a lot of things at once. But I think that when she was younger some things kind of took a back seat when she was working on, for example, walking or crawling…but now I feel like she can do more than one thing at a time a little bit better. But I think, early on, that was a bit more challenging*.”

#### Hearing & Vision Impacts

No caregivers of children with HAFOUS reported that their child had a hearing impairment. Three caregivers reported that their child wore glasses. One of those caregivers (PI:1211) specified that this was to correct farsightedness.

A fourth caregiver (PI:1203) said their child has spatial awareness and depth perception issues, but had not received any formal diagnoses. This caregiver said, “*He really struggles with spatial awareness, so if I tell him to find his shoes, he can’t. They could be right in front of him and he can’t see them, and so he’ll kind of just wander until he forgets what it was*.” Caregivers did not describe any impacts on communication.

#### Meaningful Change

All caregivers were asked, “*What would a meaningful change in communication look like for your child*?” And time permitting, most caregivers were asked “*If your child’s communication skills were to decline or get worse, which communication skills would be most important for your child to retain*?”

Parents generally interpreted “meaningful change” to mean “improvements” in communication ability or skills. During this question, some parents made the distinction of realistic or not realistic goals they had for their child. In this group, three caregivers mentioned that they would like their children to engage in back and forth conversation, with two caregivers each responding that they would like their child to use verbal words or improve verbal pronunciation (all themes in Table S18).

Table S18. Responses to “*What would a meaningful change in communication look like for your child*?” from caregivers of children with HAFOUS.

| Meaningful Improvement | 1202 | 1203 | 1204 | 1206 | 1207 | 1208 | 1209 | 1211 | 1212 | Total |
| --- | --- | --- | --- | --- | --- | --- | --- | --- | --- | --- |
| Start using verbal words | 1 |  |  |  |  |  |  |  | 1 | 2 |
| Engage in back-and-forth conversation |  | 1 |  | 1 |  |  |  |  | 1 | 3 |
| Communicate feelings and emotions |  | 1 |  |  |  |  |  |  |  | 1 |
| Unable to answer this question^1^ |  |  | 1 |  |  |  |  |  |  | 1 |
| Improved cognitive processing |  |  |  | 1 |  |  |  |  |  | 1 |
| Speak in grammatically correct sentences |  |  |  |  | 1 |  |  |  |  | 1 |
| Improved verbal word finding |  |  |  |  | 1 |  |  |  |  | 1 |
| Communicate if need help/ in danger |  |  |  |  | 1 |  |  |  |  | 1 |
| Improve verbal pronunciation |  |  |  |  |  | 1 |  | 1 |  | 2 |
| Start using sounds |  |  |  |  |  |  | 1 |  |  | 1 |
| Put two gestures/signs together to communicate a message |  |  |  |  |  |  | 1 |  |  | 1 |

^1^More information about this caregiver’s response is included in the text.

Two caregivers whose children had full sentences and fairly advanced communication discussed how HAFOUS interacts with ASD. One caregiver (PI:1206) stated, “*I don’t think [communication is] going to get any better. I don’t think anything that you could give him – would make his communication better because of who he is. He’s autistic. And he does have some brain damage. He is intelligent, but his focus lacks. He gets everything out of his communication that he needs. So – whether I would like better conversation or not is totally irrelevant. It serves his needs – and adequately. More than adequately*.” Another caregiver (PI:1203), discussed something similar when discussing meaningful change, “*That’s a tough one, because I feel like what I want is almost like at odds with who he is, like as an autistic individual, but I think like for me just to have more normal conversations. Like just be able to ask him a direct question and get a direct answer, so that I knew what he was going through.*”

One caregiver was unable to answer the question, as they felt their child’s communication was already in a good place. They (PI:1204) said, “*I feel like now we’re at a good place early on. Of course, her learning to talk and getting through all those frustrations. Things that stick out to me, like being playing on the playground and kids coming up to her and asking her if she wants to play and her not being able to respond. Those were our frustrating days. So, I feel like we’ve already passed those. So, when I hear of other parents that have kids with USP7 or other syndromes or rare diseases talk about that, I feel like we’ve kind of passed that. I’m sure there’s other things. We’re dealing with other things now. So, I feel like communication is kinda on the backburner now because she does, I don’t wanna say, well enough, but she does do it pretty good. And she’s able to communicate her needs. So, I guess if you were to ask me that 10 years ago when she was four, I would've been like “Oh my gosh. I just need her to be able to say a sentence, and play with her friends.” We have friends that have that problem right now and I just pray every day that their little kids will start talking. I feel like we’re okay right now. We don’t have that problem*.”

Of the five caregivers who were asked about skills to retain if communication were to decline, some caregivers could not conceptualize this question and did not provide an answer. Two caregivers mentioned ‘communicating wants and needs’ with a third mentioning using gestures/signs to specifically communicate needs. For example, one caregiver (PI:1204) said, “*I guess just being able to tell us her needs and how she’s feeling and what she needs.”*

### HNRNPH2-related disorders

#### Demographics

Seven caregivers of individuals with HNRNPH2-related disorders participated. All caregivers were female and on average 41.1 years of age (Table S19). No caregivers self-identified as Hispanic-Latino. Six caregivers identified as white and one as American Indian/Alaskan Native. All caregivers reported that they were currently married or living with a domestic partner. Overall, the participants reported high levels of education, employment, and annual income (Table S19).

Table S19. Demographic information for seven caregivers of individuals with HNRNPH2-related disorders.

| *Caregivers* | *n (%)* |
| --- | --- |
| Female | 7(100.0) |
| Age, years (Mean/SD) | 41.1/6.1 |
| Ethnicity |  |
| Not Hispanic or Latino | 7(100.0) |
| Hispanic-Latino | 0 |
| Race |  |
| White | 6(85.7) |
| African-American or Black | 0 |
| American Indian/Alaska Native | 1(14.3) |
| Asian | 0 |
| Middle Eastern | 0 |
| Native Hawaiian/Other Pacific Islander | 0 |
| More than one race | 0 |
| Relationship status |  |
| Single, never married | 0 |
| Married, or living with domestic partner | 7(100.0) |
| Separated | 0 |
| Divorced | 0 |
| Widowed | 0 |
| Highest grade in school |  |
| Less than high school diploma | 0 |
| High school degree or equivalent | 2(28.6) |
| Some college/University | 1(14.3) |
| College/University degree | 3(42.9) |
| Postgraduate degree | 1(14.3) |
| Occupational status |  |
| Homemaker | 1(14.3) |
| Unemployed | 0 |
| Retired | 0 |
| On disability | 0 |
| On leave of absence | 0 |
| Full-time employed | 5(71.4) |
| Part-time employed | 1(14.3) |
| Full-time student only | 0 |
| Income of U.S. residents |  |
| Less than $20,000 | 0 |
| Between $20,001 and $40,000 | 1(14.3) |
| Between $40,001 and $60,000 | 0 |
| Between $60,001 and $80,000 | 1(14.3) |
| Between $80,001 and $100,000 | 3(42.9) |
| Between $100,001 and $250,000 | 1(14.3) |
| Between $250,001 and $500,000 | 0 |
| $500,000+ | 0 |
| I prefer not to answer | 1(14.3) |
| I do not know | 0 |
| Relation to child |  |
| Mother/Step-mother | 7(100.0) |
| Father/Step-father | 0 |

Caregivers also provided demographic information for their child with HNRNPH2-related disorders. As expected for an X-linked condition, all children were female, with similar rates to caregivers in regards to ethnicity and race (Table S20). All mutations were considered ‘missense.’

Two caregivers reported their children had an ASD diagnosis and three reported that their child had epilepsy. Some children were receiving therapeutic services including physical, occupational, and speech therapy (Table S20). Five caregivers reported that their child used a high-tech AAC device. The settings in which the device was used varied (Table S20). The mean age of the child when the device was introduced was 2.6 years old (*SD* = 1.5).

When asked about their child’s verbal language, one caregiver indicated their child used ‘full sentences’, no caregivers indicated that their child used ‘short phrases’, three indicated their child used a ‘few words’, and three indicated their child used ‘no words’.

Table S20. Demographic information for seven children with HNRNPH2-related disorders.

| *Children* | *n(%)* |
| --- | --- |
| Age, years (Mean/SD) | 8.3/2.5 |
| Child gender Female | 7(100.0) |
| Ethnicity |  |
| Not Hispanic or Latino | 7(100.0) |
| Hispanic-Latino | 0 |
| Race |  |
| White | 6(85.7) |
| African-American or Black | 0 |
| American Indian/Alaska Native | 1(14.3) |
| Asian | 0 |
| Middle Eastern | 0 |
| Native Hawaiian/Other Pacific Islander | 0 |
| More than one race | 0 |
| Child’s genotype |  |
| Missense | 7(100.0) |
| Autism Spectrum Disorder (ASD) | 2(28.6) |
| Epilepsy | 3(42.9) |
| Types of therapy |  |
| Physical Therapy | 3(42.9) |
| Occupational Therapy | 5(71.4) |
| Speech Therapy | 5(71.4) |
| Other therapy  Equestrian Therapy  Orientation and Mobility Therapy  Vision Therapy | 1(14.3)  2(28.6)  2(28.6) |
| Age first introduced to device (mean/SD) | 2.6/1.5 |
| AAC device |  |
| No | 2(28.6) |
| Yes | 5(71.4) |
| Device |  |
| High tech | 5(71.4) |
| Low tech | 0 |
| Places to use the device |  |
| Home | 5(71.4) |
| School | 4(57.1) |
| Out in the community | 0 |
| Other | 0 |

#### Typical Communication Ability

The results for this section are organized by behaviors that correspond to expressive, receptive, and pragmatic (i.e. social) communication (Table S21), aligning with the conceptual framework that was developed for individuals with Angelman syndrome.^1^ Please note, although counts are reported to indicate salience of each theme, these data are limited in that they do not indicate *all* communication behaviors relevant to each child, only the ones that caregivers discussed during the interview.

Table S21. Number of caregivers that mentioned one or more examples of expressive, receptive, and/or pragmatic communication.

| Communication Behaviors | *n* (total *n* = 7) |
| --- | --- |
| Expressive | 7 |
| Receptive | 7 |
| Pragmatic (Social) | 6 |

##### Expressive

All caregivers of children with HNRNPH2-related disorders discussed expressive communication concepts. Requesting was a communication concept mentioned by all caregivers (Table S22).

Table S22. Expressive communication concepts on the ORCA measure mentioned by caregivers of individuals with HNRNPH2-related disorders.

| Expressive Communication Function | *n* (total *n* = 7) |
| --- | --- |
| Seeking Attention | 3 |
| Directing Attention | 1 |
| Refusing an Object | 5 |
| Requesting an Object | 7 |
| Requesting an Object out of View | 1 |
| Requesting More | 1 |
| Asking Questions | 2 |
| Telling Stories | 1 |

Three caregivers discussed how their child typically **seeks attention**. One caregiver (PI:701) shared that their child uses sounds to seek attention: “*So, when she’s excited about something, she’ll squeal and like scream in excitement. She’ll make other utterances that definitely alert us that she needs help. She’s not saying, but she might just make repeated “Uh, uh, uh” that we right away know like, “Oh, what’s going on here?” And we might look at her iPad, and maybe it’s either gone to a screen that requires like to put in the code so that she can get back in.”* Another caregiver (PI:704) reported that their child uses both signs and words/word approximations to seek attention: *“If she cannot get your attention the traditional way that someone using American Sign Language would, she can say “Mom,” which is another word that she can say, or she will scream and grunt until you physically put eyes on her, or she can tell you what she wants.”* One caregiver (PI:705) said that their child uses a high tech AAC device to seek attention. For example, they said, *“If she wants a member of the family, she’ll call their name on her talker.”* Finally, two parents described how their child uses physical gestures (e.g. reaching, grabbing, or pulling) to seek attention. For example, one caregiver (PI:704) said, “*Generally speaking, she will wheel over to someone in her wheelchair and get their attention. She’ll either hug them, kiss them, or basically just kind of – I would hate to say pull on them, but she would.*”

One caregiver spoke about how their child **directed attention** using their physical body movements (PI:701). They said, “*She’ll hand us things that she wants to interact with*.”

Seven caregivers of individuals with HNRNPH2-related disorders discussed how their children communicated **requests** using different modalities. Three caregivers described gestures/signs their child used to request. For example, one caregiver (PI:701) said, “*She also I think uses gestures. She’ll hand us the iPad. She’ll reach. Like I said, sometimes she’ll also do things with your hands that shows that she wants to maybe play with you, so she’ll take your two hands and clap them together..*.” Another caregiver (PI:707) said, “*So, she’ll point. It’s not a true point but she kind of has her fingers spread and her index finger moves down a little bit from the other fingers. But enough that we know that she’s pointing at something… And she has a few other signs like fish as I said she’s really into fish right now..*.” Several HNRNPH2 caregivers (*n=*3), all with older children (over age 10), discussed how their child used their AAC device to request. For example, one caregiver (PI:707) said, “*Yep, I mean, so they’re all programmed in there. All of her favorite shows and her favorite songs. So, she will literally just use her device, say the name of the show and then look at us expectantly*.” Another caregiver (PI:705) said, “…*there are some things she will actually ask for on her device… we found that eye gaze worked best for her with a communication device, and so, as I said, she’s had that for about two years now. We had gotten up to the point where she was forming three-word sentences last summer, so she’d had it a little over a year, and she was saying things like “I want eat, I want drink, I want movie*.”

Three caregivers said their child used words or word approximations to request. For example, one caregiver (PI:703) said, “*She uses a lot of the sentence structure that she’s familiar with. Like, “I want. I see. I need”…. “I want iPad. I want food. I am hungry. Let’s go outside..*.*”* Another caregiver (PI:701) said, *“We might get something like “eye bah, eye bah, eye bah,” which is iPad. But this is not happening with consistency or a lot of frequency.”* Some caregivers described how their child uses sounds to request. For example, PI:702 said, *“She just makes sounds like whiny when she needs something…* *So, she’s just start whining and getting anxiety, and we’re like, “Okay, we’ve got to figure out what she wants.” It’s either her cup or her music…* *We’ll try everything, and then it might even just be like holding her for a few minutes or taking her outside for fresh air. Just little things like that, and then she’s okay... Yeah, we just be patient with her and try to figure it out the best we could.”* Another caregiver (PI:701) said, *She’ll make other utterances that definitely alert us that she needs help. She’s not saying [help], but she might just make repeated “Uh, uh, uh” that we right away know like, “Oh, what’s going on here?” And we might look at her iPad, and maybe it’s either gone to a screen that requires like to put in the code so that she can get back in.”*

Finally, caregivers also mentioned behaviors that their child used to make requests that require more interpretation on behalf of their communication partner. For example, parents described how their child might use physical gestures to request, in addition to more symbolic communication. One caregiver (PI:705) said, “*And, just like I said, physically picking up her cup, going to the refrigerator with it to communicate she’s thirsty. She will say “want drink” on her talker, too, but she will pick up her drink and go to the refrigerator. She will go to the pantry and open the door if she’s hungry, or she will come and get anything that’s sitting out that looks edible*.”

One HNRNPH2 caregiver (PI:701) discussed how their child **requested more** of something using their AAC device. For example, they said, “*and from time to time, she’s able to on her own put maybe two buttons together, like “want more.” Instead of just “more,” now they’re trying to teach her to put two words together, like “want more*.”

One caregiver (PI:706) discussed how their child **requested objects that they cannot see** using symbolic communication (e.g., words). For example, they said, “*So, sometimes if she's wanting to do a craft with scissors but can't think of the word "Scissors," she'll just say, "Cut," or she'll say "Knife" because she knows a knife can cut something. So, she'll try to do the best she can with her own vocabulary, but when that doesn't work, she'll resort to gestures or maybe an alternate meaning of a word*.”

Five caregivers discussed how their child communicated **refusal**. Three caregivers described gestures/signs (e.g. “all done”, shake head) that their child used to refuse something. For example, one caregiver (PI:705) said, “*and actually, she does still use the sign for “done,” like she will tell you, “I am all done with whatever you are doing with me right now.”* Another caregiver (PI:706) said, “*So if I ask her, “[child name], what’s wrong?” she is not able to give me words, she will just shake her head…”* This caregiver also indicated that her child used words/word approximations to communicate refusal (e.g., *“No, no*”) in combination with shaking her head. One caregiver (PI:702) described how their child will “*push items away”* to indicate refusal. Another caregiver (PI:707) described how their child cries/fusses to indicate refusal, saying, “…*through sounds, so the sounds usually when she doesn’t want to do something she just makes an unhappy sound”* (Please note: the interviewer confirmed the “unhappy” sounds were specifically crying and fussing during the interview).

Two caregivers spoke about how their child **asked questions**. One caregiver (PI:703) said, “*[the child verbally says]* ‘*What are you doing?’ if she sees that we’re doing something that she’s familiar with*.” The other caregiver (PI:706) said, *“The first thing she'll ask people is, "what's your name?" – which she understands that social cue that when new people are meeting, "Oh, hi, I'm so-and-so, what's your name?" and so she's picked up on that.”*

One caregiver discussed how their child **told stories** about something that happened in the past. This caregiver (PI:703) said, “*So, if you say, “What did you do at school today?” Like, “What did you do at school?” She might be able to come up with, “I played,” or, “I read a book*.”

##### Receptive

Seven caregivers described examples of their child’s receptive communication. For this group, receptive communication concepts included following familiar directions (*n=*5), responding to simple questions (*n=*2), and understanding isolated words or phrases (*n=*1).

Five caregivers described how their children could **follow familiar directions**. For example, one caregiver (PI:703) stated, *“So, I’ll say if we finished dinner, “Put your cup in the sink, and throw your plate away.” So, she can follow those two-step directions. Again, those are familiarized directions, I guess. And she’s very familiar with those, so she can follow those. Now, if you said something that she didn’t understand, or didn’t really know, or wasn’t familiar with, she wouldn’t be able to do those.”*

Two caregivers spoke about their child **responding to simple questions.** For example, one caregiver (PI:704) said, *"I can ask her genuinely how her day at school has gone, and her response is, “Good.” Good is one of the very few words she can physically say."*

One caregiver mentioned that her child **understands certain words or phrases.** This caregiver (PI:701) said, *“So, I think that food and like movies, music, iPad, those things catch her attention, if I’m talking about those things. But I wouldn’t say that she has the – so, she might make eye contact with me when I talk about those sorts of things, but when I’m talking about other things, she might not even regard me at all. She might not look at me at all."*

Two caregivers discussed how it takes their child additional time for them to cognitively process what is being said to them, which impacts their response to communication (and thus, would impact how someone observes their receptive understanding). One caregiver (PI:704) used an analogy for the processing delay: “*The motor highway for our children from one side of the brain to the other side of the brain is like a congested highway. So, it reminds me of traveling through the middle of [large American city]. And they’re physically trying to maneuver getting around the vehicles to get to where they need to be. So, it’s a two, three-minute processing delay. So, I can give an instruction, or ask a question, and then you have to give adequate time for the answer. You can’t jump to the answer or repeat the question. You’ve got to physically give enough time for that processing to be relayed.*”

##### Pragmatic

For pragmatic communication, caregivers of individuals with HNRHPH2-related disorders generally mentioned how their children greeted people using social smiles, gestures, or words (*n=*5). One caregiver (PI:701) spoke about their child giving hugs/kisses, saying, “*She’ll put her hand around your neck and then pull you close to kiss you or hug you or nuzzle you”*. The same caregiver also reported that their child could **initiate a game.** For example, this caregiver said, *"Sometimes she’ll also do things with your hands that shows that she wants to maybe play with you, so she’ll take your two hands and clap them together, and she’ll even put – she’ll take your hands and put them over your eyes and take them away, and put them over your eyes and take them away, almost in a peekaboo fashion."*

#### Changes in Communication

The research team interviewed seven caregivers whose children had HNRNPH2-related disorders and all seven discussed how their child’s communication changed overtime. Every caregiver described improvements in their child’s communication, including improvements in vocabulary and AAC device usage (Table S23).

Table S23. Quotes from HNRNPH2 caregivers describing improvements in their child’s communication skills over time.

| Participant ID | Quote |
| --- | --- |
| 701 | *But on the other hand, how it’s changed, she’s learned to use buttons and she’s even self-correcting. When she hits a button that isn’t correct on the communication device, she is doubling back and correcting herself…and from time to time, she’s able to on her own put maybe two buttons together, like “want more.” Instead of just “more,” now they’re trying to teach her to put two words together…* |
| 702 | *And then just this past year, she’s been doing a lot more paying attention to like me folding clothes, watching her brother or sister walking by and just them talking – interacting with each other.* |
| 703 | *I would say maybe she had some singular words, maybe three years old…* *And then it started to build. Then it was, okay, we can put two words together. It was, “Want food. Want book. Want lovey. Want bed. Want blanket.” It was building that, and then that started, “I want to go to bed,” or, “I want to sleep.” Then it started to build into more sentences like that.* |
| 705 | *It’s been a very, very gradual process to get where we are on a device…* *she started out with literally just [sic] icons, and we are now up to – I couldn’t even tell you how many pages we’ve got now, 100 pages with 16 icons on each page…* |
| 706 | *Her communication has gotten much better; she has gone from not talking at all and just kind of grunting to the place where she has quite a vocabulary. She can have a conversation with somebody and be able to speak in sentences with people about a topic, whereas before she might be having a one-word answer, but she's actually been able to get to the point where she can have a conversation and in a sentence communicate and contribute to a particular topic about something…* *It went from being just needs-based communication to actually interacting and being interested in conversations.* |
| 707 | *So, when we started with it, she probably only had about 20 words available to her on it…* *And then as she started school, we added more words that they wanted to use in school…They said we use it lots to teach her things. And we’ll do flash cards, and we’ll show her a word and she can find that word on her talker and tell us what that word is.* |

In terms of the timing of changes, four caregivers said improvements occurred over a 1-2 year period; and two caregivers characterized these changes as *slow* or *gradual*. For example, one caregiver (PI:706) said, “*It took practice, but she just kept getting better and better, and we were just working a lot with her on those conversation skills, and we still are… If it's not a consistent working with her, it does take a little while for that new skill…to be quickly and easily accessed*.” Conversely, two caregivers said their child could learn new symbols or ASL sign language within a short time period (e.g. 1-2 weeks or even 1 day). Quotes from these two caregivers included:

- *“So, it has to be a repetitive process. So, if she’s learning something new, we incorporate that in everything that we do all day long. From school all the way carried over. And genuinely, by the next day, she kind of has it nailed down, or she uses it in something, and then it relies on the parent or the caregiver to then remember what we taught her yesterday, or they’ve taught her at school, and carry it through to be sure that we understand how she’s using the sign adequately*.” –PI:704
- *“She’s pretty quick with it. Yeah, I mean we’ll show her a few times where it is. But she’s always very interested when new symbols appear because she knows her device quite well. So, she’ll recognize that something is new. And she’ll often use it quite a bit when it’s new just because it’s new… I would say she probably picks up on the fact that it is what it’s there for [symbol on device] within a week or two. But whether or not she then actually goes on to use it will really just depend on motivation.”* – PI:707

Caregivers said certain factors could impact skill acquisition. For example, one caregiver (PI:704) described the impact of seizures on their child’s communication abilities. They said, “*They [communication skills] stick around, unless we’re having difficulty with seizure activity… as long as there’s not any seizure activity that would cause that lapse, then it’s something that she can gain. But if she’s having difficulty with seizures, and she loses that skill, it’s then many months, if not years, to regain the skill*.”

Another caregiver (PI:702) described how malnutrition affected their child’s eye gaze and alertness. They said, “*So, like her first second year, she would not do any eye gazing with you. It was just like she was always tired all the time. And I think a lot of that had to do with her malnutrition. And then once she got her feeding tube… about a month after, we noticed her having more energy, doing more eye gazing, and paying attention to little things, you know*.”

One caregiver (PI:705) said changes to their child’s normal routine affected their ability to retain abilities related to device usage, saying, “*However, when we started back to school last August, she had a lot of changes happening at one time…She had a new teacher on top of that, she had new outside therapists on top of that, and there was just a lot going on…* *So, with all of that change at one time… I don’t wanna necessarily use the term “set us back” because we do eventually get back to where we were and then beyond, but last year definitely was the most significant difference that any kind of change like that ever made because she went back to rarely wanting to use that talker.”*

Two caregivers described their child’s inability to learn fine motor and communication skills at the same time during skill acquisition:

- “*So, it’s interesting, [interviewer name], we almost felt like she might’ve been developing on one thing, and then maybe pressed pause, and then started picking up on the physical, and just doing both might’ve just been too much*.” – PI:701
- “*[child’s name] will, at times – we call it putting it in her file. If she gets overwhelmed by other things or has to focus on learning other skills, whatever skills she has currently been progressing in, she’ll just plateau for a little while and just kind of table it, and then, we’ll start seeing progress again after a little bit of time*… *Normally, it’s just like we’ll see her just stick to where she is for a while, not necessarily take steps back, but just stay where she is for a while, and then start making progress in that area again. But, what’s typically happening is she’s making progress in other areas*.” – PI:705

Caregivers of individuals with HNRNPH2-related disorders did not discuss regressions in their child’s communication abilities. However, several caregivers (*n=*4) said their child used to have certain words/word approximations and ASL signs that have disappeared over time. All four caregivers said this occurred before the age of six. Examples quotes included:

- “*So, she had a few words that she would usually repeat, and they were usually related to songs. So, she also had learned the sign “More.”… So, I would say that the verbal, the very, very limited verbal that she did have is diminished, and I would say that she had a sign or two, but we never stayed with teaching her sign, so that also is reduced to nothing at this point*.” – PI:701
- “*She never had very many verbal words. So, “Mom” and “Good” have always stuck. She has lost a few over the years*.” – PI:704
- “*[child’s name] did have a few words when she was really young… I guess there were probably two or three words that she did use with pretty good consistency. “Mama” was one, “out” was another, and “up,” and “no.” And she also learned a few signs. She learned “more,” she learned “yes,” she learned “no,” she learned “all done,” she learned “milk,” and she used those pretty consistently for a couple of years. Same with the few words that she had. And then, she had those from around the age of 2 to 4, and then, around the age of 4, the words [and signs] disappeared*.” – PI:705
- *“She had a couple of approximations that she mostly used to do animal sounds because she loves Old McDonalds, right. So, she could do an ah for a bah and an oo for moo and things like that. But those are gone now. She doesn’t have those sounds at all anymore*.” – PI:707

In this group, three caregivers also discussed their child’s fine motor ability. These caregivers specifically mentioned how their child’s fine motor ability interacted with communication, including their ability to utilize different modalities successfully (e.g. ASL, AAC devices). Example quotes included:

- “*So, a lot of the children with [sic] have issues with using all five of their fingers. Their dexterity is a little bit different than most individuals. And so, it can cause a little bit of issues with sign language, because they can’t physically do the number six or nine very well because – or I guess six or eight, really – because it’s thumb to pinkie, thumb to ring finger, thumb to middle finger and thumb to first finger. And so, that isolation of that one movement can sometimes be very difficult*.” – PI:704
- “*She uses eye gaze because she does have problems with proprioception and knowing what kind of force to swipe with or make any choices with her finger. She also has not completely mastered the fine motor skill of using her pointer finger, so, through that year of testing, we found that eye gaze worked best for her with a communication device, and so, as I said, she’s had that for about two years now*.” – PI:705
- “*And at that time, she was only about two, her fine motor skills were also quite weak. So, she had a keyguard on it, so she could lay her whole hand flat on top of the iPad and just kind of poke her finger, her thumb, through the holes to the symbols that she wanted… And probably when she was about 5, we were able to take the keyguard off because she could control her hand well enough to do it without it.”* – PI:707

#### Hearing & Vision Impacts

All seven caregivers reported that their child had no formal hearing impairment. However, one caregiver (PI:702) said their child had tubes placed in their ears at one point, but felt their hearing is not impacted. For example, they said, “*Right now, she has tubes in her ears. They did do a hearing test on her in… November of last year. They did the hearing test, and they said everything was great, that she has no damage or anything like that*.” Another caregiver (PI:703) said their child does not have issues with hearing but said the child talks very loudly and they are not sure why. For example, they said, “*We’ve had her hearing checked out, and she doesn’t have any hearing issues. So, we’re just maybe she just can’t hear herself or hear others, and so, she’s screaming or talking in a loud voice. So, I don’t know what that’s about, but she talks loud. We’re always, “Shh, ouch. That hurts my ears*.”

Six out of the seven caregivers reported that their child had a vision impairment. Impairments included cortical visual impairment (*n=*4 confirmed, one suspected), astigmatism (*n=*1) and nearsightedness (*n=*1). Only the caregivers of children diagnosed with CVI reported impacts on communication. Caregivers said CVI impacts their child’s academics and ability to use an AAC device (e.g., affects how symbols are programmed, the positioning of the device itself, and the child’s accuracy in choosing the intended symbol). Some caregivers mentioned presenting things at angles, in 3D, and in certain colors/against certain contrast colors (e.g., black) or light is necessary in order for their child to see them. One caregiver (PI:706) also described emotional impacts CVI has on her child. Please see Table S24 for specific quotes.

Table S24. Quotes from caregivers about the impact(s) of cortical visual impairment (CVI) on their child’s communication.

| Subject ID | Quote(s) |
| --- | --- |
| 701 | *So, depending on the positioning of it [the AAC device], I do think that it’s hard for her to perceive it well. So, sometimes she has mishits. So, she might be called to say something in an activity that they’re practicing certain buttons in that school, and if she has a mishit, it actually produces a different word than the one that she was looking for.*  *…although everybody might want to be a little bit judgmental about the overuse of iPads, for [child’s name], like even in the school setting for academic things, if you show her a picture on a piece of paper, it means very little. But if you present the same image to her with backlight on an iPad, she actually can attend to it better because of her CVI.* |
| 704 | *Everything that you present, it has to be presented on her left side at an angle with a high contrast color in the background. Preferably black. And then, it has to be presented in either red and white bubble lettering, she can see yellows very well… So, with that [AAC] device, I can alter the colors on one of the apps to make it more visual on some of the words for her.*  *And then, if you present a new object to her, it needs to be in 3D. So, if I was trying to teach her some new animal… I would have to physically have a replica of what it looked like in 3D to hand her, and then describe it… the touch, the vision all helps the process.* |
| 705 | *She has cortical visual impairment, and I really strongly believe that one of the primary reasons why we saw such a surge in her communication, and her ability to use her communication device, and her ability to answer questions at school and stuff is because we got this visual impairment diagnosis and made huge modifications to how things are presented to her, and to her device as well, to modify that for CVI so she can see things better…we started… immediately making modifications at home, and with everything that we do with her and on her talker… I’ve made the background on everything and every button black, I’ve outlined every button in red, if we have pages where all the buttons are not filled, I black everything else out and I try to spread the icons out…*  *We’ve had to change to whether does it look like she’s able to access the talker better on her right side or her left side, so we’ve had to change where we’re putting it sometimes... Also, using lit backgrounds helps a lot, so we have a lightboard that we use – we’ll use that for math, we have little cubes that we set on the lightboard that she can easily see and count.*  *…when we’re presenting something for the first time, it needs to be presented on a black background…and preferably slanted because she has a lot of issues, the most issues with her lower field of vision… and then, try to present any new or novel idea or object in 3D first, then a correlating 2D that’s outlined in red so that she can make that correlation.*  *When making choices for answers, again, it’s how it’s presented, and that can change with cortical visual impairment, so we have to make sure we’re paying attention to – does it look like she’s having trouble seeing? Is she always just reaching with her left hand to choose what’s on the left and totally ignoring the other two options that are in front of her? So, we’ve had to make modifications at times, noting that, and then hold them even higher. You try not to do it on each side of your face because faces are very complex and very hard for her, but if you’ve got black background on either side of your face and that’s where you’re putting the choices, that makes it even easier for her to see if it’s up high as well.* |
| 706 | *So, with her CVI she has a difficult time processing everything that's coming in, and so if there's too much sensory coming in through the eyes… it can overwhelm. And then she will go into this place of just shutting down completely, and she'll close her eyes, and she just blocks everything out… and she can't see what's around her, so then she's only relying on hearing, which then there's a lot of sensory with the hearing... And so she will cling to my side if we are out in public and just kind of get very anxious and stubborn about what she wants to do because she just needs to sit and decompress a little, if we're at home she'll remove herself from a room and go sit on the couch by herself and either cover her face or something and just kind of rock until everything subsides and she comes back and her vision is restored. But in that process she is not able to really – she forgets a lot of the skills that we've learned with communication – so if I ask her, "[child’s name], what's wrong?" she is not able to give me words, she will just shake her head and say, "No, no," she just can't talk much. Or she'll say, "Just go away," "She needs to go away." And she refers to herself as she often, so she'll just tell me that, "She needs to go away," and so I usually let her be…* |

#### Meaningful Change

All caregivers during the interviews were asked, “*What would a meaningful change in communication look like for your child?*” If time permitted, most caregivers were asked “*If your child’s communication skills were to decline or get worse, which communication skills would be most important for your child to retain?*”

In general, parents interpreted “meaningful change” to mean “improvements” in communication ability or skills. In addition, some parents made the distinction between realistic and unrealistic goals they had for their children. Four parents of children with HNRNPH2-related disorders said that it would be meaningful for their child to communicate if they were in pain or if they were sick (*n=*4) and three caregivers spoke about their child being able to communicate preferences (i.e. wants/needs; *n=*3). Other responses can be found in Table S25.

Table S25. Responses to “*What would a meaningful change in communication look like for your child*?” from caregivers of children with HNRNPH2-related disorders.

| Meaningful Improvement | 701 | 702 | 703 | 704 | 706 | 707 | Total |
| --- | --- | --- | --- | --- | --- | --- | --- |
| More words using verbal speech |  |  | 1 |  | 1 |  | 2 |
| Communicate if need help/ in danger |  |  |  |  | 1 |  | 1 |
| Initiate communication with other people (show interest in others) using any modality (words, AAC) | 1 |  |  |  |  | 1 | 2 |
| Express feelings/emotions or using any modality (words, AAC) | 1 |  |  |  |  | 1 | 2 |
| Communicate pain or illness using any modality (words, AAC) | 1 | 1 |  | 1 |  | 1 | 4 |
| Improved communication to reduce frustration (when other people do not understand) |  | 1 |  |  |  |  | 1 |
| Start using verbal words |  |  |  |  |  | 1 | 1 |
| Start using complete sentences using words |  |  |  | 1 |  |  | 1 |
| Engage in back-and-forth conversation with another person |  |  | 1 |  | 1 |  | 2 |
| Communicate preferences |  | 1 |  |  | 1 | 1 | 3 |
| Communicate toileting needs |  |  |  | 1 |  |  | 1 |
| Improved literacy/ability to read |  |  |  |  | 1 |  | 1 |

*705 was not asked question due to limited time

One caregiver (PI:704) spoke about her experience supporting her daughter’s communication, saying, “*But I think sometimes with children, you present it to them once, maybe twice, and then they remember, and they carry it forward, and then they use it in a complete sentence, and you just move on with life, and you don’t really realize how blessed you are that everything’s perfectly normal. I think that would be the most meaningful part, is that when she did communicate, I could tell her once or twice. It would stick, she could use it in a sentence, and we could move on, instead of being hung up on something, and it taking days to understand…Probably because as her caregiver, it’s tiring and taxing, and I have to be on my A game. I had to go back to college and take two years of American Sign Language just to be able to adequately teach her, and then understand. So, it takes a huge requirement, and basically life change on top of that to be able to teach your child a way to communicate that they can physically do and understand.”* The caregiver went on to talk about how limited communication abilities is an invisible disability, saying, *“You can build ramps, you can make physical adaptations, but when it’s communication, it’s not an easy adaptation for the children to function throughout society.”*

Another caregiver (PI:706) summarized what would be a meaningful change as, *“I would like – meaningful change for her would be being able to see her more independent for herself – and just some of that autonomy that she just doesn't get with her diagnosis right now, but it would be great to see her make some breakthroughs where she could have a little more autonomy throughout her day and in her decisions that, "This is something [Child’s name] wants to do, that [Child’s name] is deciding," or "[Child’s name] is evaluating and making a choice about."*

In terms of skills that would be important to retain if skills were being lost, the three caregivers who were asked this question mentioned gestures (*n=*3), receptive language skills (*n=*1), and words (*n=*1).

### Hunter syndrome

#### Demographics

Ten caregivers of individuals with Hunter syndrome participated. The majority were female (90%) and on average 37.3 years of age (Table S26). Three caregivers self-identified as Hispanic-Latino. Nine caregivers identified as white and one as African American/Black. Most caregivers (9/10) reported that they were currently married or living with a domestic partner, with 1 caregiver indicating they were single/never married. Overall, the sample reported high levels of education, employment (7/10 employed at least part-time), and annual income (Table S26).

Table S26. Demographic information for 10 caregivers of individuals with Hunter syndrome.

| *Caregivers* | *n (%)* |
| --- | --- |
| Female | 9(90.0) |
| Age, years (Mean/SD) | 37.6/7.4 |
| Ethnicity |  |
| Not Hispanic or Latino | 7(70.0) |
| Hispanic-Latino | 3(30.0) |
| Race |  |
| White | 9(90.0) |
| African-American or Black | 1(10.0) |
| Asian | 0 |
| Middle Eastern | 0 |
| More than one race | 0 |
| Relationship status |  |
| Single, never married | 1(10.0) |
| Married, or living with domestic partner | 9(90.0) |
| Separated | 0 |
| Divorced | 0 |
| Widowed | 0 |
| Highest grade in school |  |
| Less than high school diploma | 0 |
| High school degree or equivalent | 1(10.0) |
| Some college/University | 2(20.0) |
| College/University degree | 3(30.0) |
| Postgraduate degree | 4(40.0) |
| Occupational status |  |
| Homemaker | 3(30.0) |
| Unemployed | 0 |
| Retired | 0 |
| On disability | 0 |
| On leave of absence | 0 |
| Full-time employed | 5(50.0) |
| Part-time employed | 2(20.0) |
| Full-time student only | 0 |
| Income of U.S. residents |  |
| Less than $20,000 | 0 |
| Between $20,001 and $40,000 | 1(10.0) |
| Between $40,001 and $60,000 | 1(10.0) |
| Between $60,001 and $80,000 | 2(20.0) |
| Between $80,001 and $100,000 | 1(10.0) |
| Between $100,001 and $250,000 | 4(40.0) |
| Between $250,001 and $500,000 | 0 |
| $500,000+ | 0 |
| I prefer not to answer | 0 |
| I do not know | 1(10.0) |
| Relation to child |  |
| Mother/Step-mother | 9 (90.0) |
| Father/Step-father | 1 (10.0) |

Caregivers also provided demographic information for their child, all who had the severe form of Hunter syndrome. All children were male, with similar rates to caregivers in regards to ethnicity and race (Table S27). Three caregivers reported their children had an ASD diagnosis, and one reported that their child had epilepsy. Many children were receiving therapeutic services including physical, occupational, and speech therapy (Table S27). Half of the caregivers reported that their child used a high-tech AAC device. The settings in which the device was used varied (Table S27). The mean age of the child when the device was introduced was 5.0 years old (*SD* = 3.7).

When asked about their child’s verbal language, one caregiver indicated their child used ‘full sentences’, three indicated that their child used ‘short phrases’, three indicated their child used a ‘few words’, and three indicated their child used ‘no words’.

Table S27. Demographic information for boys with Hunter syndrome.

| *Children* | *n (%)* |
| --- | --- |
| Age, years (Mean/SD) | 9.0/4.1 |
| Child gender Male | 10(100.0) |
| Ethnicity |  |
| Not Hispanic or Latino | 7(70.0) |
| Hispanic-Latino | 3(30.0) |
| Race |  |
| White | 9(90.0) |
| African-American or Black | 1(10.0) |
| American Indian/Alaska Native | 0 |
| Asian | 0 |
| Middle Eastern | 0 |
| Native Hawaiian/Other Pacific Islander | 0 |
| More than one race | 0 |
| Child’s genotype |  |
| Severe | 10(100.0) |
| Autism Spectrum Disorder (ASD) | 3(30.0) |
| Epilepsy | 1(10.0) |
| Types of therapy |  |
| Physical Therapy | 6(60.0) |
| Occupational Therapy | 10(100.0) |
| Speech Therapy | 10(100.0) |
| Other therapy  Audio/Hearing Therapy  Behavioral Therapy  Enzyme Replacement Therapy  Feeding Therapy  Hippo Therapy  Vision Therapy | 2(20.0)  1(10.0)  4(40.0)  1(10.0)  1(10.0)  1(10.0) |
| Enrolled in Clinical Trial | 3(30.0) |
| Age start speech therapy (Mean/SD) | 2.6/1.0 |
| Age first introduced to device (Mean/SD) | 5.0/3.7 |
| AAC device |  |
| No | 5(50.0) |
| Yes | 5(50.0) |
| Device |  |
| High tech | 5(50.0) |
| Low tech | 0 |
| Places to use the device |  |
| Home | 3(30.0) |
| School | 3(30.0) |
| Out in the community | 0 |
| Other  Therapy | 1(10.0) |

#### Typical Communication Ability

The results for this section are organized by concepts that correspond to expressive, receptive, and pragmatic (i.e. social) communication, aligning with the conceptual framework that was developed for individuals with Angelman syndrome.^1^ Please note, although counts are reported to indicate salience of each theme, these data are limited as they do not indicate all communication behaviors relevant to each child, only the ones that caregivers discussed during the interview.

All 10 caregivers of boys with Hunter syndrome discussed expressive and receptive communication concepts and almost all (9/10) mentioned at least one pragmatic/social communication concept during concept elicitation (Table S28).

Table S28. Number of caregivers that mentioned one or more examples of expressive, receptive, and/or pragmatic communication.

| Communication Behaviors | *n* |
| --- | --- |
| Expressive | 10 |
| Receptive | 10 |
| Pragmatic (Social) | 9 |

##### Expressive

All 10 caregivers of boys with Hunter syndrome discussed expressive communication concepts. Requesting was the most common communication concept mentioned by caregivers (Table S29).

Table S29. Expressive communication concepts on the ORCA measure mentioned by caregivers of boys with Hunter syndrome.

| Expressive Communication Function | *n* (total *n=*10) |
| --- | --- |
| Seeking Attention | 6 |
| Directing Attention | 2 |
| Refusing an Object | 9 |
| Requesting an Object | 10 |
| Requesting an Object out of View | 4 |
| Requesting More | 3 |
| Asking Questions | 2 |
| Telling Stories | 2 |

Caregivers of boys with Hunter syndrome mentioned a number of modalities that their children used to **make requests** including words/word approximations (*n=*7), gestures/signs (e.g. finger point, American Sign Language, modified gesture/signs; *n=*6), physical body movements (e.g. taking an object to a caregiver; *n=*4), AAC devices (*n=*3), and sounds (e.g. ‘ah’; *n=*1). Examples of requesting included:

- “*He says, “I want a snack.” And then he’ll tell me exactly what he wants, like a Rice Krispy, or pretzels, or apple. So, I mean, he’s got a pretty good vocabulary.”* – PI:301
- “*And then, he pulls you to the fridge, and he points – point, point, point – over and over, points–to what he wants. Or, if he wants you to change his episode on that tablet, he’ll grab your hand, and put your hand on top of the tablet, and tap it with his finger*.” – PI:302
- “*And so, on his [device] he has familiar pictures, that include ‘bathroom’, ‘yes’, ‘no’, ‘drink’, ‘eat’, ‘school’, ‘mom’, ‘people’, and he will navigate that pretty fluidly and pretty consistently.*” – PI:303
- “…*if he’s hungry, he kind of just gravitates towards our snack cupboard or our refrigerator. There is no communication in regards to him showing you a picture or him telling you what he needs or wants*.” – PI:310

Three caregivers reported that their children could request **more of something.**  Two reported that they used the American Sign Language (ASL) sign for “more” with one child also being able to say the word “more”.

Six caregivers reported that their children were able to **seek attention**. Examples included:

- “*Sometimes, he’ll make sounds just to kinda get his point across, like “rah, rah, rah, rah, rah” or* *“ah, ah, ah, ah, ah.” Not really trying to say anything, but just vocalizing. Like, “Hey, pay attention.”* – PI:302
- “*So, you know, when he’s really tired and exhausted sometimes he may not have the ability to process, you know, “I wanna play, I want attention.” So instead he’ll come and, you know, hit me or he might push me or pull me rather than say come here. So, just being a little more physical when he’s not able to communicate verbally*.” – PI:304

For **directing attention**, two caregivers described how their children would use verbal language to direct attention, then described how the child would follow that action with pointing and pulling the caregiver if the caregiver did not understand what the child was trying to show them (or did not come quickly). For example, one caregiver described (PI:306): *Usually his hands, like to point, because if we don’t understand him, he’ll just say, “Look! Look!” And he’ll pull us in and show us what he’s talking about.*

Nine caregivers mentioned that their children could **refuse objects or activities**. The modalities that caregivers mentioned their child using varied and included words/word approximations (e.g. saying ‘no’; *n=*4), crying/fussing/whining (*n=*2), physical removal of body from the situation (e.g. walking away; *n=*2), throwing an object away (*n=*2), head shaking to indicate ‘no’ (*n=*10), pairing a word with a gesture (e.g. saying ‘no’ and pushing an object away; *n=*1), and AAC device to indicate ‘no’ (*n=*1). The caregiver (PI:303) that described her son using an AAC device to indicate ‘no’ had a child who had regressed/lost verbal words at this point in the disease. Example quotes around refusal included:

- “*What else –and then if there’s any resistance or any dislike for something, he’ll communicate that with you by making a noise like maybe a grunt or a whine of some sort*.” – PI:310
- “*…..we have a sitter coming today, so he’s like, “No, I don’t want…” And he’ll say whoever’s name, “I don’t want her.” And he’ll let us know who he wants [instead].” –* PI:301
- “*I know his teacher which – this is something that typically doesn’t happen but it has – where they’re asking him to do something and he doesn’t want to, so rather than telling them he doesn’t want to do it he may throw the paper, crayons on the floor, you know, to get away from it*.” – PI:304

Two caregivers of boys with Hunter syndrome discussed how their children **tell stories.** Both children were 8 years old. One caregiver (PI:304) described how her child told stories through participation in pretend play with toy characters. For example, they said, “*Like, he’ll be like, “I’m mamma Pig.” “Oh, come here Peppa, get in the car.”* This child was also able to tell stories in the past. For example, telling the caregiver that he played with his brother: *“But he can tell me, “I played with [name]” or something like that.”* The other caregiver (PI:306) explained that sometimes, the child will tell his teachers what he did at home, or on his days off from school. The caregiver did not specify what modality the child used to tell these stories, but words/word approximations was indicated by the caregiver as the child’s primary modality.

Two caregivers described how their children use words to **ask questions** related to their surroundings and/or needs. One caregiver (PI:301) described how her 12-year-old child has recently been able to ask a simple question. For example, they said, “*And then sometimes, he will ask, “Can I have water?”* The other caregiver had a 6-year-old child and described how the child asks complex questions about what he sees on TV shows, and generally what people are doing. For example, they said, “*Also, [the child asks] questions about what he’s watching, like, “What are they doing?” “What are they wearing?”* The caregiver explained that this child also asks “*How?”* questions and tries to have conversations with people he knows. For example, he says, *“Hi! What are you doing?”*

##### Receptive

All ten caregivers of boys with Hunter syndrome mentioned receptive communication concepts during concept elicitation. Examples of receptive communication for this group included making choices (*n=*4), following familiar one-step directions (*n=*1), following familiar two-step directions, (*n=*1), understanding isolated words or phrases (*n=*4), answering simple questions (*n=*5), answering more complex questions (“why” or “how”; *n=*1), and responding to their name (*n=*1). Example quotes included:

- One caregiver (PI:301) gave examples of 1) two-step directions her son can follow, 2) how her son understands signs/gestures, and 3) answering questions.
- *“Take off your pajamas, and go put them in your dirty clothes.” He’ll take it off and go put it away. Or “Go put your plate in the sink, and then go watch TV.” Or something.”*
- *“So even if he tried to sign, he can’t, because physically he’s not able to. So, if there’s a sign we can use, he understands it. So, he can use it more receptive, than actually expressive [Signs/gestures such as] I don’t know that we’re using them correctly, but either just ‘stop’ or ‘be quiet’, he understands that.”*
- *“And like yesterday, he had speech, and I was actually really surprised with a couple of the things, because she had pictures. And it was like, “What is this person doing?” And it was like, “Okay, well the baby’s sleeping.” And she went further, like, “Why?” And then he said, “Because the baby’s tired.”*

Four caregivers described how their child **made choices**. Two caregivers (PI:301 & PI:304) said that their children made choices by verbally naming the specific item they wanted. For example, one caregiver (PI:301) said, *“He will tell me when he wants to eat, for example. He basically gives me the menu for the day.”* One caregiver (PI:302) discussed how their child used a high tech AAC device to choose between different options and the impact the ability to make that choice had on their child’s mood*.* For example, they said, *“Because if you show it to him – a picture of a popsicle or a banana – he’ll tap, tap, tap on the button that he wants. And then, you give him – say it’s the popsicle – and then, he's happy.*” Finally, one caregiver (PI:307) discussed how their child would choose what they want to eat from a given list. For example, they said, *“So, if he’s in the pantry I’ll ask him “Are you hungry?” and he’ll usually say yes, then I’ll ask him if he wants a snack and he’ll say yes and then, I’ll give him some choices of a snack and he’ll pick one.”* Caregivers reported that their children made choices about food (*n=*3), shows or movies (*n=*1), and clothes (*n=*1).

Two different caregivers discussed what receptive language looks like as the disease progresses. For example, one caregiver (PI:303) said, “*And I feel like when [child’s name] regressed and lost the ability of language, I felt like he…did not lose that receptive language skill. He still wanted to be spoken to*.” Whereas another caregiver (PI:305) described how her son repeats the last word he hears, *“Maybe it’s different based on where somebody is in the progression of the disease – but [child’s name] is at the point in the disease where if I say “What do you want for breakfast?” he will try to repeat the last word that he heard.”*

##### Pragmatic

Nine caregivers of boys with Hunter syndrome described pragmatic/social communication concepts during concept elicitation. Examples given by caregivers included greetings (e.g. “hi” or “bye”), apologizing appropriately (without prompting), using “please” when making requests, using names (e.g. people or places), and engaging in pretend play. The modalities that caregivers reported their child using for pragmatic communication included words/word approximations (*n=*8), gestures/signs (*n=*1), and AAC devices (*n=*2). Note, some children could use multiple modalities.

One mother (PI:304) gave an example showing her son using multiple pragmatic skills in one sentence. To request food, he might say “*Mom, I’m hungry. I want cereal, please*.” In this example, the child addresses the caregiver by name while saying please and informing her of a request. Another caregiver described how the child will greet his friends at school using words. Lastly, a third caregiver whose child had started to experience a regression in skills stated that her son will sign “sorry”.

The majority of Hunter syndrome caregivers (8/12) said their child used verbal speech (e.g., words/word approximations) or an AAC device to name someone; most commonly their mom, dad, siblings, or other caregivers. Examples included:

- “*There are times when he knows what he wants, so he’ll tell me, “Mom I want cereal, I’m hungry.” There are times when he’s tired and he’ll tell me, “Mom I’m tired.”* – PI:304
- “*He mostly will call for Mama or Dada. He calls his dad Bubba*.” – PI:309
- *“[The child uses] names of friends in his class, neighbor’s names and their dog’s names*”. – PI:311
- “*Whenever he pushes on the [AAC device] button, it says “Mommy.” So, he has one of those, and he does well with it when we’re consistent with it*.” – PI:302

#### Changes in Communication

All 10 caregivers whose sons had Hunter syndrome discussed how their child’s communication changes overtime. During those discussions, some of the caregivers discussed when the changes occurred, whether the changes were fast or slow, and what happened to communication skills once they were learned. In contrast to the other groups within Cohort 1, over half (*n=*6) of the caregivers described **regressions** that occurred in their child’s communication. The other four caregivers described only improvements they have seen in their child’s communication.

Four out of the six caregivers said the **regressions** happened between 8-10 years old, while two caregivers (PI:302 and PI:310) said the regression happened at ages 3 and 4-5 years old, respectively. All but one caregiver of a teenage child said their son had experienced a regression in their communication. Three other caregivers of children aged 4, 5, and 9 also discussed regressions. All caregivers described regression in terms of losses in verbal speech (e.g., words or word approximations) and said the changes occurred quickly (within one year). Example quotes of regressions included:

- “*He lost most of his words approximately a year ago. Before that, he only had had 10 to 20 words at any given time. But like I said, since about a year, he’s lost all of his words.”* – PI:302
- “*Yeah, so he was down to probably five words consistently, in 2016. And then, it was down to two words, mid 2016. And then, by the end of 2016 he had no verbal language*.” – PI:303

PI:303’s child also lost motor skills and began experiencing feeding difficulties during the regression. While some caregivers (*n=*3) described their child losing all verbal speech (as above), some caregivers (*n=*3) said their child could still use single words or word approximations. Example quotes included:

- “*I think his words are just basically one-word utterances now, mostly labeling. And they’re right; he’s right. He’s not saying the wrong thing. It’s just he did used to say more like 10-word sentences*.” – PI:311
- “*Definitely less words, less sentences [now]. At one time, he was really just getting speech therapy for pronunciation, so he almost caught up to his language, and then, since then, he’s back down*.” – PI:307

These two caregivers said although their children still have some verbal language, they recently started experiencing issues with echolalia and perseveration. For example, one caregiver (PI:307) said, “*We do a lot more echolalia right now, a lot more random phrases, I feel like he gets stuck on words a lot more and it’s just very repetitive.”*

The four caregivers that described **improvements** in their child’s communication provided a range of different changes they have seen, including improvements in receptive language (*n=*3), pronunciation (*n=*2), and vocabulary (*n=*4; Table S30). Three out of the four caregivers said the most changes occurred between ages 2-5 years. Two caregivers (PI:301 & PI:309) felt speech therapy likely contributed to the improvements and one caregiver (PI:306) said the changes happened quickly when their child enrolled in a clinical trial. For example, the caregiver said, “*Well, he just got into a medical trial last August and ever since, I feel like it’s been improving a lot, ‘cause he wasn’t really verbal. And in this past year, he has started using a lot of words, and just asking a lot of questions*.”

Table S30: Examples of improvements in communication ability.

| Participant ID | Quote |
| --- | --- |
| 301 | *I would say definitely, the vocabulary has increased. And then the pronunciation has gotten better. And then, it’s been more complete sentences. Also, his receptive has gotten better, in terms of answering questions.* |
| 304 | *So, he’s still learning new words and he’s still saying new phrases.* |
| 306 | *In this past year, he has started using a lot of words, and just asking a lot of questions.*  *The way he would say the words, I don’t know if it was… I don’t know. But he started saying it more clear.* |
| 309 | *I would say we really started to notice his receptive language picking up a lot probably around the time he turned 2. So, that was about a year ago is when we started to really notice that. And that was probably around the time too where he also started to say certain words at least in his own way, whether they’re totally perfect or not, he says certain words.* |

Caregivers who discussed the **speed of improvements** in their child’s communication felt that they occurred slowly. For example, one caregiver (PI:304) said, “*So, they have improved. You know, it’s taken quite a while for it to improve, so it’s like slow improvement*.” Two caregivers felt their child’s communication plateaued around age 6, but have continued to improve slowly. Caregivers said learning or maintaining a new skill requires repetition and modeling (Table S31).

Table S31. Examples of repetition with skill development and retention.

| **Participant ID** | **Quote** |
| --- | --- |
| 301 | *And there’s a lot of maintenance still that we do. We do a lot of speech to make sure he doesn’t lose the skills that he has.* |
| 303 | *Takes a lot of repetition, and a long time. And it had to be motivating for [child’s name]. Visually stimulating, too. So… the things that have stuck with [child’s name] come from that piece of how he was learning, through visual and repetition.* |
| 304 | *And there are some skills that are a lot more difficult to acquire. So, like I said if it’s not consistent and repeatedly done then it might be difficult for him to keep the same – the same skill level.* |
| 307 | Interviewer: *Once a new communication skill is learned, does it usually stick around?*  Interviewee: *If it’s practiced daily and across multiple environments, yes. We’ve been working really hard on the yes/no questions, that has seemed to stick across the board, but we have both school, private speech, and home working on it.* |

Finally, like with other groups in cohort 1, a couple of Hunter syndrome caregivers (*n=*2) said that skills are variable and inconsistent, and other skills may disappear when their child is trying to learn a new skill. For example, one caregiver (PI:309) said, “*He kind of has thing in general with all of his skills where he’ll start to do something new. And then, for a little while he’ll do it. And then, he’ll kind of stop doing it. But he’ll start to do something else new, and then later, maybe a few months later, he’ll bring back the old skill too. So, it’s kind of like, if he’s learning something new, he can’t focus on a bunch of new things at once. So, it’s almost like his brain’s like okay, we did that. Put that on pause. We’re gonna focus on something new now. And then a little bit later, we got that. Now, we can bring it all back to use it consistently*.”

#### Hearing & Vision Impacts

Seven of the 10 caregivers of sons with Hunter syndrome reported that their child had a hearing impairment. Caregivers did not name specific hearing impairments; instead, they described the severity of the hearing problem (e.g., mild, moderate, or severe). Similarly, caregivers mentioned whether the hearing problem was occurring in one ear or both ears. Frequencies can be found in Table S32. The presence of a hearing impairment was more frequently reported for older children, likely representing disease progression.

Table S32. Specific details around hearing impairments as reported by caregivers of individuals with Hunter syndrome.

|  | Age 1-5 (*n=*3) | Age 6-8 (*n=*2) | Age 9-11 (*n=*1) | Age 12-18 (*n=*4) | Total  (*n=*10) | Participant ID |
| --- | --- | --- | --- | --- | --- | --- |
| Hearing Impairment |  |  |  |  |  |  |
| Mild |  | 2 |  |  | 2 | 304, 306 |
| Mild-Moderate |  |  |  | 2 | 2 | 301, 311 |
| Severe |  |  |  | 2 | 2 | 303, 305 |
| Wears hearing aids |  |  | 1 | 2 | 3 | 301, 303, 307 |
| Difficulty in getting child to wear their hearing aids |  | 1 |  | 2 | 3 | 304, 305, 311 |
| Vision Impairment |  |  |  |  |  |  |
| Nearsighted |  |  | 1 | 1 | 2 | 301, 307 |
| Mild CVI | 1 |  |  |  | 1 | 309 |

As noted in Table S32, three caregivers (PI:301, PI:303, PI:307) said their child wore hearing aids. Three other caregivers (PI:304, PI:305, PI:311) said their child needed hearing aids, but there were behavioral issues that impacted successful wearing. Examples of quotes about behavioral issues included:

- “*So, he does have hearing aids that were prescribed to him about eight months ago? But it’s been very difficult to get him to wear it*.” – PI:304
- “*But the battle to keep those [hearing aids] in was just not worth the overall outcome for him in terms of the development anyway, so I did not push that fight*.” – PI:305

All but one caregiver (PI:304) whose child had a hearing impairment said the hearing impairment impacted their child’s communication ability. Four caregivers with children with mild-moderate hearing loss mentioned the hearing impairment impacted their child’s pronunciation because they cannot hear certain pitches or high frequency sounds. Another caregiver (PI:305) of a child with severe hearing loss said the hearing loss impacted their child’s receptive language. This impact on receptive language was echoed by another caregiver who reported severe hearing loss in their child. Some example quotes included:

- “*He’s not able to pronounce certain words, or they’re just harder… Like high frequency S and F, and Z. Sometimes it’s harder to understand him. So, over the years, he’s gotten a little bit better, but like ‘pizza’ used to be ‘zizza’. So, there’s a few words like here and there, that it’s harder for him to say*.” – PI:301
- “*I feel like the hearing it –it really doesn’t impede his communications in general, but I know when it comes to grammatical things, he doesn’t –he has this high frequency sounds like Ss and Fs, so we don’t really get plurals or possessives because he’s not hearing that S*.” – PI:307
- “*I let him guide what things are and what they mean to him, but he can get agitated or frustrated if I don’t understand what he’s asking for or what he means and if he’s not understanding a direction that’s being given to him because he doesn’t understand the language*.” – PI:305

Three of the ten caregivers reported that their child had a vision impairment. Of these, two (PI:301 & PI:307) reported that their child was nearsighted and one (PI:309) reported that their child had mild cortical vision impairment (CVI). One other caregiver (PI:302) said, “*there is some sort of vision impairment going* *on”*, but did not have an official diagnosis because the child did not cooperate during their exam. None of the caregivers felt the vision impairment impacted their child’s communication ability.

#### Meaningful Change

All caregivers during the interviews were asked, “*What would a meaningful change in communication look like for your child?*” If time permitted, most caregivers were asked “*If your child’s communication skills were to decline or get worse, which communication skills would be most important for your child to retain?*”

Parents interpreted “meaningful change” to mean “improvements” in communication ability or skills. In addition, some parents made the distinction between realistic and unrealistic goals they had for their children.

Caregivers of children with Hunter syndrome reported several examples of meaningful improvement, with some caregivers reporting more than one (Table S33). Caregivers discussed children acquiring different modalities (e.g. pointing, sounds, speech), but also functional skills (e.g. expressing emotions). Three caregivers in this group also mentioned that it would be meaningful for their child to advance in communication so that they could carry on a conversation with another person outside of the family. Caregivers in this group listed more complex expressive communication skills including, better pronunciation, and engaging in a conversation with a person outside of the family, when compared to the other groups in Cohort 1. These goals likely represent the fact that this group is mostly already using verbal words, and short verbal phrases.

Table S33. Responses to “*What would a meaningful change in communication look like for your child*?” from 10 caregivers of boys with Hunter syndrome.

| Meaningful Improvement | 301 | 302 | 303 | 304 | 305 | 306 | 307 | 309 | 310 | 311 | Total |
| --- | --- | --- | --- | --- | --- | --- | --- | --- | --- | --- | --- |
| Verbal words return |  |  |  |  |  |  |  |  | 1 |  | 1 |
| Better communication to reduce frustration/behavior |  |  |  |  |  | 1 |  | 1 |  |  | 2 |
| Improve pronunciation so strangers can understand |  |  |  |  |  | 1 |  |  |  |  | 1 |
| More words |  |  |  |  | 1 |  |  | 1 |  |  | 2 |
| Consistent communication |  | 1 |  | 1 |  |  |  |  |  |  | 2 |
| Make choices |  |  |  |  |  |  |  |  |  | 1 | 1 |
| Start using High Tech AAC |  |  |  |  |  |  |  | 1 | 1 |  | 2 |
| Carry on a conversation with another person outside the family | 1 |  | 1 | 1 |  |  |  |  |  |  | 3 |
| More sentences |  |  | 1 |  |  |  |  |  |  |  | 1 |
| The cognitive ability to understand cause and effect |  |  |  | 1 |  |  |  |  |  |  | 1 |
| Answering questions |  |  |  |  |  |  | 1 |  |  |  | 1 |
| Stability in communication / Stop the loss of skills |  |  |  |  |  |  |  |  |  | 1 | 1 |

Regressions are expected for children with the severe form of Hunter syndrome that were included in this study. In this sample, there were older children who had already started experiencing regressions, as well as younger children who had not. Responses to the question about meaningful skill retention from the perspective of caregivers can be found in Table S34. While the responses included specific modalities (e.g. verbal words, device usage, gestures), parents in this group also mentioned general concepts like expressing needs (however it can be done) and receptive understanding.

Table S34. Responses to “*If your child’s communication skills were to decline or get worse, which communication skills would be most important for your child to retain*?”

| Worsening Communication – Skills important to retain | 301 | 302 | 303 | 304 | 305 | 306 | 307 | 309 | 310 | 311 | Total |
| --- | --- | --- | --- | --- | --- | --- | --- | --- | --- | --- | --- |
| Gesture |  | 1 |  |  |  |  |  |  |  |  | 1 |
| Receptive Communication ability |  |  |  |  |  |  |  |  |  | 1 | 1 |
| Verbal words | 1 |  |  |  |  |  |  |  |  |  | 1 |
| AAC device usage |  |  |  |  |  |  |  | 1 |  |  | 1 |
| Express needs - any modality |  |  |  | 1 |  |  | 1 | 1 |  |  | 3 |
| Express hunger or thirst |  |  |  |  |  |  |  |  | 1 |  | 1 |
| Going to the thing/taking person to item |  | 1 |  |  |  |  |  |  |  |  | 1 |
| Communicate toileting needs |  |  |  |  |  |  |  |  | 1 |  | 1 |

*303, 305, 306 were not asked this question due to time constraints

### Malan syndrome (NFIX)

#### Demographics

Eleven caregivers of individuals with Malan syndrome participated. The majority were female (10/11) and on average 38.2 years of age (Table S35). Four caregivers self-identified as Hispanic-Latino. Ten caregivers identified as white and one as mixed race. All caregivers reported that they were currently married or living with a domestic partner. Overall, the participants reported high levels of education, employment, and annual income (Table S35).

Table S35. Demographic information for 11 caregivers of individuals with Malan syndrome.

| *Caregivers* | *n (%)* |
| --- | --- |
| Female | 10(90.9) |
| Age, years (Mean/SD) | 38.2/7.4 |
| Ethnicity |  |
| Not Hispanic or Latino | 7(63.6) |
| Hispanic-Latino | 4(36.4) |
| Race |  |
| White | 10(90.9) |
| African-American or Black | 0 |
| American Indian/Alaska Native | 0 |
| Asian | 0 |
| Middle Eastern | 0 |
| More than one race* | 1(9.1) |
| Relationship status |  |
| Single, never married | 0 |
| Married, or living with domestic partner | 11(100.0) |
| Separated | 0 |
| Divorced | 0 |
| Widowed | 0 |
| Highest grade in school |  |
| Less than high school diploma | 1(9.1) |
| High school degree or equivalent | 1(9.1) |
| Some college/University | 3(27.3) |
| College/University degree | 3(27.3) |
| Postgraduate degree | 3(27.3) |
| Occupational status |  |
| Homemaker | 2(18.2) |
| Unemployed | 0 |
| Retired | 0 |
| On disability | 0 |
| On leave of absence | 0 |
| Full-time employed | 7(63.6) |
| Part-time employed | 2(18.2) |
| Full-time student only | 0 |
| Income of U.S. residents |  |
| Less than $20,000 | 0 |
| Between $20,001 and $40,000 | 1(9.1) |
| Between $40,001 and $60,000 | 1(9.1) |
| Between $60,001 and $80,000 | 0 |
| Between $80,001 and $100,000 | 2(18.2) |
| Between $100,001 and $250,000 | 7(63.6) |
| Between $250,001 and $500,000 | 0 |
| $500,000+ | 0 |
| I prefer not to answer | 0 |
| I do not know | 0 |
| Relation to child |  |
| Mother/Step-mother | 10(90.9) |
| Father/Step-father | 1(9.1) |

Note: *White and African-American or Black

Caregivers also provided demographic information for their child with Malan syndrome. Seven children were female (64%), with similar rates to caregivers in regards to ethnicity and race (Table S36). Genotypes varied and included frameshift and deletions (Table S36). Four caregivers reported their children had an ASD diagnosis, and one reported that their child had epilepsy. Many children were receiving therapeutic services including physical, occupational, and speech therapy (Table S36). Three caregivers reported that their child used an AAC device with two reporting their child used a ‘high tech’ device. The settings in which the device was used varied (Table S36). The mean age of the child when the device was introduced was 3.8 years old (*SD* = 0.8).

When asked about their child’s verbal language, two caregivers indicated their child used ‘full sentences’, six indicated that their child used ‘short phrases’, two indicated their child used a ‘few words’, and one indicated their child used ‘no words’.

Table S36. Demographic information for 11 children with Malan syndrome.

| *Children* | *n (%)* |
| --- | --- |
| Age, years (Mean/SD) | 7.3/4.7 |
| Child gender Female | 7(63.6) |
| Ethnicity |  |
| Not Hispanic or Latino | 7(63.6) |
| Hispanic-Latino | 4(36.4) |
| Race |  |
| White | 10(90.9) |
| African-American or Black | 0 |
| American Indian/Alaska Native | 0 |
| Asian | 0 |
| Middle Eastern | 0 |
| Native Hawaiian/Other Pacific Islander | 0 |
| More than one race* | 1(9.1) |
| Child’s genotype |  |
| Chromosomal Deletion | 2(18.2) |
| Deletion | 1(9.1) |
| Deletion - Splice Variant | 1(9.1) |
| Frameshift | 4(36.4) |
| Insertion | 1(9.1) |
| Missense | 1(9.1) |
| Splice variant | 1(9.1) |
| Autism Spectrum Disorder (ASD) | 4(36.4) |
| Epilepsy | 1(9.1) |
| Types of therapy |  |
| Physical Therapy | 8(72.7) |
| Occupational Therapy | 8(72.7) |
| Speech Therapy | 10(90.9) |
| Other therapy  ABA Therapy  Aquatic Therapy  Art Therapy  Developmental Therapy  Equestrian Therapy  Hearing Therapy  Music Therapy  Vision Therapy | 2(18.2)  1(9.1)  1(9.1)  2(18.2)  1(9.1)  1(9.1)  1(9.1)  1(9.1) |
| Age start speech therapy (Mean/SD) | 2.6/1.6 |
| Age first introduced to device (Mean/SD) | 3.8/0.8 |
| AAC device |  |
| No | 8(72.7) |
| Yes | 3(27.3) |
| Device |  |
| High tech | 2(18.2) |
| Low tech | 2(18.2) |
| Places to use the device |  |
| Home | 2(18.2) |
| School | 3(27.3) |
| Out in the community | 1(9.1) |
| Other | - |

Note: *White and Asian

#### Typical Communication Ability

The results for this section are organized by behaviors that correspond to expressive, receptive, and pragmatic (i.e. social) communication, aligning with the conceptual framework that was developed for individuals with Angelman syndrome.^1^ Please note, although counts are reported to indicate salience of each theme, these data are limited in that they do not indicate *all* communication behaviors relevant to each child, only the ones that caregivers discussed during the interview.

All 11 caregivers of individuals with Malan syndrome discussed expressive communication concepts and almost all (9/10) mentioned at least one receptive or pragmatic/social communication concept during concept elicitation (Table S37).

Table S37. Number of caregivers that mentioned one or more examples of expressive, receptive, and/or pragmatic communication.

| Communication Behaviors | *n* |
| --- | --- |
| Expressive | 11 |
| Receptive | 10 |
| Pragmatic (Social) | 10 |

##### Expressive

All caregivers of children with Malan syndrome discussed expressive communication concepts. Requesting was a communication concept mentioned by all caregivers (Table S38).

Table S38. Expressive communication concepts on the ORCA measure mentioned by caregivers of individuals with Malan syndrome.

| Expressive Communication Function | *n* (total *n=*11) |
| --- | --- |
| Seeking Attention | 5 |
| Directing Attention | 8 |
| Refusing an Object | 5 |
| Requesting an Object | 11 |
| Requesting an Object out of View | 4 |
| Requesting More | 5 |
| Asking Questions | 3 |
| Telling Stories | 2 |
| Communicating understanding | 1 |

Five caregivers of children with Malan syndrome discussed how their child **sought attention**. Three caregivers shared that their child used words or word approximations to seek attention. For example, one caregiver (PI:504) said, “*So, if we’re going to go-to a restaurant he’ll say, “Mommy now see I’m hungry*.” Another caregiver (PI:509) said, “*She will use words, she’ll call me by “Mom” or her dad, by “Dad” and she will continue to attempt until she gets a response. Even if it’s interrupting*.”

Another caregiver (PI: 508) reported that their child used signs to seek attention. For example, they said, *“So, like one time he was sitting at the table and he needed help with something, and he actually used the sign “help”.”* One parent (PI:505) described communication behaviors that were not symbolic, but were interpreted by the caregiver as seeking attention. This parent described how their child might use physical gestures (grabbing, hitting, pulling) to seek attention. For example, they said, “*He’ll physically come up to us and grab us and pull on us.”*

Eight caregivers spoke about how their child **directed attention** to an object, action, or event using symbolic and/or non-symbolic communication. Three caregivers reported that their children utilized gestures/signs to direct attention (e.g. finger point), and four indicated their child used words/word approximations. Examples of directing attention provided by parents included:

- “*So, yes, when she shows me stuff, she’s like, “Look, like they’re dancing,” or stuff like that*.” – PI:507
- “*She’s big on pointing out what she sees in her environment lately like if we’re driving, she likes construction vehicles or animals*.” – PI:511
- “*She will, for example, if it’s something on her phone she wants me to see, she will hold the phone in front of my face. She does some pointing, but not a lot of pointing, I would say. Some pointing. So, if there’s like a physical picture she wants me to look at, she will hold it in her hands and put it in front of my face*.” – PI:509
- “*If you use the "Show me," then he's able to take you to whatever he's talking about*.” – PI:506

Five caregivers of individuals with Malan syndrome discussed **refusal.** Three caregivers indicated that their child uses a word or word approximation (e.g. ‘no’) to indicate refusal. For example, one caregiver (PI:508) said, *“So, you know, if he’s frustrated, he’s now saying “nah” or if he doesn’t want something, he’s saying “nah” instead of “no.”* This caregiver (PI:508) described how their child also shakes their head to say no. Another caregiver (PI:509) described how their child verbally says no, saying, ”*She’ll either say, ‘No’ or she’ll say, “I don’t want that.”* Another caregiver (PI:505) said their child uses grunting sounds and a head turn to indicate refusal. For example, they said, “*He either grunts and turns his head away, or he starts to say no in his own words.”*

Three caregivers (PI: 505, 510, 509) described non-symbolic forms of communicating refusal as well. One caregiver (PI:505) mentioned how their child will turn their head away to communicate refusal. Another caregiver (PI:510) mentioned how their child will fuss to communicate refusal. For example, “*Like a typical two-year-old. You know, it’s like fussing and kind of throwing her body around. Like I don’t wanna do this. And diaper changes are a big one.”* Another caregiver (PI:509) explained how their child would physically remove themselves from situations to refuse activities. For example, they said, “*… if it’s something she doesn’t want to do be cause she’s overwhelmed by anxiety like, ‘Let’s go to school’ she’ll respond by saying ‘No,’ [sic] running up to her room, or pulling the covers over her head.”*

All 11 parents discussed how their child communicated **requests** during concept elicitation. The most commonly requested objects were food and drink, but individuals with Malan syndrome in our sample also frequently requested places and activities, like going outside. The majority of caregivers (8/11) said their child used words to request objects (Table S39). The three children with Malan syndrome that did not use words or word approximations to request objects were in the youngest age group. Many caregivers (*n=*6) said their child could self-reference while requesting. Two caregivers also said their child uses manners while requesting. For example, one caregiver (PI:509) said, “*if we order at a restaurant, she’ll say like, “May I please have chicken tenders” to the waitress; to the wait staff*.”

Table S39. Examples of requests using verbal speech from interviews with caregivers of individuals with Malan syndrome.

| Subject ID | Quote |
| --- | --- |
| 501 | *She will typically vocalize and ask in a full sentence for what she wants or what she’s needing, like, “I need a drink,” “I need to use the bathroom,” those kinds of things.* |
| 502 | *So, she'll say, “I'm hungry.” And then she'll go over to the food pantry and pick out her—the item that she wants, she'll point to it or sometimes even grab it.* |
| 504 | *So, if we’re going to go-to a restaurant he’ll say, “Mommy now see I’m hungry.” And he’ll go, “I’m hungry.” And I’ll say, “Okay, well what do you want to eat?” And he’ll tell me, you know, something, cereal or yogurt, apple, or he’ll say a restaurant.* |
| 506 | *So, we have a swimming pool. He'll say, "I want to go swimming," if it's right there. Or, if he sees a ball, he'll say he wants to play soccer.* |
| 507 | *Right now, well, since she already learned how to use the “I want,” it’s usually “I want,” and then she tells me like “the iPad” or “I want the Play-dough,” or “I want markers.”* |
| 509 | *She’ll also say, “Can I have.” “Can I have ice cream,” for example.* |

Many caregivers (*n=*7) also discussed gestures/signs (e.g. ASL sign, modified gesture/sign, finger point) their child used to request objects. For example, one caregiver (PI:501) said, “*So, she typically will do simple, one-word signs like “more,” “please,” “thank you.” Sometimes, she’ll just do “drink,” or “food”.* Another caregiver (PI:508) said, *“You know, like he understands like dinner routines, so he’ll actually point to a napkin, like he needs a napkin. He understands that there’s something on his face, and so he’ll wipe his face off.”* One other caregiver (PI:510) said, *“Like she likes to be carried around and she'll point you in the direction where she wants you to go towards the objects she wants.”*

Only one caregiver (PI:504) said their child used an AAC device to request. They said, “*So, if he says a restaurant well, where do you want to go and then he’ll try to vocalize or verbalize it and if I don’t understand he’ll get his AAC device and I have added those icons, those buttons to his device so he’ll tell me and then I’ll ask him, “So, what do you want to eat from there?” And he’ll go-to his food folder and tell me chicken nuggets, French fries, milk. And I’ll say, “Oh, what else do you want?” And he’ll continue*.” Similarly, only one caregiver (PI:502) described how their child used sounds to request. For example, they said, *“And then there is some times where she can't communicate well with her words. And she'll either point or try to make a noise to get you to go where she wants you to go or do what she wants to do.”*

When describing requesting, five parents described communication behaviors that were not symbolic, but were interpreted by the caregivers as requesting. For example, these parents described how sometimes their child might use physical gestures (e.g., taking the caregiver to an object or bringing an object to the caregiver) to request. For example, one caregiver (PI:510) said, “*When she's hungry… We have a cabinet in our kitchen that she specifically knows to go to.”* Another caregiver (PI:505) said, “*He usually pulls at us to go in the direction that he wants us to, or he’ll stand wherever the item or whatever he’s looking for, and then kind of look at it until we come that way and give it to him*.” These caregivers all had younger children between the ages of 2-6.

Five caregivers discussed how their child **requested more of something**. Four out of the five said their child used the ASL sign for “more”. One child (PI:507) could also request more of something using words.

Four caregivers discussed how their child **requested objects that they could not see** using symbolic communication (e.g., words, device, gestures, and signs). All of these caregivers said their child used words to request objects out of view. For example, one caregiver (PI:506) said, “*He'll tell me he wants to go to…our local convenience store gas station. That's his favorite place to go, because he can get a Coke or Snickers, and he'll tell me if he wants a Coke or Gatorade*.”

Three caregivers of individuals with Malan syndrome spoke about how their children **ask questions**. Some examples included:

- “*If I’m like, “Oh, we’re going here,” she’ll like “Why?” or, “we’re going to do this,” and she’s like, “Why?”* – PI:507
- *“She’ll also say, “Can I have.” “Can I have ice cream.” Or when we’re ordering, if we order at a restaurant, she’ll say like, “May I please have chicken tenders” to the waitress; to the wait staff.”* - PI:509

Two caregivers of individuals with Malan syndrome provided examples of how their child **told stories** (e.g. something that happened in the past or a story/sequence of events, could be true or untrue). Both examples show the child using verbal speech:

- “*She will tell me a fact. And sometimes, it’s a two step fact. Sometimes she’ll say, “[Name] got traded from the [sports team] to the [other sports team].”* – PI:509
- *“Right after she got out of school, she would try to tell me what would go in school. So, like they had a little party, so she’ll just be like, “Oh, the party,” and then she’ll be like, “was fun,” and then like gibberish. And then like “cupcakes” and “pizza,” and I knew that she was trying to tell me like, “Oh, we had pizza and cupcakes*.” – PI:507

##### Receptive

Ten of the 11 caregivers of children with Malan syndrome mentioned receptive communication concepts during concept elicitation. Examples of receptive communication for this group included following familiar directions (*n=*10), making choices (*n=*5), responding to simple questions (*n=*5), and engaging in simple back-and-forth conversations using words (*n=*1).

Ten participants discussed how their child **followed familiar one- and two-step directions** with actions or words. For example, PI:505 stated, “*He’s actually pretty good at [following instructions]. We will just tell him what to do. Sometimes we have to redirect his attention to us a couple of times before he actually gets it done. But he answers really well to a stern voice when he doesn't have distractions. Just like, “[Name] do this. [Name] go get your shoes and put them away,” or whatever we're asking. And if we're a little bit, not louder, not yelling, but we just have to enunciate a little better for him to really understand. And we have to slow down our speech for him to grasp everything that we're saying*.”

Five caregivers discussed how their child **made choices**. All of the caregivers indicated that their child utilized symbolic communication to make choices (e.g. AAC device, words, gestures or signs), and four reported that their children used multiple modalities to make choices. Example quotes included:

- *“We’ll give him options of what he wants to wear, or what shoes. And then he’ll kind of point at what he wants or say the color of what it is.”* - PI:505
- *“When she gets dressed in the morning, she likes to wear hockey t-shirts, so sometimes she will go to her own closet and pick out the team t-shirt she’d like to wear. Sometimes, she likes to have her parent say two choices out on her. And then, she will either point or pick out the one she wants, or she’ll say the name of the team that’s indicated on the t-shirt*.” –PI:509
- *“She's good at making choices. And she has always been good at making choices. Even before "yeah" came, if you held a yogurt and cottage cheese in front of her, she's picking what she wants… She would 100 percent grab it. And her pointing right now is with her whole hand. I am working on isolating her fingers.”* - PI:510
- *“I will say, “Okay, [name], are you thirsty?” And he’ll say no, he’ll shake his head no. And I said, “Okay, are you hungry?” And he’ll shake his head yes, and then I will give him choices. “Do you want cheese? Do you want grapes?” So, we will go with the things that he really enjoys.”* - PI:508
- *“And I’ll say, “Okay, well what do you want to eat?” And he’ll tell me, you know, something, cereal or yogurt, apple, or he’ll say a restaurant. So, if he says a restaurant well, where do you want to go and then he’ll try to vocalize or verbalize it and if I don’t understand he’ll get his AAC device and I have added those icons, those buttons to his device so he’ll tell me and then I’ll ask him, “So, what do you want to eat from there?” And he’ll go-to his food folder and tell me chicken nuggets, French fries, milk. And I’ll say, “Oh, what else do you want?” And he’ll continue.”*- PI:504

One caregiver (PI:504) described how her child could engage in **simple back-and-forth conversation** around the child’s food choices: “*And he’ll go, “I’m hungry.” And I’ll say, “Okay, well what do you want to eat?” And he’ll tell me, you know, something, cereal or yogurt, apple, or he’ll say a restaurant. So, if he says a restaurant well, where do you want to go and then he’ll try to vocalize or verbalize it and if I don’t understand he’ll get his AAC device and I have added those icons, those buttons to his device so he’ll tell me and then I’ll ask him, “So, what do you want to eat from there?” And he’ll go-to his food folder and tell me chicken nuggets, French fries, milk. And I’ll say, “Oh, what else do you want?” And he’ll continue.”*

##### Pragmatic

Ten caregivers of children with Malan syndrome discussed the social communication their child engages in. The caregivers in this group described a wide range of pragmatic communication behaviors with a specific focus on verbal speech (*n=*6) and gestures (e.g. for please, thank you, and greeting others; *n=*4). Seven caregivers mentioned that their child **used names for people** (*n=*7). One caregiver (PI:507) mentioned that their child could **recognize people’s moods** (e.g. happy/sad/mad) and would **comfort someone** if they were sad.

More advanced social communication examples included requesting to play with a peer using verbal speech (*n=*1), advocating for themselves with a stranger (*n=*1), and changing their communication based on the communication partner (*n=*1). Some examples of these social behaviors included:

- One caregiver (PI:507) described how her daughter might **request to play** with a peer, “*Then she will go in and like maybe, I would say use like the person’s name first, or try to say the person’s name because sometimes she can’t say it clearly. And then she’ll just ask, oh, like, “Play?” Or like if you’re doing something, she’ll ask like, “What is it?” or stuff like that to initiate the playing*.”
- Another caregiver (PI:509) gave an example of her child **advocating for herself,** *“We took her to see a college playoff hockey game. It was a very crowded hockey arena. There were 17,000 people in the hockey arena. And as we were exiting this crowded arena, someone, I think, stepped on the back of her shoe and she turned around in a very appropriately loud and angry voice, said, “Don’t do that.” And we were surprised and I guess, pleased, that she was expressing how she feels. But the fact that she communicated with a stranger at all, I think, only occurred because it sort of met some threshold for her where she was so angry or upset that this person had stepped on her ankle that she was willing to express herself to a stranger. But other than that, she would never initiate a conversation with a stranger.”*
- One caregiver (PI:503) described how her daughter **changed how she was communicating based on the listener**, *“I've heard her on the phone with a friend a few times and it sounds pretty much the same, maybe a little bit more involved, as like she'll do more full sentences than phrases. And maybe that's just because she can get away with it a little more [at home] since we already know half the time what she's trying to say. It's turned into almost a shorthand, I guess.”* Here we see the child is changing their language according to the social situation, and that she understands she needs to be more explicit in her communication so people outside the family understand her.

In contrast to the social communication described by PI:507, PI:509, and PI:503, there were also caregivers that described a noted lack of these pragmatic communication skills. For example, one caregiver (PI:504) described how their child does not understand the social norms of conversation, *“He is starting to initiate conversations with strangers, but there’s socializing issues, so he’ll just get too close to them or he won’t speak loudly enough for them to hear*.”

#### Changes in Communication

The research team interviewed 11 caregivers whose children had Malan syndrome and all 11 discussed how their child’s communication changed overtime. Many caregivers described improvements in their child’s communication (Table S40), including improvements in vocabulary, pronunciation, and receptive language. Five caregivers said they saw improvements once their child began attending school. For example, one caregiver (PI:501) said, “*I would say her changes were slow, and I think it increased with the more time she was spending with her peers in school*.” Another caregiver (PI:502) said, “*I would say once she roughly turned three, she started going to a new school, that she had to receive one-on-one attention. So, that one-on-one attention has really helped her a lot*.”

Table S40. Quotes from caregivers describing improvements in their child’s communication skills over time.

| Participant ID | Quote |
| --- | --- |
| 501 | *She has grown in, like I said, the number of words per sentence…* *for a long time, she only used one-word utterances and two. She kind of stuck at three for a long time, three words, and just really short. And so, it kind of grew.* |
| 502 | *Before, it was just a lot of, “Eh, eh, eh,” or a, “Eh, eh.” [And now] it's actually words. So, just in her vocabulary, it's expanding.* |
| 504 | *So, before it was very difficult for even us to understand what he was saying and that’s one of the reasons why we started using the AAC device… And now, you know, his approximations are a lot closer to being clear to understand where even some words are understood by strangers…* |
| 506 | *And then, now, he's added a few more phrases. I would say there's been a definite jump this past year in his communication, using more words than he used to.* |
| 507 | *Before, she would probably just use one word to communicate. As of now, she’s trying to put like even three-word sentences, so I would say that she’s doing pretty good.* |
| 508 | *It’s really been accelerated this year for him to start saying a lot of words.* |
| 509 | *Definitely, in her receptive language, she has developmentally increased her ability to follow – to understand and follow now, maybe two or three-step instructions versus before, she could only understand one – she could only retain and understand one set of directions. So, it’s changed in that way.* |
| 510 | *Oh my gosh, substantially because she is trying to use words and she will give you the different uhs and ahs and ohs and all of that. And I think because she does know what yes and no mean now, it's easier for her to communicate. Especially if you're asking her [something].* |
| 511 | *So, as she’s gotten older and more physically capable, she’s transitioned to trying to physically problem solve for herself more like she’ll carry a stool to the kitchen to climb up on the counter to get where the candy is hidden. If an adult or someone who can help her is in the room, she’ll kind of take your hand, and point, and kind of be like uh, not really saying things, but you know she’s wanting the treats that are up high. And then, if you say no, she’ll go get a stool or ladder, and climb up there, and try to get them herself.* |

Almost all caregivers (*n=*10) described the speed at which improvement occurs, and the majority (*n=*8) said the improvements occur slowly. Examples of caregiver quotes describing speed can be found in Table S41.

Table S41. Quotes from caregivers describing their perceptions of the speed of skill changes in their child with Malan syndrome.

| Participant ID | Quote |
| --- | --- |
| 501 | *I would say her changes were slow, and I think it increased with the more time she was spending with her peers in school. So, when we talk about communication skills, when she was learning new words, new sight words, new signs, it just takes a long time to kind of stick that into memory.* |
| 502 | *I would say I feel like it's going fast. I feel like every day, that it's getting better quickly. And she's grasping a little—things more. From, I would say, ages one to three, I felt like it was kind of we were making minimal progress anywhere. I feel like now we're taking leaps and bounds. It seems like almost daily.* |
| 503 | *I'd say slow. They were pretty gradual but they didn't take forever but they weren't a couple of days…* |
| 504 | *There was a period where his verbalizing was very slow to the point that we thought he was not gonna progress, but then when we looked at the big picture and we saw all the documentation that was taking place by the SLPs, there were like tiny baby steps that he was taking that led him to actually gain a skill…* *but it just takes forever for him to I guess reach a little goal that will open up a window, a door to so many other things. So, it was always a goal and then we had to just let it go and then we would revisit it, let it go, revisited that goal, and then it wasn’t even something we were working on and he mastered it.* |
| 506 | *So, learning the new words and then putting things – two words together, and all of that, is – in the correct steps of how he's supposed to do it is in just a much, much slower pace.* |
| 507 | *At the beginning of like when she started school, when she started therapy, I would say they were slow to begin with. As of right now, I think that now she has like a little bit of the hang of the stuff going, I think that is going a lot smoother now. I wouldn’t say fast, I would say just a little bit smoother. Like she is repeating everything better, like it’s more understandable.* |
| 508 | *I mean, it’s a good amount of time for him to learn new words. I mean, it’ll be the same words that he’ll use for a very long period of time, but I think it takes him a good like three months to pick up on something.* |
| 509 | *It’s progressed developmentally. More slowly, I think, than a typical person.* *I feel like her communication skills have continued to incrementally improve.* |
| 511 | *Slow…* *I’d say like within three months.* |

Some caregivers (*n=*3) mentioned learning a new skill requires repetition and modeling. For example, one caregiver (PI:501) said, “*It takes quite a bit for her to kind of hold on to a new skill and a lot of repetition is required*.” Another caregiver (PI:503) said, “*It does stick around to a point, as in if we continue to make sure it's being used, the skill. If it's not being used then it tends to be forgotten and it needs to be reminded, I guess... She was in speech therapy and we were practicing things daily, weekly, or like that, it tended to be remembered, but if she went a month without doing it sometimes it was something that we needed to remind her about*.” One caregiver (PI:510) mentioned that certain skills may be learned more quickly depending on her child’s motivation. For example, they said, “*She just in the last three... not even three months, two months, is letting me do hand over hand teaching. If you're trying to teach her how to do something, she picks that up, way faster, than trying to teach her this is what this is*.”

A few other caregivers said that other skills may disappear or plateau when a new skill emerges. For example, one caregiver (PI:501) said, “*When she is typically learning a new skill, her other skills don’t decline, but they kind of stagnate, and that’s across-the-board for her.”* Another caregiver (PI:502), said “*I would say sometimes, the other skills are either forgotten or, you know. I guess that's the best way of saying it. Or they regress a little bit [when learning a new communication skill].”*

Only one caregiver (PI:505) described regressions in their child’s communication. They said, “*I think actually when he first started talking, when he first started saying a couple of words, suddenly it just stopped for a while. And it really didn't come back until he was into early intervention, which was around a year and a half to two years.”* This caregiver felt the regression happened “quickly” (within one month).

#### Hearing & Vision Impacts

The research team interviewed 11 caregivers whose children had Malan Syndrome and all 11 were asked whether their child had any hearing or vision issues that impact their ability to communicate.

Three caregivers reported that their child had a hearing impairment. None of the caregivers who reported hearing impairments felt that the impairment impacted their child’s communication. Two caregivers (PI:505 & PI:509) reported “minor” or “low range” hearing loss. For example, one caregiver (PI:505) said, *“He does have minor hearing loss in each of his ears. But from what audiology had said, it doesn't require any hearing aid because what one ear doesn't have, the other one compensates for. So, they kind of still balance each other out.”* The other caregiver (PI:509) said, *“She has low-grade – low range, excuse me. Low range hearing loss in one of her ears because of a perforation. But that is the – ENT is not overly concerned about it.”*

One caregiver (PI:504) mentioned conflicting information about hearing loss saying, *“For hearing, we had an ABR [auditory brainstem response test] when he was five and a half almost six, and that ABR showed that he has mild hearing loss in one ear and mild moderate in another. But this past November we had another ABR and that one stated that he was within normal range. So, we don’t know if this one is accurate or the previous one was accurate.”*

Seven caregivers of children with Malan Syndrome reported that their child had a vision impairment. Of these, six caregivers (PI: 501, 503, 504, 505, 506, & 508) said that their child wears glasses. Many caregivers mentioned that their child has field of vision issues (e.g., issues with depth perception). Three caregivers felt this was related to their child’s strabismus diagnosis. In general, caregivers felt the field of vision issues mainly impacted their child’s gross motor abilities, not their communication (Table S42). However, one caregiver (PI:509) felt it impacted her child’s ability to text on her phone; please note this child was extremely advanced in comparison to other interviews. This caregiver said, “*But for example, when she’s trying to text on her phone, like, the letters are small, she doesn’t really understand the spelling, and I think if she’s looking at a visual keyboard on her phone, I do, I think that her ability to see where the letters are – it’s such a complex issues…in that it’s hard to tease out whether she doesn’t understand how to spell, she doesn’t recognize the numbers if the numbers are hard for her to see, like, her brain to process where they are, or she just doesn’t cognitively understand. But the short answer is, yes. I do think her visual processing impairment does affect her ability to communicate, especially communicating the words, texting – I mean, with the letters of the texting*.”

Table S42. Selected examples of parent-reported vision impairment(s) and associated quotes from interviews with caregivers of individuals with Malan syndrome.

| Subject ID | Parent-reported impairment(s) | Quote |
| --- | --- | --- |
| 501 | Strabismus, Field of vision issues (depth perception or peripheral vision), Astigmatism, Nearsightedness | *Vision impairments, she has strabismus, so what you might call a “lazy eye,” that affects her depth perception. It has mostly caused issues with her gross motor and depth perception, but as far as communicating, I don’t really think it has [an impact].* |
| 502 | Field of vision issues (depth perception or peripheral vision) | *We’ve noticed that she has depth perception issues. But they can't test her for that until she's able to communicate fully. So, we believe she has depth perception issues, though.* |
| 503 | Field of vision issues (depth perception or peripheral vision) | *She wears glasses… And I know she's got an issue with depth perception.* |
| 504 | Strabismus, Cortical Vision Impairment, Astigmatism | *Like it [strabismus] does affect his perception and that affects like walking and if it’s uneven surfaces he over compensates for that, but the effects to communication, I don’t see it affecting him.* |
| 506 | Field of vision issues (depth perception or peripheral vision), Astigmatism, Nearsightedness | *So, he appears to not be able to see anything below his chin, like that whole field of vision below our chin... And around the house, he's here every day, and it's routine; so, he's memorized the floor and everything. So, he's confident walking around at school or at the therapy places. But if something new is on the floor, he probably wouldn't see it, and would trip. And then, it's almost like he has slow visual processing. And it's like he needs to be narrated everything. So, if I say, "This is a watermelon," he can say, "Watermelon," and then, he'll probably know it in the future when he sees it, as long as it's presented to him at eye level. But if he has to look down at a dinner plate, he usually uses his hands before he's going to use his eyes, to notice what's on his plate, because of that lack of field of vision down below his chin.* |
| 509 | Field of vision issues (depth perception or peripheral vision) | *So, I think they described it as some of her peripheral vision is poor. Sometimes, those specialists have sort of described to me that it’s like if you – that her visual field is as if you’d put a – I don't know, like an eight-and-a-half-by-eleven piece of paper in front of her face that’s up, straight, in front of her face is sort of where her visual field is the strongest. Items and experiences below that visual field are harder for her to get a grasp.* |

#### Meaningful Change

All caregivers during the interviews were asked, “*What would a meaningful change in communication look like for your child?*” If time permitted, most caregivers were asked “*If your child’s communication skills were to decline or get worse, which communication skills would be most important for your child to retain?*”

Parents interpreted “meaningful change” to mean “improvements” in communication ability or skills. In addition, some parents made the distinction between realistic and unrealistic goals they had for their children (Table S43).

When discussing meaningful improvements for their child with Malan syndrome, caregivers mentioned improved interest in other people (*n=*3), communicate if they need help or are in danger (*n=*2), and more vocabulary (verbal speech; *n=*2). A full list can be found in Table S43. All but one child within this sample had verbal words, and so many caregivers’ improvements focused on increasing sentences or increasing verbal vocabulary. For example, PI:501 stated, “I *think a meaningful change in her communication would be flipping communication to the other person. You know, “How was your day?” So, her communication is currently all about herself…And, you know, I think communication too is a two-way. So, maybe listening and responding. She is good communicating her needs and about herself, but maybe if we are talking about maybe something that doesn’t interest her, it’s all self-based. She’s not listening and responding to those kinds of things.*”

Table S43. Responses to “*What would a meaningful change in communication look like for your child*?” from caregivers of children with Malan syndrome.

| Meaningful Improvement | 501 | 502 | 503 | 504 | 505 | 506 | 507 | 508 | 509 | 510 | 511 | Total |
| --- | --- | --- | --- | --- | --- | --- | --- | --- | --- | --- | --- | --- |
| Showing an interest in other people, and asking them questions, listening, and responding appropriately (either within or outside family | 1 |  | 1 |  |  |  |  |  | 1 |  |  | 3 |
| Better communication to reduce frustration/behavior |  | 1 |  |  |  |  |  |  |  |  |  | 1 |
| Start using complex sentences using verbal words |  |  | 1 |  |  |  |  |  |  |  |  | 1 |
| Communicate if need help/ in danger |  |  |  |  | 1 |  | 1 |  |  |  |  | 2 |
| Improved receptive communication |  | 1 |  |  |  |  |  |  |  |  |  | 1 |
| Telling about events with details |  |  |  | 1 |  |  |  |  |  |  |  | 1 |
| Consistent AAC device usage |  |  |  |  | 1 |  |  |  |  |  |  | 1 |
| More words |  |  |  |  |  | 1 |  |  |  |  | 1 | 2 |
| Express feeling and emotions |  |  |  |  |  |  | 1 |  |  |  |  | 1 |
| Back and forth play with another child (around the same age) |  |  |  |  |  |  |  | 1 |  |  |  | 1 |
| Express needs with verbal words |  |  |  |  |  |  |  |  |  | 1 |  | 1 |

In terms of skill loss, caregivers tended to mention specific modalities when discussing what skills they would want their child to retain. These included gestures, verbal speech, AAC device usage, sounds, and eye gaze (Table S44).

Table S44. Responses to “*If your child’s communication skills were to decline or get worse, which communication skills would be most important for your child to retain*?” from caregivers of children with Malan syndrome.

| Worsening communication | 501 | 502 | 503 | 504 | 506 | 511 | Total |
| --- | --- | --- | --- | --- | --- | --- | --- |
| Gesture |  |  |  |  | 1 | 1 | 2 |
| Caregiver unable to this answer/conceptualize this question |  |  | 1 |  |  |  | 1 |
| Verbal words |  | 1 |  |  |  |  | 1 |
| AAC device usage |  |  |  | 1 |  |  | 1 |
| Express needs - any modality | 1 |  |  |  |  |  | 1 |
| Sounds |  |  | 1 |  |  |  | 1 |
| Eye gaze |  |  |  |  | 1 |  | 1 |

*505, 507, 508, 509, 510 were not asked this question due to time constraints

### Phelan McDermid Syndrome (PMS)

#### Demographics

Ten caregivers of individuals with PMS participated. The majority were female (7/10) and on average 42.1 years of age (Table S45). One caregiver self-identified as Hispanic-Latino. Eight caregivers identified as white and two as Asian. Most caregivers reported that they were currently married or living with a domestic partner (6/10), with three who were divorced, and one single/never married. Overall, the participants reported high levels of education, employment, and annual income (Table S45).

Table S45. Demographic information for 10 caregivers of individuals with Phelan McDermid syndrome (PMS).

| *Caregivers* | *n (%)* |
| --- | --- |
| Female | 7(70.0) |
| Age, years (Mean/SD) | 42.1/5.9 |
| Ethnicity |  |
| Not Hispanic or Latino | 9(90.0) |
| Hispanic-Latino | 1(10.0) |
| Race |  |
| White | 8(80.0) |
| African-American or Black | 0 |
| American Indian/Alaska Native | 0 |
| Asian | 2(20.0) |
| Middle Eastern | 0 |
| More than one race | 0 |
| Relationship status |  |
| Single, never married | 1(10.0) |
| Married, or living with domestic partner | 6(60.0) |
| Separated | 0 |
| Divorced | 3(30.0) |
| Widowed | 0 |
| Highest grade in school |  |
| Less than high school diploma | 0 |
| High school degree or equivalent | 0 |
| Some college/University | 2(20.0) |
| College/University degree | 4(40.0) |
| Postgraduate degree | 4(40.0) |
| Occupational status |  |
| Homemaker | 0 |
| Unemployed | 0 |
| Retired | 0 |
| On disability | 0 |
| On leave of absence | 0 |
| Full-time employed | 8(80.0) |
| Part-time employed | 2(20.0) |
| Full-time student only | 0 |
| Income of U.S. residents |  |
| Less than $20,000 | 0 |
| Between $20,001 and $40,000 | 1(10.0) |
| Between $40,001 and $60,000 | 1(10.0) |
| Between $60,001 and $80,000 | 0 |
| Between $80,001 and $100,000 | 0 |
| Between $100,001 and $250,000 | 6(60.0) |
| Between $250,001 and $500,000 | 1(10.0) |
| $500,000+ | 1(10.0) |
| I prefer not to answer | 0 |
| I do not know | 0 |
| Relation to child |  |
| Mother/Step-mother | 7(70.0) |
| Father/Step-father | 3(30.0) |

Caregivers also provided demographic information for their child with PMS. Half of the children were male and half female, with similar rates to caregivers in regards to ethnicity and race (Table S46). Most children were classified as having Class II Deletions (7/10), with two having Class I and one having a Class I Sequence variant (Table S46). Five caregivers reported their children had an ASD diagnosis, and one reported that their child had epilepsy. Many children were receiving therapeutic services (i.e. physical, occupational), with all 10 reportedly receiving speech therapy (Table S46). Six caregivers reported that their child used an AAC device with five reporting that the device was considered ‘high tech’. The settings in which the device was used varied (Table S46). The mean age of the child when the device was introduced was 5.5 years old (*SD* = 3.1).

When asked about their child’s verbal language, two caregivers indicated their child used ‘full sentences’, no caregivers indicated that their child used ‘short phrases’, two indicated their child used a ‘few words’, and six indicated their child used ‘no words’.

Table S46. Demographic information for 10 children with PMS.

| *Children* | *n (%)* |
| --- | --- |
| Age, years (Mean/SD) | 7.5/4.9 |
| Child gender Female | 5(50.0) |
| Ethnicity |  |
| Not Hispanic or Latino | 9(90.0) |
| Hispanic-Latino | 1(10.0) |
| Race |  |
| White | 8(80.0) |
| African-American or Black | 0 |
| American Indian/Alaska Native | 0 |
| Asian | 2(20.0) |
| Middle Eastern | 0 |
| Native Hawaiian/Other Pacific Islander | 0 |
| More than one race | 0 |
| Child’s genotype |  |
| Class I Deletion | 2(20.0) |
| Class I Sequence Variant | 1(10.0) |
| Class II Deletion | 7(70.0) |
| Autism Spectrum Disorder (ASD) | 5(50.0) |
| Epilepsy | 1(10.0) |
| Ring Formation | 3(30.0) |
| Types of therapy |  |
| Physical Therapy | 8(80.0) |
| Occupational Therapy | 8(80.0) |
| Speech Therapy | 10(100.0) |
| Other therapy  ABA Therapy  Equestrian Therapy  Feeding Therapy  Music Therapy  Vital Stim Therapy | 3(30.0)  2(20.0)  1(1.0)  1(1.0)  1(1.0) |
| Age start speech therapy (Mean/SD) | 1.6/1.1 |
| Age first introduced to device (Mean/SD) | 5.5/3.1 |
| AAC device |  |
| No | 4(40.0) |
| Yes | 6(60.0) |
| Device |  |
| High tech | 5(50.0) |
| Low tech | 1(10.0) |
| Places to use the device |  |
| Home | 4(40.0) |
| School | 6(60.0) |
| Out in the community | 2(20.0) |
| Other | - |

#### Typical Communication Ability

The results for this section are organized by behaviors that correspond to expressive, receptive, and pragmatic (i.e. social) communication, aligning with the conceptual framework that was developed for individuals with Angelman syndrome.^1^ Please note, although counts are reported to indicate salience of each theme, these data are limited in that they do not indicate *all* communication behaviors relevant to each child, only the ones that caregivers discussed during the interview.

Most caregivers of individuals with PMS discussed aspects of expressive, receptive, and pragmatic/social communication during concept elicitation (Table S47).

Table S47. Number of caregivers that mentioned one or more examples of expressive, receptive, and/or pragmatic communication.

| Communication Behaviors | *n* |
| --- | --- |
| Expressive | 10 |
| Receptive | 9 |
| Pragmatic (Social) | 6 |

##### Expressive

All caregivers of children with PMS discussed expressive communication concepts. Requesting was a common communication concept mentioned by caregivers (Table S48).

Table S48. Expressive communication concepts on the ORCA measure mentioned by caregivers of individuals with PMS.

| Expressive Communication Function | *n* (total *n=10*) |
| --- | --- |
| Seeking Attention | 4 |
| Directing Attention | 5 |
| Refusing an Object | 4 |
| Requesting an Object | 9 |
| Requesting an Object out of View | 2 |
| Requesting More | 3 |
| Asking Questions | 1 |
| Telling Stories | 1 |

Four of the caregivers of children with PMS discussed how their child typically **sought attention**. One caregiver (PI:402) reported that their child utilized multiple modalities to seek attention, saying, *“She is more tapping to people when she wants their attention. And she can use the card [low tech AAC device], too.”* Two other parents described how their child might use physical gestures (grabbing, hitting, pulling) to seek attention. For example, one caregiver (PI:411) said, “*She will definitely grab me and say, almost like "Mom, I need you" type thing. She gets loud when I don't respond*.” The other caregiver (PI:407) had a 15 month old child that primarily relied on crying and fussing to seek attention.

Five caregivers spoke about how their children **directed attention** to an object, action, or event. One caregiver described their child using a finger point and another described their child using physical body movements. Example quotes included:

- “*He points near, but not far; meaning, if he's reading a book, there's* *something in the book, he may point at particular objects in the book*.” – PI:410
- “…*Or try to take your hand and take you over to what she wants to do*.” – PI:406

Four caregivers spoke about how their child indicated **refusal** (e.g. ‘no’). One caregiver (PI:402) described symbolic forms of communication (e.g., an AAC device and ASL sign) that their child uses to communicate refusal. For example, they said, “*Like, “Do you wanna share the food?” and she picks [the low tech AAC card] “no.”* This child was also in the process of learning to use a head shake to indicate “no”. For example, the caregiver said*, “Not always, but sometimes she shakes her head. It’s not always, so I’m not sure she is using “no” and shaking. I think she does but not always.”* One caregiver (PI:411) of an older child described how their child used word approximations combined with a gesture to indicate refusal. They said, *“She’ll push it away and do that, “Nyah nyah nyah nyah” sound.”* Some caregivers provided other examples of refusal that required more interpretation. For example, one caregiver (PI:408) said, “*She fusses a lot to let us know some need needs to be met. So, we always go through the motion of changing her diaper, giving her something to drink, offering her a snack, changing the channel if there’s a movie on that she might not like until we can make her stop fussing*.”

Nine out of the 10 caregivers of children with PMS discussed how their child communicated **requests** during concept elicitation. The one caregiver that did not mention requesting during concept elicitation had a child under the age of two. All nine caregivers indicated that their child utilized symbolic communication to request (e.g. gestures, signs, words, word approximations, AAC device), although the specific modality differed between children. Some of the caregivers also reported other non-symbolic communication behaviors that they interpreted as requesting (e.g. taking the caregiver over to an object that the child would like). The most commonly requested objects were food and drink, but individuals with PMS in our sample also requested entertainment (i.e. TV), places/activities (i.e. outside), and people. Some examples of requesting included:

- “*He would look at them [snacks]; he doesn't point; he would look at them, and you know he wants something. I think he does that very well*…” – PI:410 (1 year old child - youngest in sample)
- *“When he wants me to turn on the TV to watch Mickey Mouse. He can stand there and point at the remote. And which I know he means he wants me to turn it on” -* PI:404
- *“She touches something if she wants it. If she wants the TV on, she touches the fireplace because the TV sits above the fireplace.” -* PI:408
- *“She knows very little sign language, but she does know a couple things. She does put her hand up to her mouth for food.” -* PI:411
- “*When [the child] was little, she can grab my hand and take me somewhere. If she wants water, she takes me to the water. But now, she’s using the PECS [low-tech device] and the AAC device. So, it’s just more detail, whatever she wants. So, it helps she can do more with the AAC, and I can understand what she wants*…” – PI:402
- “*If she’s already sitting at the table and we have her talker there, she will use it to request a drink. If she wants a particular drink or especially if we’re out and she can’t just walk over – she can’t just go – at home, she’ll normally just go the kitchen. If it were out, she’s more likely to use her talker*…” – PI:406

When describing requesting, six parents described communication behaviors that were not symbolic, but were interpreted by the caregivers as requesting. For example, parents described how their child might use physical gestures to request. For example, one caregiver (PI:401) said, “…*He tends to go to the refrigerator if he wants to get a drink*.” Another caregiver (PI:406) said, “*So, if she’s hungry, I mean, most typically she will just go over to the fridge or to the cupboard*.”

Three caregivers discussed how their child **requested more of something**. Two caregivers (PI:401 & PI:402) said their child used the ASL sign for “more”. For example, one caregiver (PI:402) said, “*If she wants something, she’s gonna grab my hand and make “more” sign and the “help” sign*.” The other caregiver (PI:408) said their child used their AAC device to request more. For example, they said, “*So, she is using switches [high-tech device]* *at school, which is a button that she presses to request – She can request more. And they added another button to request all done*.”

Two caregivers discussed how their child **requested objects that they cannot see** using symbolic communication. Both caregivers (PI:403 & PI:405) said their child used words to request objects out of view. For example, one caregiver (PI:403) said, “*He can talk about what is for breakfast, what’s for lunch meals, food, what he’s doing, what he wants to, where he wants to go, what he wants to wear, things like that*.” The other caregiver (PI:405) said their child could request different people that she wanted to see. For example, they said, “*[the child communicates] Her daily and wants and needs, her interests, her interactions with her sister, or wants to communicate with others like see her grandparents, or something like that*.”

One caregiver of an individual with PMS reported an example of their child **asking questions.** This caregiver (PI:405) said, “*And then, we’ve got some things that she likes to do. She likes to water plants. So, she inquired about, ‘Does this plant need water?’ Stuff like that.”*

The same caregiver (PI:405) also provided an example of their child **telling stories**. For example, they said, “*She also talks about her dolls because now she does a lot of doll playing. So, she talks about what the dolls are doing and things like that.”*

##### Receptive

Nine of the 10 caregivers of children with PMS mentioned receptive communication concepts during concept elicitation. Examples of receptive communication for this group included following familiar directions (*n=*5), making choices (*n=*4), following multi-step directions (*n=*1), engaging in simple back-and-forth conversations using words (*n=*1), understanding and responding to humor (*n=*1), and responding to their caregiver’s voice (*n=*1).

Five caregivers described how their child could follow **one- or two-step familiar directions**. Only one caregiver described how their child could follow multi-step new/novel directions with words or actions. Several caregivers mentioned that their children only followed directions on their terms or when they felt like it. For example, PI:411 stated, “*My daughter is on her-terms-and-her-terms-only, mostly. I must say, she will follow directions when she wants to. But most of the time, not. When she's downstairs and I'm upstairs, and I tell her to come up to eat, or come to the bathroom or whatever, we have to go get her. Okay? But sometimes, pick up the cup; do this, or do that; she'll do it right away. So, it's an on-and-off situation.”* Based on the nature of how children with PMS follow directions (e.g., on their own terms) parents often paired one and two step directions when reporting on this skill.

Four out of the 10 discussed how their child **made choices** during concept elicitation. All of the caregivers indicated that their child utilized symbolic communication to make choices (e.g. AAC device, words, gestures or signs). Two caregivers (PI:402 & PI:408) described how their children made choices using a low tech AAC device. One caregiver (PI:402) said, “*If I don’t know what she wants at home, I show her the PECS card. And then she picks the card. At school, she started to use the AAC device on the iPad, the app. She just is using [that] okay, so far.”* Two caregivers (PI:402 & PI:411) described how their children used a gesture (e.g., pointed or grabbed items) to make a choice, with one (PI:411) caregiver saying, *“She'll point to it or grab it. I always give her choices, with even snacks. Obviously not dinner, but snacks, books. I make her get up, and on my TV, you can point to the shows that she wants. So, I make her point to what she wants. She will let you know.”* The other caregiver (PI:402) said, *“Usually, I show it to her in front of her. And then, she’s just gonna pick, grab the one she wants… She can point but not in the air. She can actually point to the object, but she cannot point in the air. And then, sometimes she’s just gonna grab instead of pointing.”* Finally, one caregiver (PI:405) reported that their child was able to verbally name their choice using words. For example, they said, *“I ask her what she wants, and I give her some choices. And then, she [says] this that or the other.”*

There were only two children in this group with the ability to speak in full sentences using verbal words. The caregivers of these two children described how their children used more complex communication skills. PI:403 described how his child was able to **engage in simple conversations**. PI:405 described how her daughter was able to **understand jokes and humor and laugh appropriately**.

Two caregivers (PI:407 & PI:408) of children under 4 years old used the phrase “*in their own world*” when discussing how their children did not typically respond to communication, such as turning towards the sound of their name.

##### Pragmatic

Six caregivers of individuals with PMS described the social communication their child engages in during concept elicitation. Examples of pragmatic communication included the use of eye contact and social smiling to engage with others (*n=*2), greeting others (e.g. waving or saying ‘*good morning’*; *n=*2), pretend play (*n=*1), turn-taking game (*n=*1), and laughing appropriately (*n=*1). No caregivers of individuals with PMS mentioned their children using specific names to reference people during the concept elicitation portion of the interview.

One caregiver (PI:411) described a turn taking game and said, “*When Grandpa is sitting next to her, he's in the chair, she's on the futon, and her foot's hanging over the futon, and he hits her foot. Well, he didn't hit her foot one day, and she hit his foot. So, it was almost like she remembered, "This is what he did. Why isn't he doing it now? I'm going to do it back, and then she starts giggling when she hits his foot.*”

PI:403 also described a lack of pragmatic communication skills, when asked whether his child used a different communication tactic when being misunderstood, the caregiver responded, “*He’ll only repeat himself if I ask him to. He doesn’t realize that he’s not being understood unless I tell him*.”

#### Changes in Communication

The research team interviewed 10 caregivers whose children had PMS and all 10 discussed how their child’s communication changed overtime. Many caregivers described improvements in their child’s communication (Table S49), including improvements in AAC device usage, receptive language and sound production. The caregivers that described improvements in sound production had young children between ages 1-3. Several caregivers said speech therapy contributed to these improvements. For example, one caregiver (PI:405) said, “*And there was a ton of speech therapy that she received there that was very specific and targeted for these oral motor speech production mechanisms. And that’s what really made the difference. And she acquired a lot of syllables, a lot of words, and started to speak in some simple sentences*.”

Some caregivers (*n=*4) described the speed at which improvement occurs, and all four said the improvements occur slowly. For example, one caregiver (PI:402) said, “*Slowly. At the ABA therapy, we keep telling her you need to tap for the attention. And we would be telling her for maybe a year, and she’s using the tapping more now than grabbing*.” Another caregiver (PI:403) said, “*It took him a long [time to use words] because with his condition, the muscles in his mouth, and his jaw, and his tongue don’t actually form the right shapes to make the sounds. So, it’s not like other kids that have other issues where they physically could talk if everything were wired correctly, for lack of a better term. But he can’t actually make the sounds*.”

Table S49. Quotes from caregivers that described improvements in communication over time.

| Participant ID | Quote |
| --- | --- |
| 401 | *I took him to an outside source, to an AC specialist this last year to get him evaluated [and] get an AAC device that is more appropriate for him. So, he’s been using that more so that has expanded his vocabulary, for sure.* |
| 402 | *When [she] was little, she can grab my hand and take me somewhere. If she wants water, she takes me to the water. But now, she’s using the PECS and the AAC device.* |
| 404 | *He’s gotten better with his AAC, but he still doesn’t rely on it as a primary [mode of communication].* |
| 405 | *But, of course, her understanding [has] evolved dramatically.* |
| 407 | *He’s only 15 months old. But we’ve definitely noticed more squeals and sounds. When he was younger he just cried, but now he’s got some sounds that he’s thrown in there.* |
| 408 | *Just over the weekend, we’ve noticed she’s making a lot of mm sounds like yum and more and mom.* |
| 410 | *He's more capable of actually making sounds now, compared to when he was at 20 month, for example.* |
| 411 | *So, I guess the first thing is that her eye contact has definitely changed, with the person that she's trying to communicate with. Before, it looked like she was just talking in the air, looking around; and now, she will actually look at you and talk to you.*  *She's definitely more vocal now than she ever was, with sounds. That has changed.* |

Parents also discussed that changes in communication are variable and inconsistent, with some skills coming and going over time (Table S50). Some caregivers mentioned this only in regards to verbal speech (e.g., that words disappear and may reappear over time), but other caregivers said they’ve seen other communication modalities disappear over time if their child learns to communicate in a different way.

Table S50. Quotes from caregivers when speaking about communication changes over time.

| Participant ID | Quote |
| --- | --- |
| 401 | *He has gained words, lost words. When I’m talking about “words,” he probably has maybe five or 10 words that he says, or approximate, and those 10 words, some have completely gone. Some have left and come back. So, as far as actual speech, it has varied in that way… It feels like he has a filing cabinet. He can only hold so many words. And so, in order to gain a new word, he has to lose another word; although I feel like his filing cabinet has gotten a little bit larger since he was little.*  *He doesn’t use as many signs as he used to. He did more signing like of “all done,” “more,” “eat,” “drink.” He uses less of that because he tends to go to the refrigerator if he wants to get a drink…*  *So, I feel like with the NOVA Chat, I feel like he is leaving behind some of those other skills... I have other kids that are neurotypical. They learned sign, and once their speech started improving, they stopped using their sign language. So, I feel like it’s the same for him except for it’s just been a much longer period.* |
| 402 | *For example, she learned the “help” sign. And then, after that, she was using “help” sign only when she could [also] use more.* |
| 404 | *He will have words and he’s lost words before, which is common with this disorder. He could have a word for three months, and then overnight, it’s gone and it’ll never come back.*  *When he was learning sign language five years ago, he would learn some signs and lose them.* |
| 406 | *Or sometimes she would gain one [word] and stop using another one. When she started saying “I want,” she stopped saying, “I need.”* |
| 410 | *I've spoken to other parents. It's like you gain a skill and you may lose another skill. But I think in [child’s name] case, I wouldn’t say this is very obvious that he lost it... He's just not interested in doing clapping anymore, because he can make more sounds now.* |
| 411 | *We kind of feel like she gets bored with the old way and doesn't want to do it the old way anymore, and wants to do it the new way, the way she's being taught.* |

In this sample, four caregivers of individuals with PMS also spoke about loss of skills, some using the term ‘regression’ (Table S51). Notably, all three caregivers of children with Class I deletion mentioned regressions in their child’s communication. Two out of the four caregivers said their child had experienced regressions in their mobility as well.

Table S51. Quotes from caregivers describing loss in their child’s skills over time.

| Participant ID | Quote |
| --- | --- |
| 406 | *So, she’s had a big regression this year. When she was 1 or 2 years old she probably had 10 to 12 words and then up through pre-K and kindergarten she had phrases, like she would say, “Where’s the doggy?” Or “What is it?” “What’s that?” Or “I see it.” “I do it.” “I want.” Kinda like pretty much sentences that were appropriate that were used in context. “I need,” “I want.” She used to – and this is all up until kindergarten. She used to be able to say “A, B, C.” She used to be able to sing “Happy Birthday.” Right now, as of today, I’ve heard her say maybe two words in the last month. And those are “no” and her brother’s name which is [name].*  *I mean, she’s had a big regression in every skill this year. Up until this fall she could put on her own pants and socks and shoes. She does not do that anymore. She would make choices between what shirt she wanted to wear. She doesn’t really do that anymore. She used to feed herself really properly with her silverware and to color and draw. She doesn’t do that anymore.* |
| 407 | *So, he’s already – he has lost some skills. He used to clap, and he doesn’t clap anymore. And he used to attempt to self-feed and would self-feed sometimes, and he doesn’t anymore. So, we have noticed that he’s lost some skills that he previously had.* |
| 408 | *Well, she did used to talk, and she regressed. So, she did have a couple words up until between about 18 months and 24 months, she kind of lost everything. But I do feel like she’s starting to gain some things back.* |
| 410 | *When he was 12 month, he used to say like, "Mama, papa," just mimicking, but that was like 11, 12 month old. And then, once he got to 15, 16 month, he stopped doing that. So, right now, he doesn't mimic words at all* |

Finally, like in other groups, two PMS caregivers mentioned learning a new skill requires repetition and modeling. For example, one caregiver (PI:404) said, “*He can retain the other skills as long as they are practiced at least occasionally. Otherwise, it’s just like learning another language. If you don’t use it, you’ll lose it.*” Another caregiver (PI:408) said, “*She has to practice her skills, honestly, or she will lose them.”*

#### Hearing & Vision Impacts

The research team interviewed 10 caregivers whose children had PMS and all 10 were asked whether their child had any hearing or vision issues that impact their ability to communicate. Two caregivers (PI:401 & PI:407) reported that their child had a hearing impairment. Neither caregiver felt the child’s hearing loss impacted their communication ability.

One caregiver (PI:401) reported “mild hearing loss”, saying, *“His hearing. He can be really loud and I [think] that he has a mild hearing loss. And sometimes he’s really loud, so I’m wondering if that's because of his hearing loss. But he still listens to directions and follows directions and can communicate that, whether with compliance or non-compliance.”* This caregiver said the child is supposed to wear hearing aids, but does not. Another caregiver (PI:407) said their child has moderate hearing loss and said, *“So, he does have a cleft palate. He does have fluid in his ears. And he had a hearing test done with audiology a couple of months ago, and it did say moderate hearing loss. Just because of the fluid in his ears.”*

Five caregivers (PI: 401, 404, 405, 408, & 411) reported that their child had a vision impairment. Impairments included nearsightedness, nystagmus, functional vision deficits with intermittent exotropia [a type of strabismus], astigmatism, and farsightedness. None of the caregivers reported that their child’s vision impairment impacted their communication. However, one caregiver (PI:405) said their child’s functional vision deficits impact their ability to read. For example, they said, “*You have to write things in large font and leave spaces between the letters horizontally, much more so than otherwise, because her eyes get really tired trying to focus at this reading type distance when the font is small and then when there’s very little distance horizontally between symbols…* *So, I guess the point is that functional motor, while it doesn’t necessarily affect her speech, right, it certainly affects her ability to show what she can do in terms of reading and things like that. And if I didn’t know she had this, I’d just think that, “Oh, well, she doesn’t know these sounds.” She knows these sounds. She just can’t see them.”*

#### Meaningful Change

All caregivers during the interviews were asked, “*What would a meaningful change in communication look like for your child?*” If time permitted, most caregivers were asked “*If your child’s communication skills were to decline or get worse, which communication skills would be most important for your child to retain?*”

Parents interpreted “meaningful change” to mean “improvements” in communication ability or skills. In addition, some parents made the distinction between realistic and unrealistic goals they had for their children.

When discussing meaningful improvements for their child with PMS, caregivers mentioned improved communication to reduce frustration (*n=*4), communicating pain or illness (*n=*3), and more vocabulary (AAC device or verbal speech; *n=*2). A full list can be found in Table S52.

Table S52. Responses to “*What would a meaningful change in communication look like for your child*?” from 10 caregivers of children with PMS.

| Meaningful Improvement | 401 | 402 | 403 | 404 | 405 | 406 | 407 | 408 | 410 | 411 | Total |
| --- | --- | --- | --- | --- | --- | --- | --- | --- | --- | --- | --- |
| More vocabulary on high tech AAC | 1 |  |  |  |  |  |  |  |  |  | 1 |
| More words using verbal speech | 1 |  | 1 |  |  |  |  |  |  |  | 2 |
| Communicate if need help/ in danger | 1 |  |  |  |  |  |  |  |  |  | 1 |
| Communicate with people outside of immediate family | 1 |  |  |  |  |  |  |  |  |  | 1 |
| Express feelings/emotions or using any modality (e.g., AAC, words) |  | 1 |  |  |  | 1 |  |  |  |  | 2 |
| Communicate pain or illness using any modality |  |  |  |  |  | 1 | 1 | 1 |  |  | 3 |
| Improved communication (e.g., pronunciation, words) to reduce frustration (when other people do not understand) |  |  | 1 | 1 | 1 |  |  |  |  | 1 | 4 |
| Start using verbal words |  |  |  | 1 |  |  |  |  | 1 |  | 2 |
| Consistent communication using AAC |  |  |  | 1 |  |  |  |  |  |  | 1 |
| Communicate toileting needs |  |  |  |  |  | 1 |  |  |  |  | 1 |
| Back and forth conversation with another person |  |  |  |  |  | 1 |  |  |  |  | 1 |
| Express preferences |  |  |  |  |  |  | 1 |  |  | 1 | 2 |

Responses to the question about meaningful skill retention from the perspective of caregivers can be found in Table S53. While the responses included specific modalities (e.g. eye gaze, device usage, gestures), parents in this group also mentioned general concepts like expressing needs and skills for independent living. When responding to this question, many caregivers also stated their child did not have skills to lose. For example, one caregiver (PI:408) stated, “*But right now, the communication is just so little that I don’t – There’s not much to keep. They’re working on stuff at school. But like I said, she’s not quite there. So, yeah. There’s not much for her to lose.”*

Table S53. Responses to “*If your child’s communication skills were to decline or get worse, which communication skills would be most important for your child to retain*?” from 10 caregivers of children with PMS.

| Worsening communication | 401 | 403 | 404 | 405 | 406 | 408 | 410 | Total |
| --- | --- | --- | --- | --- | --- | --- | --- | --- |
| Caregiver unable to this answer/conceptualize this question |  | 1 | 1 | 1 |  |  |  | 3 |
| Communicate hunger and thirst |  |  |  |  | 1 |  |  | 1 |
| AAC device usage | 1 |  |  |  |  |  |  | 1 |
| Mobility | 1 |  |  |  |  |  |  | 1 |
| Eye gaze |  |  |  |  |  | 1 |  | 1 |
| Skills for independent living |  |  |  |  |  |  | 1 | 1 |

*402, 407, 411 were not asked this question due to time constraints.

### Schinzel-Giedion syndrome (SGS)

#### Recruitment Challenges

As described in the methods section, SGS is an ultra-rare disorder with extremely severe impacts on the child. Families and caregivers often manage a number of complex and severe symptoms related to the disorder, and children diagnosed with the classical form of SGS have a life expectancy of around 18-48 months. To maximize recruitment, we left this cohort’s enrollment period open for a prolonged period (>4 months) and opened up recruitment to English-speaking participants in other countries. Despite the strong involvement of our patient advocates, we had difficulty recruiting caregivers of children with SGS during the project period.

During the enrollment period, we had to remove two caregivers from our ‘potential recruitment list’ provided by the foundation due to their child passing away. We screened seven total participants; of those, two were screened but not enrolled (re: eligibility) and one participant was enrolled but withdrawn prior to the interview after we lost contact. We were successfully able to conduct four caregiver interviews.

#### Demographics

Four caregivers of children with SGS participated. Three caregivers were female and one was male. On average, they were 41.8 years old (SD=8.4). One caregiver self-identified as Hispanic-Latino. Three caregivers identified as white and one as Native Hawaiian/Other Pacific Islander. All four caregivers reported that they were currently married or living with a domestic partner. Levels of education, employment, and income can be found in Table S54.

Table S54. Demographic information for four caregivers of children with SGS.

| *Caregivers* | *n (%)* |
| --- | --- |
| Female | 3(75.0) |
| Age, years (Mean/SD) | 41.8/8.4 |
| Ethnicity |  |
| Not Hispanic or Latino | 3(75.0) |
| Hispanic-Latino | 1(25.0) |
| Race |  |
| White | 3(75.0) |
| African-American or Black | 0 |
| American Indian/Alaska Native | 0 |
| Asian | 0 |
| Middle Eastern | 0 |
| Native Hawaiian/Other Pacific Islander | 1(25.0) |
| More than one race | 0 |
| Relationship status |  |
| Single, never married | 0 |
| Married, or living with domestic partner | 4(100.0) |
| Separated | 0 |
| Divorced | 0 |
| Widowed | 0 |
| Highest grade in school |  |
| Less than high school diploma | 0 |
| High school degree or equivalent | 1(25.0) |
| Some college/University | 0 |
| College/University degree | 1(25.0) |
| Postgraduate degree | 2(50.0) |
| Occupational status |  |
| Homemaker | 0 |
| Unemployed | 1(25.0) |
| Retired | 0 |
| On disability | 0 |
| On leave of absence | 0 |
| Full-time employed | 2(50.0) |
| Part-time employed | 1(25.0) |
| Full-time student only | 0 |
| Income of U.S. residents |  |
| Less than $20,000 | 1(25.0) |
| Between $20,001 and $40,000 | 0 |
| Between $40,001 and $60,000 | 0 |
| Between $60,001 and $80,000 | 1(25.0) |
| Between $80,001 and $100,000 | 0 |
| Between $100,001 and $250,000 | 2(50.0) |
| Between $250,001 and $500,000 | 0 |
| $500,000+ | 0 |
| I prefer not to answer | 0 |
| I do not know | 0 |
| Relation to child |  |
| Mother/Step-mother | 3(75.0) |
| Father/Step-father | 1(25.0) |

Caregivers also provided demographic information for their child with SGS. One child was female and three were male, with the same identified ethnicity and race as caregivers. The ages of the children were 5, 7, 10, and 11 years. Three children had atypical SGS and one child had classical SGS. When asked about their child’s verbal language, all four caregivers indicated that their child used ‘no words’.

One caregiver reported their child had an ASD diagnosis, and two reported that their child had epilepsy (one reported upwards of 100 seizures a month, the other reported 150-200 seizures per month). Three of the children were receiving physical therapy, three were receiving occupational therapy, and two were receiving speech therapy. None of the children with SGS used an AAC device.

#### Typical Communication Ability

At the beginning of the concept elicitation portion of the interview, caregivers of individuals with SGS (*n=*4) were asked to discuss how their child typically communicates, including what their child communicates about, who their child communicates with on a typical day, and what modalities (e.g., gestures/signs, sounds, words, device etc.) they use to communicate.

Children in this sample were severely impacted; in general, caregivers tended to describe their child’s communication as “*limited*”. According to parents, none of the four children used words, American Sign Language (ASL), or an AAC device. Caregivers described symbolic gestures, as well as physical gestures and sounds their child used that required interpretation by the caregiver. Caregivers also discussed the fact that they have to interpret all or almost all of the behaviors their child exhibits, and that it can take a lot of time to understand what their child means or what they need.

##### Expressive

Every SGS caregiver in our sample discussed how their child **refused activities** during concept elicitation. Most caregivers described how their child used physical gestures to refuse, such as looking away or turning their head away. One caregiver said their child refused more often when they are tired and two caregivers said they tend to see refusal behaviors during medical procedures (e.g., blood draws, eye/ear cleaning, etc.). Some example quotes included:

- “*He will turn his head like, “I’m not going to eat,” and he will close his mouth. He also is bottle-fed. And during that, when he’s having a really hard time, we have to syringe it, because he will literally not close his mouth around a bottle*.” – PI:901
- “*He will basically communicate, “I’m not going to play with you,” and he will turn his little body away – he will scoot away from us if we try to play with him when he’s really tired*.” – PI:901
- “*Yeah, or if we get the eye boogers out of his eyes. He doesn’t like that. So, he’ll try to move his head away. And same if you’re cleaning his ears. He doesn’t really like it. So, he’ll try to squirm to get away*.” – PI:903
- “*He can say, if he doesn’t want to eat, he won’t just open his mouth at all, or he will just do this with his mouth closed. So, that’s one of kinda like a big thing, when he’s expressing something that he doesn’t want, especially when eating or drinking*.” – PI:904
- “…*if bloods are taken from one of her wrists or anything, then she can be extraordinarily non-compliant and pull and tug and you need a lot of people to hold her. And that’s obviously a form of communication ‘cause she’s telling us that she doesn’t like that*.” – PI:905

Caregivers also described interpreting certain behaviors to mean that their child was **uncomfortable or upset.** However, since these behaviors were non-specific, caregivers were often uncertain whether these behaviors were related to gastrointestinal issues, pain, fatigue/tiredness, or something else. Some example quotes included:

- “*So, when he’s up for a very long time, he’s very – it’s like he wants you to help with the problem, but he doesn’t know how to say it. So, he will grab hair. He will pull and pinch at your face like he’s clawing at you. He’s trying to express that something is wrong and hurting. But we’ve been to so many doctors, and been admitted into the hospitals. And pretty much, we have not been able to determine what it is that’s giving him a hard time… He is very squirmy, and not relaxed. So, we know that he’s struggling. And so, he’s very hard to hold, and hard to comfort, ‘cause basically, nothing works*.” – PI:901
- “*More of the communication-based stuff with [child’s name] is more due to discomfort or like gastro-related things, like when his stomach is upset, or those kinds of things. He’s more apt to have a sense of communication when he’s uncomfortable… So, like groaning. Increased agitation and fidgeting type of stuff like that*.” – PI:903
- “*We know when he cries, something is really hurting, or he’s not really comfortable because he has a high pain tolerance, we noticed. So, when he’s not feeling really good, we know when he cries, it’s really something uncomfortable*… *Because especially when he’s not feeling well, because when we hold him, he’s kinda screaming and tensing up, and then as soon as we put him in the rocking chair, he will just loosen up. His muscles will be relaxed. And if he’s crying, when we put him down there, he will stop crying*.” - PI:904
- “*In terms of body language, so, for instance, in the water, it’s quite clear when she’s relaxed and when she’s not. So, and that’s just the way she separates her legs. Either they’re crossed, which means she’s tense. So, that’s a form of communication I would say. And then, when she uncrosses her legs and just lets them float and they’re separate, then that means she’s clearly telling me or whoever’s in the pool with her that she is relaxed..*.” – PI:905

Two caregivers observed behaviors (e.g. smiles, sounds) that they interpreted to mean their child is **happy or comfortable.** For example, one caregiver (PI:901) said, “*He has sounds, but I haven’t been able to link any of the sounds to really, anything other than – he has certain sounds when I feel that he is happy. And usually, they’re “Lalala,” “Ledeledele,” anything that kind of starts with L or a B.”* Another caregiver (PI:904) said their child is happy when their family sings songs to him. For example, they said, “*And yeah, I guess when he’s wearing his hearing aid, when my daughter comes to him and singing to him, and also when we eat dinner because we eat dinner together. We include him on the table. So, he seems very excited on that. He will have a good, big smile on his face*.”

Three caregivers spoke about how their child would make simple **requests** (i.e. to be picked up) or how they know their child needs something (i.e. hunger, diaper change). Example quotes included:

- “*But I could probably count a few times within the last month or two that he has reached his arms both up in order to get picked up. And on cue*.” – PI:901
- “*So, yeah, and sometimes too, with the sounds, when in the middle of the night, if he has a soiled diaper, he will definitely make a big sound... It’s just a big sound, like ah, something like that or – I don’t even remember how he makes those sounds, but it’s really loud sound to let us know, come check on me, something like that. So, most of the time, he’s wet, or he had a soiled diaper*.”

“*Oh, when he’s hungry, he will do the – it was really loud. So, in the middle of the night, when he wakes up, sometimes I’m kinda like, maybe it’s just – I’ll wait for at least maybe 15 minutes, if he’s awake, and he’s been doing that. I’m like, okay, he’s really hungry, and he needs a drink*.” – PI:904

- “*So, if you put her into a bath and it’s a bit too hot, she will tell you. She’ll actually absolutely tell you that it’s too hot. And the way she tells that is by making noises…They’re more shock noises, which is, “Hoo, hoo.” [When it is cold]… Or, “Ah, ah,” like that, you know, where you just feel she – you know, it’s just too hot*.” – PI:905

##### Receptive

Every caregiver of a child with SGS mentioned at least one receptive communication concept during concept elicitation including responding to familiar directions/routines and to familiar voices. Some examples included:

- “*When I get him out of his bed in the morning, our first thing to do is to just change his diaper right before I pick him up. And he has laid down on cue for me. I’ll say, “Lay down. Let’s get your diaper changed,” and he will lay down*… *But I’m not sure if it’s communication or just he knows that for the last 10+ years that we change his diaper in the morning in his bed, and that’s right before he gets up... It may be because he sees the diaper, or maybe because I say, “Lay down.” I’m not certain*.” – PI:901
- “*If I say “We’re gonna go take a bath,” then sometimes he’ll clap his hands*.” - PI:901
- “*You can definitely tell when he hears someone that he knows well, like me, his mom, or his dad. I think the grandparents too, he really recognizes them, and his face almost brightens up some when he’s awake. Sometimes, he’ll smile*.” - PI:903
- “*Yeah, especially with tickling kids. I guess, ever since he was a baby, I always do that after I change his diaper. So, whenever I do this, he was anticipating it. You can see on his facial expression, he’ll be trying to close his eyes like it’s coming kinda thing. So, I kinda always do that every time I change him... And I know he’s expecting it. He’s anticipating it*.” – PI:904

One caregiver gave examples of how they communicate and interact with their child as they move through their child’s daily routine. For example, this caregiver (PI:904) modeled giving their child choices and said, *“Normally, so let’s say for a shirt, we have a blue here, and a red shirt here... If he looked at, let’s say red, just like, okay, you’d like red to wear today, something like that. Or sometimes, with his pureed food, so like, do you like the avocado, or do you like the mango? So, that’s kinda, if he looks at one thing, so like, okay, you like this one, something like that. So, sometimes we don’t get a reaction or something like that, or sometimes we’re like, oh, if he blinks, okay. I think he liked this one*.”

Some caregivers mentioned that it was hard to determine if their child’s behavior was intentionally communicative because responses could be subtle. For example, one caregiver (PI:905) said “*I think [she] definitely [turns to] familiar voices. I think that’s fair to say... I think she does have a tendency sometimes to recognize people. But again, is it hope or coincidence or actually the case? It’s difficult to say*.”

##### Pragmatic

Three caregivers of individuals with SGS mentioned pragmatic communication concepts during concept elicitation (Table S55). Caregivers shared how their children respond when they see familiar people or hear familiar voices. The responses that caregivers noticed in their children included social smiling, cuddling, clapping, touching, and extending their feet towards a loved one.

Table S55. Examples of pragmatic communication from caregivers of children with SGS.

| Participant ID | Quote(s) |
| --- | --- |
| 901 | “*So normally, he loves being held. He likes to kind of snuggle in. And with me especially. If he’s with me or his dad, we – we call it him giving us hugs, but he presses his forehead against our forehead… it’s his way we feel of being social, and communicating to us that. And he’s always smiling when he does it, and he just bumps heads, and then just pushes. Just the slightest pressure. And so, we push back just a little bit. And we always say, “I love you too.” And it just seems to be our way of hugging*.”  “*He does tend to clap when his dad walks in the door from work.”*  “*He has three other siblings. And he will definitely touch their leg, or squeeze their hand, and smile at them, and kind of be social… I do sense that he kind of misses them [siblings] in a way. And so, when I talk to them on Facebook, he’ll kind of react and make some “Lele,” “Lala” noises whenever that he sees their faces on the phone*.” |
| 904 | “*Yeah, he seems very excited, but especially when my daughter comes to him. And yeah, I guess when he’s wearing his hearing aid, when my daughter comes to him and singing to him, and also when we eat dinner because we eat dinner together. We include him on the table. So, he seems very excited on that. He will have a good, big smile on his face*.” |
| 905 | “*She has done this with her grandmother, one of her grandmothers, where she’ll kind of smile at them. And again, the smiling has some seizural connotations where it could be pre-seizural, but if she smiles and doesn’t seizure after, then you could assume that she has smiled at someone as opposed to smile, and then that leads into a seizure. So, she does smile*.”  “*And then, the most subtle form of communication that she has are rather than extending her hands to you or indeed talking to you or anything like this, she will tend to extend one of her feet, typically her right foot towards something or someone and is very happy when you grab hold of it… So, the nurse that we had today, as an example, because again, this is very fresh as in it happens on a daily basis, said goodbye, and then instead of putting her hands out, she just put both her feet up and started almost waving with her feet. And I think that it’s a little bit to close to the saying goodbye for it to be random. That’s not a coincidence ‘cause it’s happened too many times*.” |

#### Changes in Communication

The research team interviewed four caregivers whose children had SGS and all 4 discussed how their child’s communication changed overtime.

The caregiver of a child with classical SGS (PI:903; 7 years old) said they do not believe their child’s communication has changed. Instead, they, as caregivers, feel they have “*adapted to understanding what his needs are*.”

The two caregivers of older children with atypical SGS (10 and 11 years old) described declines in their child’s communication abilities.

- “*If anything, I would say it’s a little less as he has gotten older, versus – he kind of peaked maybe around 1.5 years old. He could be in a little gate trainer, and he would take a few steps towards us, and we’d say, “Come here!” And we would think that he would kind of respond and push himself. But as he’s gotten older, not as responsive to really, any commands, or a want to really reach or do or change positions on the floor. He used to kind of want to try to roll, or if we’d place him on his tummy, he would kind of try to scoot around. But no, not anymore. So, I think communication, along with mobility, has gotten worse as he got older… I would say it’s been a pretty consistent downward slide for [child’s name]. Everything from he used to play with his toes, or he used to hold a ball and put it in both hands. He does it sometimes, but not as much as what he did. So yeah, it’s been pretty consistently just losing skills*.” – PI:901
- “*Her favorite activity is to be in a warm pool…And in the past, she had been extraordinarily communicative... That, you know, me changing her, putting her swimming suit on and so on before the swim where she just went completely ecstatic with that... And when I say ecstatic, it’s a lot of cooing and noises and arms and legs up, etc. Just knowing that, you know, that she’s going into the pool. She doesn’t do that anymore. So, from that peak, which was very brief, she does not do that anymore*.” – PI:905

The same caregiver (PI:905) also mentioned some improvements with their child’s communication, saying, “*So, one form of communication I’d say, would be with her eyes. And seeing if she looks at you and say, “Yes, I’m listening to you,” and so on. That for the first certainly year and a half of her life, again, understanding that she’s 11. That was catastrophically bad. So, she spent certainly the first six months of her life with her eyes closed or barely open. And would always, always look away. So, if you’re trying to look at her in the eye, she would look away, look away, look away, whichever place you would place your head, she would just look away and look away. And we have had a substantial change in medication that we give her which seems to make her more alert*.” The child also used to “*scream the house down as a small child*”, but this stopped once the seizure medication was implemented.

The fourth caregiver (PI:904) had the youngest child (age 5; atypical SGS) in the sample. They described only improvements in their child’s communication abilities. For example, they said, “*He barely laughed when he was a baby. And also, with him closing his mouth when he doesn’t wanna eat or drink anymore, I think that’s a really big change since he was a baby. And also moving his head when he doesn’t want to eat or drink anymore*… *And he will definitely kinda – responds to your play. When I do the peekaboo, he likes peekaboo. So, he’ll laugh when I do peekaboo. When he was baby or for a year or two, no reaction, but for the past couple of years, I would get some laughter during peekaboo time*.”

#### Hearing & Vision Impacts

Two caregivers of children with SGS reported that their child had a hearing impairment. One caregiver (PI:903; classical SGS) specifically used the term “*moderate to severe hearing loss*” and said their child occasionally wears hearing aids; however, the aids can be “*overstimulating*” and lead to increased seizure activity.

Another caregiver (PI:904; typical SGS) also said their child wears hearing aids sometimes (because of unspecified hearing loss) but it had been challenging recently to try and get the frequency correct. For example, the caregiver said, “*We just had a recent audiology appointment, and they have to lower [them] down because there were so many times that whenever we put the hearing aid on, he would cry. So, they lowered it down, and it seems that he’s tolerating it*.”

Lastly, one other caregiver (PI:905) said their child had no formal hearing impairment, but believed their child hears high pitched sounds better than low pitched sounds. For example, they said, “*And recent findings seem to suggest that she can hear very high-pitched sounds. So, she reacts and will turn her eyes towards the sound. But absolutely does not hear low-pitched sounds. So, she probably doesn’t hear my voice ‘cause my voice is so low. But she will here someone with a much higher tone of voice, such as some of the teachers or indeed maybe the nurse today...”*

Only one SGS caregiver had a child with a vision impairment (cortical visual impairment; CVI). When asked about the impact of this on their child’s communication, the caregiver said, “*It’s really hard with him, because he has cortical visual impairment as well. So, he doesn’t necessarily look at you all the time, but he does sometimes*.”

One other caregiver (PI:905) said their child had no formal vision impairment but said they are not sure what the child can see. For example, they said, “*The first thing is that we’ve never been really able to ascertain how much she can hear, and how much she can see. We know that her eyes are “fine,” and [sic] her hearing is “fine,” but her ability to process both sets of information is extraordinarily depressed, as in very low. And therefore, it’s been very difficult to know in receiving information whether she grasps it and therefore on top of that getting a reaction and some sort of communicative reaction is also very difficult*.”

#### Meaningful Change

All four caregivers of individuals with SGS were asked, “*What would a meaningful change in communication look like for your child?*” One caregiver was unable to conceptualize the question or provide an answer. The other three caregivers, mentioned communicating likes/dislikes (*n=*2) and communicating pain or illness (*n=*2).

For expressing pain/illness, one caregiver (PI:901) stated, *“But I just feel like you’re always kind of guessing what it is that’s wrong, or that’s right. He’s on a very very strict diet. I don’t know if he likes his food. He eats it. He has a lot of problems with bowel movements. I don’t know if that hurts him. I guess I just wish he could say, “Yes mom, you’re doing everything right.” And that’s hard. ‘Cause it’s never gonna happen.”*

Please note, no caregivers in this group were asked about communication worsening.

### SCN2A-related disorders

#### Demographics

Twelve caregivers of individuals with SCN2A-related disorders participated. The majority were female (10/12) and on average 40.1 years of age (Table S56). Two caregivers self-identified as Hispanic-Latino. Eleven caregivers identified as white and one as Asian. Nine caregivers reported that they were currently married or living with a domestic partner, with three reporting they were single/never married. Overall, the participants reported high levels of education, employment, and annual income (Table S56).

Table S56. Demographic information for 12 caregivers of individuals with SCN2A-related disorders.

| *Caregivers* | *n (%)* |
| --- | --- |
| Female | 10(83.3) |
| Age, years (Mean/SD) | 40.1/9.1 |
| Ethnicity |  |
| Not Hispanic or Latino | 10(83.3) |
| Hispanic-Latino | 2(16.7) |
| Race |  |
| White | 11(91.7) |
| African-American or Black | 0 |
| American Indian/Alaska Native | 0 |
| Asian | 1(8.3) |
| Middle Eastern | 0 |
| More than one race | 0 |
| Relationship status |  |
| Single, never married | 3(25.0) |
| Married, or living with domestic partner | 9(75.0) |
| Separated | 0 |
| Divorced | 0 |
| Widowed | 0 |
| Highest grade in school |  |
| Less than high school diploma | 0 |
| High school degree or equivalent | 0 |
| Some college/University | 5(41.7) |
| College/University degree | 3(25.0) |
| Postgraduate degree | 4(33.3) |
| Occupational status |  |
| Homemaker | 3(25.0) |
| Unemployed | 1(8.3) |
| Retired | 0 |
| On disability | 0 |
| On leave of absence | 0 |
| Full-time employed | 5(41.7) |
| Part-time employed | 3(25.0) |
| Full-time student only | 0 |
| Income of U.S. residents |  |
| Less than $20,000 | 1(8.3) |
| Between $20,001 and $40,000 | 1(8.3) |
| Between $40,001 and $60,000 | 2(16.7) |
| Between $60,001 and $80,000 | 1(8.3) |
| Between $80,001 and $100,000 | 4(33.3) |
| Between $100,001 and $250,000 | 2(16.7) |
| Between $250,001 and $500,000 | 1(8.3) |
| $500,000+ | 0 |
| I prefer not to answer | 0 |
| I do not know | 0 |
| Relation to child |  |
| Mother/Step-mother | 9(75.0) |
| Father/Step-father | 2(16.7) |
| Other – Adoptive Mother | 1(8.3) |

Caregivers also provided demographic information for their child with an SCN2A-related disorder. Children were evenly split in terms of gender, with similar rates to caregivers in regards to ethnicity and race (Table S57).Variants for each child were classified with four as GoF, five as LoF, and three as mixed function. Six caregivers reported their children had an ASD diagnosis, and eight (67%) reported that their child had epilepsy. Many children were receiving therapeutic services including physical, occupational, and speech therapy (Table S57). Six caregivers reported that their child used an AAC device, with four reporting that the device was considered ‘high tech’. The settings in which the device was used varied (Table S57). The mean age of the child when the device was introduced was 4.3 years old (*SD* = 2.0).

When asked about their child’s verbal language, one caregiver indicated their child used ‘full sentences’, one caregiver indicated that their child used ‘short phrases’, one indicated their child used a ‘few words’, and nine indicated their child used ‘no words’.

Table S57. Demographic information for 12 children with SCN2A-related disorders.

| *Children* | *n (%)* |
| --- | --- |
| Age, years (Mean/SD) | 7.7/5.1 |
| Child gender Female | 6(50.0) |
| Ethnicity |  |
| Not Hispanic or Latino | 8(66.7) |
| Hispanic-Latino | 4(33.3) |
| Race |  |
| White | 8(66.7) |
| African-American or Black | 1(8.3) |
| American Indian/Alaska Native | 0 |
| Asian | 1(8.3) |
| Middle Eastern | 0 |
| Native Hawaiian/Other Pacific Islander | 0 |
| More than one race* | 2(16.7) |
| Child’s genotype |  |
| Gain of Function | 4(33.3) |
| Loss of Function | 5(41.7) |
| Mixed Function | 3(25.0) |
| Autism Spectrum Disorder (ASD) | 6(50.0) |
| Epilepsy | 8(66.7) |
| Types of therapy |  |
| Physical Therapy | 8(66.7) |
| Occupational Therapy | 10(83.3) |
| Speech Therapy | 10(83.3) |
| Other therapy  ABA Therapy  Aquatic Therapy  Feeding Therapy  Hippo Therapy  Life Skills  Vision Therapy | 4(33.3)  1(8.3)  1(8.3)  1(8.3)  1(8.3)  1(8.3) |
| Age start speech therapy (Mean/SD) | 2.0/1.3 |
| Age first introduced to device (Mean/SD) | 4.3/2.0 |
| AAC device |  |
| No | 6(50.0) |
| Yes | 6(50.0) |
| Device |  |
| High tech | 4(33.3) |
| Low tech | 2(16.7) |
| Places to use the device |  |
| Home | 4(33.3) |
| School | 5(41.7) |
| Out in the community | 1(8.3) |
| Other - Therapy | 1(8.3) |

Note: *White and African-American or Black (n=1), unspecified (n=1)

#### Typical Communication Ability

The results for this section are organized by behaviors that correspond to expressive, receptive, and pragmatic (i.e. social) communication, aligning with the conceptual framework that was developed for individuals with Angelman syndrome.^1^ Please note, although counts are reported to indicate salience of each theme, these data are limited in that they do not indicate *all* communication behaviors relevant to each child, only the ones that caregivers discussed during the interview.

Most caregivers of individuals with SCN2A-related disorders discussed expressive and receptive communication concepts (Table S58).

Table S58. Number of caregivers that mentioned one or more examples of expressive, receptive, and/or pragmatic communication.

| Communication Behaviors | *n* |
| --- | --- |
| Expressive | 11 |
| Receptive | 7 |
| Pragmatic (Social) | 9 |

##### Expressive

Almost all caregivers (11/12) of children with SCN2A-related disorders discussed expressive communication concepts. Requesting was a common communication concept mentioned by caregivers (Table S59).

Table S59. Expressive communication concepts on the ORCA measure mentioned by caregivers of individuals with SCN2A-related disorders.

| Expressive Communication Function | *n* (total *n*=12*)* |
| --- | --- |
| Seeking Attention | 7 |
| Directing Attention | 3 |
| Refusing an Object | 4 |
| Requesting an Object | 11 |
| Requesting an Object out of View | 1 |
| Requesting More | 2 |

Seven caregivers discussed how their child **sought attention**. Only one caregiver (PI:610) reported that their child used words (specifically, song lyrics) to seek attention. For example, they said, *“I don't know if you know who Laurie Berkner is. She's a children's folk musician and she has a lot of silly songs. So, like there's one that's "I'm Gonna Catch You, You Better Run," so if we're walking in front of her or something and she wants to catch our attention, she might say that.”* The other caregivers described communication behaviors that were not symbolic, but caregivers interpreted them as seeking attention. All five of those caregivers described how their child cried, fussed, or whined to seek attention. Example quotes included:

- “*If he’s really hungry, he’ll kind of do like this, “Ahhh,” like really loud ahs. I think we’ve just kind of picked up over time that that means he wants a bottle because he normally takes it. So, we don’t have any real purposeful communication to be honest with you. I feel like he uses sounds just to either explore his voice or let us know he needs some sort of attention*.” - PI:607
- “*Like when she's wet, she'll squeal out, like scream out a scream. Like right when she goes, when she voids. And she'll kind of like cry little by little until you change her. If someone's eating in the house and she wants their food, she'll squeal for it and then cry if you don't give it to her*.” -PI:611
- “*He whines a lot. Sometimes, he’s just vocalizing to get our attention, so he’s not necessarily in pain or wants something.*” - PI:612
- “…*she will make noises to try to get us over there. Or, she does kind of a slamming motion with her arms and feet as kind of attention-seeking – “Hey, don’t forget about me. I’m over here. Come check me out*.” – PI:608

Three caregivers discussed how their child **directed attention** to an object, action, or event. Example quotes included:

- “*She’ll kinda go from eye contact with you, to eye contact with the item, and then back at you as like, “Hey, look over here,” kind of an idea*.” – PI:608
- “*So, like if she wants to play like for example [with] siblings, she’ll get them, and she’ll grab their hand, and kind of start scooting them over, kind of like pulling at them to kind of them to where she wants to go. And it’s kind of you’re following her lead to kind of see where she’s gonna take you to. But it’s usually her grabbing your arm and kind of trying to push you to the way she needs you to go*.” – PI:602
- “…*if he’s playing in his bedroom with his tractors, he’ll come up and get us and take us back there to see what he’s done and then we’ll play with him and he enjoys that*.” – PI:604

Four caregivers discussed how their child communicated **refusal**. In terms of symbolic communication, one caregiver (PI:605) mentioned that their child could say the word ‘no’ and shake their head. However, this caregiver spoke about challenges with interpretation of their child’s verbal ‘no’, saying, *“She’ll say no, but then I’ll put it in front of her and she’ll really want it. You know, she’ll actually want it. So, then she’ll get distracted and be fine with whatever is given to her, you know? But at first she’ll just say no just to say no.”* Another caregiver said their child used their AAC device to say ‘all done’. The other caregiver said their child utilized their body to indicate refusal (e.g. turning away). One caregiver described non-symbolic communication behaviors (e.g., child simply not engaging in an unwanted activity) that they interpreted as refusal.

Eleven of the 12 caregivers discussed how their child **made requests** during concept elicitation. Ten out of the 11 caregivers indicated that their child utilized symbolic communication to request (e.g. gestures, signs, words, word approximations, AAC device, etc.). Three caregivers also reported other non-symbolic communication behaviors that they interpreted as requesting. One of these children (PI:602, age 4) relied solely on these behaviors. Two out of the 11 children used words to request, but only one of them (PI:610, age 15) was able to self-reference while making requests. For example, the caregiver said, “*She can verbally speak to an extent. She can't really carry on a conversation so she will say words or phrases or she can, you know, ask for something like, "I want hamburger"...”*

Half of the caregivers said their child used gestures/signs (e.g. ASL sign, finger point) to request objects. For example, one caregiver (PI:606) said, “*But so, say, if you have an item, say, a bag of chips, he may tap on it, showing intent*.” Another caregiver (PI:607) said, “*Like he will just bring both hands midline like he’s trying to reach for the bottle and bring it to his mouth. So, that’s the only sort of “I want that” sort of thing that he does*.”

Some caregivers (*n=*3) described their child using eye gaze to request. For example, one caregiver (PI:608) said, “*So, most of her communication style is with her eyes – looking at things, smiling, kinda nodding – encouraging if it’s something that she wants*.” Caregivers also discussed their child using sounds to request. For example, one caregiver (PI:611) said, “*If someone's eating in the house and she wants their food, she'll squeal for it and then cry if you don't give it to her*.” Another caregiver (PI:607) said, “*If he’s really hungry, he’ll kind of do like this, “Ahhh,” like really loud ahs. I think we’ve just kind of picked up over time that that means he wants a bottle because he normally takes it*.”

When describing requesting, three parents described communication behaviors that were not symbolic, but were interpreted by the caregivers as requesting. These caregivers had younger children (ages 5, 4, and 2 years old). These parents described how their child might use physical gestures (taking the caregiver to an object or bringing an object to the caregiver) to request. For example, one caregiver (PI:601) said, “*And he goes to the ice maker and will make a mess, just spray ice all over the floor and everything. So, we’re like “Okay. Okay. Yes. You want water*.” Another caregiver (PI:602) said, “*So, if we’re like sitting and she wants something, she’ll come get our hand and take us, kind of walk us to where she wants us to be leaded to. If she wants something from the refrigerator, she’ll open the refrigerator. If she wants water, she’ll stand next to the water dispenser.*”

Two caregivers (PI:601 and PI:612) discussed how their child **requested more of something** using the ASL sign for “more”. For example, one caregiver (PI:612) said, “*So, he will sign for more, and that’s the only extent of sign language that he uses appropriately and repeatedly, I guess. That’s usually when we feed him or when he wants something, he’ll sign for more*.”

Only one SCN2A caregiver (PI:610) discussed how their child **requested objects that they could not see** using symbolic communication (e.g., words).

##### Receptive

Seven of the 12 caregivers of children with SCN2A-related disorders mentioned receptive communication concepts during concept elicitation. Examples of receptive communication for this group included making choices (*n=*4), responding to their name (*n=*3), understanding isolated words or phrases (*n=*3), following familiar directions (*n=*2), and responding to simple questions (*n=*2).

Four caregivers discussed how their child **made choices** during concept elicitation. One caregiver (PI:612) said their child could choose between different items, but did not describe how. The three other caregivers indicated that their child utilized symbolic communication to make choices (e.g. AAC device, gestures or signs). One caregiver (PI:605) reported that they recently started introducing an AAC device for their child to make choices. This caregiver said they were also trying to encourage their child to use ASL signs to make choices. For example, they said, *“So, right now I think what we’re trying to do is mainly try to stick with sign language and use the tablet [AAC device] for a visual preference if she needs it.”*

Two caregivers (PI:608 & 610) described how their child used gestures (e.g., a finger point or grab) to make a choice. For example, one of the caregivers (PI:608) said, *“So, usually, our selection process is holding both of them up and allowing her to select. Or, putting two down on the mat and offering her that type of possibility. Or, like if…putting on clothes in the morning, we’ll give her two shirts. You know, “Pick out the color you want,” and she’ll grab one...”* The other caregiver (PI: 610) said, “*Or if, for instance, when she's getting ready in the morning and I want to know what she wants to wear, I might give her some choices and pull out some shirts. Or what color shirt do you want to wear? And if she's not answering me then I'll pull out three or four and hold them up and have her pick. Or sometimes I use my hands and just kind of limit things to two options. "Do you want the red shirt or the blue shirt?" And she'll touch which hand for the appropriate choice*.”

**Understanding isolated words** was one of the more common receptive communication skills discussed by caregivers of children with SCN2A. For example, PI:605 remarked, *“Oh, yes. Like there’s certain words we just can’t say. If I’m telling her dad, say, we were at her grandpa’s house, and he has a Jeep for her to ride in, and if I say “Yes, she was in the Jeep,” she’ll automatically catch on to what I’m talking about.”*

##### Pragmatic

Nine caregivers of children with SCN2A-related disorders described the social communication their child engages in. The most commonly described behavior was social smiles with eye contact and excitement (*n=*4). Other examples included using names (*n=*2), greetings (*n=*1), pretend play using gestures (*n=*1), initiating a game (*n=*1), laughing appropriately (*n=*1), and turn-taking (*n=*1). For example, one caregiver (PI:611) described the child’s social smile, saying, “*She does sometimes smile at other people. She'll get excited when she sees someone she knows…Other than that, there's not a whole lot of communication*.”

Two caregivers of individuals with SCN2A-related disorders mentioned that their child used **names for people in their life.** One caregiver reported the child using signs for ‘mom’ and ‘dad’ and the other caregiver (PI:604) said, “*One of the pictures is an older man fishing on a boat. And he’ll say “Grandpa’s on a boat… He names our pets. So, he can sometimes yell at the dog, even if the dog’s not around, [dog’s name], get down.”*

#### Changes in Communication

The research team interviewed 12 caregivers whose children had SCN2A-related disorders and all 12 discussed how their child’s communication changed overtime.

Over half (*n=*6) of the caregivers described regressions that occurred in their child’s communication (Table S60). Please note, any description of ‘major’ or noticeable loss in skills were considered regressions by the study team, even if the caregiver did not use that specific term. Four out of the six caregivers said the regression occurred when their child began experiencing seizures, and the ages that regressions occurred at varied (*n=*2 around 4/5 years old, *n=*4 around age of 1 year). Four out of the six caregivers said the regression occurred when their child began experiencing seizures. Two caregivers said their child also experienced losses in motor ability during the regression. Of the six caregivers that described regressions, three had a child with LoF mutation and three had a child with mixed function mutation.

Table S60. Quotes from caregivers describing regressions (i.e. significant or noticeable loss of skills, typically in multiple domains of functioning).

| Participant ID | Quote |
| --- | --- |
| 601 | *He used to babble and he used to say a couple of words, but all that was lost at around a year.* *He used to say “Mama,” “Papa,” “Agua,” which is water in Spanish. I think those were the main ones. All that went away and he hasn’t been able to say anything after that. I can’t pinpoint to a specific time, but I would say it was around a year, and he had a lot of challenges at that time. He had his first seizures. The seizures that he had were febrile seizures.* |
| 604 | *Pre-seizures, he was far more communicative with other people. He just said a lot more.* *When he started having seizures, it was a very rapid decline pretty much in everything. Seizures increased dramatically and he was in the hospital a lot and not going to school and not receiving therapies. And he lost a lot of mobility and other things in addition to the speech and language.* |
| 606 | *No. I mean, when he was really young and he was doing mama, dada. But once he started having seizures, that basically hit the reset button.* |
| 607 | *As a baby, he did things pretty typical until about six months. He cooed and made noises and did all the things. And then moving into eight or nine months when he were diagnosed, we just hit a huge regression. He wasn’t making eye contact, and I’m not sure that he was present in general for, like I’ve expressed, a long time up until about now. So, his communication has just been not there.* |
| 608 | *So, kinda pre-seizures, she did have words. She had “Mama,” “Dada,” “dog”…* *And all of those have [been] lost – those words don’t exist anymore…* |
| 611 | *She was able to use signs when she was four and five. She knew about ten signs. She's lost all of them.* |

Only a few caregivers described improvements in their child’s communication over time. Some caregivers mentioned new skills like pulling and signing (PI:602), smiling and eye gaze (PI:609), or increases in speech (PI:610).

Two caregivers reported a plateau in their child’s skills (e.g., child’s skills remaining stable over time). For example, one caregiver (PI:604) said, “*I haven’t noticed any new for a very long time. It’s just kind of he’s stalled. I would say that his current baseline he’s had for close to two and a half to three years.*” Another caregiver (PI:605) said, “*I can’t say it’s really improved too much. It’s been about the same. Really, she does just whine a lot and point at things. She’s just frustrated with trying to get that point across, you know? So, not much has really changed with it. It’s always been signs and whining*.”

In this sample, an epilepsy diagnosis was common (8/12), and seizures and seizure-control (i.e. with medication) were linked to changes in communication. For example, one caregiver (PI:608) said, “*When seizures are bad and we’re having hundreds of seizures a day – there’ve been times where communication is completely lost – even the eye contact. And in those scenarios, she’s just trying to get through the day…and so are we. And there’ve been long periods where we’ve been able to have sustained seizure control – no seizures a day for… Our longest period was for a year after seizure onset. And whenever we get control, it’s like this reemergence of communication, and progress, and inch stones that we’re able to work towards and accomplish. And then, loss of seizure control – it’s like an ebb and flow like that. So, through those ebbs and flows, her communication style does change*.” Another caregiver (PI:609) said, “*And then his seizures are uncontrolled, so he would do so good for a few months, and then he would have a very bad month with seizures. So, it was like starting all over again*.”

Several caregivers said learning a new skill requires repetition and modeling. For example, one caregiver (PI:601) said, “*It’s a lot of repetition.* *It takes a long time and if you don’t do it for – for a little bit, for days, like if you stop one day, two days, like that, then he’s gone. He absolutely forgets about it.”* Another caregiver (PI:612) said, *“I mean, he needs constant repetition. And even now, as we’re exploring with the Eyegaze and stuff, it’s kind of inconsistent, obviously – with him looking and us teasing out – “Oh, for sure, he’s doing this with flying colors,” I guess. Now, it’s not that easy.”* One caregiver (PI:610) said other skills may disappear when her child is learning something new, saying, “*It's kind of like her processing, like I said, some of the other skills – I don't think she necessarily regresses, she just kind of puts them on the back burner so she can focus on that one thing. So, she may not demonstrate the skill while she's learning something new, but she may not have completely forgotten it either.”*

#### Hearing & Vision Impacts

The research team interviewed 12 caregivers whose children had SCN2A-related disorders and all 12 were asked whether their child had any hearing or vision issues that impact their ability to communicate.

Only one caregiver of a child with SCN2A (PI:602) suspected their child had a hearing impairment. However, the caregiver said they were having difficulty getting the child tested to confirm. For example, they said, “*They haven’t been able to get a full good test with the headphones because she fights them with that. It’s been pretty hard for them to be able to retest her, but that is still pending. We’ll go back in and try to hopefully get her tested with the headphones to kind of see if there still is something. So, at one point, we were told there was like a mild hearing loss. I don’t know if anything has changed because that’s been a while now.”* This caregiver did not report that the potential hearing impairment had any impact on communication. One other caregiver (PI:606) did not report a hearing impairment, but said his child had sensitive ears. For example, they said, “*No, his ears are fine. He's very – most of these kids, they really, got sensory issues. So, he does wear headphones*.”

Seven of the 12 caregivers reported that their child had cortical visual impairment (CVI). Caregivers provided a variety of impacts they felt CVI had including the ability to recognize faces or objects, eye contact, reading, attention, depth perception and the type of AAC device their child could use (Table S61).

Table S61. Parent-reported impact of cortical visual impairment (CVI) on communication from interviews with caregivers of individuals with SCN2A-related disorders.

| Subject ID | Quote |
| --- | --- |
| 601 | *I will say it’s really hard because he’s not recognizing – I would say my face, mainly. He – it was really hard in the beginning also because he – I mean, I can’t really tell how much he sees of the item he wants or whatever is going on around him* |
| 604 | *The biggest factor is that he’s just so distracted by any kind of movement that’s going on around him. And so, I think it’s hard for him to focus on what’s being said or what’s in front of him. Because anything that moves catches his eye.* |
| 606 | *But like I said, he's got CVI, which is, he can't do steps. I mean, he can go up steps, but he can't go down, because he basically sees a straight line.* |
| 609 | *So, he has a clinical vision impairment. So, he doesn’t come into eye contact a lot. Well, he does probably 60% of the time, 70% of the time he does.* |
| 612 | *So – we set up the page set so there’s only two options, and because of his CVI, there’s challenges with his vision… And with CVI, it’s a lot of the processing time as well. So, it slows everything down* |

#### Meaningful Change

All caregivers during the interviews were asked, “*What would a meaningful change in communication look like for your child?*” If time permitted, most caregivers were asked “*If your child’s communication skills were to decline or get worse, which communication skills would be most important for your child to retain?*”

Parents interpreted “meaningful change” to mean “improvements” in communication ability or skills. In addition, some parents made the distinction between realistic and unrealistic goals they had for their children.

When discussing meaningful improvements for their child with SCN2A-related disorders, caregivers mentioned communicating pain or illness (*n=*3), expressing preferences and having them be understood (*n=*3), consistent communication with any modality (*n=*2), and better communication to reduce frustrating behaviors (*n=*2). A total list can be found in Table S62.

One caregiver (PI:608) discussed how if the child had the ability to express that she was in pain, it would improve the child’s overall quality of life; “*Anything progressing towards being able to do that [communicate pain] more successfully would be huge for our family – particularly with when she’s not feeling good and something’s wrong. For [child’s name], her biggest seizure trigger is visceral pain. Anything kind of internally like gas pain, acid reflux – she’s had kidney stones in the past. Those seem incredibly acute for her – and often trigger seizures. So, trying to understand – usually, when we lose [seizure] control, it’s with one of those going sideways. And then, we’re always kind of left trying to catch up. And retrospectively, I think that there were always kind of signals that something was going on, and we were never able to catch it in time. And it always takes a long time to be able to figure out and diagnose what the problem is, Like, playing the scenario out, if she was able to communicate better and then she was having bad stomach and gas pains, she could have told us about it months ago before losing seizure control, and we could’ve taken interventional steps to prevent it from ever escalating to chronic seizures. Because it always seems like it’s a ramp up, and then once [seizures] are there, then they’re really hard to get back under control.”*

Table S62. Responses to “*What would a meaningful change in communication look like for your child*?” from caregivers of children with SCN2A-related disorders.

| Meaningful Improvement | 601 | 602 | 603 | 604 | 605 | 606 | 607 | 608 | 609 | 610 | 611 | 612 | Total |
| --- | --- | --- | --- | --- | --- | --- | --- | --- | --- | --- | --- | --- | --- |
| Start using verbal words | 1 |  |  |  |  |  |  |  |  |  |  |  | 1 |
| Better communication to reduce frustration/behavior |  | 1 |  |  | 1 |  |  |  |  |  |  |  | 2 |
| Expressing needs using any modality |  |  | 1 |  |  |  |  |  |  |  |  |  | 1 |
| Responding to simple (yes/no questions) |  |  |  | 1 |  |  |  |  |  |  |  |  | 1 |
| Consistent communication with any modality (e.g., AAC, gestures, etc.) | 1 | 1 |  |  |  |  |  |  |  |  |  |  | 2 |
| Communicate if need help/ in danger |  |  |  | 1 |  |  |  |  |  |  |  |  | 1 |
| Communicate pain or illness |  |  |  |  |  | 1 |  | 1 |  |  | 1 |  | 3 |
| Expressing preferences and have them understood |  |  |  |  |  |  | 1 | 1 |  |  |  | 1 | 3 |
| Understand cause and effect |  |  |  |  |  |  |  |  | 1 |  |  |  | 1 |
| Participate in a back-and-forth conversation |  |  |  |  |  |  |  |  |  | 1 |  |  | 1 |

In terms of skills that would be important to retain if skills were being lost, the three caregivers who were asked this question and were able to conceptualize an answer mentioned expressing needs (any modality; *n=*1) and using their physical body to make a request (i.e. going to the thing, taking a person to the item, or bringing the item to the caregiver; *n=*2).

###

### SETBP1-haploinsufficiency disorder (SETBP1-HD)

#### Demographics

Nine caregivers of individuals with SETBP1-HD participated. The majority were female (6/9) and on average 46.3 years of age (Table S63). No caregivers self-identified as Hispanic-Latino. Eight caregivers identified as white and one as Asian. Seven caregivers reported that they were currently married or living with a domestic partner and two reported they were single/never married. Overall, the participants reported high levels of education, employment, and annual income (Table S63).

Table S63. Demographic information for nine caregivers of individuals with SETBP1-HD.

| *Caregivers* | *n (%)* |
| --- | --- |
| Female | 6(66.7) |
| Age, years (Mean/SD) | 46.3/6.6 |
| Ethnicity |  |
| Not Hispanic or Latino | 9(100.0) |
| Hispanic-Latino | 0 |
| Race |  |
| White | 8(88.9) |
| African-American or Black | 0 |
| American Indian/Alaska Native | 0 |
| Asian | 1(11.1) |
| Middle Eastern | 0 |
| Native Hawaiian/Other Pacific Islander | 0 |
| More than one race | 0 |
| Relationship status |  |
| Single, never married | 2(22.2) |
| Married, or living with domestic partner | 7(77.8) |
| Separated | 0 |
| Divorced | 0 |
| Widowed | 0 |
| Highest grade in school |  |
| Less than high school diploma | 0 |
| High school degree or equivalent | 2(22.2) |
| Some college/University | 1(11.1) |
| College/University degree | 0 |
| Postgraduate degree | 6(66.7) |
| Occupational status |  |
| Homemaker | 1(11.1) |
| Unemployed | 0 |
| Retired | 0 |
| On disability | 0 |
| On leave of absence | 0 |
| Full-time employed | 7(77.8) |
| Part-time employed | 1(11.1) |
| Full-time student only | 0 |
| Income of U.S. residents |  |
| Less than $20,000 | 0 |
| Between $20,001 and $40,000 | 2(22.2) |
| Between $40,001 and $60,000 | 1(11.1) |
| Between $60,001 and $80,000 | 1(11.1) |
| Between $80,001 and $100,000 | 2(22.2) |
| Between $100,001 and $250,000 | 2(22.2) |
| Between $250,001 and $500,000 | 1(11.1) |
| $500,000+ | 0 |
| I prefer not to answer | 0 |
| I do not know | 0 |
| Relation to child |  |
| Mother/Step-mother | 6(66.7) |
| Father/Step-father | 3(33.3) |

Caregivers also provided demographic information for their child with SETBP1-HD. Children were almost evenly split in terms of gender (5 females; 4 males), with similar rates to caregivers in regards to ethnicity and race (Table S64). Based on classifications made by the foundation upon entry into their community, four children had a ‘nonsense’ mutation, two had ‘frameshift’, two had ‘missense’, and one had ‘deletion’.

One caregiver reported their children had an ASD diagnosis. No caregiver reported that their child had epilepsy. Many children were receiving therapeutic services including physical, occupational, and speech therapy (Table S64). Three caregivers reported that their child used an AAC device with two reporting the device was considered ‘high tech’. The settings in which the device was used varied (Table S64). The mean age of the child when the device was introduced was 3.3 years old (*SD* = 1.2).

When asked about their child’s verbal language, two caregivers indicated their child used ‘full sentences’, five caregivers indicated that their child used ‘short phrases’, one indicated their child used a ‘few words’, and one indicated their child used ‘no words’.

Table S64. Demographic information for nine children with SETBP1-HD.

| *Children* | *n (%)* |
| --- | --- |
| Age, years (Mean/SD) | 8.4/4.6 |
| Child gender Female | 5(55.6) |
| Ethnicity |  |
| Not Hispanic or Latino | 9(100.0) |
| Hispanic-Latino | 0 |
| Race |  |
| White | 7(77.8) |
| African-American or Black | 0 |
| American Indian/Alaska Native | 0 |
| Asian | 2(22.2) |
| Middle Eastern | 0 |
| Native Hawaiian/Other Pacific Islander | 0 |
| More than one race | 0 |
| Child’s genotype |  |
| Missense | 2(22.2) |
| Deletion | 1(11.1) |
| Frameshift | 2(22.2) |
| Nonsense | 4(44.4) |
| Autism Spectrum Disorder (ASD) | 1(11.1) |
| Epilepsy | 0 |
| Types of therapy |  |
| Physical Therapy | 4(44.4) |
| Occupational Therapy | 7(77.9) |
| Speech Therapy | 7(77.9) |
| Other therapy  ABA Therapy  Behavioral Therapy  Play Therapy | 1(11.1)  2(22.2)  1(11.1) |
| Age first introduced to device (Mean/SD) | 3.3/1.2 |
| AAC device |  |
| No | 6(66.7) |
| Yes | 3(33.3) |
| Device |  |
| High tech | 2(22.2) |
| Low tech | 1(11.1) |
| Places to use the device |  |
| Home | 2(22.2) |
| School | 3(33.3) |
| Out in the community | 0 |
| Other – Therapy | 1(11.1) |

#### Typical Communication Ability

The results for this section are organized by behaviors that correspond to expressive, receptive, and pragmatic (i.e. social) communication (Table S65), aligning with the conceptual framework that was developed for individuals with Angelman syndrome.^1^ Please note, although counts are reported to indicate salience of each theme, these data are limited in that they do not indicate *all* communication behaviors relevant to each child, only the ones that caregivers discussed during the interview.

Table S65. Number of caregivers that mentioned one or more examples of expressive, receptive, and/or pragmatic communication.

| Communication Behaviors | *n* |
| --- | --- |
| Expressive | 9 |
| Receptive | 8 |
| Pragmatic (Social) | 8 |

##### Expressive

All caregivers of children with SETBP1-HD discussed expressive communication concepts. Requesting was a communication concept mentioned by all caregivers (Table S66).

Table S66. Expressive communication concepts on the ORCA measure mentioned by caregivers of individuals with SETBP1-HD.

| Expressive Communication Function | *n* (total *n=9*) |
| --- | --- |
| Seeking Attention | 4 |
| Directing Attention | 4 |
| Refusing an Object | 5 |
| Requesting an Object | 9 |
| Requesting an Object out of View | 1 |
| Requesting More | 2 |
| Asking Questions | 2 |
| Telling Stories | 3 |

Four caregivers spoke about how their child typically **sought** **attention** using words/word approximations. For example, one caregiver (PI:807) said, *“But when he wants to speak very quickly, he says “Mom or Dad, come, come.”* One parent (PI:806) also described how their child might use physical gestures (such as grabbing and pulling) to seek attention when their symbolic communication doesn’t accomplish their goal. For example, they said, “*Firstly, he gets whoever…attention that he wants to communicate with. He ensures that he gets their attention. If he is struggling to communicate any which way to whoever he’s talking to, he will eventually try to show them what he’s talking about. So, he’ll grab you by the hand….”*

Four caregivers spoke about how their child **directed attention**. Three out of the four caregivers indicated that their child utilized symbolic communication to direct attention (e.g. gestures, signs, words, word approximations, AAC device). For example, one caregiver (PI:802) said, “*She says, “Daddy, see.”… Or another thing I’m trying to move her away from in some ways, she just grunts sometimes. And points and grunts.”* Another caregiver (PI:805) said, “*She’ll grab your hand and pull you to stuff. She’ll gesture like this, like to come here, to follow me*.” Some caregivers also reported other non-symbolic communication behaviors (e.g., bringing the caregiver places or bringing things to the caregiver) that they interpreted as directing attention. One caregiver (PI:806) only described these behaviors. For example, they said, “*So, he’ll grab you by the hand and walk you over to something that he’s wanting or wanting to do, right? So, if he wants to color or – he’ll bring me to his crayons or something like that*.”

The research team interviewed nine caregivers whose children had SETBP1-HD and all nine discussed how their children **communicated requests** during concept elicitation. All caregivers provided examples of how their child requests using symbolic communication (Table S67). The most commonly requested objects were food and drink, but individuals with SETBP1-HD in our sample also frequently requested TV, music, specific toys, places and activities (e.g., outside). Many caregivers (*n=*5) said their child could self-reference while requesting. One caregiver (PI:807) also said their child used manners while requesting. For example, they said, “*Please could you – Mommy, could you give me the….*”

Table S67. Examples of requests provided by caregivers of individuals with SETBP1-HD.

| Subject ID | Quote |
| --- | --- |
| 801 | *“Mama, I’m hungry,” always. She would request what she wants. She would say, can we go to so-and-so’s house? Can we go out to play? Can we go to the park? So, she would use all those words properly for short sentences.* |
| 802 | *I’m really trying to work on her, to move away from what she said, her typical phrase, which is, “I need.” I’m trying to get her to say, “I would like,” or, “I want,” and to kind of stretch out her vocabulary. But honestly, that’s her go to, her habit, is to say, “I need.”*  *She says, “Daddy, I’m hungry.”* |
| 804 | *He’ll say, you know, Mommy, can I have an insert name of whatever he wants. So, he’ll use a full sentence to ask for it… or can I have this, can I have that.* |
| 805 | *Sometimes, like if she wants to dance, she’ll say like shake your butt, because she wants to like – her wants her dad to put music on. She’ll say like go outside or go upstairs, or like bath time.*  *Like she’ll say yogurt or she’ll go to the fridge and open it and get a yogurt out*. |
| 806 | *They're very short sentences and whatnot, but they're starting to come more. He’ll say, “I want water,” or –so, as an example. Or “I need help.”* |

Five caregivers also discussed how their child uses gestures/signs to request objects. For example, one caregiver (PI:806) said, “*He usually rubs his tummy [for hungry], and he communicates a lot by grabbing your arm or hand or something… He’ll maybe put his hand closer to his mouth and pretend like he’s eating.”* Another caregiver (PI:810) said, *“He uses a combination of signs that his therapists have taught him... Like he’ll make the signal for he’s hungry. He wants something to eat.”* Two caregivers provided examples of how their child paired multiple modalities (gestures/signs and sounds) together to request. For example, one caregiver (PI:805) said, “*She has [a] little motorized car, so if she wants to go ride her car, you know, like she’ll try to open the door and she’ll grunt or like, you know, tap the door, because she wants somebody to open it*.” Another caregiver (PI:810) said, “*He will make sounds. Like he’ll point towards the kitchen. He’ll go eh-eh-eh if he wants something*.”

Two caregivers discussed how their child **requested more of something** using words or an ASL sign. For example, one caregiver (PI:805) said, “*She can use lots of signs, but she can use – she will use more, drink, mom, dad. Like all those, she does regularly*.” The other caregiver (PI:810) said, “*And he started to say more. He said more a couple of times. But he won’t use it a lot. He’ll say it just kind of out of the blue, kind of randomly. But then he won’t say it again for a while*.”

One caregiver (PI:805) discussed how their child **requested objects that they could not see** using words. For example, they said, “*Bubbles, she’s like obsessed with like adults chewing gum and blowing bubbles, so she’ll ask for bubbles*.”

Five caregivers discussed how their child **communicated refusal**. Three caregivers described gestures/signs that their child used to refuse something. For example, one caregiver (PI:805) said, “’*All done,’ [sign] she does regularly. Like when she wants out of her high chair, she’ll say all done.”* Another caregiver (PI:810) described how their child used a modified gesture/sign to communicate refusal, saying, “*[the child will] wave his hands side by side…it’s not the proper sign language symbol for all done. That’s kind of how he does it. But we know what it is.”* Two caregivers indicated that their children used words or word approximations to communicate refusal. For example, one caregiver (PI:806) said, *“So, if you ask him to do something, he’ll do it. Sometimes he’ll say no, sometimes he’ll say yes ‘cause those are words that we worked on.”* One caregiver (PI:810) indicated that their child used an AAC device as one of the ways their child communicates refusal and another caregiver (PI:804) indicated that their child would sometimes hit to communicate refusal, saying, *“Depending on his mood, he will either say, you know, no, or I don’t want to do that, or if he’s in a bad mood, he’ll hit.”*

Three caregivers spoke about how their children **asked questions**. For example, one caregiver (PI:801) shared, “*She would say, can we go to so-and-so’s house? Can we go out to play? Can we go to the park? So, she would use all those words properly for short sentences.”* Another caregiver (PI:802) shared that they believed their child does ask questions, but this required some interpretation by the communication partner: “*I mean, it’s clear to me, but not always kind of linguistically clear. She’ll create a little narrative, but with a little question at the end. […] Like ‘My mom’s dog.’ ‘[name] go away,’ ‘[name] go away today too.’ Because my sister went somewhere far away. She’d say something like that, to which I’d answer, ‘No, no, no, she’s at home. She’s at home.’*”

Three caregivers spoke about how their child **told stories** about something that happened in the past. Example quotes from these caregivers included:

- “*I don’t know if that is the right word to use, but she has a lot of, I don’t know, subject hopping, memory hopping. She would talk about something, and then she would relate that something with something else, which happened a week ago, or even five years ago. It’s very random. Things keep popping up in her head*… *[for example] When I say okay, we are going to someone’s house in this area, she knows the way. She recognizes places. So, when I say we’re going to someone’s house, and then she would say, “Oh, we’re going to play in their house.” And she would remember that the last time we went to their house, she ate something from McDonald’s. And then, she would say, “Oh, there’s a library near McDonald’s, and I like to go to the library.” And I want – her name, and she wants to play there. And then, suddenly, she would remember, oh, there is something with – bad, which happened. There was a firetruck there, and she is sensitive to sound. And she suddenly, get very agitated. And she would say, “Okay, I don’t wanna go anymore*.” – PI:801
- “*So, it’s such a cute, funny thing she does. She just starts, and she’ll say, “Bad news:” Like… “Bad news: my neighbors drive by my house at night, make noise.” That’s kind of one of her most complicated statements she’s ever made. Verbal. Clear… Or “Bad news: a bear ate a bird.” She makes things up. Or “I saw a bear and a deer fighting*.” – PI:802
- “*If he’s trying to tell me about something, let’s say, that like happened at school or something that happened before, that’s an area that he really struggles with. He’ll tell me, you know, a couple things, like you know, we went to – you know, they had a field trip the other day. We went to the baseball stadium, but he won’t – he kind of waits for me to ask a question, and then he’ll answer it, but he’s not capable of continuing a paragraph discussion of like I saw this, and then I went here, and then I went that. You know, I went to the baseball stadium, and then I’ll ask, “So, what did you see?” And then, he’ll tell me what he saw. You know, what did you do? He’ll tell me what he did, but he doesn’t have quite the ability to just put it all together in one cohesive narrative*.” – PI:804

##### Receptive

Eight caregivers of children with SETBP1-HD mentioned receptive communication concepts during concept elicitation. Examples of receptive communication concepts for this group included following familiar directions (*n=*6), responding to simple questions (*n=*4), following novel directions (*n=*3), making choices (*n=*1), turning toward their name (*n=*1), and understanding isolated words/phrases (*n=*1).

Six participants discussed how their child **followed familiar two-step directions**. In addition, three of those participants also discussed how their children followed new/novel directions. One caregiver (PI:806) gave the example, *“If I ask him to go get something, I can just tell him, “[Child’s name], can you go get daddy a paper towel?” And he’ll grab a piece of paper towel and bring it to me. So, he follows instructions no problem at all.*” Another caregiver (PI:804) spoke about their child following new directions at soccer practice, *“Yeah, and he can follow directions pretty well. Like he plays on a recreational soccer team and he does well at practice and follows directions from the coaches and will communicate with them."*

Another caregiver (PI:810) gave an example of their child **responding to a simple question**, “*If you ask him a question like, “Hey, [Child’s name]. Do you want to go swinging?” and he’s doing something. He'll stop and say, “Yes,” or make the sign for yes.*”

##### Pragmatic

Eight caregivers of individuals with SETBP1-HD discussed pragmatic communication concepts during concept elicitation. Concepts included greeting people (*n=*7), laughing appropriately (*n=*1), pretend play (*n=*1, this child played with Barbie dolls), comforting others (*n=*1), and following rules/turn taking in a game (*n=*1).

One caregiver (PI:804) discussed how their child **plays games**. They said, “*It depends on the level of the board game. He’s nine, but he can’t play at the level of a nine-year-old. If, you know, there’s like games like Scrabble Junior, which is meant for kind of younger kids. He can play that with someone else and you don’t have to guide him. He knows what to do. You know, so someone takes a turn. He takes his turn for Sorry. The game Sorry, he likes to play that. You know, he knows to take the card, move his pieces, and then stuff like that. If it’s a more complicated game like that involves more reading or, you know, like Clue. He can’t play that without guidance.*”

Two caregivers, (PI:801 & PI:804) discussed the challenges their children have with taking turns in **conversations**. For example, one caregiver (PI:801) stated, “*If she goes to the park, she would say hi to other children. And she would say “Hi,” and “what’s your name?” And they would say their name, and they would ask hers. And she would say her name too. And she does not know what to say after that…So, she would just – she’s stuck, if they ask her, how old are you or something, she does not know the answer to, she would just think for a bit, and then just laugh. And then – or after that, she would just babble something*”.

One caregiver (PI:806) mentioned that their child **comforts others**, *“He’s good with kids. I know that, and in school he’s very popular. He’s very empathetic type, so, he tends to respond to people that are not feeling good or got hurt and he consoles them.”*

Another caregiver (PI:808) discussed how their child can feel isolated, “*I guess this is probably typical of any child that’s different than other kids. She has a lot of sadness because she feels left out or kids are making fun of her or – but she can talk to them and tell them anything she wants to tell them.”* The description of the child feeling sadness because of social isolation has not come up in previous interviews.

#### Changes in Communication

The research team interviewed nine caregivers whose children had SETBP1-HD and all nine discussed how their child’s communication changed overtime. Almost every caregiver described improvements in their child’s verbal language (Table S68).

Table S68. Quotes from SETBP1-HD caregivers describing improvements in their child’s communication skills over time.

| Participant ID | Quote(s) |
| --- | --- |
| 801 | *She surprises us every now and then with new words and phrases… And I think she’s got a lot of phrases by just listening to people and listening to us or listening to the television, her programs on the television. And sometimes, she surprises – like, what’s going on here, or something’s fishy. These are the phrases we would not use in everyday language, but one’s she’s caught on from the TV, and she knows exactly where to use.*  *I feel like that suddenly, she’s understanding more things. Suddenly, she has matured. Suddenly, she’s talking more. And I think, compared to three years ago, I feel she understands more now than before… I feel now she listens, she understands better. She tries to follow instructions better than two, three years ago…* |
| 802 | *But one thing I have noticed that she does changed recently is that she talks to herself a little bit while she’s doing something else. That’s developed. I mean, her glimpses of that before, but now consistently, I can hear her. Again, not often. But like yesterday, I heard her talking to her dolls when she was doing something. I registered that as a significant development.* |
| 803 | *When she was six…she didn’t have any communication… she knew two words. So, she’s learning a lot of new words. She’s making short sentences. And she’s relying less on her facial expressions.* |
| 804 | *You know, now, we can have a full conversation with him about how his day is. You know, it may not be quite as detailed as the conversations I have with his sister who’s ten, but you know, I don’t have to – I can ask him about his day and he can generally tell me at least parts of it, maybe not all of it.* |
| 805 | *She’s just attempting to say more words. Like now, she’s saying colors, and letters, she’s counting. She can say her full name…* |
| 806 | *It’s definitely improved. He’s got some singular words and with some assistance, he can make a sentence. But we’re still working on them. They're very short sentences and whatnot, but they're starting to come more. He’ll say, “I want water,” or –so, as an example. Or “I need help.”…* *He maybe had five words in total prior to seeing this speech therapist. So, I think now, we’re around 30 or 40 words.* |
| 807 | *Now he says more words than before. Now we can have two words, action verbs…* |
| 810 | *And every week, he’s learning more and more signs with his speech therapist.* |

When discussing the perceived speed of changes in communication, five caregivers characterized these changes as *slow*. For example, one caregiver (PI:807) said, “*And the changes are very slow…during one year, we see very few improvements, a little improvement*.” One caregiver (PI:810) said their child can learn new ASL signs within a week and also learned how to use their AAC device “*fast*”. Four caregivers specified the ages they saw the most improvements, the ages included 4-6, 6-11, 8, and 10 years.

This group had a number of children who used full sentences or short phrases (7/9). As such, some caregivers (*n=*4) detailed the development of their child’s verbal language and how they progressed through utilization of different pre-verbal modalities. Three caregivers described their children using sounds and ASL signs prior to gaining words, and then once they gained verbal speech, dropped these other modalities. They noted that their child’s verbal speech began around school age (4-5 years old). For example, one caregiver (PI:801) said, “*Around 1 year, she started – I think first, she started saying mama and those same sounding words, mama, and dada, and you know… she started babbling. I [also] started trying to teach her some signs since she was 8 months… And she actually picked it up… She was able to sign that it’s hot, or she wanted water, so just signs and gestures*… *And [then] she was going to school, preschool, early intervention. And she just took off, I guess, from like 5, 6, 7 [with verbal language].* Another caregiver (PI:804) said, ““*He didn’t talk until he was three. He wasn’t really talking in sentences until he was, you know, late four, maybe five”.* Another caregiver (PI:802) said, “*Almost no sign language [now]. We used to use I would say a fair amount until she was about – oh – two years ago or so… her language has just exploded since she started kindergarten last year, and then school.”*

Some caregivers (*n=*4) mentioned learning a new skill requires repetition and modeling. For example, one caregiver (PI:803) said, “*If she doesn’t repeat things again, and again, and again, then she’ll lose them, like the days of the weeks or the months of the year. If she doesn’t repeat those, then she’ll forget*.” Another caregiver (PI:807) said, “*We have to repeat, repeat, repeat*.”

A couple of caregivers said that other skills may stay “*stagnate*” when their child is trying to learn a new communication skill. One caregiver (PI:810) said new skills can be inconsistent and fleeting. For example, they said, “*He said more a couple times. But he wont use it a lot. He’ll say it just kind of out of the blue, kind of randomly. But then he wont say it again for a while*.”

#### Hearing & Vision Impacts

All nine caregivers reported that their child had no formal **hearing impairment**. However, one caregiver (PI:808) said their child had tubes placed in their ears at one point, but had passed a recent hearing test. For example, they said, “*She has had tubes put in her ears one time. She’s never had to have them replaced, or as far as I know, they're still intact. She recently passed a hearing test at the end of last year*.” Another caregiver (PI:801) said it has been difficult getting their child’s hearing tested but suspects their child has “sensitive ears”. For example, they said, “…*well, she has sensitive hearing, and she is sensitive to sound.”*

Four caregivers of children with SETBP1-HD reported that their child had a **vision impairment**. Impairments included astigmatism (*n=*2), colorblindness (*n=*1), and farsightedness (*n=*1). No caregiver described any impacts of these impairments on their child’s communication. One caregiver (PI:801) of a child with astigmatism said their child needs glasses but the family had not taken steps to fit the child for glasses. For example, they said, “*I’m sure she needs glasses. Doctors are sure she needs glasses… for astigmatism, but we haven’t found a prescription for her, the power for her glasses yet. So, I’m not in a rush to get her glasses because she wouldn’t keep them on, and they are quite expensive*.”

#### Meaningful Change

All caregivers during the interviews were asked, “*What would a meaningful change in communication look like for your child?*” If time permitted, most caregivers were asked “*If your child’s communication skills were to decline or get worse, which communication skills would be most important for your child to retain?*”

Parents interpreted “meaningful change” to mean “improvements” in communication ability or skills. In addition, some parents made the distinction between realistic and unrealistic goals they had for their child. Three parents of children with SETBP1-HD said that improved verbal pronunciation would be meaningful for their child. One caregiver stated (PI:801), *“Definitely more, the clarity of pronunciation. She still would – there are some [sounds] like starting a word with R, she would say L. For room, she would say loom. ”* Other responses can be found in Table S69.

Table S69. Responses to “*What would a meaningful change in communication look like for your child*?” from caregivers of children with SETBP1-HD.

| Meaningful Improvement | 801 | 802 | 803 | 804 | 805 | 806 | 807 | 808 | 810 | Total |
| --- | --- | --- | --- | --- | --- | --- | --- | --- | --- | --- |
| Improved verbal pronunciation | 1 |  |  | 1 |  |  |  | 1 |  | 3 |
| Staying on-topic, not interrupting with the communication partner | 1 | 1 |  |  |  |  |  |  |  | 2 |
| Improve/increase eye contact with communication partner |  | 1 |  |  |  |  |  |  |  | 1 |
| Improved verbal word finding |  |  | 1 |  |  |  |  |  |  | 1 |
| Telling about events with details |  |  |  | 1 | 1 |  |  |  |  | 2 |
| Engage in back-and-forth conversation with another person |  |  |  |  | 1 |  |  |  |  | 1 |
| Communicate preferences using words |  |  |  |  | 1 |  |  |  |  | 1 |
| Initiate communication with other people using words |  |  |  |  |  | 1 |  |  |  | 1 |
| More words using verbal speech |  |  |  |  |  |  | 1 |  |  | 1 |
| Consistence use of verbal sentences |  |  |  |  |  |  | 1 |  |  | 1 |
| Start using verbal words |  |  |  |  |  |  |  |  | 1 | 1 |

In terms of skills that would be important to retain if skills were being lost, the four caregivers who were asked this question mentioned verbal speech (*n=*3), gestures (*n=*1), AAC device usage (*n=*1), and being able to communicate if they needed help or if they were in danger (*n=*1).

### STXBP1-related disorders

#### Demographics

Twelve caregivers of individuals with STXBP1-related disorders participated. The majority were female and on average 39.3 years of age (Table S70). Two caregivers self-identified as Hispanic-Latino. Ten caregivers identified as white, 1 as African American/Black, and 1 as mixed race. Most caregivers (10/12) reported that they were currently married or living with a domestic partner, with 2 caregivers indicating they were single/never married. Overall, the sample reported high levels of education, employment (12/12 employed at least part-time), and annual income (Table S70).

Table S70. Demographic information for 12 caregivers of individuals with STXBP1-related disorders who participated in the qualitative interview study.

| *Caregivers* | *n (%)* |
| --- | --- |
| Female | 10(83.3) |
| Age, years (Mean/SD) | 39.3/5.2 |
| Ethnicity |  |
| Not Hispanic or Latino | 10(83.3) |
| Hispanic-Latino | 2(16.7) |
| Race |  |
| White | 10(83.3) |
| African-American or Black | 1(8.3) |
| Asian | 0 |
| Middle Eastern | 0 |
| More than one race* | 1(8.3) |
| Relationship status |  |
| Single, never married | 2(16.7) |
| Married, or living with domestic partner | 10(83.3) |
| Separated | 0 |
| Divorced | 0 |
| Widowed | 0 |
| Highest grade in school |  |
| Less than high school diploma | 0 |
| High school degree or equivalent | 1(8.3) |
| Some college/University | 4(33.3) |
| College/University degree | 2(16.7) |
| Postgraduate degree | 5(41.7) |
| Occupational status |  |
| Homemaker | 0 |
| Unemployed | 0 |
| Retired | 0 |
| On disability | 0 |
| On leave of absence | 0 |
| Full-time employed | 10(83.3) |
| Part-time employed | 2(16.7) |
| Full-time student only | 0 |
| Income of U.S. residents |  |
| Less than $20,000 | 0 |
| Between $20,001 and $40,000 | 0 |
| Between $40,001 and $60,000 | 2(16.7) |
| Between $60,001 and $80,000 | 1(8.3) |
| Between $80,001 and $100,000 | 1(8.3) |
| Between $100,001 and $250,000 | 5(41.7) |
| Between $250,001 and $500,000 | 3(25.0) |
| $500,000+ | 0 |
| I prefer not to answer | 0 |
| I do not know | 0 |
| Relation to child |  |
| Mother/Step-mother | 10(83.3) |
| Father/Step-father | 2(16.7) |

Note: *White and Middle Eastern

Caregivers also provided demographic information for their child with an STXBP1-related disorder. Five children were male, with similar rates to caregivers in regards to ethnicity and race (Table S71). Mutation types were classified via participation in the Ciitizen registry, which required submission of genetic reports. Within Ciitizen, the variants are classified using the process recommended by the American College of Medical Genetis and Genomics^81^ and include variant interpretation by a trained individual at the clinical lab that conducted the sequence testing (e.g. GeneDx, Invitae, etc). To further confirm mutation type, the c. and p. addresses included within the child’s genetic report were recorded by the Duke study team during the screening process. Mutation types included: 4 missense, 3 splice-site, and 1 each of deletion/duplication, frameshift, nonsense, and partial deletion. One participant did not provide c. and p. addresses to the Duke study team in order for their child’s variant type to be classified.

Five caregivers reported their children had an ASD diagnosis, and 9/12 reported that their children had epilepsy. The majority of children were receiving therapeutic services, including physical, occupational and speech therapy (Table S71). Some caregivers (5/12) reported their child used an AAC device, with 3 parents reporting that their child’s device was considered ‘high tech’ (e.g. iPad) and 2 ‘low tech’ (e.g. picture book). The settings in which the device was used varied (Table S71). The mean age of the child when the device was introduced was 4.6 years old (*SD* = 4.3).

When asked about their child’s current verbal language, no caregivers indicated their child used ‘full sentences’, 2 caregivers indicated that their child used ‘short phrases’, 3 indicated their child used a ‘few words’, and 7 indicated their child used ‘no words’.

Table S71. Demographic information for children with STXBP1-related disorders.

| *Children* | *n (%)* |
| --- | --- |
| Age, years (Mean/SD) | 8.3/4.5 |
| Child gender Female | 7(58.3) |
| Ethnicity |  |
| Not Hispanic or Latino | 9(75.0) |
| Hispanic-Latino | 3(25.0) |
| Race |  |
| White | 8(66.7) |
| African-American or Black | 2(16.7) |
| American Indian/Alaska Native | 0 |
| Asian | 0 |
| Middle Eastern | 0 |
| Native Hawaiian/Other Pacific Islander | 0 |
| More than one race* | 2(16.7) |
| Child’s genotype |  |
| Deletion/Duplication | 1(8.3) |
| Frameshift | 1(8.3) |
| Missense | 4(33.3) |
| Nonsense | 1(8.3) |
| Partial deletion | 1(8.3) |
| Splice-site | 3(25.0) |
| Unknown | 1(8.3) |
| Autism Spectrum Disorder (ASD) | 5(41.7) |
| Epilepsy | 9(75.0) |
| Types of therapy |  |
| Physical Therapy | 10(83.3) |
| Occupational Therapy | 9(75.0) |
| Speech Therapy | 10(83.3) |
| Other therapy  ABA Therapy  Autistic Learning Support  Behavioral Therapy  Hippo Therapy  Music Therapy  Vision Therapy | 2(16.7)  1(8.3)  1(8.3)  1(8.3)  1(8.3)  1(8.3) |
| Age start speech therapy (Mean/SD) | 1.6/1.1 |
| Age first introduced to device (Mean/SD) | 4.6/4.2 |
| AAC device |  |
| No | 7(58.3) |
| Yes | 5(41.7) |
| Device |  |
| High tech | 3(25.0) |
| Low tech | 2(16.7) |
| Places to use the device |  |
| Home | 3(25.0) |
| School | 4(33.3) |
| Out in the community | 0 |
| Note: *White and Middle Eastern (n=1), White and African-American or Black (n=1) |  |

#### Typical Communication Ability

The results for this section are organized by behaviors that correspond to expressive, receptive, and pragmatic (i.e. social) communication, aligning with the conceptual framework that was developed for individuals with Angelman syndrome.^1^ Please note, although counts are reported to indicate salience of each theme, these data are limited as they do not indicate all communication behaviors relevant to each child, only the ones that caregivers discussed during the interview.

All 12 caregivers of children with STXBP1-related disorders spoke about their child’s typical communication, with expressive communication concepts being mentioned by all caregivers (Table S72). Most caregivers (7/12) also mentioned aspects of pragmatic (social) communication.

Table S72. Number of caregivers that mentioned one or more examples of expressive, receptive, and/or pragmatic communication during concept elicitation.

| Communication Behaviors | *n* |
| --- | --- |
| Expressive | 12 |
| Receptive | 8 |
| Pragmatic (Social) | 7 |

##### Expressive

All 12 caregivers of children with STXBP1-related disorders discussed different types of expressive communication concepts. The most common concept mentioned by caregivers was requesting (Table S73).

Table S73. Expressive communication concepts on the ORCA measure mentioned by caregivers of children with STXBP1-related disorders.

| Expressive Communication Function | *n* |
| --- | --- |
| Seeking Attention | 2 |
| Refusing an Object | 3 |
| Requesting an Object | 12 |
| Requesting an Object out of View | 5 |
| Requesting More | 6 |
| Telling Stories | 1 |

The modalities used by children with STXBP1-related disorders to **make requests** were varied and included words/word approximations (*n=*4), gestures/signs (e.g. finger point, American Sign Language, modified gesture/sign; *n=*8), AAC devices (*n=*3), sounds (*n=*2), and eye gaze (*n=*1). Seven caregivers also mentioned physical body movements that require caregiver interpretation (e.g. bringing an object to the caregiver). Examples of requesting included:

- “*She most frequently will use the “I want” button [on the AAC device] when she’s going in or the people button. If she uses the people button, there are pictures of people that are basically just the people I just named, her parents, grandparents, sibling, and nanny. So, she can ask for people.*” – PI:210
- “*So, she'll request, she’ll sign "book" a lot. Pointing…She can sign, “more.” She’ll sign for “thirsty” by putting her finger down, like a line on her throat. And sometimes she'll sign for – like, that shows to eat by putting her fingers to her mouth*.” – PI:212
- “*For something to drink, he does actually the more sign with his hands. We’ve gotten to know that means he wants something to drink.*” – PI:203
- “*She’ll say, “I want car,” “I want Papa,” “I want Grandma.” She’ll say, “I want pool,” “I want outside;” that’s usually the things that she says when she wants to go somewhere*.” – PI:205
- “*Then, she will use [her AAC device to say], “I want,” and when she goes into “I want,” there’s options like, “I want to play,” “I want to watch,” “I want to eat,” “I want to drink,” and most frequently she does go into, “I want to play.” Then, there are choices with different toys and activities that she can choose from. Occasionally, she’ll use, “I want to watch,” and there are programs, TV shows and such that she can choose from*.” – PI:210
- *“He will go and try to engage with whatever it is that he wants whether that be food in the pantry. So, he will go over to the pantry and try to go into the pantry to tell us he’s hungry.* *He’ll either throw [plates] on the floor, or he’ll actually bring them over to the counter and set them down. He’s actually brought them to me before... So, he’s trying to tell me, hey, I want to eat.”* – PI:203
- *“She usually does it by actually going to where the food is that she wants, and actually getting it. Or if for some reason she can’t reach it, she will kind of rummage around to where we can tell she’s trying to get something, and then we can kind of help her look at her options.”* – PI:202

Two caregivers provided examples of how their child **seeks their attention**:

- “*[the child uses] kind of just like “ahah,” you know when they’re trying to get your attention. Just “eheh,” “uhuh*.” – PI:207
- “*She will cry, she will definitely look at you for a long period of time to get your attention, and she will absolutely pull on your shirt to get your attention.”* – PI:211

Three caregivers discussed how their children **refused** **objects and activities**:

- “*Then, he started telling us [using the AAC device] stop, stop, stop. Then, go, go, go. Then, he got up and was trying to leave. He was telling us he wanted to stop doing this assessment and that he wanted to go*.” – PI:203
- “*When she’s really trying to refuse something, she will physically push it away. That’s kind of the most common way I see her refusing is just she’ll take an item or a person or whatever she’s refusing and just close her hand on it and just push it away*…*If she pushes you away or whines about something, you know that she’s unhappy about it*.” – PI:210
- “*And when she’s eating she will show you a clear preference through you know, reaching for something or pushing something away to tell you what she likes and doesn’t like*.” – PI:211

##### Receptive

Caregivers had opportunities to talk about their child’s receptive communication skills throughout the concept elicitation portion of the interview. The most common place for caregivers to start this discussion was in the first question, “What kinds of things does your child typically communicate about?” with the follow up probe, “How does your child respond to your communication?”

Eight caregivers of children with STXBP1-related disorders described different receptive communication concepts, including making choices (*n=*6), following familiar directions (*n=*3), understanding isolated words or phrases (*n=*1), responding to their name (*n=*1), and responding to simple questions (*n=*1). Example quotes included:

- Following routine directions:
  One caregiver (PI:202) described how her child can follow directions that are part of her routine: “*[Child’s name], come in here.” She won’t do anything like that. But there’s something about the food and the context of that that she will come in for. There are a few things. If I’m changing her clothes, and I say, “[Child’s name], raise up your arms,” she’ll do that. And putting her shoes and socks on, and I say, “Give me your leg.” She’d do that. But again, I don’t know if it’s language or if it’s just the habit, the routine.”*
- Understanding isolated words:

*“She does know some body parts. And like before, she couldn't respond. She couldn't point to any of those things. She couldn't show that she knew any of the body parts. Now she's able to point to certain body parts. And now like, for example, she could point to her ear when I say “ear”. But the cool or funny thing is, if we say a word that sounds like, “ear,” like, say I was in a conversation with a different family member, and I say, “Oh, last year we did such a such,” and I say the word, “year,” she'll just quickly point to her ear because she's like, “It sounds like ear.” So, she's showing she's actually listening to the conversations around her.” –* PI:212

- Responding to yes/no questions:
  *“So we kind of ask a question and then pause, ask a question again, pause, and giver her time to kind of register what's being asked. But recently, she's been nodding her head with, like, a slow up and down motion. So, we take that as yes is her answer…So, normally, the questions we would ask is: Did you have a good day in school? Did you eat? How was your day? Did you get in trouble? Those type of questions. And she'll just nod her head up and down. So, not every time, but I guess when she feels like answering.”* – PI:206

The most common receptive communication concept mentioned by half of the caregivers was **making choices**. Caregivers reported that their children made choices about what they want to eat (*n=*3), what toy they want to play with (*n=*3), which shows or movies they want to watch (*n=*4), and what activities they wanted to do (*n=*3). Three caregivers described how their child made choices by reaching for a specific item from a group, with one caregiver (PI:202) explaining, *“She’s really trying to be clear about what she wants. It’s not that she’s just hungry, but it’s that she wants this trail mix. There’s a certain thing. She goes into a full pantry, and she’ll rifle past the vegetables and the beans and the pasta, and she knows exactly what it is that she’s grabbing. What she’s grabbing is what she wants to eat, and it’s not just an indication of, “I have food. I’m hungry.”*

Three caregivers reported that their child uses an AAC device (high or low tech) to make a choice about an activity. For example, one caregiver (PI:203) said, “*So, we do try to help him with the pointing as well. Sometimes, though, the pointing is pretty right on. I think it just depends on him on that day. I think it also depends on his motivation to point for something. But usually, it’s a picture board or they will show him picture icons like two or three picture icons. They’ll hold them up for him and ask him which one he wants or what he wants to do. Then, he’ll look at the one he wants or wants to do. Then, like I said, we’ll help to guide him to point to it*.” Two caregivers (PI:203 & PI:206) discussed how their children would select a choice after being given a list of options. One caregiver (PI:205) described that their child would request a specific item they want by name, saying, *“So, I kind of ask her, "What do you want to look at?" And we'll go through – she likes the same shows. So, we'll go through a series. So, it's between Sister Act, Blippi, Yo Gabba Gabba!, or she likes DJ Cassidy because she loves music. And so, I will pretty much go through the list until she gives me some kind of joy or smile or clap, and I'm like, okay, that's what you want to look at.”*

##### Pragmatic

Seven caregivers of children with STXBP1-related disorders mentioned different pragmatic/social communication concepts during concept elicitation. Examples given by caregivers included greetings (e.g. “hi” or “bye”), using names (e.g. people or places), and playing games. The modalities that caregivers reported individuals using for pragmatic communication included words/word approximations (*n=*6), gestures/signs (*n=*1), and AAC devices (*n=*1). Note, some children could use multiple modalities.

Two caregivers described generally how their children desire to connect socially with those around them. Both discussed how the child makes eye contact with those around them to show they want to engage. Relatedly, a third caregiver noted how, over time, her child has become more aware of how a communication partner fits within the exchange of information, or cause and effect. She stated that her child now has knowledge of, “*expectations that you need to understand in order to interact with your communication partner. So, the expectations are, okay, well, I have to do this so that she understands this.*”

Many caregivers said their child used verbal speech (e.g., words/word approximations), AAC devices, or even signs to name someone; mostly, parents, siblings, important figures (e.g. television characters), family members (e.g. grandparents), or other caregivers (e.g. nanny). Quotes of children using names included:

- “*She’ll say, “I want car,” “I want Papa,” “I want Grandma.” She’ll say, Mama,” and those are her three main phrases.”* – PI:205
- *“…She’ll say bru bru for brother”* – PI:206
- *“She can kind of do a ‘da-da’ that we think is basically relating to either parent.”* – PI:211
- “*He right now is very much into Blaze and the Monster Machines, which is a cartoon. And he gets to know their name. And he knows some names from the Paw Patrol as well. But he’ll communicate – if he sees the one on TV or he has the specific one toy in his hand, he’ll say their proper name, obviously to the best of his ability*.” – PI:208
- “*Oh, she did learn for Thomas the Train, she’ll say “Tom.” “Tom, Mia,” something close to it*.” – PI:212
- “*She most frequently will use the “I want” button when she’s going in or the people button. If she uses the people button, there are pictures of people that are basically just the people I just named, her parents, grandparents, sibling, and nanny. So, she can ask for people. She does do that. On occasion, she will go there and choose a person*.” – PI:210
- “*She created her own sign for “baby gorilla,” and then she created some, like made up her own sign for three of her grandparents. So, they each have a different gesture for the grandparents.”* – PI:212

#### Changes in Communication

All 12 caregivers whose children had STXBP1-related disorders discussed how their child’s communication changed overtime. During those discussions, some of the caregivers discussed when the changes occurred, whether the changes were fast or slow, and what happened to communication skills once they were learned.

Caregivers described different changes they have seen in their child’s communication abilities, including both improvements and regressions. Caregivers described seeing improvements in their child’s receptive language (*n=*3), physical gestures/mobility (*n=*5), and vocabulary (*n=*4). See Table S74 for specific examples. Eight caregivers said they saw the improvements happen between the ages of 3-6.

Table S74: Examples of improvements in communication ability.

| Participant ID | Quote |
| --- | --- |
| 202 | *There’s still a lot of guessing, but in some of those situations, like I mentioned especially with food or drink or wanting to move throughout the house if she’s in a chair, we at least know what she wants. And for a long time, she would just sit there and cry.* |
| 203 | *But sometimes he’ll grab my hand and take me to his playroom or something like that if he wants me to go with him. So, these kinds of things have gotten better over the years. Just as the course of his mobility has gotten better, his physical communication skills have gotten better.* |
| 204 | *So, I guess his ability to mobilize himself better, would be one area that he’s improved.* |
| 206 | *So, I'm noticing as she's getting older, she's picking up a little more. Definitely, on understanding and processing.*  *As far as communicating, she verbalizes a little more in different ways, whether it's hand gestures or eye gazing or reaching or now she'll – I forgot to mention. She'll walk up to you and kind of like grab your hand to show you what she wants. Like, come with me and this is what I want you to do.* |
| 207 | *He’s improving by using words more.* |
| 208 | *I mean, he’s definitely a lot more vocal and verbal now than he was. You know. He’s definitely taken on a little bit more broad number of words that he uses now. So, I definitely think that has grown as far as the vocabulary that he does have.* |
| 210 | *She’s definitely gotten more expressive in her gestures and her facial expressions…*  *So, she’s gotten much better at choosing between two items. Even over the short period of time we’d use the AAC, we’ve seen significant improvement with her ability to leverage that as well and choose between multiple items and even say a short sentence.* |
| 211 | *While I mentioned she doesn’t have words, she can kind of do a ‘da-da’ that we think is basically relating to either parent. And she can do like an ‘eeee’ which we think is kind of associated with eating. And those are more recent developments so I guess we would call those word approximations. And those have developed in the last year. Her gestures and signs are also something that has developed more over the last year and a half.* |
| 212 | *But the cool or funny thing is, if we say a word that sounds like, “ear,” like, say I was in a conversation with a different family member, and I say, “Oh, last year we did such and such,” and I say the word, year,” she'll just quickly point to her ear because she's like, “It sounds like ear.” So, she's showing she's actually listening to the conversations around her... That's a big development that a couple years ago, she didn't do.* |

A few caregivers (*n=*3) also described **regressions** in their child’s communication. For one child, the regression happened between ages 11-12. Examples of quotes describing regressions/loss in skills included:

- “*I would say sounds and words and approximations of words were things that we’ve seen over time that kind of rotated in and out. But some have completely left including all of his actual words have completely left.”* – PI:203
- “*Overall, I feel like she's made more gains than regression. But there are a few things where she used to say a word but no longer says it. So, she's lost a couple words, but overall, the bigger picture, I think she's just kind of made small gains over the years*.” – PI:212

Seven caregivers discussed the **speed of changes** in their child’s communication abilities and all seven said changes happen *slowly* (Table S75).

Table S75: Caregiver perceptions on the speed that changes in communication occur.

| Participant ID | Quote |
| --- | --- |
| 201 | *He’s improving slowly.* |
| 202 | *I think it’s a slow change. I would say pretty much everything with her is a very slow change.* |
| 206 | *No, actually, it's like a slow change. So, a little bit at a time. Not quick at all.* |
| 207 | *I would say, I guess, slow. I don’t know. Sometimes they’re fast, and sometimes they’re slow. Like, the “help” was pretty quick. And “bye-bye,” he starts saying it quick, but from the point we start teaching. Because, you know, we’ve been saying “bye-bye” since he was a baby and he just started saying it. So, slow.* |
| 209 | *Slowly. I would say within the six months; it’s gotten better.* *It’s just a slow learning process, really.* |
| 211 | *Well, still slow compared to a normally developing child, absolutely.* |
| 212 | *Well, we're finding for many, many, many years, like, we would just repeat, and repeat, and repeat. And she may or may not grasp the new thing or echo it back. But I think I feel like she's finally – like, she just turned 13…so I feel like certain things are starting to come together for her. Like, after all this repetition, I know, she's slowly adding some new words and some new signs.* |

Like caregivers of individuals with SYNGAP1-related intellectual disability, some caregivers of individuals with STXBP1-related disorders also mentioned changes occurring after adjusting medications (*n=*2) and with changes in seizure frequency/control (*n=*1). Some example quotes include:

- “*Really, to be honest, when she started with her new ADHD medicine, Dyanavel… I noticed that it kind of slows her thought process down and she's able to understand, grasp, and pay attention. So, since then, she has had time to kind of just pick up on things instead of without the proper medicine for her ADHD, kind of all over the place. So, it wasn't like, she couldn’t just sit there and focus and be like, "Okay, I understand what you're saying." It was like boom, boom, boom, boom, boom all over the place. Whereas now, it's kind of like the medicine kicks in and she's focused*.” – PI:206
- “*And we got her seizures under control when she was about one year of age and that really opened the door for her to engage with the world around her and communicate with the world around her.”* – PI:211

Many caregivers (*n=*8) mentioned learning a new skill requires repetition and modeling. Some illustrative quotes included:

- “*I just know that for a child living with STX they need lots of repetition to learn, and they need to, I don't know, just be given a chance and be able to try something. They need to just try things over and over and over again to possibly get it*.” – PI:212
- “*I will say his pointing has gotten better. He now knows how to point, whereas that took a long time. It wasn’t until he was home with me during COVID and I was really working with him on the pointing that he really started to get the hang of, oh, that’s what you’re wanting me to do*.” – PI:203

A few caregivers (*n=*3) mentioned that certain skills may be learned more quickly depending on the child’s motivation. For example, one caregiver (PI:212) said, “*Another thing, for her to learn, like I said, for her to want to say a certain word, it has to be within one of her special interest or has to be something really profound*.”

Caregivers shared mixed opinions about skills disappearing over time or certain skills being lost as new skills were developed. While six caregivers mentioned inconsistency across skill gains vs loss (Table S76), four caregivers said they do **not** think any of their child’s skills disappear when they are learning a new communication skill.

Table S76: Examples of communication being variable and inconsistent.

| Participant ID | Quote |
| --- | --- |
| 201 | *Sometimes they [communication skills] stay. Sometimes they disappear.* |
| 203 | *When he was younger, I always felt like he only had a capacity for so many words. It was like once he lost one, then maybe he might gain another one. But you wouldn’t see that other one – you either wouldn’t see it again at all, or maybe you might hear it later. This is the same thing with sounds too. I would say sounds and words and approximations of words were things that we’ve seen over time that kind of rotated in and out.* |
| 204 | *He’s learned how to use a pointer finger. But it’s just a skill that doesn’t stick with him. So, it kind of comes and goes* |
| 205 | *There definitely have been things that have come and gone, like phrases maybe I don’t hear anymore.* |
| 208 | *Because sometimes he’ll have words but then you don’t hear them. He used to say his name and now he doesn’t.* |
| 211 | *There is some ebb and flow to that that is frustrating as a parent in terms of she may have a gesture that she seems to be using well and then you know, for example, signing more when she wants something. And then seemingly losing that or at least using it less.* |

#### Hearing & Vision Impacts

None of the 12 caregivers indicated that their child had a hearing impairment. However, three caregivers reported that their child had a confirmed vision impairment, and one additional caregiver reported that they suspected their child had cortical vision impairment (CVI), but had not been diagnosed yet.

Two caregivers (PI:208 and PI:212) said their child was diagnosed with mild CVI. Neither was sure that this impacted their child’s communication; however, one caregiver believed it impacted their child’s depth perception and ability to walk. For example, they said, “*It [CVI] changes other things like walking safely because she has poor depth perception. But it's like I don't really know if it impacts her communication*.” The third caregiver (PI:206) said their child was farsighted and needed to see things at certain angles. For example, they said, “*I can't really say if I know how it affects her communication. I know that seeing up close, it has to be at a certain angle for her to see. So, if I'm talking to her and I have something in front of her and it's down, she really doesn’t hold her head down and look. Everything's like right up* *front, like straight*.”

#### Meaningful Change

All caregivers during the interviews were asked, “*What would a meaningful change in communication look like for your child?*” If time was available, most caregivers were asked “*If your child’s communication skills were to decline or get worse, which communication skills would be most important for your child to retain*?”

Parents interpreted “meaningful change” to mean “improvements” in communication ability or skills. During this question, some parents made the distinction between realistic and unrealistic goals they had for their children.

Caregivers of children with STXBP1-related disorders reported several examples of meaningful improvement, with some caregivers reporting more than one (Table S77). Caregivers discussed children acquiring different modalities (e.g. pointing, sounds, speech), and functional skills (e.g. expressing emotions). Three caregivers in this group also mentioned that it would be meaningful for their child to advance in communication so that other people, less familiar with the child, could understand them.

Table S77. Responses to “*What would a meaningful change in communication look like for your child?*” from 12 caregivers of children with STXBP1-related disorders.

| Meaningful Improvement | 201 | 202 | 203 | 204 | 205 | 206 | 207 | 208 | 209 | 210 | 211 | 212 | Total |
| --- | --- | --- | --- | --- | --- | --- | --- | --- | --- | --- | --- | --- | --- |
| Start using verbal words | 1 |  | 1 |  |  |  |  |  |  | 1 | 1 |  | 4 |
| Better communication to reduce frustration/behavior |  |  |  |  |  |  |  | 1 |  |  |  |  | 1 |
| Start using complex sentences - any modality (e.g., AAC, verbal words, etc.) |  |  |  |  | 1 |  | 1 |  | 1 | 1 |  |  | 4 |
| Communicate if need help/ in danger |  |  |  |  |  |  | 1 |  |  |  |  |  | 1 |
| Express emotions, opinions - any modality |  |  |  |  | 1 |  |  | 1 |  |  |  |  | 2 |
| Express Feelings/Emotions AND pain |  | 1 |  |  |  |  |  |  |  |  |  |  | 1 |
| Consistent communication |  |  |  |  |  |  |  |  | 1 |  | 1 |  | 2 |
| Start pointing |  |  | 1 |  |  |  |  |  |  |  |  |  | 1 |
| Make choices |  |  |  | 1 |  |  |  |  |  |  |  |  | 1 |
| Communication advance so people not familiar with the child can understand (family has spent a lot of time figuring out what things mean) |  |  |  |  | 1 |  | 1 |  |  | 1 |  |  | 3 |
| Start using High Tech AAC |  |  |  |  |  |  |  |  |  |  |  | 1 | 1 |

Discussing meaningful change related to worsening of communication ability was challenging for some caregivers to conceptualize or answer. In this group, caregivers mentioned different modalities including eye gaze, gestures, verbal speech, and AAC device usage as skills that would be important for their child to retain (Table S78).

One parent (PI:202) described why there is overlap in wanting the child to describe pain and emotions when children are unable to communicate what they are feeling: “*We have days where she cries. If there’s a string of three or four days, it’s like, “She’s really crying more than normal” or her teacher’s like, “She’s fussier. More than normal,” it’s not anything that we can pinpoint. All we can do is take her to the doctor and have them run a battery of tests and bloodwork to try to find something. Or is it her teeth? Do we need to go to the dentist? It’s really a challenging situation, whereas if she could just come up and say, “I have a headache,” then it’s like, “Okay.” If she can point to her head, and some sort of sign for pain, then it’s like, “Okay, Tylenol. We don’t need to go to the dentist*.”

Table S78. Responses to “*If your child’s communication skills were to decline or get worse, which communication skills would be most important for your child to retain*?”

| Worsening communication – Skills important to retain | 201 | 202 | 203 | 204 | 205 | 206 | 207 | 208 | 209 | 210 | 211 | 212 | Total |
| --- | --- | --- | --- | --- | --- | --- | --- | --- | --- | --- | --- | --- | --- |
| Gesture |  |  |  |  |  |  |  |  | 1 | 1 |  |  | 2 |
| Caregiver unable to this answer/conceptualize this question |  |  |  |  |  | 1 |  |  |  |  |  |  | 1 |
| Verbal words |  |  |  |  |  |  | 1 |  |  |  |  |  | 1 |
| AAC device usage |  |  |  |  |  |  |  | 1 |  | 1 |  |  | 2 |
| Express needs - any modality | 1 |  |  |  | 1 |  |  |  |  |  |  |  | 2 |
| Express hunger or thirst |  | 1 |  |  |  |  |  |  |  |  |  |  | 1 |
| Eye gaze |  |  | 1 |  |  |  |  |  |  |  |  |  | 1 |
| Going to the thing/taking person to item |  |  | 1 |  |  |  |  |  |  |  |  |  | 1 |
| Child remains happy |  |  |  |  |  |  |  |  |  |  | 1 |  | 1 |

*204, 212 were not asked this question due to time constraints

### 12. SYNGAP1-related intellectual disability

#### Demographics

Twelve caregivers of individuals with SYNGAP1-related intellectual disability participated. The majority of caregivers were female and on average 39.3 years of age (Table S79). One-third of caregivers (4/12) self-identified as Hispanic-Latino. Nine caregivers identified as white, 2 as African American/Black, and 1 as Asian. Most caregivers (10/12) reported that they were currently married or living with a domestic partner, with 2 caregivers indicating they were divorced. Overall, the sample reported high levels of education, employment (8/12 employed at least part-time), and annual income (Table S79).

Table S79. Demographic information for 12 caregivers of individuals with SYNGAP1 who participated in the qualitative interview study.

| *Caregivers* | *n (%)* |
| --- | --- |
| Female | 10(83.3) |
| Age, years (Mean/SD) | 39.3/6.5 |
| Ethnicity |  |
| Not Hispanic or Latino | 8(66.7) |
| Hispanic-Latino | 4(33.3) |
| Race |  |
| White | 9(75.0) |
| African-American or Black | 2(16.7) |
| Asian | 1(8.3) |
| Middle Eastern | 0 |
| More than one race | 0 |
| Relationship status |  |
| Single, never married | 0 |
| Married, or living with domestic partner | 10(83.3) |
| Separated | 0 |
| Divorced | 2(16.7) |
| Widowed | 0 |
| Highest grade in school |  |
| Less than high school diploma | 0 |
| High school degree or equivalent | 2(16.7) |
| Some college/University | 2(16.7) |
| College/University degree | 3(25.0) |
| Postgraduate degree | 5(41.7) |
| Occupational status |  |
| Homemaker | 3(25.0) |
| Unemployed | 1(8.3) |
| Retired | 0 |
| On disability | 0 |
| On leave of absence | 0 |
| Full-time employed | 6(50.0) |
| Part-time employed | 2(16.7) |
| Full-time student only | 0 |
| Income of U.S. residents |  |
| Less than $20,000 | 0 |
| Between $20,001 and $40,000 | 0 |
| Between $40,001 and $60,000 | 2(16.7) |
| Between $60,001 and $80,000 | 3(25.0) |
| Between $80,001 and $100,000 | 1(8.3) |
| Between $100,001 and $250,000 | 4(33.3) |
| Between $250,001 and $500,000 | 2(16.7) |
| $500,000+ | 0 |
| I prefer not to answer | 0 |
| I do not know | 0 |
| Relation to child |  |
| Mother/Step-mother | 10(83.3) |
| Father/Step-father | 2(16.7) |

Caregivers also provided demographic information for their child with SYNGAP1. Five children were male, with similar rates to caregivers in regards to ethnicity and race (Table S80). Mutation types were classified via participation in the Ciitizen registry, which required submission of genetic reports. Within Ciitizen, the variants are classified using the process recommended by the American College of Medical Genetis and Genomics^81^ and include variant interpretation by a trained individual at the clinical lab that conducted the sequence testing (e.g. GeneDx, Invitae, etc). To further confirm mutation type, the c. and p. addresses included within the child’s genetic report were recorded by the Duke study team during the screening process. Mutation types included: 6 frameshift, 3 nonsense, and 1 intronic, missense, and multigenic deletion.

Eleven caregivers reported their children also had an Autism Spectrum Disorder (ASD) diagnosis, and all 12 reported that their children had epilepsy/seizures. The majority of children were receiving therapeutic services, including physical, occupational, and speech therapy (Table S80). The majority of caregivers (9/12) reported their child used an Augmentative and Alternative Communication (AAC) device, and all of those parents reported the device was considered ‘high tech’ (e.g. iPad). The reported settings in which the device was used varied (Table S80). The mean age the device was introduced to the child was 4.4 years old (*SD* = 1.3).

When asked about their child’s current verbal language, no caregivers indicated that their child used ‘full sentences’, 2 caregivers indicated that their child used ‘short phrases’, 4 indicated their child used a ‘few words’, and 6 indicated their child used ‘no words’.

Table S80. Demographic information for children with SYNGAP1-related intellectual disability.

| *Children* | *n(%)* |
| --- | --- |
| Age, years (Mean/SD) | 7.8/4.9 |
| Child gender Female | 7(58.3) |
| Ethnicity |  |
| Not Hispanic or Latino | 7(58.3) |
| Hispanic-Latino | 5(41.7) |
| Race |  |
| White | 7(58.3) |
| African-American or Black | 2(16.7) |
| American Indian/Alaska Native | 0 |
| Asian | 1(8.3) |
| Middle Eastern | 0 |
| Native Hawaiian/Other Pacific Islander | 1(8.3) |
| More than one race* | 1(8.3) |
| Child’s genotype |  |
| Frameshift | 6(50.0) |
| Intronic | 1(8.3) |
| Missense | 1(8.3) |
| Multigenic deletion | 1(8.3) |
| Nonsense | 3(25.0) |
| Autism Spectrum Disorder (ASD) | 11(91.7) |
| Epilepsy | 12(100.0) |
| Types of therapy |  |
| Physical Therapy | 8(66.7) |
| Occupational Therapy | 10(83.3) |
| Speech Therapy | 10(83.3) |
| Other therapy  ABA Therapy  Music Therapy  Sensory Therapy  Vision Therapy | 7(58.3)  1(8.3)  1(8.3)  1(8.3) |
| Age start speech therapy (Mean/SD) | 2.0/1.2 |
| Age first introduced to device (Mean/SD) | 4.4/1.3 |
| AAC device |  |
| No | 3(25.0) |
| Yes | 9(75.0) |
| Device |  |
| High tech | 9(75.0) |
| Low tech | 0 |
| Places to use the device |  |
| Home | 7(58.3) |
| School | 8(66.7) |
| Out in the community | 4(33.3) |
| Other  Therapy | 2(16.7) |

Note: *White and African-American or Black

#### Typical Communication Ability

The results for this section are organized by behaviors that correspond to expressive, receptive, and pragmatic (i.e. social) communication, aligning with the conceptual framework that was developed for individuals with Angelman syndrome.^1^ Please note, although counts are reported to indicate salience of each theme, these data are limited in that they do not indicate *all* communication behaviors relevant to each child, only the ones that caregivers discussed during the interview.

All 12 caregivers of children with SYNGAP1-related intellectual disability spoke about their child’s typical communication. All caregivers spoke about expressive communication behaviors they observed in their child, and most caregivers also mentioned pragmatic (social) communication behaviors (Table S81).

Table S81. Frequency of caregivers that mentioned one or more examples of expressive, receptive, and/or pragmatic communication during concept elicitation.

| Communication Behaviors | *n* (total *n=*12*)* |
| --- | --- |
| Expressive | 12 |
| Receptive | 6 |
| Pragmatic (Social) | 8 |

##### Expressive Communication

All 12 caregivers of children with SYNGAP1-related intellectual disability discussed different types of expressive communication concepts (Table S82). The most common concept mentioned by caregivers was requesting.

Table S82. Expressive communication concepts on the ORCA measure mentioned by caregivers of children with SYNGAP1-related intellectual disability.

| Expressive Communication Function | *n (total n=12)* |
| --- | --- |
| Seeking Attention | 5 |
| Directing Attention | 1 |
| Refusing an Object | 3 |
| Requesting an Object | 12 |
| Requesting an Object out of View | 4 |
| Requesting More | 1 |
| Telling Stories | 1 |

For **requesting**, the communication modalities mentioned by caregivers varied and included symbolic and non-symbolic communication behaviors. Symbolic modalities included words/word approximations (*n=*6), gestures/signs (e.g. finger point, American Sign Language, or modified gesture/sign; *n=*7), AAC device (*n=*3); and sounds (e.g. ‘ah’; *n=*1). Younger children tended to rely on non-symbolic forms of communication to communicate requests (e.g. bringing the caregiver to something they want). Caregivers in our sample said their child is better at requesting things that they like or are motivated by, and that their child is most successful in their communication when they’re making requests. Some caregiver-reported examples of requesting included:

- *“She will say uh for up, oh for open, or sometimes she will point to the TV and say E for TV*.” – PI:104
- “*You know. Sometimes like I said, he’ll rub his hands for carwash. But he’s generalizing that to water*.” – PI:103
- “*Normally, she will just point out what she wants, or she will open the fridge and she will just point out. Sometimes, she will make a gesture of eating or drinking…or if she wants juice, she will just do like a sippy cup [gesture]*.” – PI:106
- “*He has a talker – a speech device. He always says he wants to watch ceiling fans…he usually has to say “I,” and then “want,” and then “to watch,” and then he goes to the watch page, “ceiling fans.”* – PI:109
- “*So, if she wants us to open a toy for her or get it to spin or light up and she can't do it, she'll take our hand and put it on the toy. Or if she wants something to eat, she'll guide us to where the item is, or she'll go stand by her chair to indicate that she's hungry, or she'll bring it to us so we can open it for her*.” – PI:101
- “*For instance, if he has a sippy cup that’s on the counter he’ll just grab our hand and kinda thrust it towards the cup. And we know that that’s what he wants. Or if he wants to go outside he’ll pull us, pull us, pull us to the door and we know that he wants to go outside.”* – PI:110

Five caregivers mentioned that their child will **seek attention**. One of those 5 caregivers did not describe *how* the child will seek attention, only that they typically do. For the other four caregivers, examples of seeking attention included:

- “*He has more eye contact with us, now. We’ll see that he’s trying to get our attention because he’ll just look at us and he didn’t do that before*.” – PI:110
- “*She will make a grunting noise if she wants me to look at her. She’ll say my name. She’ll say mom*.” – PI:105
- “*He does a lot of tapping to get your attention*.” – PI:109
- “*So, initiation sometimes will look like him just coming up and grabbing your hand, and looking at you to let you know that he's trying to communicate.”* – PI:112

One caregiver (PI:102) spoke about how her child would label objects, actions, or people in her environment around her using words (called ‘tacting’), and this labeling helped to **direct the caregiver’s attention**. This caregiver mentioned that the child’s therapist was working on this skill specifically.

Three caregivers mentioned that their children were able to **refuse** something or indicate ‘no’. One child used words (e.g. ‘no’), one used gestures (meaning ‘all done’ or ‘stop’), and one caregiver did not detail which modality the child used to refuse.

Only one caregiver (PI:102) mentioned that their child liked to “*list out recent exciting things that have happened to her*” using words, which seemed analogous to the concept ‘**telling stories’** that was also infrequently seen in the Angelman syndrome sample.^1^

##### Receptive Communication

Caregivers had opportunities to talk about their child’s receptive communication skills throughout the concept elicitation portion of the interview. The most common place for caregivers to start this discussion was in the first question, “What kinds of things does your child typically communicate about?” with the follow up probe, “How does your child respond to your communication?”

Six caregivers of children with SYNGAP1-related intellectual disability described different receptive communication concepts, including following familiar directions (*n=*3), understanding isolated words or phrases (*n=*4), and making choices (*n=*1). One caregiver (PI:104) described how her child will follow familiar directions when she is motivated, “*If you ask her, ‘[Child’s name], go get Mommy’s shoes for me’, that type of thing. And then she’ll be like um, no, like I don't want to, like what is in it for me. But if you tell her that, ‘hey, [Child’s name], go get Mommy’s shoes and then I will let you watch cartoon show’ or something like that, then she will go.”* Another caregiver (PI:103) stated, *“We often say the walls are listening. So, I think he understands what’s going on in his environment. And if you’re talking on the side, I think he’s listening. He can hear the conversation. He understands what’s being said. So, I think we’ve observed if we’re talking and we’re talking about him, you can see maybe there’s a change in his deposition. Or he’ll do a sign. He’ll sign, “stop,” or whatever.”* Finally, PI:106 described how their child would choose food items by opening the fridge and pointing to a specific item or verbally naming the item. For example, they said, *“Normally, she will just point out what she wants, or she will open the fridge and she will just point out. Sometimes, she will make a gesture of eating or drinking, or she will just say a few words, like pan, like it’s bread, or – yes, that’s kinda like the one word that she – agua, water.”*

##### Pragmatic Communication.

Seven caregivers of children with SYNGAP1-related intellectual disability described the pragmatic/social communication their child engages in, with one additional caregiver discussing their impression that their child would like to communicate more socially. Examples given by caregivers included saying “bye”, apologizing appropriately (e.g. without prompting), using “please” when making requests, and using names (e.g. people or places). The modalities that caregivers reported individuals using for pragmatic communication included words/word approximations (*n=*4), gestures/signs (*n=*1), and AAC devices (*n=*2). One caregiver (PI:112) spoke about how she believed her child wanted to be social with those around him but was limited by his expressive communication abilities. The child seemed frustrated because “*he has really good receptive language*”, and thus, the caregiver believed he understood most of what was being said to him.

Two caregivers reported their children using names when communicating. One caregiver (PI:109) described how their child will name the people that the caregiver is talking about with their AAC device. For example, they said, “*I’ll be talking about my parents a lot, and [the child will use the symbol] “Grandma! Grandpa!”* Another caregiver (PI:102) said their child “*Likes to talk about the people that she knows and list out people”* using verbal speech (e.g., words or word approximations).

#### Changes in Communication

All 12 caregivers whose children had SYNGAP1-related intellectual disability discussed how their child’s communication changed overtime. During those discussions, some of the caregivers discussed when the changes occurred, whether the changes were fast or slow, and what happened to communication skills once they were learned.

Caregivers described different changes they have seen in their child’s communication abilities, including both improvements and regressions. In terms of improvements, caregivers described seeing improvements in their child’s pronunciation, receptive language, and vocabulary (e.g., an increase in the number of words, symbols, or gestures in a child’s repertoire or gaining the ability to string words/gestures/symbols together). Table S83 provides specific examples. Four caregivers said they saw the improvements happen between the ages of 4-6.

Table S83: Examples of caregiver-observed improvements in communication ability.

| Participant ID | Quote |
| --- | --- |
| 102 | *So, we've gone from single word or word approximation, really, that was only being able to be uttered in a single syllable, to now, she has quite a good number of two-word phrases.* |
| 104 | *Back in the day, her receptive language was like, we don’t know how much she understood, but now we know that she understood [the] majority of what we want.* |
| 105 | *And then, once she learned to point it was kind of like a floodgate opened and that was a good turning point for her.* *I guess the talker now, too because the talker was another turning point because we would play games like a chalk game where she’d hand me a piece of chalk and she’d want me to draw something. Circle and triangle sounded the same when she made the word approximation, but then when we got the talker, she could touch either the circle or the triangle button. Since the word approximation sounded the same, I had no idea, but then once we got the talker, I knew exactly what she wanted to say because she could just touch the button.* |
| 106 | *Well, she’s getting more words. It’s a slow process, but she’s learning to pronounce the words in a better way.* |
| 107 | *He improved a ton. He was being non-verbal completely, and now he [has] emerging speech.* |
| 108 | *It has improved. She actually has been able to speak – not full full sentences, but at least put somewhat sentences together… plus she’s aware of more words. I think right now, her vocabulary, it’s very vast, compared to what it was a few years ago.* |
| 109 | *It’s actually gotten a lot better, and he has more patience to listen to what we’re telling him, and he’s done a ton more, like if I’m talking to my other son or somebody, he hears it, and he’ll go to his talker and pick the words out that we said. Anywhere I’m talking to him, he’s just in the background, and he’s there, so he’s just repeating what we said, and he’s getting really, really good at that. He never did that when he was little.* |
| 110 | *He has more eye contact with us, now. We’ll see that he’s trying to get our attention because he’ll just look at us and he didn’t do that before. He’ll come over to us and get our attention that way. Then he’ll just pull us. So, I would say as he’s getting older he’s trying to do less crying and more – I won’t say gestures but more – just letting us know what he wants without getting fussy about it.* |

Six caregivers discussed the **speed of the changes** in their child’s communication ability and all six said changes in their child’s communication abilities happen *slowly* (Table S84).

Table S84: Caregiver perceptions on the speed that changes in communication occur.

| Participant ID | Quote |
| --- | --- |
| 101 | *It happens slow because I think she's resistant, she does things her own way. I think it does take a little bit of time.* |
| 102 | *Faster now than they used to be, but still slowly. When she gets something new, it takes her longer to master it.* |
| 105 | *She’s always learning more… Her pronunciation of words is continuing to get better. It’s super-slow. It’s really slow, but it’s happening, so it’s fun to watch.* |
| 106 | *It’s a slow process, but she’s learning to pronounce the words in better way. So, I would say that it’s improving slowly, but it’s improving.* |
| 107 | *So, I would say that he typically improves fairly fast in the summertime, but it’s definitely a gradual thing. It’s not like a light switch.* |
| 108 | *It’s not as fast as it was when we first removed the meds. It’s something like it’s gradual. But it’s still improvement.* |

Three caregivers (PI:104, PI:106, and PI:108) said the most changes in their child’s communication occurred when they adjusted one of their child’s medications (e.g., putting the child on a new medication or discontinuing a medication). Example quotes included:

- “*I think it was mainly when we removed one of her meds. And then, a few months later, she started to kind of be more vocal, and being able to verbalize a little more*.” – PI:108
- “*I think when she started her anti-seizure medication, which is at two years and two months, that was when we saw a switch in her receptive language. It seemed like nothing, nothing, nothing, and then we started the medication, and in a month, she started walking, and then she just started understanding*.” – PI:104

Four caregivers (PI:104, PI:101, PI:105, and PI:106) with children under the age of seven said seizures greatly impact their child’s communication. Example quotes included:

- “*She has severe GI issues, and we just found out that she has seizures. So, I think a lot of these issues, when they're severe, they prevent her from trying to communicate and learn. But when she's feeling better, that's when I notice that the learning improves, and she tries to communicate more*.” – PI:101
- “…*these kids have seizures on and off, and so whenever she’s having a period of time with a lot of seizures, sometimes other learning will slow during that time, or stop, or even regress a little bit. Then, she might catch back up to where she was before*.” – PI:105

Some caregivers (*n=*4) mentioned that learning a new skill requires repetition and modeling. For example, one caregiver (PI:105) said, “…*having to teach [child’s name] sign language, it was so different because it was just so many repetitions of just this one single sign. We’d work on one sign for weeks at a time, and it was just so slow going*.” A few caregivers mentioned that certain skills may be learned more quickly depending on the child’s motivation. For example, one caregiver ((PI:109) said, “*He’ll pick words out that he knows well, or if it’s something he really likes to talk about, I guess... I think I was talking with somebody one day about a building, and I’m like, “I hope they have an elevator in that building” or something, and he was like, “Elevator! Elevator!” because he loves elevators. So, I think it’s things that he likes... It’s never anything random*.”

Two caregivers also described **regressions** in their child’s communication. For one child, the regression happened between ages 7-8. That caregiver (PI:103) said, “*I don’t know that we’ve had an increase in vocabulary. In fact, I’d probably say we’ve had some regression in some words. At one point he understood what signs for boy and girl were. And he kinda lost those.”* The other caregiver (PI:112) said the regression happened around age 3. For example, they said, “*So, when he was younger he actually had a couple sounds. He was able to make the B sound, and he had some D sounds. As he's gotten older those sounds have actually kind of gone away, and he doesn't have those sounds anymore. So, I would say that part regressed a little bit.”* In contrast to regressions, which imply a temporary or permanent loss of skills, caregivers also mentioned the **variable nature of their child’s communication**. This included statements about how the trajectory might be different than that of typically developing children. Example quotes can be found in Table S85.

Table S85: Examples of communication being variable and inconsistent.

| Participant ID | Quote |
| --- | --- |
| 101 | *And then like I said there are periods where she's just using it [a new communication skill] often and then she has regressions where we don't see it at all for a while, and then it just pops up again.* |
| 104 | *Every single day is different. Some days, she has more babbling; some days, she has none. Some days, she has more. If you ask her – like she knows all her colors, but if you say, say orange, and then she will be like “or”, and if you say, say green, and she will say “E”. And some days, she will repeat after you. She will try; some days, she won’t.* |
| 105 | *Like all skills, it’s like two steps forward and one step back or whatever they say. So, sometimes we will have a short period of time where it’s like, I thought she was learning, and then it seems like maybe she didn’t get it, and then finally she’ll get it.* |
| 108 | *But there are days where she cannot use the, “This,” or, “Is.” But yet, she’s able to put sentences together…* |

Two caregivers described how other communication skills disappear when their child is trying to learn a new skill (see quotes below), while four caregivers said they do not think any of their child’s skills disappear when they are learning a new communication skill. One caregiver (PI:107) said they sometimes see temporary regressions in other skill domains (e.g., fine motor) when their child is trying to learn a new skill. For example, they said, “*Like, whenever he learned to walk, then other things took a back seat too*.” All of these caregivers had children that were younger on average (all but one under the age of 7). Quotes from the two caregivers that discussed skills disappearing included:

- “*Yeah, we do see the disappearing of certain learned sounds, that she like – what we thought had mastered certain sounds or certain combination of sounds. And then it goes away for a long time. And I think, from what I understand, that's like the nature of apraxia sometimes, where she's learned something new and she’s mastering that, but then now she's lost the ability to say something else, a different word that she was doing very well before*.” – PI:102
- “*Sometimes he'll retain skills even when he learns new skills, but sometimes those older skills. They'll disappear. He won't maintain them.”* – PI:112

#### Hearing & Vision Impacts

All 12 caregivers were asked whether their child had any hearing or vision issues that impact their ability to communicate. No caregivers of individuals with SYNGAP1-related intellectual disability reported that their child had a hearing impairment. Only one caregiver (PI:112) reported that their child had a confirmed vision impairment (CVI, astigmatism and wore glasses). This caregiver said these vision impairments, particularly CVI, does impact their child’s expressive and receptive communication, saying, “*So, he has like certain fields of vision that are good for him, and then other fields that aren’t good for him. So, if his ACC device – like if it doesn’t have what he wants right in his field of vision, he can't necessarily touch that word… If the ACC device and what he needs is not in that field of vision and he can't see it – he can't obviously touch the button.”*

#### Meaningful Change

All caregivers during the interviews were asked, “*What would a meaningful change in communication look like for your child?*” If time was available, most caregivers were asked “*If your child’s communication skills were to decline or get worse, which communication skills would be most important for your child to retain?*” Parents commonly interpreted “meaningful change” to mean “improvements” in communication ability or skills. In response to this question, some parents made the distinction of realistic and unrealistic goals they had for their children.

For caregivers of children with SYNGAP1-related intellectual disability, they provided several examples of meaningful improvements, with some caregivers reporting more than one (Table S86). Caregivers discussed children acquiring different modalities (e.g. pointing, sounds, speech), and also functional skills (e.g. expressing emotions).

Table S86. Responses to “*What would a meaningful change in communication look like for your child?*” from 12 caregivers of children with SYNGAP1-related intellectual disability.

| Meaningful Improvement | 101 | 102 | 103 | 104 | 105 | 106 | 107 | 108 | 109 | 110 | 111 | 112 | Total |
| --- | --- | --- | --- | --- | --- | --- | --- | --- | --- | --- | --- | --- | --- |
| More pointing | 1 |  |  |  |  |  |  |  |  |  |  |  | 1 |
| More sounds | 1 |  |  |  |  |  |  |  |  |  |  |  | 1 |
| Start using verbal words | 1 |  |  | 1 |  |  |  |  |  | 1 | 1 |  | 4 |
| Better communication to reduce frustration/behavior | 1 | 1 | 1 |  |  |  | 1 |  | 1 |  |  |  | 5 |
| Start using complex sentences – any modality (e.g., AAC, verbal words, etc.) |  | 1 |  | 1 |  | 1 | 1 |  | 1 |  |  |  | 5 |
| Communicate if need help/ in danger |  | 1 |  |  |  |  |  |  |  |  |  |  | 1 |
| Express emotions, opinions - any modality |  |  | 1 |  |  |  |  |  |  |  |  |  | 1 |
| Express Feelings/Emotions AND pain |  |  |  |  | 1 |  |  |  | 1 | 1 | 1 | 1 | 5 |
| Improve pronunciation so strangers can understand |  |  |  |  | 1 |  |  | 1 |  |  |  |  | 2 |
| More words |  |  |  |  |  | 1 |  |  |  |  |  |  | 1 |
| Consistent communication |  |  |  |  |  | 1 |  |  |  |  |  |  | 1 |
| Catch up with peers’ communication ability |  |  |  |  |  |  | 1 |  |  |  |  |  | 1 |
| Improved receptive communication |  |  |  |  |  |  |  |  | 1 |  |  |  | 1 |
| Stop perseverating |  |  |  |  |  |  |  |  | 1 |  |  |  | 1 |
| Communicate toileting needs |  |  |  |  |  |  |  |  |  | 1 |  |  | 1 |

Discussing if communication ability was to **worsen** for their child was a challenging concept for some caregivers to conceptualize or answer. Some parents tried to link the change to a specific mechanism that would cause the regressions. For example, one caregiver (PI:104) stated, “*I don’t know? Thinking about what are the scenarios, like she can’t use her finger, or that she can’t process her train of thoughts because of encephalopathy? So, that’s kind of a really broad question for me to think about scenarios because it’s different like if she hurt her hand, she can’t use the device. Or if she hurt something, then she can’t move, or if her brain is worsened, that would be like, what else can she do type of thing.*” For caregivers that provided an answer to this question, the skills that were important for their child to maintain included gesturing, pointing, AAC device usage, and general mobility (more examples in Table S87).

Table S87. Responses to “*If your child’s communication skills were to decline or get worse, which communication skills would be most important for your child to retain*?”

| Worsening communication – Skills important to retain | 101 | 102 | 103 | 104 | 105 | 106 | 107 | 108 | 109 | 110 | 111 | 112 | Total |
| --- | --- | --- | --- | --- | --- | --- | --- | --- | --- | --- | --- | --- | --- |
| Gestures | 1 |  |  |  |  |  |  |  |  |  |  |  | 1 |
| Bring things to caregiver | 1 |  |  |  |  |  |  |  |  |  |  |  | 1 |
| Caregiver unable to answer/conceptualize this question |  |  | 1 | 1 |  |  |  |  |  |  |  |  | 2 |
| Receptive communication ability |  |  |  | 1 |  |  |  | 1 |  |  |  |  | 2 |
| Pointing (specifically) |  |  |  |  | 1 |  |  |  |  |  |  |  | 1 |
| Verbal words |  |  |  |  |  | 1 | 1 | 1 |  |  |  |  | 3 |
| AAC device usage |  |  |  |  |  |  |  |  | 1 |  |  | 1 | 2 |
| Mobility |  |  |  |  |  |  |  |  |  | 1 |  |  | 1 |

*102, 111 were not asked this question due to time constraints.

###

### Figure. Frequency of parents discussing concepts/themes by NND.


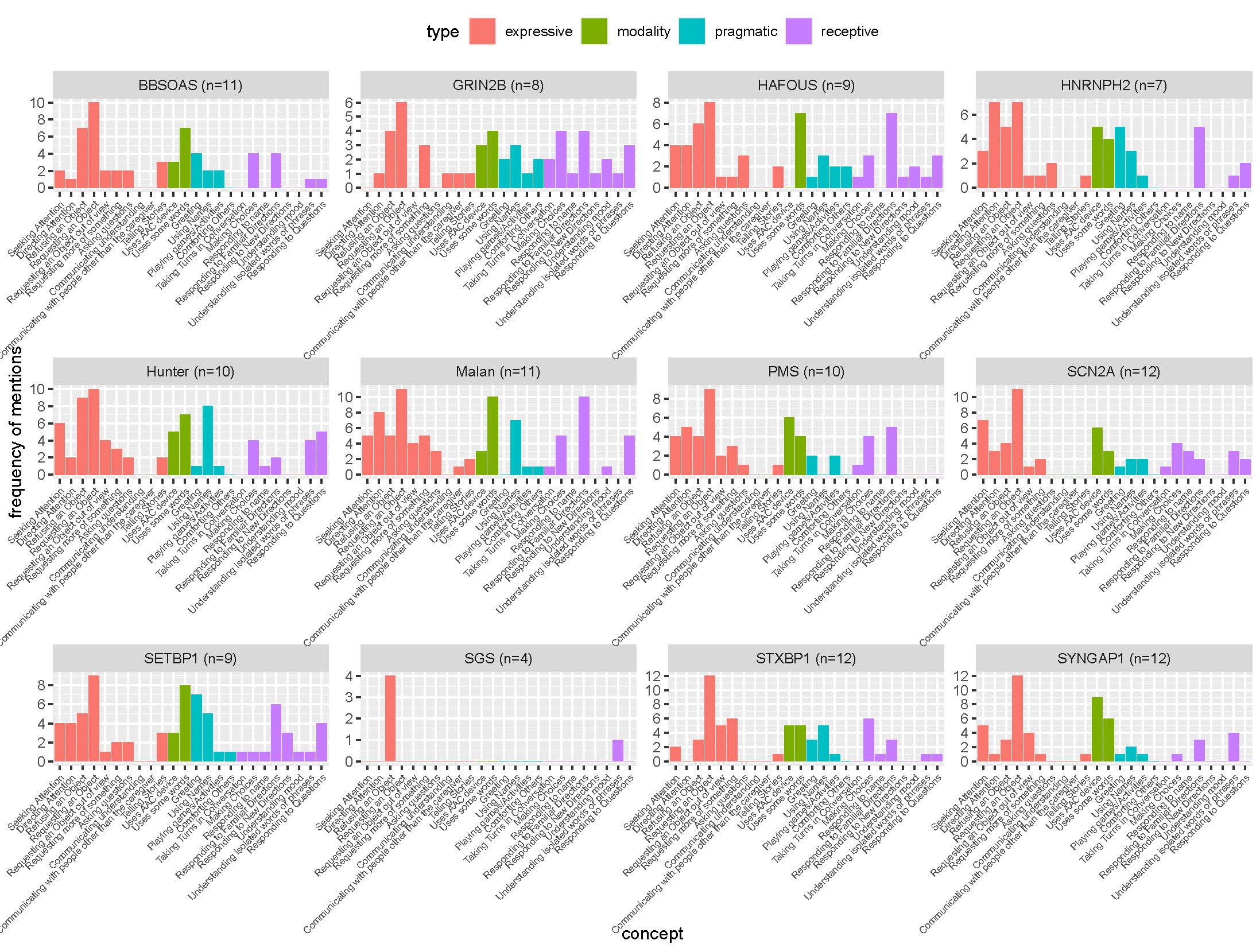


### Clinical Experts

#### Demographics

Nine clinical experts participated. The mean age was 47.4 years (range: 33-75 years). Demographic information for clinicians can be found in Table S88.

Table S88. Demographic information for nine clinical experts.

| Variable | *n(%)* |
| --- | --- |
| Gender |  |
| Male | 4 (44) |
| Female | 5 (56) |
| Ethnicity |  |
| Hispanic-Latino | 2 (22) |
| Race |  |
| White | 9 (100) |
| Education – Highest Degree Obtained |  |
| Masters | 4 (44) |
| PhD | 2 (22) |
| MD | 3 (33) |
| Expert in Augmentative & Alternative Communication |  |
| Yes | 5 (56) |
| No | 4 (44) |
| Clinical Trial Experience |  |
| Yes | 7 (78) |
| No | 2 (22) |
| Professional Title |  |
| Speech Language Pathologist | 4 (44) |
| Professor of Pediatrics | 1 (11) |
| Pediatric Neuropsychologist | 1 (11) |
| Pediatric Neurologist | 1 (11) |
| Special Education Teacher | 1 (11) |
| Child and Adolescent Psychiatrist | 1 (11) |
| Years in the field *mean (range)* | 18.4 (5-50) |
| # of individuals with NDDs cared for in 1 year *mean (range)* | 194.1 (7-700) |
| Most common types of NDDs worked with* |  |
| Angelman syndrome | 3 (33) |
| Autism Spectrum Disorder | 7 (78) |
| CDKL5 disorder | 3 (33) |
| Cerebral palsy | 2 (22) |
| Down syndrome | 2 (22) |
| *GRIN-related neurodevelopmental disorder* | 3 (33) |
| Intellectual disability | 2 (22) |
| *Phelan McDermid syndrome* | 4 (44) |
| Rett syndrome | 4 (44) |
| *SCN2A-related disorders* | 3 (33) |
| *STXBP1-relatd disorders* | 4 (44) |
| *SYNGAP1-related intellectual disability* | 5 (56) |
| *Malan syndrome* | 1 (11) |
| Current Practice Location (State) |  |
| CO | 1 (11) |
| FL | 1 (11) |
| MA | 1 (11) |
| MD | 1 (11) |
| NC | 3 (33) |
| PA | 1 (11) |
| TX | 1 (11) |

*Experts were asked specifically about NDDs involved in this study (in italics), but were also encouraged to disclose other NDDs they’ve worked with in the past. One clinician each mentioned: ADNP, ASXL3, CDLS, MERRF, IQSEC2, SHANK, FOXG1, Batten Disease, Epilepsy, CHARGE syndrome, Fragile X, DiGeorge syndrome, Wilson Disease.

#### Communication Ability: Definition & Components

When defining communication ability, five out of the nine communication experts referenced all three forms of communication (expressive, receptive and pragmatic forms) currently captured by the ORCA measure. The other four experts mentioned only expressive communication or described communication modalities. Many described communication ability as the ability to express wants, needs and feelings, transfer information, and engage in social closeness or exchanges. For example, one expert (PI:008) said, “…*when I think about evaluating a child’s communication, kind of three broader areas, receptive language, what we think that they are understanding, expressively what they’re doing to communicate with others, and then also a pragmatic aspect of their engagement with others*.”

Every communication expert also described different communication modalities while answering this question. The different communication modalities mentioned included verbal language (words, sounds, or approximations) and nonverbal language (gestures, signs, Augmentative and Alternative Communication devices, eye gaze, body language, and facial expressions). One expert (PI:003) said reading and writing are also forms of communication.

When asked how their definition of communication ability changes in the context of individuals with NDDs, several experts acknowledged that verbal speech is often limited or non-existent in many NDD populations, so most communication is through nonverbal modalities. For example, one expert (PI:004) said, “…*the change in definition, at least as far as I see it and discuss it with the parents is really facilitating the ability to functionally convey one’s needs without necessarily having to be through a verbal production, so by whichever means possible, even if it’s – hinders some of the social pragmatic aspects, or you know, where we traditionally would expect communication*.” Another expert (PI:008) noted that often times these nonverbal modalities are used successfully and are well understood by parents, but may not be understood across environments and communication partners. For example, they said, “*So, I think that there is kind of a standard of what we would consider receptive understanding and expressive, maybe using verbal speech. That can look very different in someone with [a] disorder or that might be neurodiverse. So a lot of times we find that they are communicating, however there may be a lot of reliance on caregivers that know them really well that intuit a lot of what those things mean. They’re not universally understood by people at large or unfamiliar communication partners. And so, they may be quite successful at communicating with the people that they’re close with using body language and vocalizations and subtle changes and facial expressions. But typically these aren’t things, methods of communication that without some other modality that’s more universally understood, maybe signs or speech generating device that allow them to communicate across environments and across communication partners*.”

Another expert (PI:001) noted that depending on the NDD, a range of modalities may be used or one modality may be dominant. For example, they said, “*Some disorders will be restricted to one modality for communication, whereas others may employ a repertoire across those different ways of communicating*.” This expert also noted that this statement is true within one NDD as well, as there could be wide heterogeneity within one group. Another expert (PI:002) said something similar, noting that communication can look very different even within one disorder, saying, “*So, some disorders – and even within disorder – there’s different mutations. So, like in SCN2A we have a loss and gain of function, those are two different functional presentations of the disorder. And so, in the kids with loss of function, their social communication is often more impaired than kids who have a gain of function. And so, I think there’s variability even within disorder*.”

Two experts (PI:002 and PI:008) noted that unlike with communication in typically developing children, communication ability is not dependent on age in individuals with NDD’s. Similarly, you must look at things like cognition and motor ability when considering communication in the context of individuals with NDDs. For example, one of the experts (PI:002) said, “*So, there’s lots of uneven development that happens in our kids with neurodevelopmental disorders. They’re not a 12-year-old who has communication skills at a six-month level, they may have motor skills at a 5-year-old level. And so, they’re not all even. In a typically developing child who is six months of age, all their skill are generally around a six-month level. But when you have a child who is older and developed atypically you have really a lot of variability in skills. And so, how a child is 10 years old functioning at a six-month-old level from a communication standpoint, they might demonstrate higher social communication skills because they have the motor capacity to do so. So, you might think about those other skills that are there in other domains and how the child is using them to support their communication.”* One expert (PI:006) said pragmatic (or social) communication has to be looked at differently in the context of individuals with NDDs as well because some individuals will also have Autism Spectrum Disorder (ASD) which may impact how they communicate socially.

#### Levels of Communication Ability

Most experts (*n=*6) described ‘advanced communicators’ as those that use verbal speech. Two experts said high level communicators could also be those who are using an AAC device very sophisticatedly (e.g., combining multiple symbols together). Three experts noted some individuals with NDDs may have normal language, but it might just be an issue with articulation or that they have ASD which impacts receptive and social communication. For example, one expert (PI:004) said, “…*so I see a lot of, you know, from the very minimally verbal/nonverbal/zero communication, very aloof children who don’t have any gesture language, don’t really have any eye contact, don’t really have any babbling. You know, there’s a very profound receptive expressive language delay all the way through to, you know, individuals who are 12, 13, 14 that I’m diagnosing for the first time with autism based on a wealth of more social pragmatic impairments, but they have very clear functional communication. I mean, they use, let’s say, the expected amount of words. They can, you know, form conversational language. It’s just the nuances within that. That varies, though*.”

Two experts (PI:001 & PI:008) said high level communicators are understood by more people. For example, one of the experts (PI:001) said, “*So, the individual may tend to be more on the say have more language versus more signs, but within the disorder, there will be a spectrum where that one individual will be what you might say was higher functioning. In other words, they have the ability to communicate with more people in the community because it’s spoken language, which is what most of us do, whereas other individuals will be more at the level of gesture, which will have a limited ability to communicate outside of their family or caregiver group*.” Two other experts said high level communicators communicate unprompted, which is another level of independence. For example, one expert (PI:002) said, “*It’s important to ask if they’re getting prompts [from the parents] because that gives you a sense of how independent the child is. If you’re at home, say what do you want? And then they say I want banana or whatever, that’s one thing. But if they go to school and the teacher doesn’t know to ask them, what do you want? They could just sit there all day hungry. So, it’s different. And the idea is with skill development you obtain a skill with supports and then you start to fade the supports. The goal of them being independent and using it when needed all the time*.”

Conversely, experts described low level communicators as those with minimal or no verbal speech that may only utilize eye contact, facial expressions, sounds or physiological/unintentional reflexive behaviors to communicate. For example, one expert (PI:003) said, “*When I work with really, extremely – children with the most extreme communication problems, many of their communication is interpreted, so I also include interpretation – and that’s based on physiological reactions – they’re hot because they’re sweating, they cry in a certain way when they’re in pain versus they’re hungry or they’re sick, and good caregivers are able to differentiate those kinds of signals*.” One expert (PI:005) also said low level communicators often have lower receptive and pragmatic communication and poorer fine motor ability. Two experts (PI:007 & PI:008) said these communicators are just learning cause and effect. For example, one of the experts (PI:008) said, “*Kind of at the basic level, it’s cause and effect. That if they do something that something else will occur. And trying to build some understanding with that. We try to figure out, even if it’s the very most basic reflective response or there are things that – what does the child enjoy or like or dislike. Usually, even if it’s kind of unintentional, the family can tell us, “Oh, they really like when music is playing,” that kind of thing. Maybe their body just calms a little bit. And so we will use that to try to build some cause and effect understanding using a strategy that we call verbal referencing. So, “Oh, I see that you are smiling or I see that you’re body calmed. I think you really like this music, it seems to make you feel good.” Or conversely, “Oh, you were grimacing or I heard you turning on your voice and you did not sound happy. I wonder if you are uncomfortable, if you’d like me to move you*.” One expert (PI:002) said some low level communicators that are very afflicted may be in a “minimally conscious state”.

#### Assessing Communication Ability

Communication experts were asked to provide details about how they assess communication ability in individuals with NDDs. Seven out of the nine said they use at least one standardized measure (caregiver reported or observer-reported measure) to assess communication ability. Depending on the number and types of NDD’s the expert worked with, some experts said the measure chosen for each child depends on the child’s baseline communication (e.g., do they appear to be higher functioning with words or are they non-verbal?), as well as their developmental age. If the child presents to them with verbal speech, some of the experts would also assess articulation, etc. Two experts (PI:008 & PI:009), both speech-language pathologists, noted completing standardized measures was necessary for diagnostic and insurance purposes. One expert (PI:002) said although the standardized measures “*were made for kids who have a typical trajectory of development*” and “*may not give you information about that child’s optimal functioning*”, they still give information about how that child performs under the same context each time. This expert did feel that these types of measures do well at capturing communication ability for the higher functioning children who are verbal.

Almost every communication expert (8/9) said that they also gather qualitative information from parents when assessing a child’s communication ability. Two experts created their own informal checklists to ask parents questions like, “*How do you know what they want, how do you ask questions, how do you engage*?” Experts said they also inquire about what motivates the child to communicate and what modalities the child uses to communicate at home. One expert (PI:002) said they may consult with the child’s teachers to understand how the child is communicating at school. For example, they said, “*So, I take a I guess – let’s see, one, two, three, four, five-pronged approach. The first is obviously I’m a neuropsychologist so I’m going to use standardized measures and compare to standardization sample. So, that’s one method. Another method is interviewing families and just getting qualitative information about how their child communicates and comparing that to what I know about typical development and then also what I know about these disorders. So, that’s the second. The third is have the parent fill out standardized questionnaires, so like rating scales that are standardized to a standardization sample. And then the fourth is I look at school records and or talk to teachers and I have them actually fill out standardized measures as well or just look at qualitative information about their communication. That’s the fourth. And then the fifth is just my informal observation of the child..*. *So, it’s like how consistent are the different sources of information? And then if they’re different, why*?”

After the expert gets a better understanding of how functional the child’s communication is, they then begin planning therapy and goals. For the individuals who are lower functioning, the goal is to begin building symbolic language. For example, one expert (PI:004) said, “… *if we’ve been working, say, with a four-year-old on things like toilet training and that type of thing, because they have a severe language delay and that type of thing. We’re seeing that the child is now displaying signage that they want to go to the toilet, or something like that, and that’s what I’m looking for in that instance*.” This expert mentioned goals are very individualized because families often have different priority/focus areas they want to work on. Similarly, when conducting repeated assessments, experts recognize every child is going to move at a different pace (depending on many different factors). For example, one expert (PI:006) said, “*The other assumption that I make is that every kid I am working with is going through stages of development, and if they are not acquiring language or different tasks at this age, that doesn’t mean they’re not going to acquire it in two years. And, it might not change what I think is their ability to achieve mastery of a skill. It’s just it might take a little bit longer and might look a little bit different*.”

#### Health Impacts on Communication Ability

Experts mentioned a variety of health issues that can impact the communication ability of a child with an NDD. Four experts said hearing and vision issues may impact what type of AAC device a child can use. For example, if a child has cortical vision impairment (CVI), this will impact how the device needs to be positioned and how symbols need to be programmed (e.g., against dark contrast, as a physical picture vs. cartoon picture). For example, one of the experts (PI:008) said, “*Other times, it [CVI] might impact the size of the symbols, if we make them high contrast, or if there are colors – maybe the child sees a vision therapist and they decide yellow on a black paper is more helpful, we can make the system match them, whether it’s low tech or high tech. So we try to take all of them into account… We may also try to do a system that is auditory plus visual, or just auditory if we really think the vision is being that impacted. I, personally, have seen that even with that label, most of my kids with CVI can still use an eye-based device. Not always. But a lot of times it does not inhibit us from using vision. And then they get the auditory feedback, as well, when they make a selection*.” One of the experts felt CVI might also impact a child’s eye contact. One expert (PI:007) said hearing issues can impact articulation.

When discussing health impacts, four experts talked about how seizures impact communication ability. All four experts said seizures can cause either permanent or temporary losses in skills and may be responsible for the variability in skill acquisition. Examples can be found Table S89. One expert (PI:009) felt sensory issues could make a child hyper or hypo sensitive which could cause the child to act out or withdraw themselves during certain situations, impacting communication. For example, they said, “*And a lot of children with neurodevelopmental have sensory issues. So, they are not receiving input through their senses in a normal fashion – in a normal balanced fashion. And, therefore, they cannot respond just physically, physiologically, to the environment and to the people who are trying to communicate to them in a normal fashion. And, therefore, their bodies are not grounded. They’re not settled in their own body where they can just then receive the input from the communication and the communicative partner in a normal fashion… So, all the senses, if they’re not working in a – or functioning in a normal typical fashion, they’re either hypersensitive or hyposensitive*… *And children with sensory issues are gonna be acting out, meaning in quotations. Some say and “acting out” and that they’re trying to cope with their communication disorder, or in this case, her apraxia and her sensory issues…you know, children develop compensatory behaviors by running around or pointing or they shut down, so you’re gonna have a “flight or fright” response. It’s really what’s most typical because when a child’s not being – they’re just trying to cope, and they’re at the mercy of the adult, who their caretaker is. And so, if the caretaker is not able to communicate with their child, they are doing the best they can, but there’s a mismatch. And so, that’s where I come in. And so, depending on so many variables, it can be the education level of the parents or the parenting style, or it can be the severity of the language delay or the disorder that’s gonna be impacting how that child comes to me. Some of them are gonna shut down because the world is too overwhelming. And so, I have to really – I coach the parents, and I teach them how to meet the child where they’re at, at their communicative ability. And then I show them how the child is communicating and how we go from there. So, they can be nonverbal and extremely either shutting down or extremely hyper running around, you know, flight or fright. And that’s even – a child who can have pretty typical cognition*.”

Table S89. Quotes from clinical experts describing their opinions on seizures and their impact on communication.

| Subject ID | Quote |
| --- | --- |
| 001 | *[a regression in communication could signal] Any number of things, either is it – well, it’s a red flag that requires further investigation because this could be due to a mood issue, a sleep issue, an epilepsy issue, a progressive nature of the disease issue that I think should call your attention to address.* |
| 002 | *Sometimes it [a communication skill] will come back if they have the therapy there to do it. But sometimes it doesn't unless they’re using it consistently either functionally or in therapies. I’ve seen both where sometimes it maybe just doesn’t come back because then they have increased seizure burden or epilepsy formed burden and then it doesn’t come back and they’re at a totally different place developmentally. But sometimes yeah it can come back if they just get practice with it.* |
| 003 | *I find that kids who have lots of seizures tend to learn, and then they lose what they’ve learned. That’s a real problem for kids. Vision, visual problems. And then, all the comorbid – they have visual problems along with seizure disorders and motor control issues, and we often see that with this group. Motor, vision, hearing – I guess those are the main ones. And then, just not feeling well, just some of those issues about their difficulty with eating, so they’re being tube fed, and you have to be concerned about nutrition. There’s a whole plethora of potential difficulties, from sensory systems to motor systems and, of course, the cognitive issues.* |
| 007 | *Our kids are not as simple as one diagnosis almost ever. It seems like where one is there are many other diagnoses. So, there’s that that plays into whether something is mastered because a student may be consistently using a skill for a time period, and then seizure activity may interfere. And knock us back where you feel like, “Okay. This is a skill we’re gonna keep working on.” So, it can be hard to define that mastery.* |

#### Change over time

During the concept elicitation interviews, communication experts discussed how communication abilities change over time in individuals with neurodevelopmental disorders (NDDs) including, what factors affect changes, when the changes typically occur, and what changes are meaningful. Communication experts also discussed what early skill acquisition and skill mastery look like.

Four experts said changes in communication ability cannot occur unless good, quality therapy is being provided to a child. Similarly, without therapy or providing access to different resources (e.g., AAC devices), it is impossible to know what the child may be capable of. For example, one expert (PI:003) said, “*Continuous instruction, a good therapist or teacher. That’s probably the key [factor]*.” Another expert (PI:001) said, “*Well, I think that there’s an aspect of they can often be quite impaired from – compared to neurotypical individuals, but yet when provided with appropriate resources, they can do things that are often grade appropriate*.”

Another expert (PI:008) said, “*We see a lot of patients that come to us from across the state and actually outside of the state, too, who are getting therapy services and perhaps their clinician just has not felt like they are trained enough in AAC. Even though AAC is technically under the scope of all therapists, many do not feel comfortable with it. They feel like they have limited to no training. So, they’ll say they’ve just been working on verbal speech with the child that has been non-verbal. And two years have passed, just didn’t feel equipped to provide anything else in the meantime. So, I would say caregiver buy in and carry over and high quality, consistent therapy services is pretty critical, as well.”*

Like caregivers, communication experts said changes in communication skills happen slowly. For example, one expert (PI:005) said, “*I would say for more of my students it is a slower pace. We’re really just kind of planting the seeds to see that grows with the future*.” Another expert (PI:008) said, *“I think sometimes the buy in [from caregivers] is hard because it is extremely slow. We’re in this for the long haul. We always use the analogy, just like you speak verbally to a child for their whole first year of life and they might not say one word back to you until they’re close to a year of age, that same volume and type of input needs to be provided with these language systems that we’re putting in place. We might do that type of input for a long time, a year even, before you really start to see the fruits of all the labor and time that you’ve put in. For some caregivers, that’s really hard. They need something or they want to see more. They hope that it takes off more quickly. So, we try to help them look for those subtle things that we’ve been measuring. “Oh, but wait, did you see how she paused her body when you modeled more on the device? She’s noticing that. She’s listening. That’s what we want to see.” And so, I think sometimes you have to help families figure out how to measure it other than just number of symbols or number of words.”*

Experts mentioned a variety of different factors that can influence changes in communication and contribute to the slow nature of skill acquisition. Four experts said one factor that influences the pace at which an individual learns a communication skill is the modeling and support the parents, school system and/or therapists provide. Modeling of the communication skill across settings and partners allows the child to receive repeated exposure. For example, one expert (PI:002) said, “*Yeah, so parents have different levels of ability to engage their child in terms of practicing and making them engage in skills. And there is so much family variability in terms of that piece…So, I think that obviously the more opportunity the child gets to demonstrate the skill and practice the skill, it’s going to be good to a certain extent*.” Another expert (PI:008) said, “*I’m going to say the biggest factor I personally have seen, regardless of the child’s cognitive ability, is how often it’s being implemented and carried over at home. I’ve had patients that are very much what I would consider emerging make amazing progress because of the follow through, the carry over by the caregiver… The more buy in we have at home, the faster [we see changes]. On the other hand, I’ve had patients that cognitively are very much farther along in their communication but [the] family kind of sees the 30 minutes with me once a week is the only time that it gets worked on. In that case, progress can be painfully slow... So, I think as far as when they really master it just depends on how consistently they’re getting that support at home. That varies so greatly from patient to patient.”* Two experts felt the amount of time and modeling a family can put in may be related to socio economic status. For example, one of the experts (PI:003) said, “*Parents who are able to provide the services that they need – there’s all kinds of health inequity, and if we can get by that. Socioeconomic certainly has a bearing on the out – I think if we were to study that, has some bearing on the outcome simply because they’re able to afford or get their children to the services and make more demands on the school system*.”

Four experts said changes depend on the NDD disorder and that skill acquisition will be faster in certain disorders, depending on cognitive ability and severity of impairment. For example, one expert (PI:005) said, “*With the severe level of students that I teach, it does take a very long time to see [improvements] that start to come together and kind of bridge that gap. But now within a mod setting, that does become a lot quicker. I think that’s just their cognitive ability to retain and reciprocate.* *Within my experience with students in the mod classroom and the individual with Malan syndrome, I would say I have seen improvements even from the start of a nine-week period to the end of a nine-week period. My students* *in the severe classroom, it could take two to three years before we really start to see improvements there.”* However, one expert (PI:009) cautioned that “*Every patient is an individual and no two children manifest the same disorder in the same way.”* Likewise, the types of changes you would expect to see are going to vary by disorder and an individual’s baseline (i.e. where is the child starting?). For example, for a child with a higher baseline (e.g., a more advanced communicator) a goal could be for the child to start communicating about a wider array of topics over time (e.g., the child begins communicating about more things other than requests). For example, one expert (PI:008) said, “*Maybe if they’re just request[ing], we want them to expand to demonstrate communicative functions consistently over an amount of a time... So how can we get more sophisticated things like commenting, sharing personal information like your name, your birthdate*.” This tends to run alongside the developmental growth in what the child is interested in communicating about.

One expert (PI:004) said the changes you might see in a child will also depend on what the parent is prioritizing or choosing to focus on. For example, the expert said, “…*if we’ve been working, say, with a four-year-old on things like toilet training and that type of thing, because they have a severe language delay and that type of thing. We’re seeing that the child is now displaying signage that they want to go to the toilet, or something like that, and that’s what I’m looking for in that instance. Right? If I’m seeing a child who the parents are very focused on evolving, let’s say, social abilities or evolving ability to produce gestures, and signs, and that type of thing, that’s what we’re gonna be focusing on, in terms of is he pointing*…” This expert was also alluding to the fact that typically, from a therapeutic standpoint, they measure change in terms of moving from non-symbolic to symbolic communication. Three other experts (PI:003, PI:005 and PI:008) also spoke to this. Some example quotes included:

- “*I guess what hopefully happens is that you often move from – early on, it’s a lot of interpretation, and then they become more competent in being able to do things like – we talk about being able to specifically request things that they want*.” – PI:003
- *“Maybe if they’re just request[ing], we want them to expand to demonstrate communicative functions consistently over an amount of a time. So maybe they’re starting to reject and do this in a way where they’re using more language. So, we’re placing some of those oh, I pushed the object away with a symbol*…” – PI:008

Change in communication can really trend up and down and is often times not linear. For example, one expert (PI:002) explained it as sometimes when a skill is emerging, it can be fleeting and variable and happen really inconsistently. They said, “*This happens a lot with the reporters – is that these measures were made for kids who have a typical trajectory of development. They were not made for those who have neurodevelopmental disorders. Those that have not linear development... and a lot of times kids especially with these rare neurodevelopmental conditions they will be working on a skill in therapy, and they’ll have it mastered in therapy and then they move onto something else in therapy and they lose the skill. So, they’re not able to maintain it once they’re not practicing anymore.* *Sometimes it will come back if they have the therapy there to do it. But sometimes it doesn't unless they’re using it consistently either functionally or in therapies.”* Experts also mentioned how other medical issues, such as seizures or illnesses, can affect communication and cause temporary or permanent regressions. Some example quotes included:

- “*I’ve seen both where sometimes it [a communication skill] maybe just doesn’t come back because then they have increased seizure burden or epilepsy formed burden and then it doesn’t come back and they’re at a totally different place developmentally. But sometimes yeah it can come back if they just get practice with it.” – PI:002*
- *“I’m thinking more my classroom the varying abilities could easily be their sleep patterns, their overall health, their inconsistencies of attendance, if we’re off schedule versus on schedule*.” – PI:005
- “*…with NDDs so frequently there’s other health complications that can impact the day-to-day… Our kids are not as simple as one diagnosis almost ever. It seems like where one is there are many other diagnoses. So, there’s that that plays into whether something is mastered because a student may be consistently using a skill for a time period, and then seizure activity may interfere. And knock us back where you feel like, “Okay. This is a skill we’re gonna keep working on.” So, it can be hard to define that mastery*.” – PI:007
- “*I’ve had patients be doing amazing and then go through some seizures, maybe a shunt failure, different things like that. And then we’ve had to kind of regroup. And go back and do some relearning. I can only imagine how hard that is. But I feel like part of my job is to help the families see the change that is happening if they’re not able to themselves*.” – PI:008

Finally, two experts (PI:007 & PI:008) said a child’s motivation and willingness to engage in communication, and how they might want to communicate, can change over time. Quotes included:

- “*So, my motivation as a child versus as an older student may be very different as my experience with things changes. Right? So, experience shapes how I react to things, or what things I’m interested in, etc. As a young child I feel like there’s a wider range of experiences that maybe would be new that you can tap into. You have a huge advantage in that there’s so many things that a student hasn’t experienced yet that you can present to try to gain motivation or to try to reinforce the communicative behaviors that you want, etc.*.. *And depending on the experiences that they’ve had I feel like so much as a student gets older is based on the experiences that they have had. To use a simple example, if the whole world has been an iPad to this child, if the behavioral system at home has become, “Here’s an iPad, spend some time with it, lots of time with it” you’re gonna have a really different evaluation than with a young child who is not as focused on that device. You can really get stonewalled in your initial experiences with some students when they are so focused on one very preferred item or experience as they get older. It can be hard to if turning pages in a book is what is the preferred activity, and that’s maybe more consistent behavior. It can be hard to break through that to kind of get the interaction*.” – PI:007
- *“Maybe their engagement or their interest in communicating, that can be one of the hardest to establish, I think, at the beginning. Their regulation and their interest in communicating, their desire to communicate. Not that anybody’s not wanting to do that. But just building some understanding with that and that this helps us kind of get what they want. I think that can be one that varies… I had a patient with SYNGAP that, I think we spent, I don’t know, four months coming in, establishing a routine, really following her lead, and just getting her comfortable with me, with our activities. We did a lot of things around her interests. We modeled without expectations on a system. She didn’t even have to look. We started with low tech, and we would just point to the symbol and model it while we were continuing to do an activity. The progress was very, very small. But over time with that consistency, she actually ended up being an excellent high tech user. It took a whole lot of time of just meeting her where she was at, helping her to feel safe and regulated with the environment, and building up a desire to engage and communicate. And then after that, the progress was much more what I would say measurable and typical. I mean, I think it would have been easy to abandon it within those four months, assuming it wasn’t going to work out. I do think her age helped. She definitely, obviously, was getting older during that time. And she was getting to know me and I was getting to know her. And I think that really can’t be underestimated*.” – PI:008

#### Caregivers as reporters

Communication experts discussed the different communication modalities caregivers are good at recognizing. Many communication experts (*n=*4) stated that parents are good at noticing and cataloguing the verbal words their child has. For example, one expert (PI:007) said, “*Or if I say ball, or even an approximation of ball, that’s gonna be much more understood versus if I see somebody else playing with the ball, and I start crying. A parent may not make that connection as quickly*.” Some experts (*n=*3) said parents are good at noticing and interpreting a child’s different sounds/vocalizations. For example, one expert (PI:003) said, “*And then, families will tell you that they cry when they’re hungry, and they also cry when they’re in pain, and there’s a distinct difference between a cry when you’re hungry and a cry when you’re in pain. I absolutely believe that.”* Three experts said caregivers are also good at interpreting gestures, like a finger point. For example, one expert (PI:008) said, “*I find they’re pretty good with gestures that we would expect like pointing or pushing away, things like that*.” Another expert (PI:006) said, “*The other thing I think caregivers can easily pick up is pointing… [or] when their hand is placed on the cookie jar, the caregiver is aware that their child wants a cookie, and seems to pick up on that relatively quickly*.” One expert (PI:001) said many of these behaviors are only understood by the caregivers who spend “*24/7*” with the child and have picked up on the consistency of the behaviors.

Three experts said caregivers are good at recognizing expressive communication; specifically, when their child is requesting or expressing likes or dislikes. These requests can be in the form of symbolic gestures and signs, or they can include things the child is “looking at, or reaching for, or grabbing for”. One expert (PI:007) said, “*I think a lot of parents are maybe better at recognizing requests in the more traditional senses*.” Another expert (PI:005) said, “*I think initially just because they come to me so young, they really pick up on likes and dislikes. I think that’s probably the biggest first component for them*.”

Similarly, experts said caregivers are good at recognizing their child’s feelings and emotional states. Caregivers know when their child is frustrated or upset and are good at picking up on non-verbal ques that indicate this. For example, one expert (PI:004) said, “*Obviously, it’s imperfect, but they [parents] know better when a child is grimacing because…they’re constipated or when their child is grimacing because they are disgusted at something, or when they’re grimacing because they’re sad and that type of thing*.” Another expert (PI:007) said, “*I would say the place where most parents are able to recognize communication in most is maybe in their child’s emotions, right? I think as parents they’re seeing and understanding the general emotion that a child is expressing in whatever attempt they make at communicating*.”

Five of the communication experts stated that caregivers can have trouble noticing all the different forms of non-verbal (“subtle”) communication such as eye contact, and body language. Two of the experts said parents, in particular, do not understand certain behaviors their child does that actually indicate they are listening and taking in information. For example, one expert (PI:008) said, “*Or more subtle body language like turning away or that calming your body, no vocalization could be a sign that they are comfortable with something versus vocalizing to show discomfort. I find that a lot of times we take a calm body to be listening and taking in information. That is communicating something, in fact. I find that those things don’t usually come about until I ask specific questions.”* Another expert (PI:009) said, “*I was gonna tell you that sometimes parents misunderstand. So, if a child is trying to seek, he’s hyper and he’s seeking proprioceptive input, so he wants to get grounded. He wants to feel his body where it is so he can feel comfortable in his own body. The parent might think, “Oh, my child doesn’t listen. He’s not paying attention. He’s acting out.” Rather than, “He’s really trying.” When you see that, that’s how he’s trying to pay attention. It changes their focus and their attitude because some parents might think that that’s a bad, again in quotation marks, behavior.”* Relatedly, two other experts said caregivers have a difficult time interpreting other types of “bad behaviors” and their communicative functions. For example, one expert (PI:007) said, “*Is there [sic] communication in the fact that I’m crying, or that I’m hitting my head, or that I’m biting my hand. Right? Those things are telling me something, it’s a communication attempt… Parents know that their children are reacting, but they may not necessarily understand the communicative intent, and how to break that attempt down into the components of how to consider looking at what that attempt means*.”

Three experts said caregivers have a difficult time recognizing their child’s receptive communication skills. For example, one expert (PI:005) said, “*I have one student who came to me. He’s kindergarten, and immediately I was like oh he’s got it. I think receptively it’s there…He has the ability to look left to right and hold that gaze. When I share that with the family, I just think they miss the mark*.” One expert (PI:003) felt caregivers overestimate their child’s receptive communication skills, saying, “…*and parents say they understand yes/no questions. And then, when you really push them, they say half the time, they get it right. You know that’s just chance.”*

Three experts said caregivers can misinterpret perseverative or echolalic communication. Two experts said parents sometimes do not notice their child is using the repetitive or echolalic communication in a functional way. For example, one expert (PI:004) said, “*So, sometimes, it’s part of the learning process and I think that some parents either undermine it [echolalia] and I think this is just something that they told me it’s fine and it’s very clearly, you know, not something that you would expect, or… they fail to notice that there’s a lot of neo-Socratic language, a lot of scripted language that’s made functional by the child*.” This expert also noted that parents can be concerned about echolalia or perseveration, but depending on age, this could be a typical behavior. The other expert (PI:002) said caregivers can misinterpret preservation as meaningful, but it might not always be. For example, they said, “*So I would say especially in autism. A lot of times repetitive stereotype kinds of vocalizations. Sometimes families are like oh they’re telling me about their day. And sometimes nonverbal vocalizations are where kids are like, yada-yada and clearly there’s jargon. But other times it's like they’re self-stimming and it’s not necessarily got a communicative intent at least from I think how most people would view it. But if their family believes it, hey who cares?*”

Two experts said parents may not notice certain abilities, but also may not witness skills if they are not in the day-to-day routine. For example, one expert (PI:005) said, “*Yes. It’s hard because, especially in an ID-severe classroom, a lot of times parents either, 1.) don’t see the ability or, 2.) don’t know how to access the ability*.” The other expert (PI:006) said, “*It took them [the family] five or six years to realize their child knew the alphabet because they never said the alphabet. And, it was someone else that let them know that their child knew the alphabet. And, these parents are on top of things. They’re observers. And, it took someone else to be like, “Oh, yeah. No. They can point at it.” But, who would’ve guessed to have laid out the alphabet for the child and had the child point at, “Show me the letter D?” That just wasn’t in the wheelhouse piece*.”

Four experts said some caregivers are just better at observing and recognizing their child’s communication behaviors than others. For example, one expert (PI:003) said, “*Some people are just better at it, and that’s not connected to socioeconomic, it’s not connected to race, it’s just some people are just better observers, and they respond to it, and as a result, I think their children are more comfortable and seem to be less agitated*.” Another expert (PI:008) said, “*So, I feel like – and I should say I feel like a parent is either really good at… [providing] us with a wealth of things they notice. Or they possibly, due to interactions with other providers, or just this very rigid definition of communication, they don’t know how to answer that question*.”

### NDD Specific Quotes on Novel Communication Topics Identified During Interviews

Please note, the following sections include examples from participants across all cohorts. Thus, the child’s diagnosis is listed alongside the participant ID number to provide relevant context.

#### Feelings, Emotions, and Bodily Sensations

A number of caregivers said that their child could communicate, or has attempted to communicate, their emotions or how they are feeling (e.g. when feeling sick, experiencing a belly ache, etc.) using symbolic communication. Examples can be found in Table S90.

Table S90. Quotes from caregivers describing their children communicating their feelings, emotions, or bodily sensations using symbolic communication.^1^

| Subject ID | Neurodevelopmental disorder | Quote |
| --- | --- | --- |
| PI:102 | SYNGAP1-related intellectual disability | *A new kind of emerging skill, she's starting to have words for some of the emotions… before she really only had behavior to tell us how she was feeling. So, now that she can say, “Mad,” that helps, instead of acting out in an aggressive way, or a self-injurious way* |
| PI:103 | SYNGAP1-related intellectual disability | *He has a gesture for when he’s sort of mad* |
| PI:205 | STXBP1-related disorders | *She’s still not great at it, but she can kind of point to the body part and let me know that she’s not okay*. |
| PI:304 | Hunter syndrome | *There’s times when he’s tired and he’ll tell me, “Mom I’m tired.”* |
| PI:306 | Hunter syndrome | *And also expression words, he started using, like that he was happy, he was sad.* |
| PI:506 | Malan syndrome | *So, he'll just yell like, "Happy birthday to you," when he's mad, and he'll be like – yeah, I'm sure someday he'll curse at me, but until then, I'll take "Happy birthday to you." But he can't say, "I'm mad," "I'm frustrated," because he can't that word out right away, yet. So, he will yell just random phrases, like, "Happy birthday to you*… *He has started to get a few new sentences, like "You're being mean to me." He'll say that, because we've been working a lot on self-advocacy skills with him*. |
| PI:703 | HNRNPH2-related disorders | *…* *so I feel she can at least communicate to us if she’s hungry or if something hurts. “I got a boo boo, ouch.” Or she can point to it*. |
| PI:705 | HNRNPH2-related disorders | *She will communicate sometimes if she’s feeling happy or sad, although this is newly developing, so we’re not sure yet if that’s intentional because that is on a device.* |
| PI:1003 | BBSOAS | *She's improved a lot in communicating what her feelings are; other than just crying, yelling, she's gotten a lot better of trying to explain what's wrong and how she's feeling [with verbal words]. If she is upset about something before, she would just continue to cry without verbalizing what the issue was.* |
| PI:1011 | BBSOAS | *I mean, he’ll say, “I’m mad.” When he don’t get his way, or whatever, but I think that’s just a learned thing that he has. He doesn’t – like I don’t hear him like rationalizing. You know, like explaining why he’s upset, or what would change, you know, like the – like explaining to me like this boy, you know, he said this to me or this made me upset.* |
| PI:1106 | GRIN2B-related neurodevelopmental disorder | *Just if she’s tired, she’ll say, “night-night”. Letting us know what she needs, like if she needs to rest or if something is hurting, she’ll say “hurt”.* |
| PI:1108 | GRIN2B-related neurodevelopmental disorder | *And so now, when she responds, you can see a lot more of her emotions, whereas before, we kind of just knew she was in a lot of distress, or if she was really overjoyed. And we had no real in-between. Now, she will even tell you, “I’m so sad.” “I’m angry.” And is able to literally give you a little more emotion.* |
| PI:1206 | HAFOUS | *He can express that he’s angry, he can express that he’s hungry; he can express he wants to go somewhere, or how much he enjoyed something, or whether somebody’s annoying him, or whether he’s hurt, or whether he’d like your attention with something – whether he’d like to play a game, whether he liked – individual components of the game. Whether he’s not enjoying it, whether he’s not feeling up to it today – “Actually, I’m really tired. Can we go do so and so instead?”* |
| PI:1207 | HAFOUS | *So, we communicate about their wants and needs –what’s their feelings – what’s going on with them at school, at home – We’re pretty much talking about everything at this point.* |

^1^PI:111 said that their child had a gesture/sign for ‘mad’ but issues with the audio in the transcript did not allow the quotation to be pulled.

Currently, there are no questions on the ORCA measure assessing a child’s ability to communicate their feelings and emotions or bodily sensations like pain/illness. However, some of these concepts are likely captured under the requesting, seeking attention, directing attention, and answering questions concepts included in the current version of the ORCA measure.

In addition to the caregivers described above, other caregivers described behaviors that were interpreted by the caregivers as their child communicating their feelings/emotions, and potentially, sickness/pain. However, this communication was not as clear as the examples provided above (i.e. the child signing ‘my head hurts’). It sometimes was functionally successful in that the caregivers were able to interpret the child’s internal state. For example, one caregiver (PI:210, STXBP1-related disorder) said, “*If she’s in any kind of pain, she will kind of whimper or cry out her discomfort and let us know that… You can kind of read more on her face as to whether she’s happy or bored or frustrated or disgusted*.” Other examples included:

- “*She also has a really sophisticated, complex vocabulary of raspberries. Which sounds ridiculous, but she has so many different varieties of raspberry – some that are playful and fun, some that are very angry… And the degree and intensity of raspberry and kind of sounds made in the back of her throat with the raspberry have a lot of meaning. And we can use those to interpret different scenarios as well*.” – PI:608 (SCN2A-related disorder)
- *“We can tell when he’s in pain because it’s more of a cry, versus kinda whining about something, versus happy about something. So, we can kinda tell if he’s okay or not*.” – PI:612 (SCN2A-related disorder)
- “*If she does not feel well, she bangs her head with her hand or bangs her head on the back of something. That is... that's how we know she does not feel well*.” PI:611 (SCN2A-related disorder)
- *“But he always needs some sort of physical contact, I guess. So, he hugs me a lot or [verbally] asks me to pick him up a lot. And it involves a lot of him touching. I guess he’s got a sensory thing with that.” -* PI:806 (SETBPT1-HD)
- “*Sometimes when she’s angry or like frustrated or not getting what she wants when she wants it, she might take her hand and kind of get a little bit pinchy. You know, like you feel like she’s squeezing your hand tight…I think that you can tell when she’s tired…she may not be telling you, but you can see it because she has that gestural things that she does to help you understand how she’s feeling...” -* PI:701 (HNRNPH2-related disorders)
- “*Well, she will bite herself at times – her hand – if she’s very frustrated, or excited, it goes either way…She’ll grab your arms and give you a facial expression that definitely lets you know she’s frustrated or unhappy about something. She’ll communicate that she’s excited by jumping up and down, and smiling, and giggling, that kind of thing*.” - PI:705 (HNRNPH2-related disorders)
- “*It’s more like her tonation can increase or decrease. It can kind of – and you kinda know what mood she’s in… So, a lower pitched sound maybe if she’s just sitting watching television like a music video, it’s more so in regards to if she’s happy, where she just kinda sits there and just that “Mm. Mm.”... We know when she’s frustrated or irritable or hungry. Her tone just kinda changes. It increases or she goes to a cry or she’ll kinda – when we know she’s tired, she’ll kinda just lay down on the floor and kinda mellow out a little bit. So, just over time, we’ve figured it out*.” – PI:1004 (BBSOAS)
- “*It’s very clear if she’s happy or not. She laughs a lot. She’ll make noises when she’s unhappy. Sometimes, she’ll cry. That doesn’t happen too often. But yeah, usually more…just kind of…maybe sort of like a moan, but like a moan of unhappiness*.”– PI:1008 (BBSOAS)
- “*I mean, right now, it’s a guessing game... He’ll cry or, like I said, he’ll throw a tantrum if he doesn’t get something that he wants*.” –PI:1010 (BBSOAS)
- “*He squeals, he laughs. He'll definitely let us know if he's not happy…It's like different vowels [sounds]… Like, if he's upset, he gets real loud*.” - PI:1101 (GRIN2B)
- “*So, she really only communicates about, I guess, a couple of things. But when she’s bothered, upset, or something is bothering her, and then other times, that’s her communication is smiling or glaring. You can just tell the happiness when she’s happy… If she’s bothered and something in her trach is not sitting correct completely, like there’s some air or gap or something, and she’s upset, she’ll make a loud little cry*.” – PI:1102 (GRIN2B)
- “*And it’s a completely different cry [for pain] than we would hear her cry for example when she’s bored or for example she’s tired. So, it’s a certain type of cry. It’s more of a – I would say more of a typical pain crying. We would see tears falling off of her eyes. Yeah, it’s a really sad cry when she’s in pain*.” – PI:1105 (GRIN2B)

Every caregiver of a child with SGS in our sample discussed communication around feelings and emotions. These caregivers were attuned to their child’s specific behaviors that indicated discomfort, pain, upset and/or happiness, contentedness. However, it was challenging for caregivers to fully know what their child was feeling. Please see the SGS typical communication section for more details. One example is included here for illustrative purposes:

- “*We know when he cries, something is really hurting, or he’s not really comfortable because he has a high pain tolerance, we noticed. So, when he’s not feeling really good, we know when he cries, it’s really something uncomfortable*… *Because especially when he’s not feeling well,; because when we hold him, he’s kinda screaming and tensing up, and then as soon as we put him in the rocking chair, he will just loosen up. His muscles will be relaxed. And if he’s crying, when we put him down there, he will stop crying*.” - PI:904 (SGS)

Several caregivers in cohort 2 (*n=*4), cohort 3 (*n=*1), and cohort 4 (*n=*4) also discussed how their child’s anxiety or frustration impacted their communication. Example quotes included:

- “*As part of her disorder, she’s really reluctant to leave the house and she has very severe anxiety. So, she keeps asking me – some of the questions are permission questions. So, she’s saying to me, “Can I stay in the hotel room? Can I stay in the hotel room? Can I stay in the hotel room?” because she’s worried that she’s going to have to do something which is frightening to her which is go and be in this meeting, or hotel, or conference, or presentation. So, her questions also are about permission, “Do I have –?” She’s asking – because she’s trying to figure out a way to avoid what is so upsetting to her*.” - PI:509 (Malan syndrome)
- “*So, I think... she has a lot of sensory processing issues and like I said, anxiety. So, transitions are really hard for her and whenever she's transitioning to something new, her verbal communications shut down*.” – PI:610 (SCN2A-related disorder)
- *“…what happens with the CVI is her eyes will just kind of shut off and she can't see what's around her, so then she's only relying on hearing, which then there's a lot of sensory with the hearing, or there's textures or touches – it just seems with that particular thing when she is in an overwhelming situation she begins to shut down. And so she will cling to my side if we are out in public and just kind of get very anxious and stubborn about what she wants to do because she just needs to sit and decompress a little, if we're at home she'll remove herself from a room and go sit on the couch by herself and either cover her face or something and just kind of rock until everything subsides and she comes back and her vision is restored. But in that process she is not able to really – she forgets a lot of the skills that we've learned with communication – so if I ask her, "[child’s name], what's wrong?" she is not able to give me words, she will just shake her head and say, "No, no," she just can't talk much. Or she'll say, "Just go away," "She needs to go away." And she refers to herself as she often, so she'll just tell me that, "She needs to go away," and so I usually let her be*…” –PI:706 (HNRNPH2-related disorder)
- “*He does not like to be in places he doesn’t know. He likes to go to school, and he likes to be at home. When you take him too many places that he doesn’t know, he just shuts down*.” – PI:1109 (GRIN2B)
- “*Two or three options – he can’t keep it in his head. So, it’s like you have to keep reminding him which options he’s choosing from and perhaps narrow it down a bit, and then – If you gave him four or five, that’s it – it’d be flawed. He could not do that. There’d be too many to hold in his head to work out which of those might prioritize. And then, he just gets really frustrated, and really flustered, and he just gives up completely and storms off – because he's frustrated with himself…He gets confused so easily, and flustered, and frustrated, and angry, and – he’ll just lose the thread completely*.” –PI:1206 (HAFOUS)
- “*And so, he often struggles to give his brother his turn and will just say, “It’s my turn,” even if 10 minutes he had – he’ll bounce back and forth from being able to take turns to not being able to take turns, and sometimes, like if it’s time for him to take a turn and he can’t and, you know, we – I step in and I say, “Hey. This is your brother’s turn. Remember, we’re giving him space, or we’re letting him go through this time.” They push these big trucks around a lot of times he’ll struggle to communicate at that point and I guess they call it like stemming. He likes to make this high-pitched sound over and over again or he makes this sound that’s like clearing his throat, so it’s like, for some reason, I think his speech therapist said it’s like he loses access to speech sometimes when he’s frustrated or struggling, and then it’s kind of hard to bring him back in to the conversation and the game*.” – PI:1203 (HAFOUS)

Further speaking to salience, the idea of a child successfully communicating their feelings, emotions and/or pain, came up multiple times when parents were discussing meaningful improvements. The need to heavily interpret feelings/emotions was frustrating for parents. One parent (PI:202; child with an STXBP1-related disorder) described why there is overlap in wanting the child to describe pain and emotions when children are unable to communicate what they are feeling: “*We have days where she cries. If there’s a string of three or four days, it’s like, “She’s really crying more than normal” or her teacher’s like, “She’s fussier. More than normal,” it’s not anything that we can pinpoint. All we can do is take her to the doctor and have them run a battery of tests and bloodwork to try to find something. Or is it her teeth? Do we need to go to the dentist? It’s really a challenging situation, whereas if she could just come up and say, “I have a headache,” then it’s like, “Okay.” If she can point to her head, and some sort of sign for pain, then it’s like, “Okay, Tylenol. We don’t need to go to the dentist*.”

Another parent of a child with an SCN2A-related disorder (PI:608) said, “*Anything progressing towards being able to do that [communicate pain] more successfully would be huge for our family – particularly with when she’s not feeling good and something’s wrong. For [child’s name], her biggest seizure trigger is visceral pain. Anything kind of internally like gas pain, acid reflux – she’s had kidney stones in the past. Those seem incredibly acute for her – and often trigger seizures. So, trying to understand – usually, when we lose [seizure] control, it’s with one of those going sideways. And then, we’re always kind of left trying to catch up. And retrospectively, I think that there were always kind of signals that something was going on, and we were never able to catch it in time. And it always takes a long time to be able to figure out and diagnose what the problem is, Like, playing the scenario out, if she was able to communicate better and then she was having bad stomach and gas pains, she could have told us about it months ago before losing seizure control, and we could’ve taken interventional steps to prevent it from ever escalating to chronic seizures. Because it always seems like it’s a ramp up, and then once [seizures] are there, then they’re really hard to get back under control.”*

Another caregiver of an individual with SYNGAP1-related intellectual disability (PI:112) said, “*I would like him to be able to understand what's happening in his body. So, he would be able to communicate his needs at that point. ‘Cause I feel like that is a big struggle for us. Like when he's sad or he's crying, I don't know if he's crying because he's hurting, or if he's crying because it's an emotional thing. I think that that would probably be the biggest change I would like to see for him*.”

Another caregiver of an individual with SGS (PI:901) said, “*So, when he’s up for a very long time, he’s very – it’s like he wants you to help with the problem, but he doesn’t know how to say it. So, he will grab hair. He will pull and pinch at your face like he’s clawing at you. He’s trying to express that something is wrong and hurting. But we’ve been to so many doctors, and been admitted into the hospitals. And pretty much, we have not been able to determine what it is that’s giving him a hard time… He is very squirmy, and not relaxed. So, we know that he’s struggling. And so, he’s very hard to hold, and hard to comfort, ‘cause basically, nothing works*.”

#### Commenting

Commenting is considered an aspect of expressive communication and commonly defined as verbal or written remarks expressing opinions or reactions.^82^ Eighteen caregivers in cohort 1, 16 in cohort 2, 8 in cohort 3, and 8 in cohort 4 discussed how their children comment. The examples that they provided focused generally on children indicating their likes and dislikes.

Although commenting was commonly mentioned across the interviews, examples were highly variable across families in terms of positive/negative comments, the content of the comments, and the amount of interpretation required by the caregivers in understanding, and spanning across symbolic and non-symbolic communication. Example quotes included:

- “*She loves Chuck E. Cheese, so she’s consistently talking about Chuck E. Cheese… Basically, she talks a lot about things that makes her happy*.” – PI:108 (SYNGAP1-related intellectual disability)
- “*So, in regards to eating, he’ll use the sign language for more and he’ll say like, “Yum” just as a general term for eating and showing satisfaction within the meal that he’s eating*.” – PI:310 (Hunter syndrome)
- “*This is another really weird word he says when we think he just wants to do something different, is, “Airplane.” But that has nothing to do with anything, but he’ll like, “Airplane. Airplane.” And it’s just – he just wants to do something different, or he’s bored of what he’s doing*.” – PI:311 (Hunter syndrome)
- “*He can talk about what is for breakfast, what’s for lunch meals, food, what he’s doing, what he wants to, where he wants to go, what he wants to wear, things like that*.” –PI:403 (PMS)
- “*It looks like when she comes home from school, she’ll say, “Mom. I’m home.” So, it’s just more than just needs now. So, she’ll talk about classes at school, her friends, she likes to communicate on FaceTime, and she’ll ask to call someone and then want to have a conversation*.” – PI:501 (Malan syndrome)
- “*And then once I stop on that, there’s a huge smile on his face and he claps, and then when he gets what he wants, he’ll make the, “Mmmm,” like he’s enjoying what he’s eating*.” – PI:508 (Malan syndrome)
- “*She communicates about [a favorite sport]. I’m not kidding…players, specifically, how old they are, what teams they play for, whether they’ve given press conferences. That’s the majority of what she communicates about*.” – PI:509 (Malan syndrome)
- “*I guess cooing – a little of cooing, but it’ softer. It’s more pleasant, I would say, not whining. And it’s accompanied by a smile, usually. A little bit. So, we think that’s just a positive thing that he’s just happy and not really that he wants something in return*.” – PI:612 (SCN2A-related disorder)
- “*The other day, I put a dress on her. And she goes, “I’m so pretty.” And that’s because I would put dresses on her, and I would be [like], “[child’s name], you’re so pretty. You look so pretty.”… So, she picks up on a lot of the things we say.”* PI:703 (HNRNPH2-related disorder)
- “*And I think she’s got a lot of phrases by just listening to people and listening to us or listening to the television, her programs on the television. And sometimes, she surprises [us by saying] – like, what’s going on here, or something’s fishy. These are the phrases we would not use in everyday language, but one’s she’s caught on from the TV, and she knows exactly where to use*.” – PI:801 (SETBP1-HD)
- *“So, when she’s excited about something, she’ll squeal and like scream in excitement.”* - PI: 701 (HNRNPH2-related disorder)
- “*So normally, he loves being held. He likes to kind of snuggle in. And with me especially. If he’s with me or his dad, we – we call it him giving us hugs, but he presses his forehead against our forehead… it’s his way we feel of being social, and communicating to us that. And he’s always smiling when he does it, and he just bumps heads, and then just pushes. Just the slightest pressure. And so, we push back just a little bit. And we always say, “I love you too.” And it just seems to be our way of hugging*.” – PI: 901 (SGS)
- “*He’ll ask the guys, “Do you want to have a sleepover?” Or you know, he’ll say, “I’m inviting you to dinner at my house.” Things like that, but always to adults*.” – PI:1011 (BBSOAS)
- “*She’ll say phrases that she knows. She’ll say, “I want XYZ.” Or if she knows certain phrases like, “Let’s go, everybody,” or, “I don’t want it.” “Get down*.” – PI:1106 (GRIN2B)
- “*Let’s see… she’ll say, “More,” she’ll say, “Stop,” she’ll say, “Bad, bad, bad,” she’ll say, “I dancing,” she’ll say, “Sing it,” she’ll say, “Hungry,”… if siblings are misbehaving, she’ll say, “Bad, bad, bad,*”…” – PI:1108 (GRIN2B)
- “…*or like if I give him directions and he doesn’t like what I said, he likes to pull lines from his favorite TV show, from the old Thomas or sometimes I don’t know where he gets this stuff from, but like he’ll say things that he’s heard in similar situations. Something they say on Thomas is, “You are causing confusion and delay,” so if he gets frustrated, he’ll kind of just try to say something snippy to me. He told me one time, “I don’t want to hear your excuses.” Which came from a TV show... He’s reiterating something he’s observed before*.” – PI:1203 (HAFOUS)
- “*So, she will – sometimes she says, “Oh, it’s raining. We’re not going to have recess.” So, she can make that connection. She knows already that when it rains, they don’t let them go out to recess. Or if it’s too cold*.” – PI:1211 (HAFOUS)

Other more infrequent examples of commenting that caregivers provided included their child telling someone they loved them, giving a thumbs up/down, telling someone they were hot or cold, or communicating that they were shocked/surprised. Although there are no specific sections on the current ORCA measure that captures commenting more generally, some of the listed behaviors are likely captured under existing item sets on the ORCA measure.

#### Humor: Comprehension and Use

Five caregivers in cohort 1, five in cohort 2, two in cohort 3, and five in cohort 4 discussed humor as a concept that was relevant and important to their family, with a number of those having a teenage child (13-17 years old). Discussion around humor spanned from the child’s understanding of humor (e.g. laughing at things that should be funny, like a T.V. show) as well as the child’s use of humor (e.g. the child saying something silly to prompt a laugh or initiating a joke). Caregivers also spoke about how meaningful these interactions were, or how they contributed to their child’s social connections. Some example quotes included:

- *“Well, this isn't a phrase, but she had a stuffed animal named – a little baby gorilla named Ooh-Ooh. So, she learned how to Ooh-Ooh. And then as a joke, I would say, “Poopoo.” And then she really learned how to say poopoo Ooh-Ooh, which is like putting four syllables together. And it's kind of like, I guess it's a joke we say. Ooh-ooh, it's a joke. So, that is I guess a humorous joke.”* – PI:212 (STXBP1-related disorders)
- “*But we’ll talk about – we just kinda talk together with him about what show he’s been watching, and what funny thing they’re doing on the show. Because I think he understands… because he laughs at the right parts, and he loves all his favorite parts. But he just doesn’t necessarily – can’t say the words about it*.” – PI:311 (Hunter syndrome)
- *“She knows when somebody’s being funny or somebody’s saying something sarcastically, and it just doesn’t fit. She forms pretty strong relationships with people. And there’s specific jokes that certain people like, for instance, or respond to, and then she would make sure that these are the jokes that are sort of are brought out the next time she sees that person even if it’s been a few months between that type of communication”* – PI:405 (PMS)
- *“But she has crushes on these famous [sports team] players. So, sometimes, she will make – she’ll try and use humor like, oh, she’s going to marry them. Or she’ll say, “Oh, [name] should date so-and-so [sports team] player.” And she does it with some humor in her voice...”* – PI:509 (Malan syndrome)
- *“Her sense of humor’s actually pretty keen. She’s usually the first one to laugh at jokes on TV or being said around the house – even ones laced with a lot of sarcasm. So, I don’t think she really has the capacity to kind of make that a two-way street and tell jokes, but she does have the capacity to respond to things that she finds [funny]… things on TV, and…if it’s later at night and we’re even watching The Office or kinda more adult-esque humor, a joke will fly by the screen, and even before we laugh, she’s laughing hysterically. Which, having her capacity for humor kind of being sophisticated is incredibly funny for us. So, whenever she starts laughing at something from more sophisticated humor standpoint, it ends up being really funny for everybody in the room.”* – PI:608 (SCN2A-related disorders)
- *“Sometimes she also just communicates to be goofy. She just says silly things to get a reaction or because she likes the way it sounds... she likes to say silly things and then have you say them back. She likes for you to repeat what she says*.” – PI:610 (SCN2A-related disorders)
- “*One thing she loves… is that we play with Siri. Like when we’re in the car. And probably a lot of parents do this. So, I haven’t heard about it, but probably a lot of parents do silly stuff like this, when I say, “Okay Siri, how many times did you go poopy in the woods today?” And then Siri says, “That’s not very nice.” And that makes us crack up… She loves kind of poopy humor a lot*.”

“*Lately, she’s done that kind of thing where she’s dressing up my mom’s dog. She’ll put a blanket over it. We have to always watch out for stuff like that, and make sure it’s safe. But she’ll put a blanket over the dog, ‘cause it’s funny, and pretend it’s clothes*.” – PI:802 (SETBP1-HD)

- “*So, she does have OCD, and she’ll say – our name – 60,000 times, and then will just be like, “Doodoo.” And it’s just like, “What? Why?” I mean, it’s like she trolls us in a way. So, yeah, I think she does do things with humor. Or, she’ll jump out and try to scare us, or – do little things like that*.” – PI:1001 (BBSOAS)
- “*He’ll like turn around and fart and say, “That’s my trumpet.” Those kind of like – I’m like – and he’ll like laugh. Like ha, ha, ha, ha. And of course, dad laughs, because boys laugh at all fart jokes. So, little things like that, or he’ll try – if he gets like a knock knock joke and he’ll try to repeat the knock knock joke, and he’s like, “You get it? You get it?” He also definitely gets humor. When he watches like Spongebob Squarepants and things like that, he like dies laughing*.” – PI:1011 (BBSOAS)
- “*But he likes to lick his tray on his feeding chair. It’s so yucky. And so, we’re – he’ll lean his head down and look over at me, and I’m like, “No, that’s gross. We don’t do that.” And so, he knows. And sometimes he’ll still do it and then look up at me and grin. So, I think he understands things like that, so some understanding of that*.” – PI:1109 (GRIN2B)
- “*So, if somebody trips or something, she loses her marbles. She thinks it very funny. If someone’s joking around really loud or whatever, she’ll think that’s kind of funny. Sometimes, when she gets startled, she thinks that funny. She’s almost got a kind of a bit of a warped sense of humor, if that makes sense. She really likes to joke around. If we were nudging her, she thinks that funny. If we tickle her, I squeeze her [sic]. She thinks that sort of thing is quite humorous*.” – PI:1212 (HAFOUS)

Other caregivers reported more general comments about the child’s humor that seemed related to positive affect; for example one caregiver (PI:211; STXBP1-related disorder) said, “*But she certainly communicates when she is…entertained or something is humorous; she communicates that very well.”* Another (PI:1204) described their child as *“funny and kinda witty.”* Currently, there are no items on the ORCA measure that capture a child’s ability to understand or express humor.

#### Self-advocacy

As discussed under the results for children with Malan syndrome, a novel pragmatic skill was reported by two separate caregivers. Notably, both children utilized verbal speech and were relatively advanced in their communication skills compared to other children in this study.

One of the caregivers (PI:509; Malan syndrome) gave an example of her child advocating for herself, *“We took her to see a [sporting event]. It was a very crowded arena. There were 17,000 people in the [venue]. And as we were exiting this crowded arena, someone, I think, stepped on the back of her shoe and she turned around in a very appropriately loud and angry voice, said, “Don’t do that.” And we were surprised and I guess, pleased, that she was expressing how she feels. But the fact that she communicated with a stranger at all, I think, only occurred because it sort of met some threshold for her where she was so angry or upset that this person had stepped on her ankle that she was willing to express herself to a stranger. But other than that, she would never initiate a conversation with a stranger.”* The other caregiver (PI:506) said, “*He has started to get a few new sentences, like "You're being mean to me." He'll say that, because we've been working a lot on self-advocacy skills with him*.”

It was noted during an External Technical Advisory Committee meeting (April, 2023) that there may be cultural implications to consider related to self-advocacy as a communication skill as well as its relevancy to all families.

#### Children who do not utilize symbolic communication often or at all

When children did not utilize symbolic communication or more advanced modalities to communicate (e.g. words, gestures, AAC devices), caregivers were required to interpret non-specific communication behaviors on a regular basis and they reported that it took a lot of time to understand what their child means or what they need. We would expect that some items on the current ORCA measure within certain concepts like requesting or refusal may be endorsed by caregivers like the ones in this sample at least ‘some of the time’. However, there may be opportunities to expand the measure to cover communicative behaviors seen in severely affected children such as responding to familiar voices, responding to familiar routines, and/or communicating happiness/comfort or discomfort/pain.

## Results for Research Question 6: Findings across NDDs

### Frustration Due to Communication Issues & Impacts on the Family

Eleven caregivers in cohort 1, eight caregivers in cohort 2, two caregivers in cohort 3, and 3 in cohort 4 mentioned how frustrated their child can get when 1) they are not being understood by a communication partner; 2) when they are not able to express themselves successfully; 3) or when they are being asked to do something they don’t want to do (Table S91). This was discussed often by caregivers of individuals with SYNGAP1-related intellectual disability (7/12), but was also mentioned by caregivers of individuals with other NDDs. Caregivers said this frustration often leads to behavioral issues. This topic also was identified during the meaningful change discussion as a ‘meaningful’ improvement (e.g., it would be meaningful for the child to develop some communication skills that could be understood by others to reduce the frustrating behaviors).

Table S91. Examples of communication frustration leading to challenging behaviors.

| Participant ID | Neurodevelopmental Disorder | Child Age | Quote |
| --- | --- | --- | --- |
| 101 | SYNGAP1-related intellectual disability | 3 | …*the meltdowns are related to frustration, or not being understood, I think that causes a lot of them.* |
| 102 | SYNGAP1-related intellectual disability | 6 | *I think going back to, like the frustration piece, I think, just the more that she's able to communicate and the more she feels understood, we see a decrease in some of those problem behaviors.* |
| 104 | SYNGAP1-related intellectual disability | 3 | *Sometimes she gets frustrated if she can’t get her point across… And I guess more like when she can’t say what she wants, it’s more like a cause of tantrum.* |
| 106 | SYNGAP1-related intellectual disability | 6 | *Sometimes, if we don’t understand what she wants, then she could get frustrated and that could escalate, but it’s just improving that communication. It does improve her mood and the way that she behaves during the day.* |
| 107 | SYNGAP1-related intellectual disability | 5 | *So, sometimes if he gets frustrated; if he can’t get that [communication] across then he starts acting out…. It’s hitting. It’s kicking. It’s throwing.* |
| 108 | SYNGAP1-related intellectual disability | 16 | …*there are instances where she’s enabled to vocalize what she wants, or people do not comprehend what she wants. And then, that can get her frustrated, and that can trigger the behavior, and that can affect everything else within her day.* |
| 112 | SYNGAP1-related intellectual disability | 7 | *So, sometimes he gets really frustrated ‘cause he's trying to communicate, and he can't really communicate cause he's still trying to use his ACC device for a lot of things. And he's still learning how to use that so, if it's not on there and he doesn't necessarily know a sign for it... or it’s something that he's trying to communicate like his body hurting, sometimes he'll get frustrated, and then at that point is when we start seeing the tantrums, or the meltdown behaviors.* |
| 208 | STXBP1-related disorders | 7 | *He acts out a little bit sometimes. And I think that has to do with, again, the lack of him being able to properly communicate as far as his wants or his needs. Another child might say, “Well, I don’t wanna do that” or “I don’t wanna change to do this or to have to go to do that.” And obviously with him not being able to say something like that or express that, then it turns into hitting, kicking, screaming.* |
| 302 | Hunter syndrome | 4 | *At home, definitely, he’ll get frustrated if we don’t understand [him] or we don’t know…And he kinda gets frustrated, and we get frustrated–after a little while of guessing. Especially with TV shows. He wants a specific TV show on, but we don’t know what it is. So, we kinda play around for a couple minutes, and we’re like, “All right. Mickey Mouse is going on. Just deal with it because I don’t know what you want.” And he’ll get frustrated and “Ah!” – kinda yell out.* |
| 306 | Hunter syndrome | 8 | *He’s got a little bit of anxiety I feel, because we don’t understand. So, he just doesn’t like being asked over five times, “What?” or, “What did you say?”* |
| 309 | Hunter syndrome | 3 | *I think probably it’s his lack of ability to communicate affects him a lot because he gets very frustrated because he can’t express what he needs, and he’s pretty limited* |
| 403 | Phelan-McDermid syndrome | 10 | *When he’s saying something, but the pronunciation is off, and the other person, which sometimes is me, doesn’t understand what he’s saying because the word just isn’t being pronounced correctly or clearly… I think, is one of the things that leads to frustration sometimes for him.* |
| 405 | Phelan-McDermid syndrome | 14 | *When people are completely unfamiliar with her, it may be difficult for them to understand. So, that causes some frustration because she’s saying something, and then they don’t understand or misinterpret it. And she tries to repeat herself, and then they still don’t understand. So, it causes frustration.* |
| 502 | Malan syndrome | 4 | *I mean, for her, I think it's more understanding the answers back to her at this point. Like if she's communicating with us. Because a lot of times, she doesn't get it. And I could tell it getting frustrating on her end. So, I think once she can comprehend the answers back to her, that'll be beneficial to her.* |
| 504 | Malan syndrome | 11 | *So, before it was very difficult for even us to understand what he was saying and that’s one of the reasons why we started using the AAC device. He was becoming extremely frustrated that he was not understood and he wasn’t able to communicate, so when he first got his AAC device, we noticed a difference in behaviors. It diminished a lot of behaviors and his frustration level we noticed lowered a lot, so he wasn’t as frustrated throughout the day.* |
| 508 | Malan syndrome | 2 | *But yes, he’s a bit of a stubborn individual, so he definitely knows what he wants, when he wants it. It’s just that frustration piece of not being able – for us not to know what he wants.* |
| 602 | SCN2A-related disorders | 4 | *You can see the difference where she’s gonna struggle because not everybody else at the house understands her needs. They don’t understand if she starts crying and whining. It’s not that she’s throwing a tantrum. No. She’s frustrated. That’s her way of expressing. She can’t talk.* |
| 604 | SCN2A-related disorders | 9 | *He’ll raise his voice if he’s getting upset or if we’re not understanding. Because he’s got some – I don’t know that they’re words or just sounds – some things that we don’t understand. And you can tell when he’s getting upset that we’re not understanding.* |
| 605 | SCN2A-related disorders | 8 | *Because she just has meltdowns every day over just not being understood…* *She goes after us as the parents and tries to hurt us, or she throws things, kicks things, slams on the walls. Bangs on the doors, slams the doors.* |
| 805 | SETBP1-HD | 3 | *She kind of throws like little tantrums if she’s trying to express something and we don’t understand what she’s saying in the moment*. |
| 810 | SETBP1-HD | 2 | *So, early on, he was having a lot of meltdowns, if you will, if he couldn’t communicate to us what he wanted… So, now that he’s able to better do that, we’re finding he’s having less communication meltdowns* |
| 1001 | BBSOAS | 11 | *Honestly, the medication – You can just see – she was very impulsive. She has autism as well, so, she was very impulsive before – very – quick to hit, pull hair – her own hair when she was frustrated – hit her own head, things like that, but we don’t see those behaviors anymore. I mean, she has to be really, really upset, so I would say like once – a week where she’s really upset because she can’t figure out what it is she wants to say or – how to put it together, but yeah. I mean, thankfully because of that medication, our house is much calmer.* |
| 1006 | BBSOAS | 4 | *So, she comes in and tries to tell me what she wants and I don’t understand or she’s just going “Ah! Ah! Ah!” I’ll say, “Tell me,” “Use your words,” “Do you want this?” “Do you want that?” and if it’s continuous – I can tell it’s not what she wants – she will just get more and more fussy to the point where she is screaming.* |
| 1010 | BBSOAS | 4 | *Well, just the gaining of the signs has been really helpful, because before that, we virtually had no way to communicate with him at all, so that was very challenging. Even just – I mean, as limited as those are, those signs have made a difference for his ability to communicate. I feel like it still is frustrating for him that he can’t really express himself. Sometimes that results in tantrums, but the limited signs definitely has helped*.^1^ |
| 1104 | GRIN2B | 9 | *To be able to talk with people that don’t know him well and have them understand all or most of the time would be a drastic change for him…Because he wants to interact with people, and he wants to talk to everyone he sees. And he – it doesn’t frustrate him anymore, like he kind of understands. It used to frustrate him badly. But it makes him sad that he can’t. And it’s isolating.* |
| 1212 | HAFOUS | 14 | *And I think her level of frustration has definitely increased with her lack of communication…We’ve tried so many things just to see what would get her moving, and not a lot seems to motivate her…She can sign about 10 things now. And that’s probably just been over the last kind of three years or so. She’ll look at you, and she’ll wanna communicate and tell you stuff but just can’t. So, definitely an increase in frustration.* |

1. Two other caregivers within cohort 4 (PI:1010 & PI:1204) also said that frustration and challenging behaviors used to be an issue for their child, but have decreased as their child’s ability has increased.

### Medical Issues & Communication Ability

Across cohorts, caregivers discussed how their child communicates differently when they are sick/not feeling well or experiencing certain medical issues (e.g., seizures, GI issues). Example quotes included:

- “*She has severe GI issues, and we just found out she has seizures. So, I think a lot of these issues, when they’re severe, they prevent her from trying to communicate and learn. But when she’s feeling better, that’s when I notice that the learning improves, and she tries to communicate more*.” – PI:101 (SYNGAP1-related intellectual disability)
- “*[sic] he gets so frustrated, and he might have something that hurts, and that usually leads to behaviors…”* – PI:109 (SYNGAP1-related intellectual disability)
- “*We have had periods where he wasn’t doing well, and where he if you want to say “regressed”. But it was because something was going on with him. It was either an illness or it was a reaction to a medication. During those times, he lost everything. He wasn’t hardly interacting with us at all. So, his physical interactions, his eye gaze, everything was gone... But when he is doing well, then his eye gaze is pretty spot on*.” – PI:203 (STXBP1-related disorders)
- “*But mainly, he cries, he pulls at his stomach because he has stomach issues and when it gets really bad, he’ll start hitting us and biting us and just kind of being in our face trying to figure out how to tell us “This is bothering me*.” – PI:307 (Hunter syndrome)
- “*Because I feel, when he gets sick, his communication goes down*.” – PI:401 (PMS)
- “*If she’s having a bad seizure day or she’s really tired, that level of engagement’s not there*.” –PI:608 (SCN2A-related disorder)
- “*More of the communication-based stuff with [child’s name] is more due to discomfort or like gastro-related things, like when his stomach is upset, or those kinds of things. He’s more apt to have a sense of communication when he’s uncomfortable… So, like groaning. Increased agitation and fidgeting type of stuff like that*.” – PI:903 (SGS)
- *“…her type of epilepsy is occipital lobe epilepsy…and on days when she’s going to have a seizure, her language – I mean, that’s a sign that we know she’s going to have a seizure – it’s incredible. She’s conversation, she’s stringing together five-, six- – word sentences – It’s crazy. And then, she has a seizure…”* – PI:1001 (BBSOAS)

Some caregivers describe lower frequencies of successful communication behaviors or regressions during times of illness; whereas, other caregivers report higher levels of behaviors that they interpret as indicating pain/discomfort. One caregiver of a child with BBSOAS reported higher levels of communication ability prior to a seizure. This caregiver (PI:1001) also said, “*So, her seizures take place at night – like, in the middle of the night. So – sometimes, the next day, she’s tired out and she doesn’t talk as much, but then, other times, she wakes up and she’s perfectly fine and is talking just as frequent as she was the day before the seizure.”* In addition, some caregivers report higher levels of challenging/frustration behaviors during an illness.

### Mood & Communication

Two caregivers of children with BBSOAS (PI:1001 & PI:1004) mentioned their child’s mood can impact their communication; particularly, whether their child will follow through with instructions given by the parent. PI:1004 also felt sleep impacted their child’s communication, saying, “*However, I almost feel like it [communication] can still be affected by just what type of day she’s having. If she hasn’t gotten enough sleep or she’s just kind of frustrated or she’s constipated or has something internally going on, it does inhibit her ability to really kinda like – or at least what we know, the gestures that she knows. It kinda inhibits her ability to do that…”*

Another caregiver (PI:1211) of a child with HAFOUS also mentioned there are days when their child’s communication is “off” but could not pinpoint the cause. For example, they said, “*We do notice in her is sometimes she has her switch off... Like, she’s off…articulation is even worse in those periods. Like, she struggles. She’s aloof. So, we always say like, “Oh, I think she woke up with her switch off today. Like, she’s not functioning like she was functioning yesterday…it can be one day, it can be a few days. They happen and we’re like, “Ah, there’s something going on.” It could be like one day or it could be like a few days that she’s like that…I haven’t figured it out if she’s sick or anything. It’s just like, she wakes up, and it’s not like she’s totally not there. It’s just like it’s harder for her to comprehend. It’s harder for her to express herself. She has no patience when we don't understand. It’s harder. You know, like, when you see a person is having a rough day, kind of. We think it’s more like an on/off switch…There could be a month that she’s fine, but then, suddenly we have that…So, maybe once a month, but not necessarily every month*.”
